# Supplementary figures and images for: SARS-CoV-2 nsp16 is regulated by host E3 ubiquitin ligases, UBR5 and MARCHF7 (part 1 of 3)
Source: eLife. 2025 May 13;13:RP102277. doi: 10.7554/eLife.102277 (PMC12074641; doi:10.7554/eLife.102277)

Figure 1A

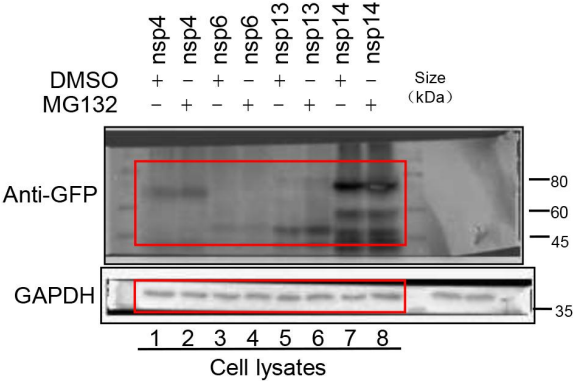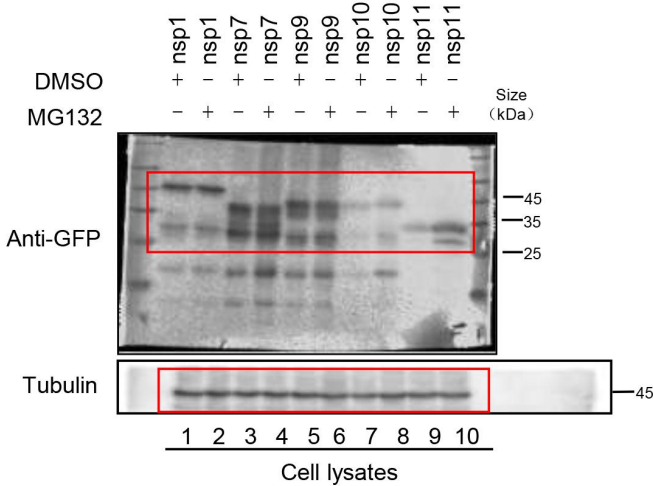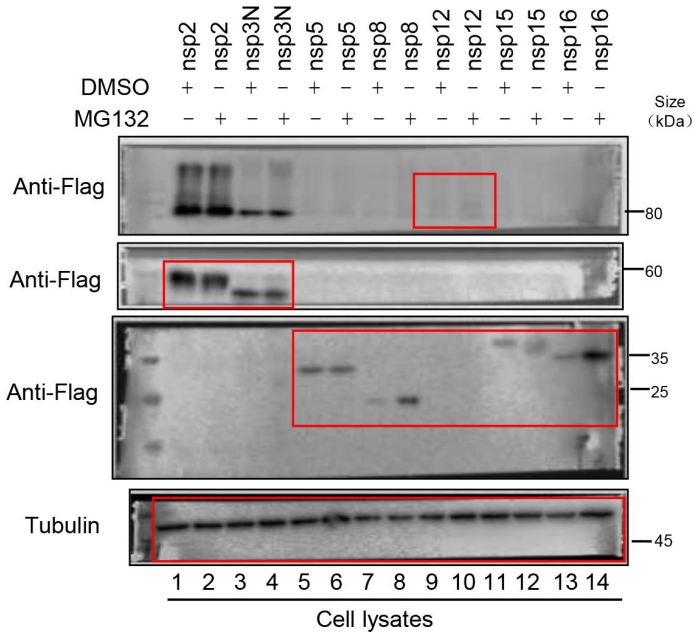

Supplement: Figure 1—source data 1. [file elife-102277-fig1-data1.zip › Figure 1-source data 1/Figure 1A-source data 1 .pdf]

Figure 1B

Fig.1B

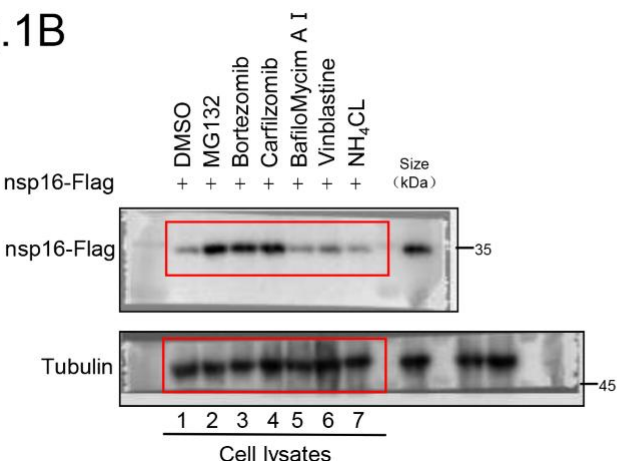

Supplement: Figure 1—source data 1. [file elife-102277-fig1-data1.zip › Figure 1-source data 1/Figure 1B-source data 1.pdf]

Figure 1C

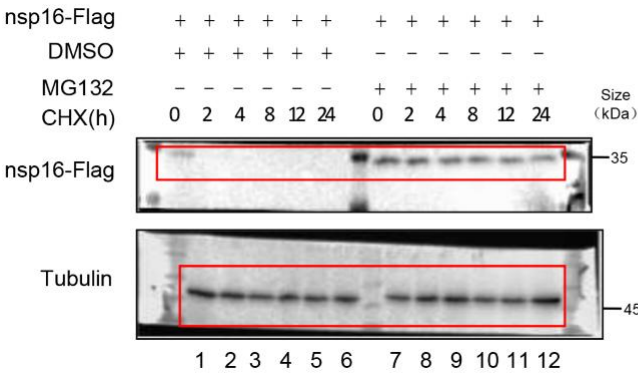

Supplement: Figure 1—source data 1. [file elife-102277-fig1-data1.zip › Figure 1-source data 1/Figure 1C-source data 1.pdf]

Figure 1E

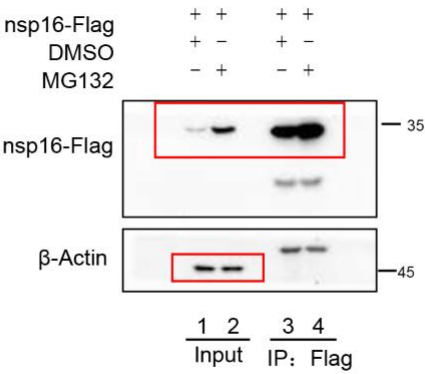

Supplement: Figure 1—source data 1. [file elife-102277-fig1-data1.zip › Figure 1-source data 1/Figure 1E-source data 1.pdf]

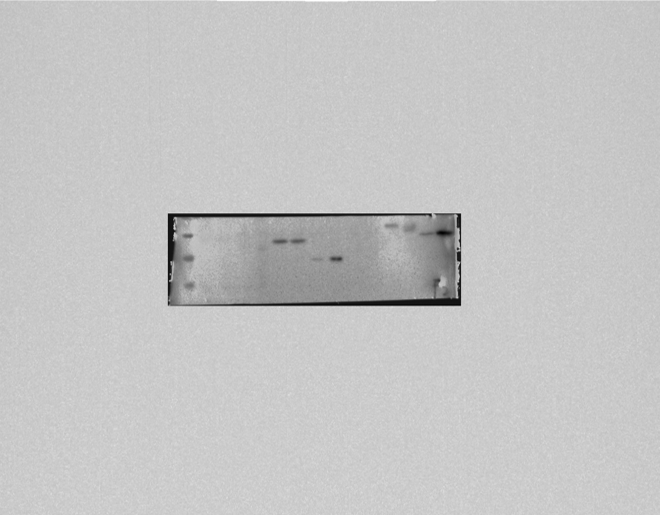

Supplement: Figure 1—source data 2. [file elife-102277-fig1-data2.zip › Figure 1-source data 2/Figure 1A-source data 2/anti-Flag nsp.tif]

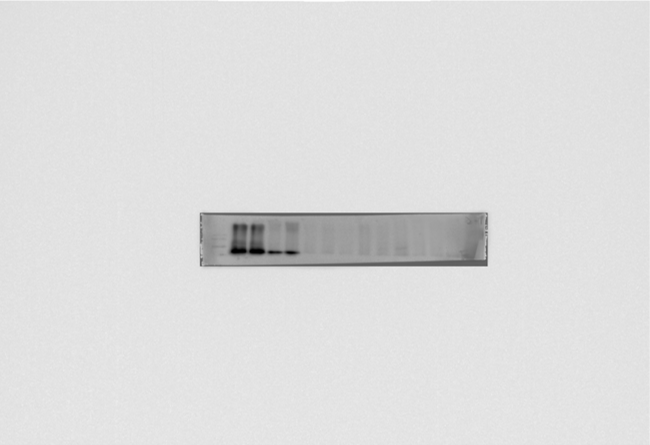

Supplement: Figure 1—source data 2. [file elife-102277-fig1-data2.zip › Figure 1-source data 2/Figure 1A-source data 2/anti-Flag nsp12.tif]

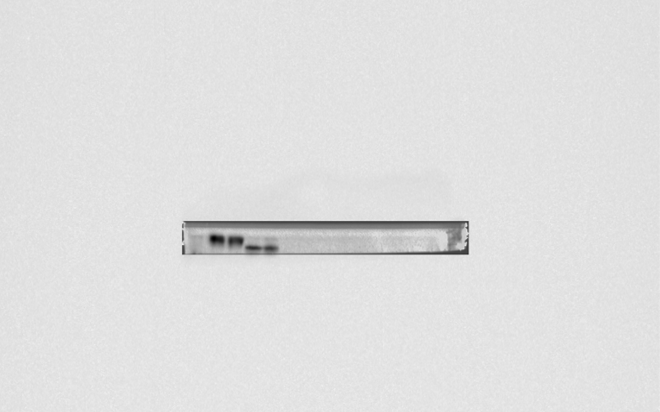

Supplement: Figure 1—source data 2. [file elife-102277-fig1-data2.zip › Figure 1-source data 2/Figure 1A-source data 2/anti-Flag nsp2 3N.tif]

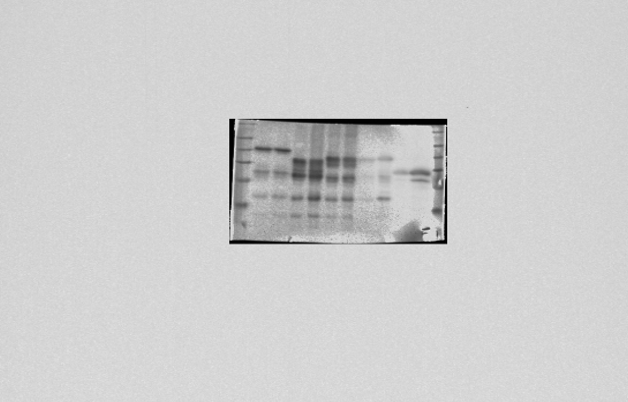

Supplement: Figure 1—source data 2. [file elife-102277-fig1-data2.zip › Figure 1-source data 2/Figure 1A-source data 2/anti-GFP nsp-2.tif]

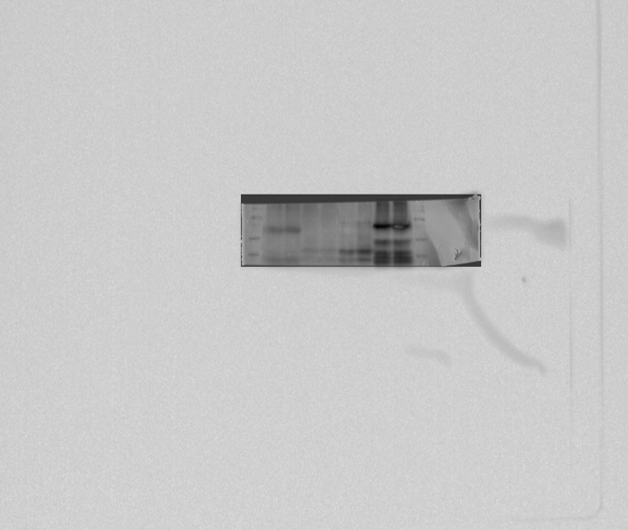

Supplement: Figure 1—source data 2. [file elife-102277-fig1-data2.zip › Figure 1-source data 2/Figure 1A-source data 2/anti-GFP nsp.tif]

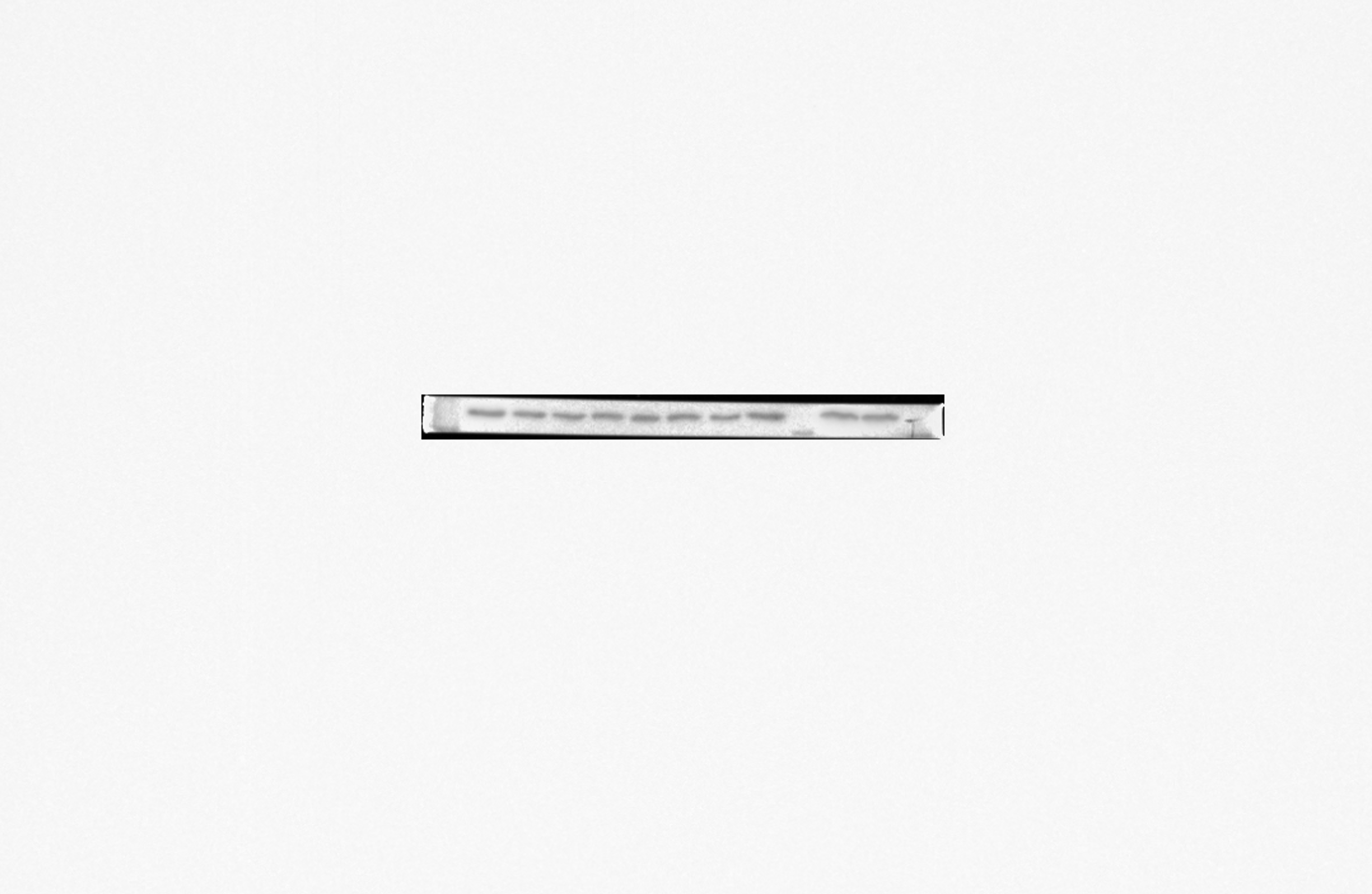

Supplement: Figure 1—source data 2. [file elife-102277-fig1-data2.zip › Figure 1-source data 2/Figure 1A-source data 2/GAPDH.tif]

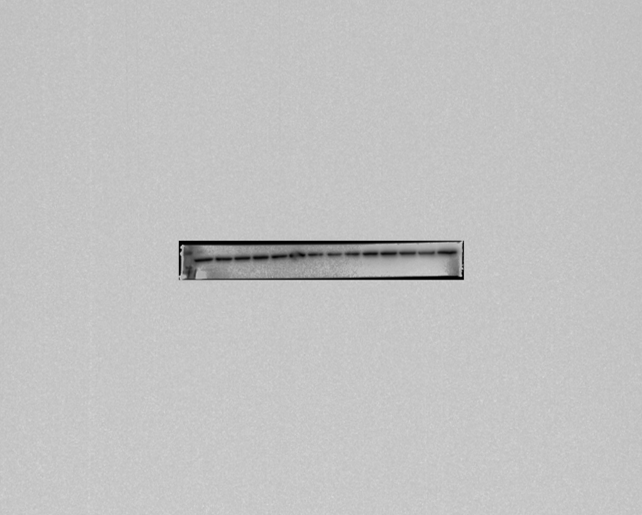

Supplement: Figure 1—source data 2. [file elife-102277-fig1-data2.zip › Figure 1-source data 2/Figure 1A-source data 2/Tubulin-2.tif]

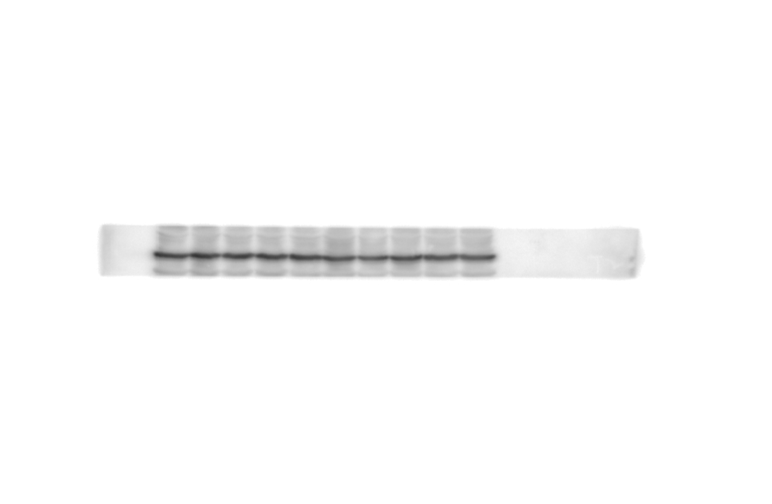

Supplement: Figure 1—source data 2. [file elife-102277-fig1-data2.zip › Figure 1-source data 2/Figure 1A-source data 2/Tubulin.tif]

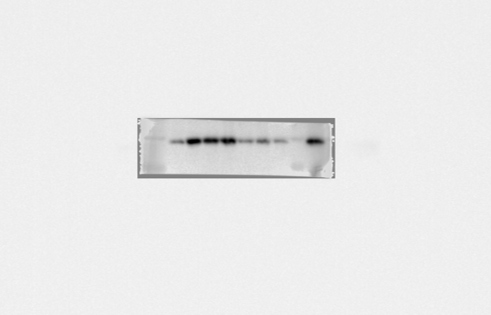

Supplement: Figure 1—source data 2. [file elife-102277-fig1-data2.zip › Figure 1-source data 2/Figure 1B-source data 2/anti-Flag nsp16.tif]

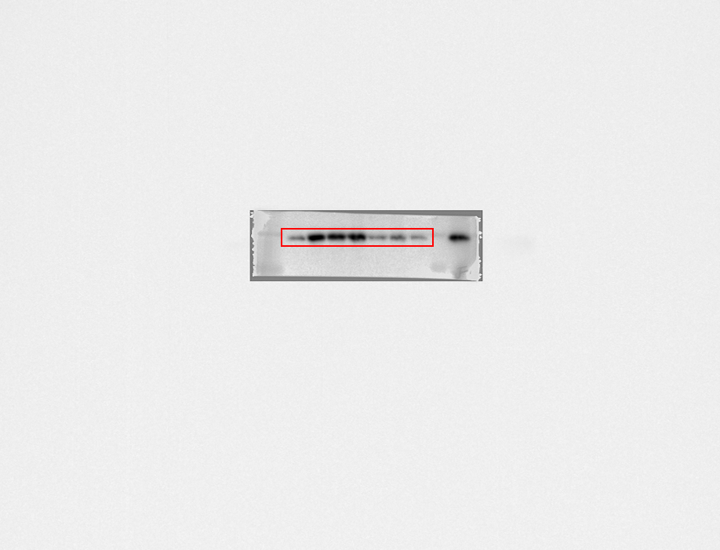

Supplement: Figure 1—source data 2. [file elife-102277-fig1-data2.zip › Figure 1-source data 2/Figure 1B-source data 2/anti-Flag nsp16_2.tif]

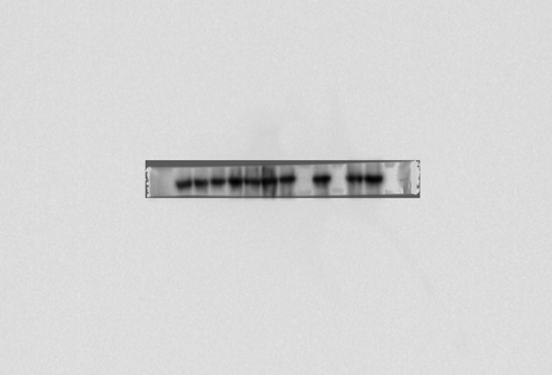

Supplement: Figure 1—source data 2. [file elife-102277-fig1-data2.zip › Figure 1-source data 2/Figure 1B-source data 2/Tubulin.tif]

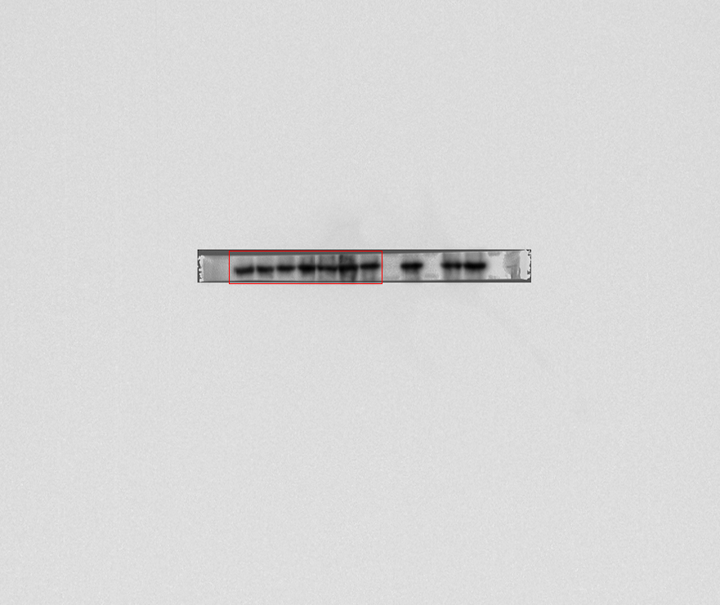

Supplement: Figure 1—source data 2. [file elife-102277-fig1-data2.zip › Figure 1-source data 2/Figure 1B-source data 2/Tubulin_2.tif]

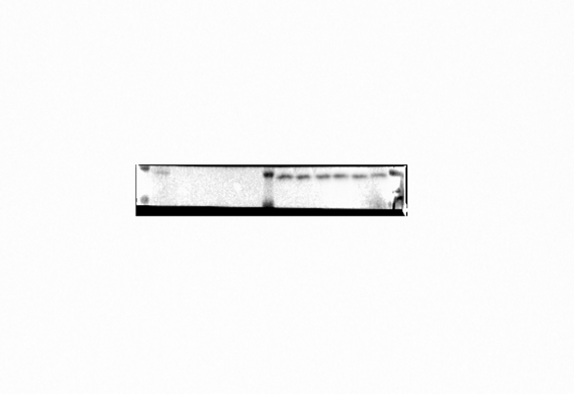

Supplement: Figure 1—source data 2. [file elife-102277-fig1-data2.zip › Figure 1-source data 2/Figure 1C-source data 2/anti-Flag nsp16.tif]

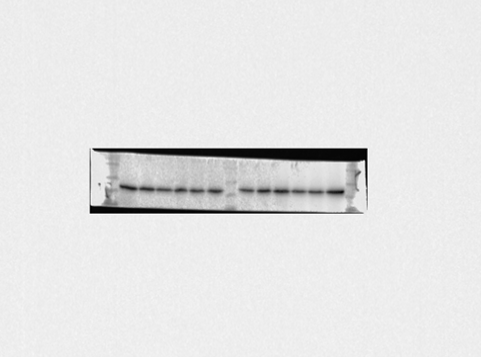

Supplement: Figure 1—source data 2. [file elife-102277-fig1-data2.zip › Figure 1-source data 2/Figure 1C-source data 2/Tubulin.tif]

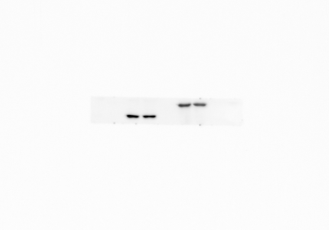

Supplement: Figure 1—source data 2. [file elife-102277-fig1-data2.zip › Figure 1-source data 2/Figure 1E-source data 2/anti-actin.tif]

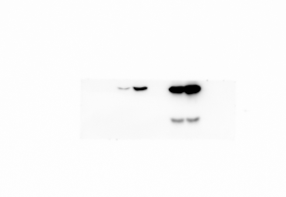

Supplement: Figure 1—source data 2. [file elife-102277-fig1-data2.zip › Figure 1-source data 2/Figure 1E-source data 2/anti-Flag nsp16.tif]

Figure 2A

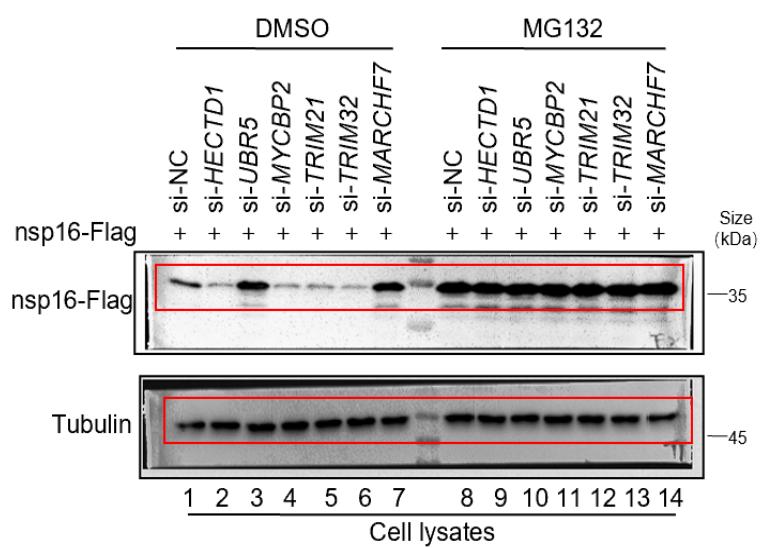

Supplement: Figure 2—source data 1. [file elife-102277-fig2-data1.zip › Figure 2-source data 1/Figure 2A-source data 1 .pdf]

Figure 2B

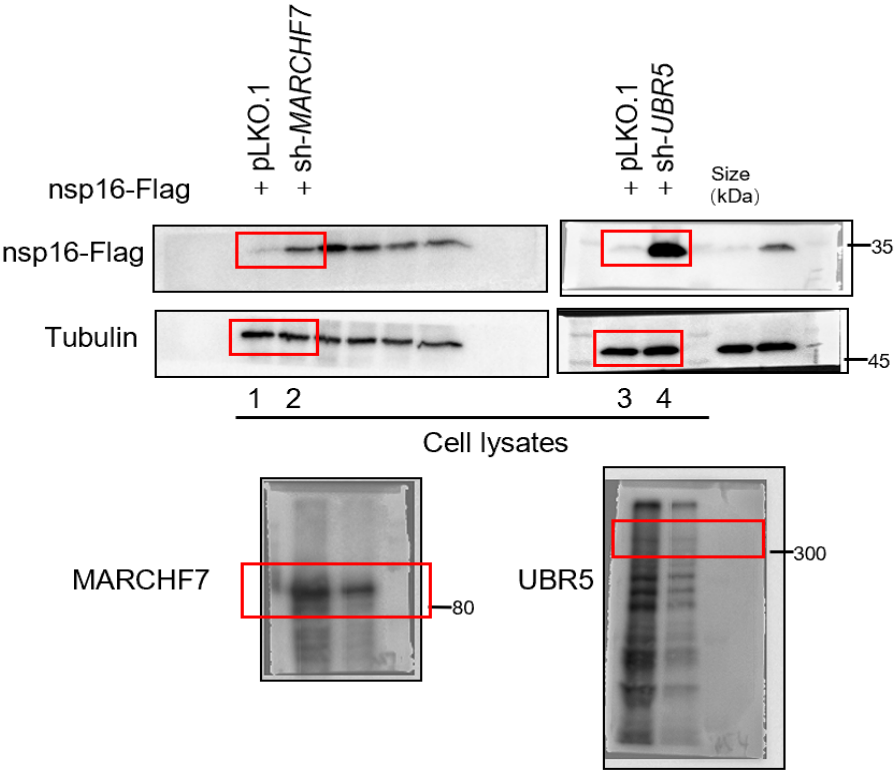

Supplement: Figure 2—source data 1. [file elife-102277-fig2-data1.zip › Figure 2-source data 1/Figure 2B-source data 1.pdf]

Figure 2C

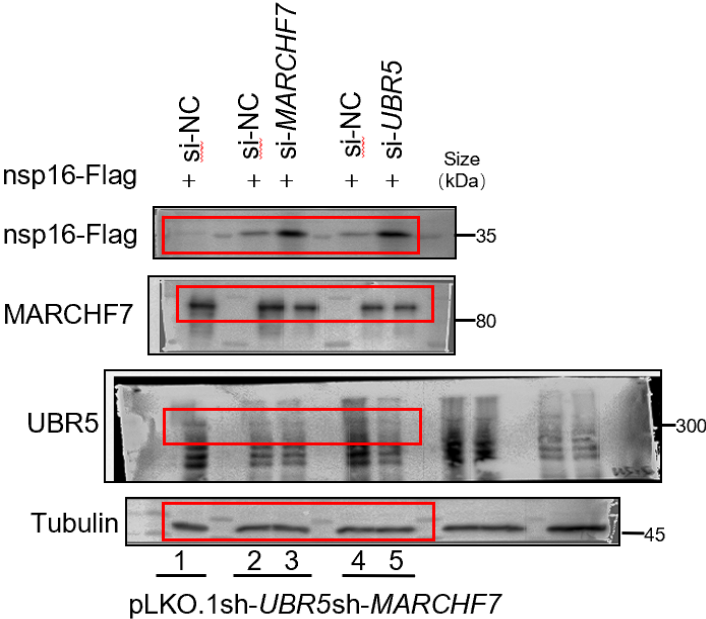

Supplement: Figure 2—source data 1. [file elife-102277-fig2-data1.zip › Figure 2-source data 1/Figure 2C-source data 1.pdf]

Figure 2D

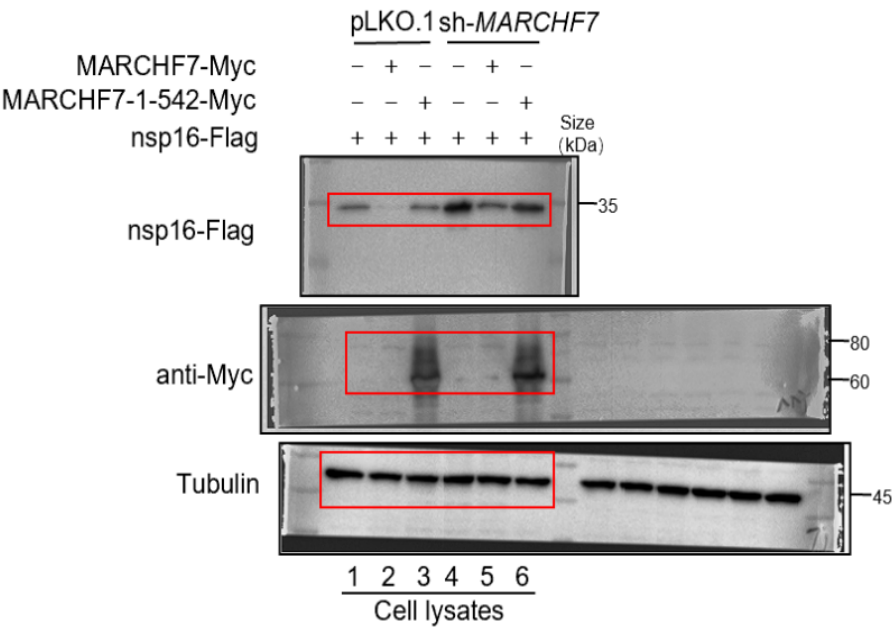

Supplement: Figure 2—source data 1. [file elife-102277-fig2-data1.zip › Figure 2-source data 1/Figure 2D-source data 1.pdf]

Figure 2E

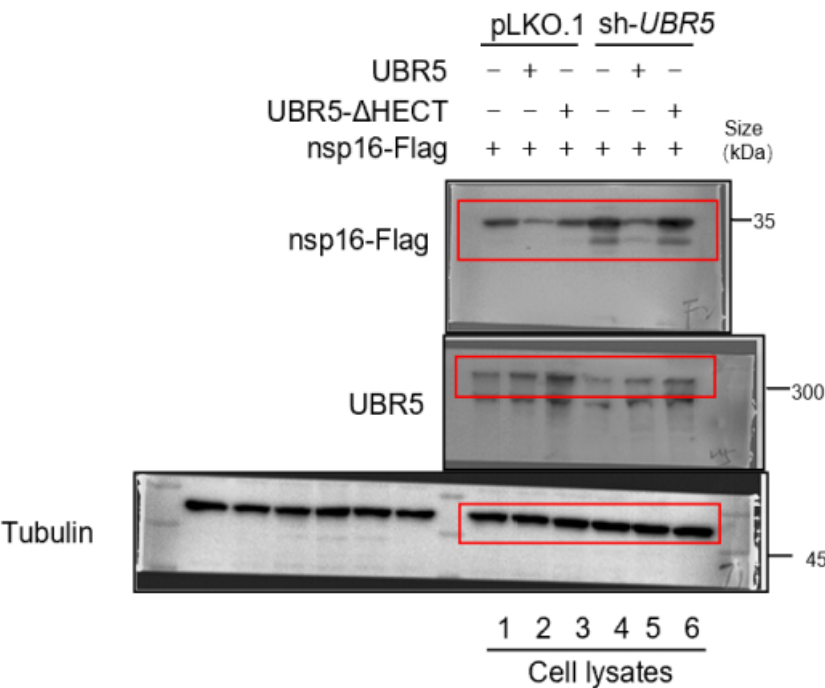

Supplement: Figure 2—source data 1. [file elife-102277-fig2-data1.zip › Figure 2-source data 1/Figure 2E-source data 1.pdf]

Figure 2—figure supplement 1A

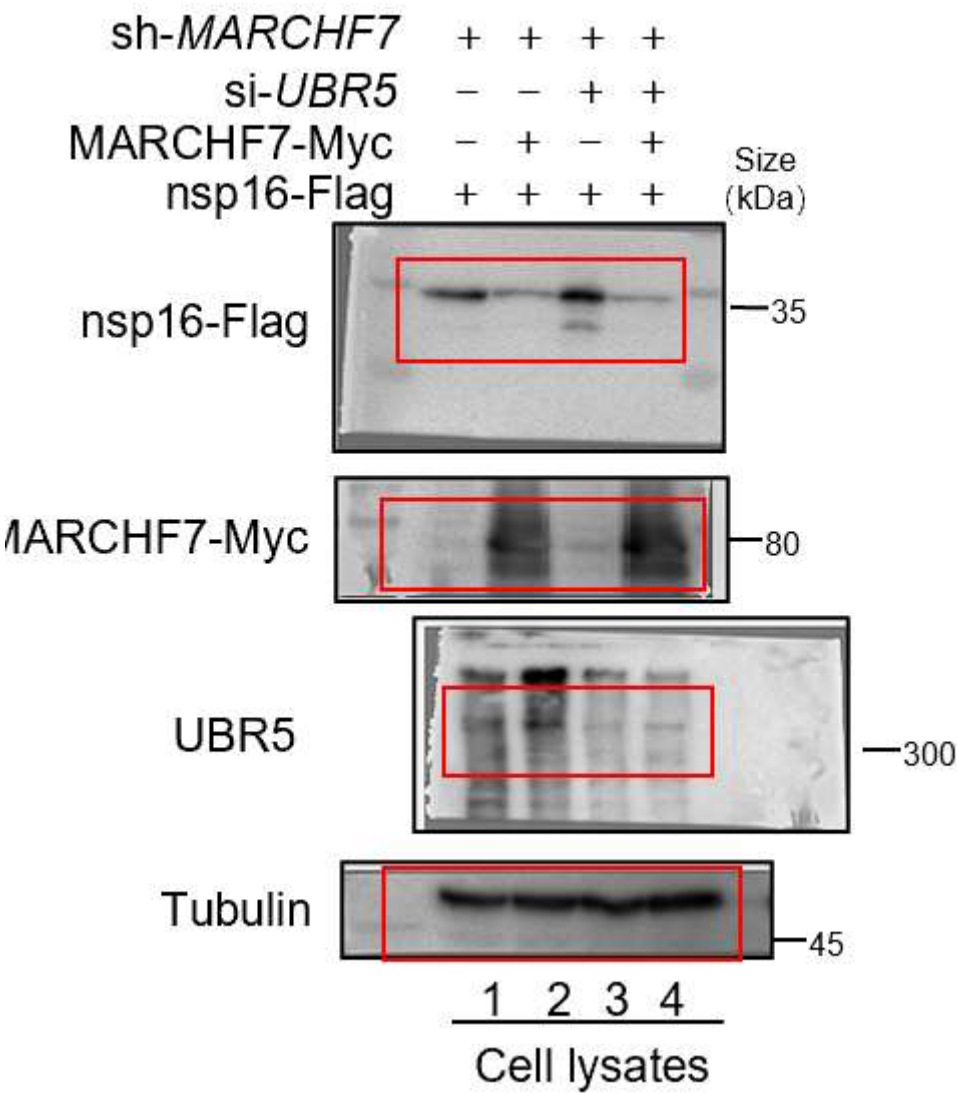

Supplement: Figure 2—figure supplement 1—source data 1. [file elife-102277-fig2-figsupp1-data1.zip › Figure 2—figure supplement 1-source data 1/Figure 2—figure supplement 1A-source data 1.pdf]

Figure 2—figure supplement 1B

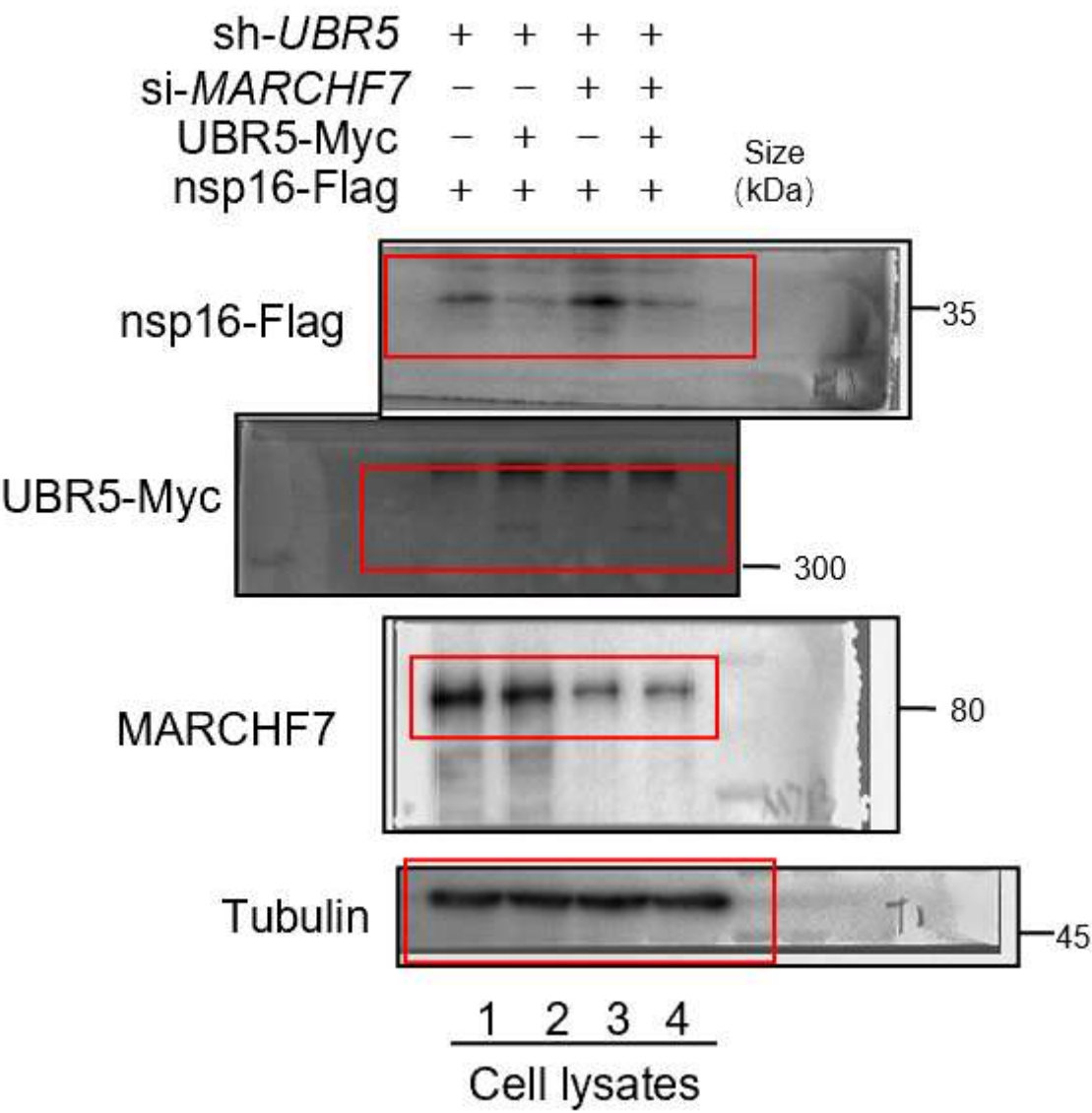

Supplement: Figure 2—figure supplement 1—source data 1. [file elife-102277-fig2-figsupp1-data1.zip › Figure 2—figure supplement 1-source data 1/Figure 2—figure supplement 1B-source data 1.pdf]

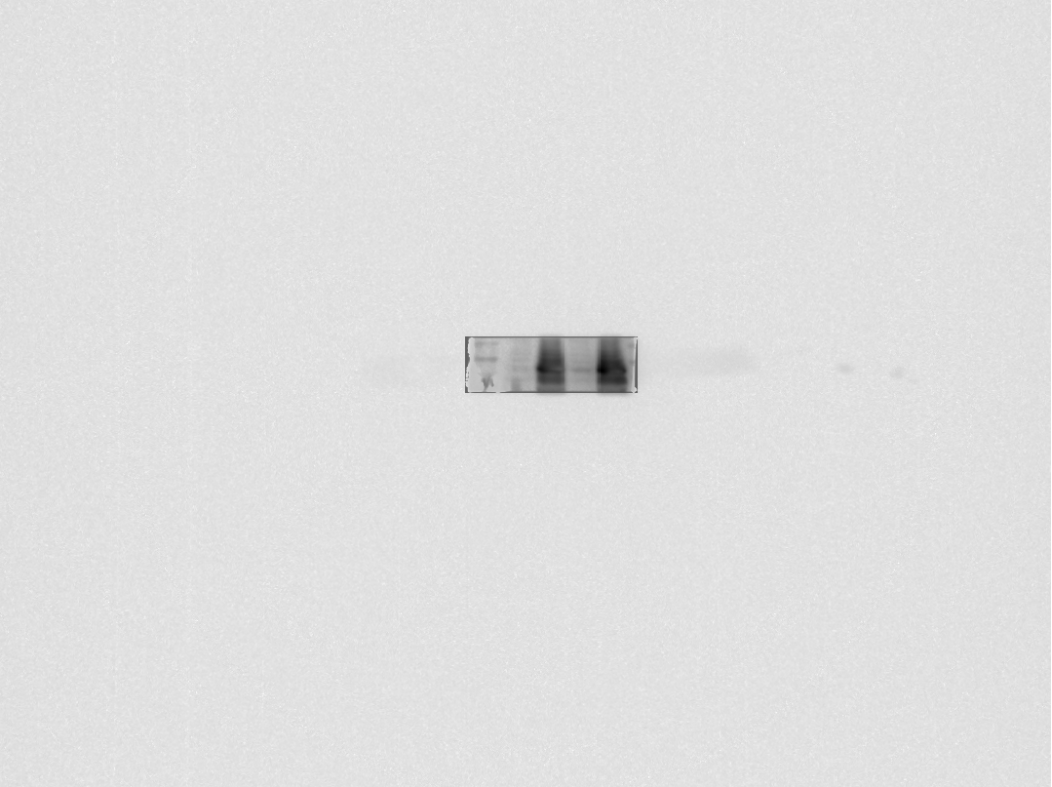

Supplement: Figure 2—figure supplement 1—source data 2. [file elife-102277-fig2-figsupp1-data2.zip › Figure 2—figure supplement 1-source data 2/Figure 2—figure supplement 1A-source data 2/MARCHF7.tif]

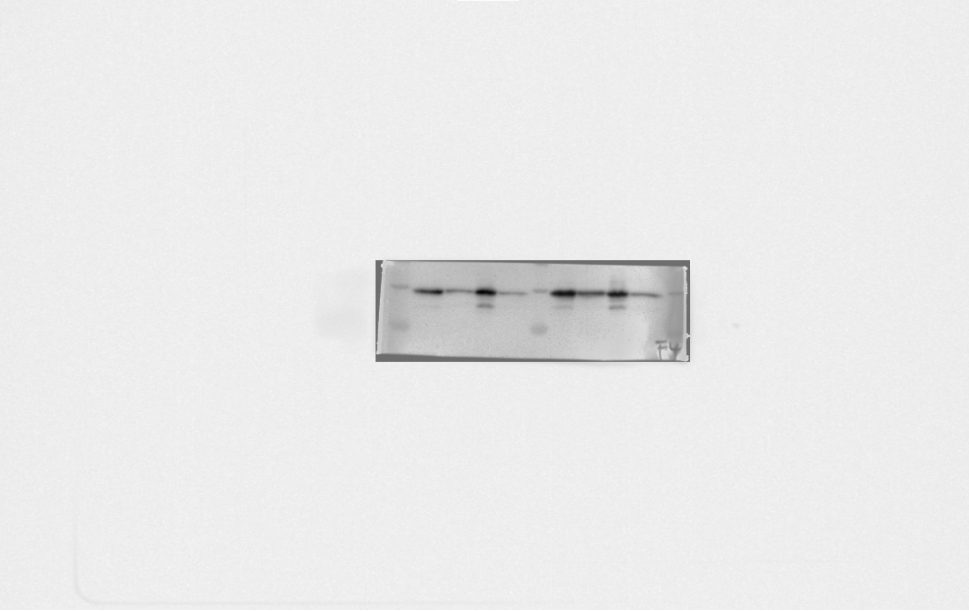

Supplement: Figure 2—figure supplement 1—source data 2. [file elife-102277-fig2-figsupp1-data2.zip › Figure 2—figure supplement 1-source data 2/Figure 2—figure supplement 1A-source data 2/nsp16-Flag.tif]

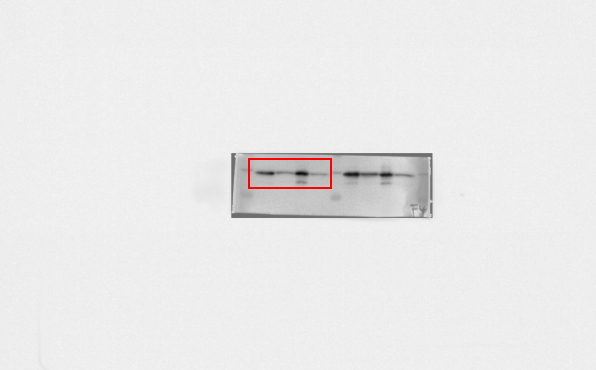

Supplement: Figure 2—figure supplement 1—source data 2. [file elife-102277-fig2-figsupp1-data2.zip › Figure 2—figure supplement 1-source data 2/Figure 2—figure supplement 1A-source data 2/nsp16-Flag_2.tif]

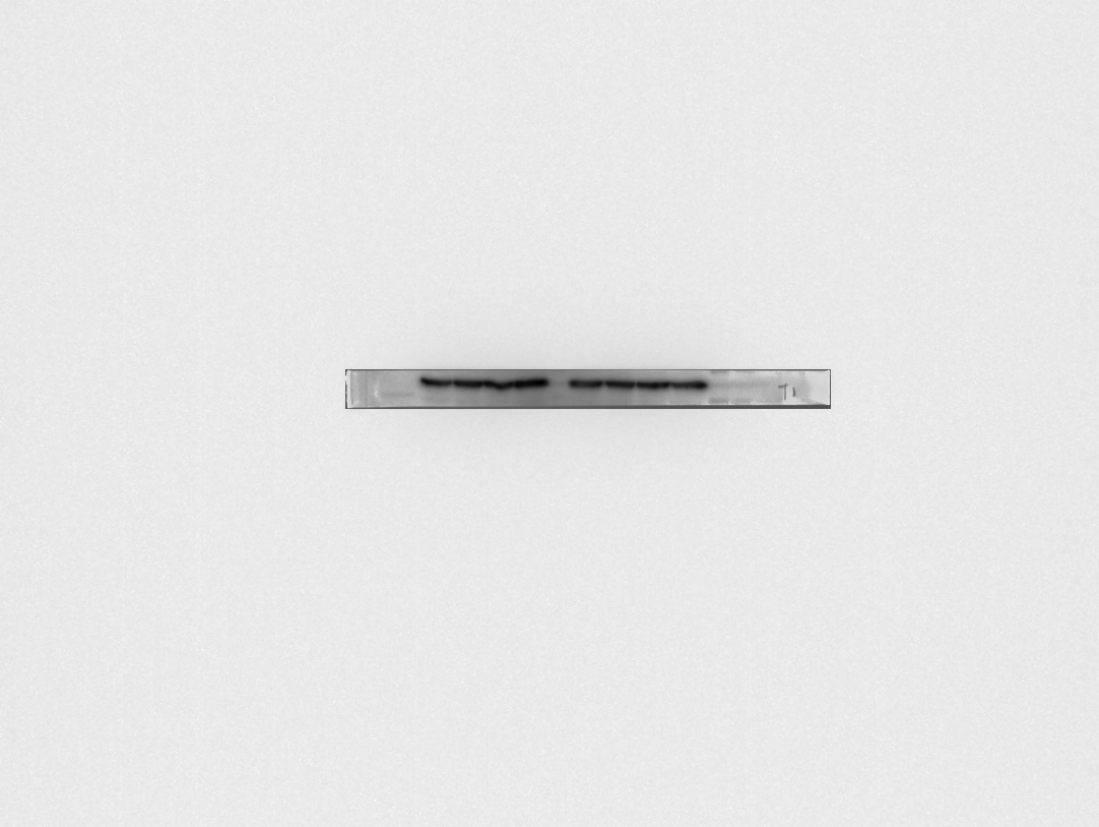

Supplement: Figure 2—figure supplement 1—source data 2. [file elife-102277-fig2-figsupp1-data2.zip › Figure 2—figure supplement 1-source data 2/Figure 2—figure supplement 1A-source data 2/Tubulin.tif]

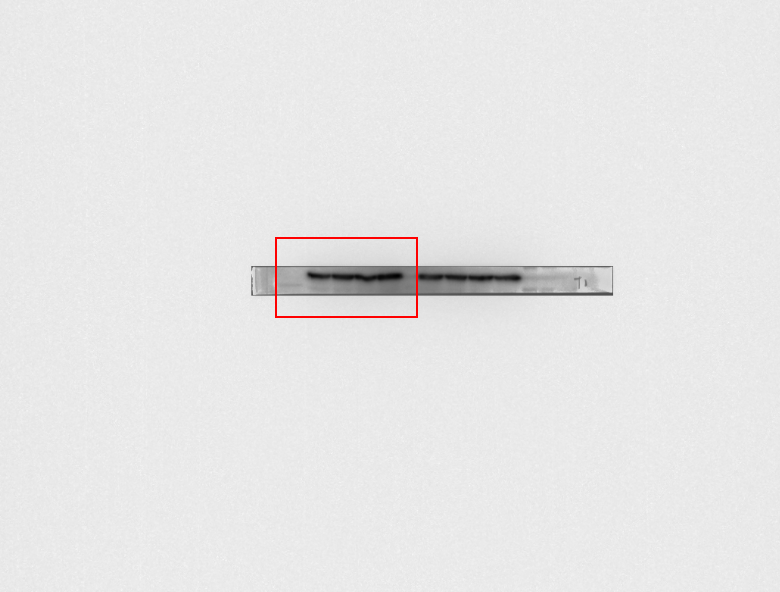

Supplement: Figure 2—figure supplement 1—source data 2. [file elife-102277-fig2-figsupp1-data2.zip › Figure 2—figure supplement 1-source data 2/Figure 2—figure supplement 1A-source data 2/Tubulin_2.tif]

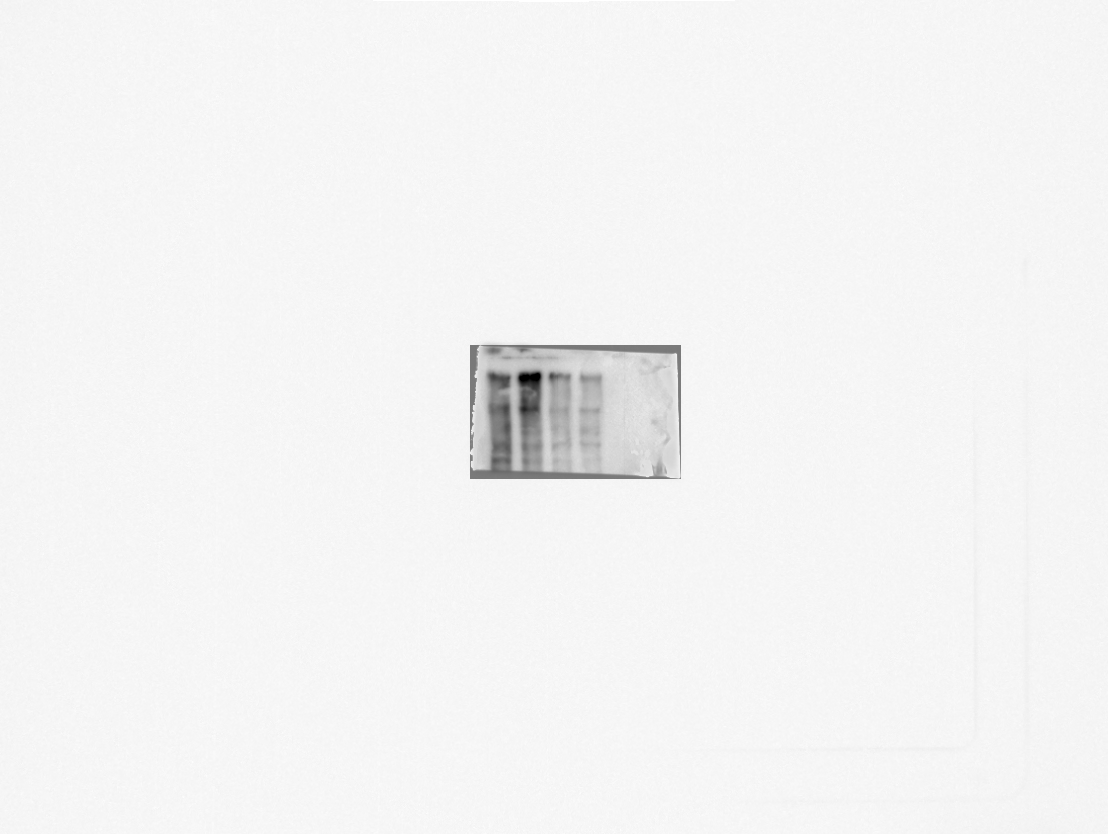

Supplement: Figure 2—figure supplement 1—source data 2. [file elife-102277-fig2-figsupp1-data2.zip › Figure 2—figure supplement 1-source data 2/Figure 2—figure supplement 1A-source data 2/UBR5.tif]

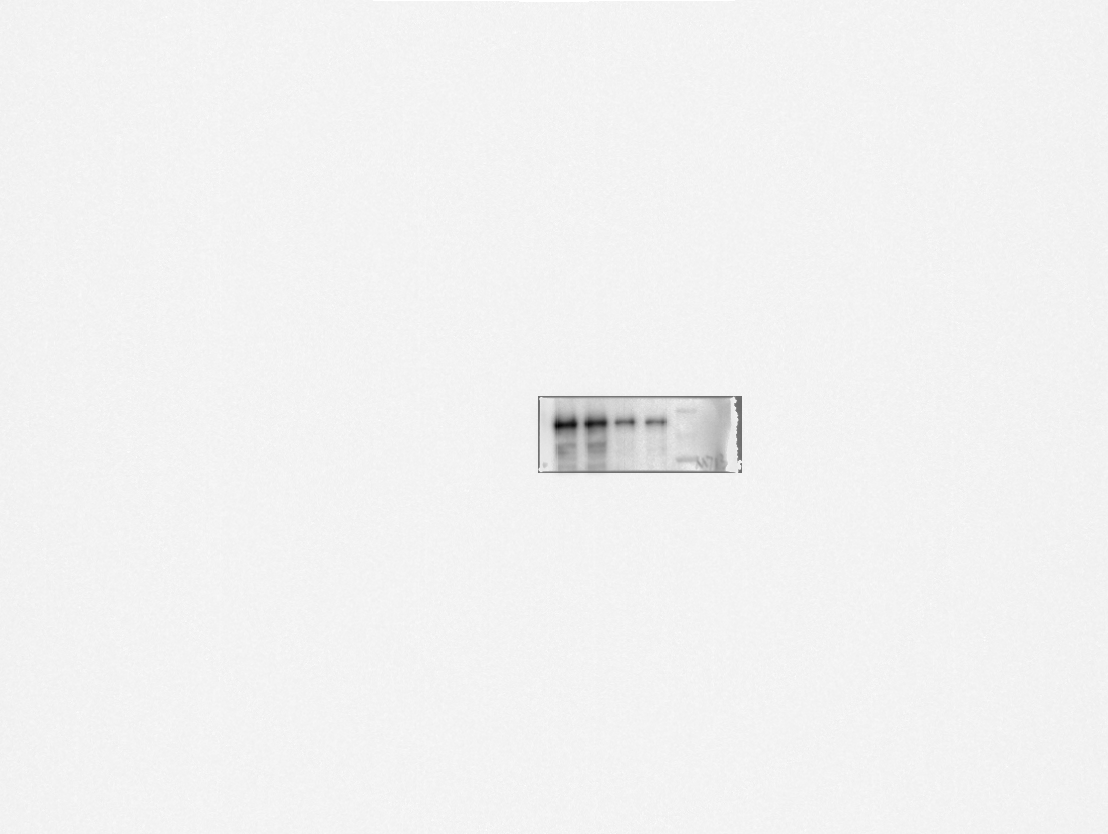

Supplement: Figure 2—figure supplement 1—source data 2. [file elife-102277-fig2-figsupp1-data2.zip › Figure 2—figure supplement 1-source data 2/Figure 2—figure supplement 1B-source data 2/MARCHF7.tif]

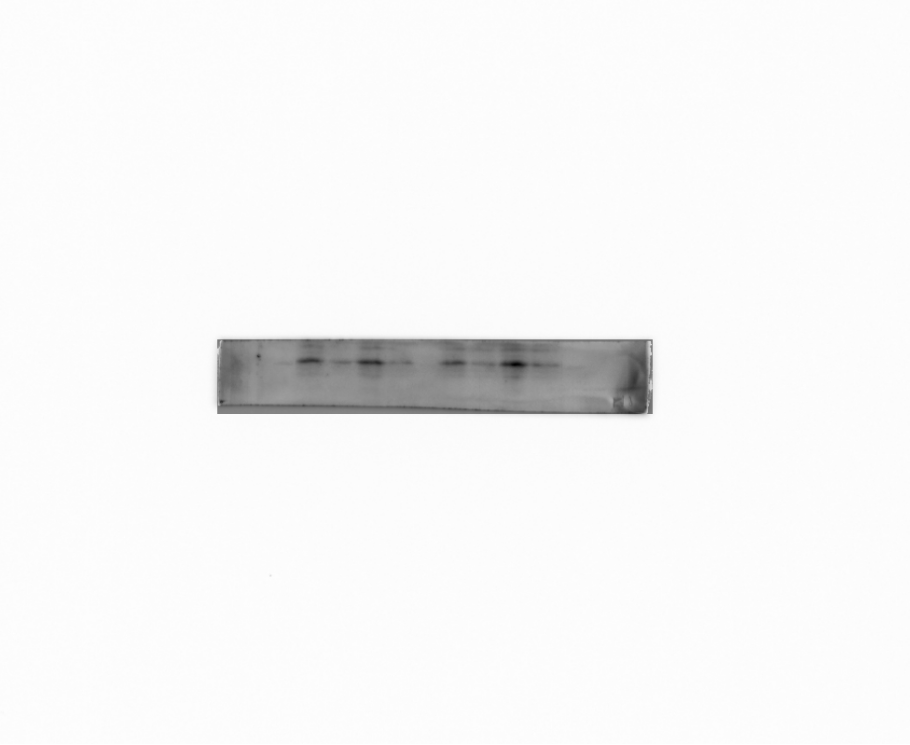

Supplement: Figure 2—figure supplement 1—source data 2. [file elife-102277-fig2-figsupp1-data2.zip › Figure 2—figure supplement 1-source data 2/Figure 2—figure supplement 1B-source data 2/nsp16-Flag.tif]

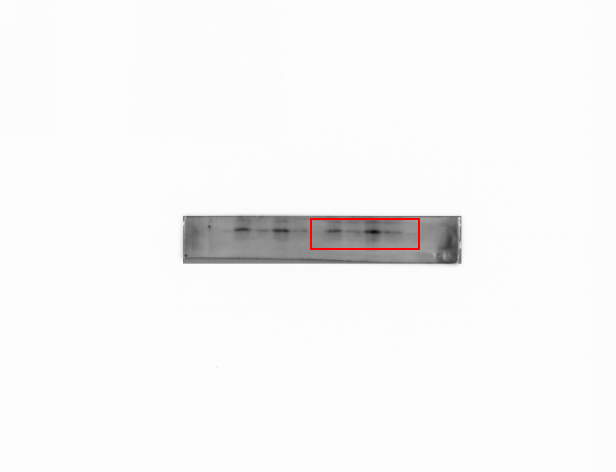

Supplement: Figure 2—figure supplement 1—source data 2. [file elife-102277-fig2-figsupp1-data2.zip › Figure 2—figure supplement 1-source data 2/Figure 2—figure supplement 1B-source data 2/nsp16-Flag_2.tif]

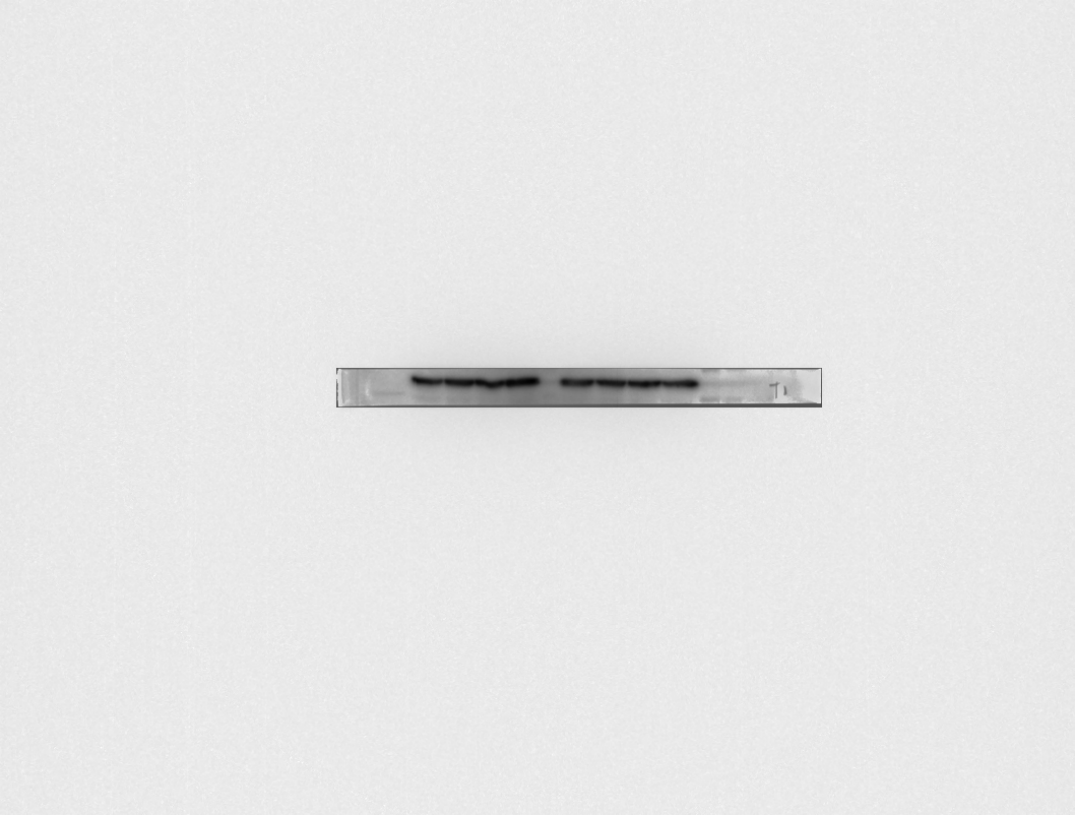

Supplement: Figure 2—figure supplement 1—source data 2. [file elife-102277-fig2-figsupp1-data2.zip › Figure 2—figure supplement 1-source data 2/Figure 2—figure supplement 1B-source data 2/Tubulin.tif]

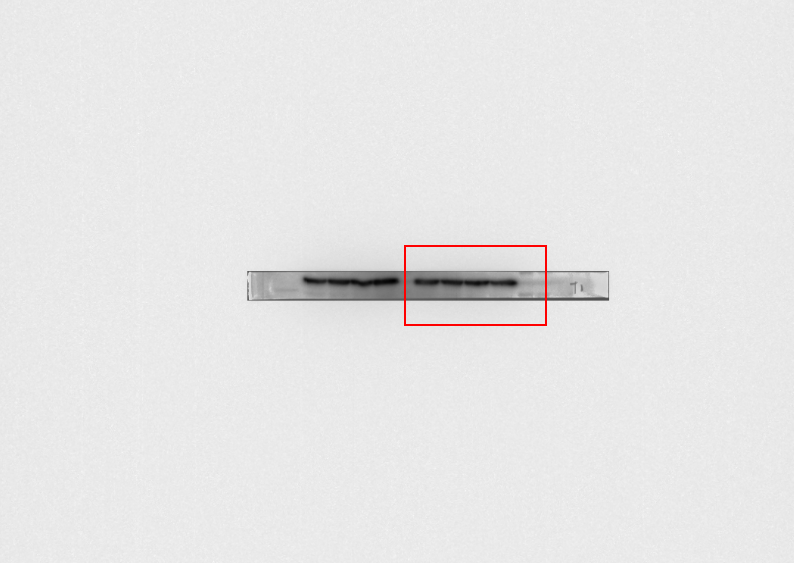

Supplement: Figure 2—figure supplement 1—source data 2. [file elife-102277-fig2-figsupp1-data2.zip › Figure 2—figure supplement 1-source data 2/Figure 2—figure supplement 1B-source data 2/Tubulin_2.tif]

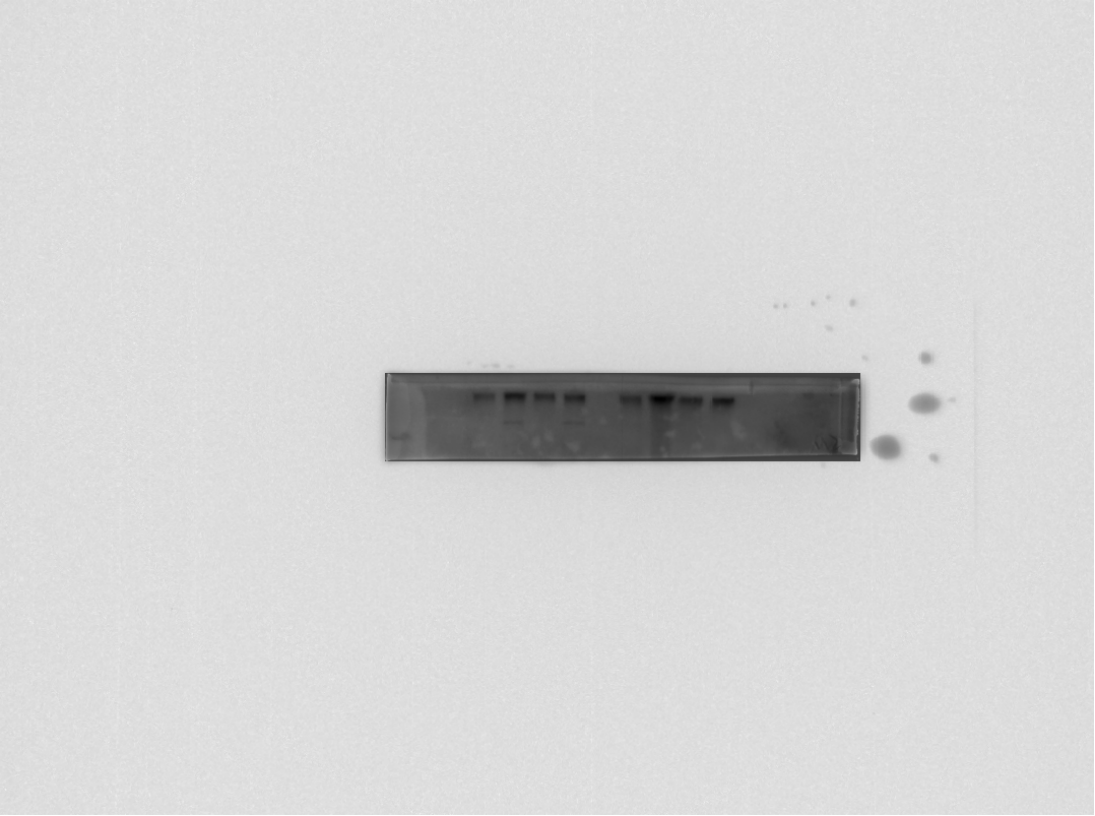

Supplement: Figure 2—figure supplement 1—source data 2. [file elife-102277-fig2-figsupp1-data2.zip › Figure 2—figure supplement 1-source data 2/Figure 2—figure supplement 1B-source data 2/UBR5.tif]

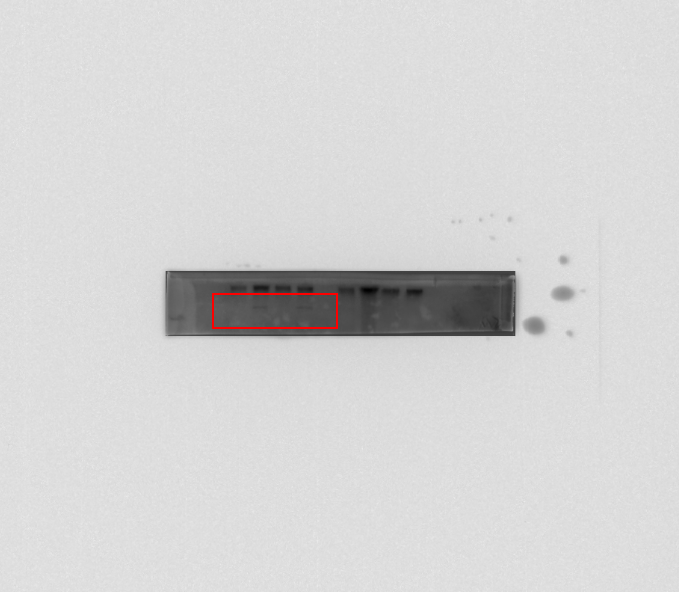

Supplement: Figure 2—figure supplement 1—source data 2. [file elife-102277-fig2-figsupp1-data2.zip › Figure 2—figure supplement 1-source data 2/Figure 2—figure supplement 1B-source data 2/UBR5_2.tif]

Figure 3A

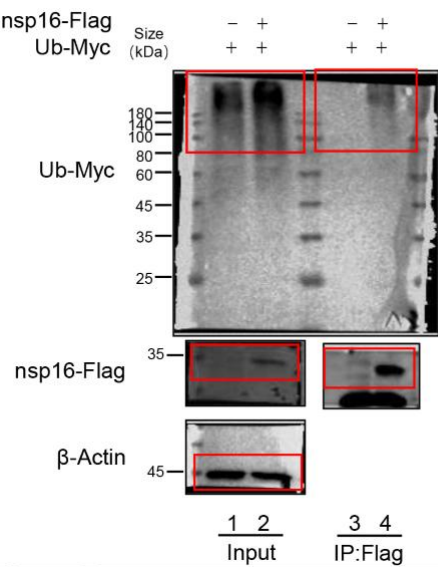

Supplement: Figure 3—source data 1. [file elife-102277-fig3-data1.zip › Figure 3-source data 1/Figure 3A-source data 1.pdf]

Figure 3B

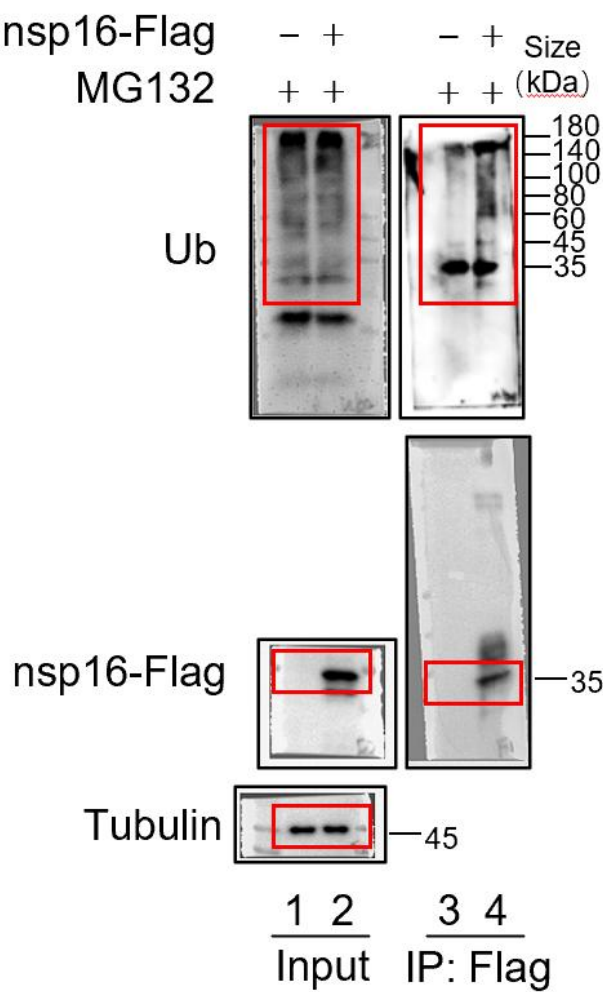

Supplement: Figure 3—source data 1. [file elife-102277-fig3-data1.zip › Figure 3-source data 1/Figure 3B-source data 1 .pdf]

Figure 3C

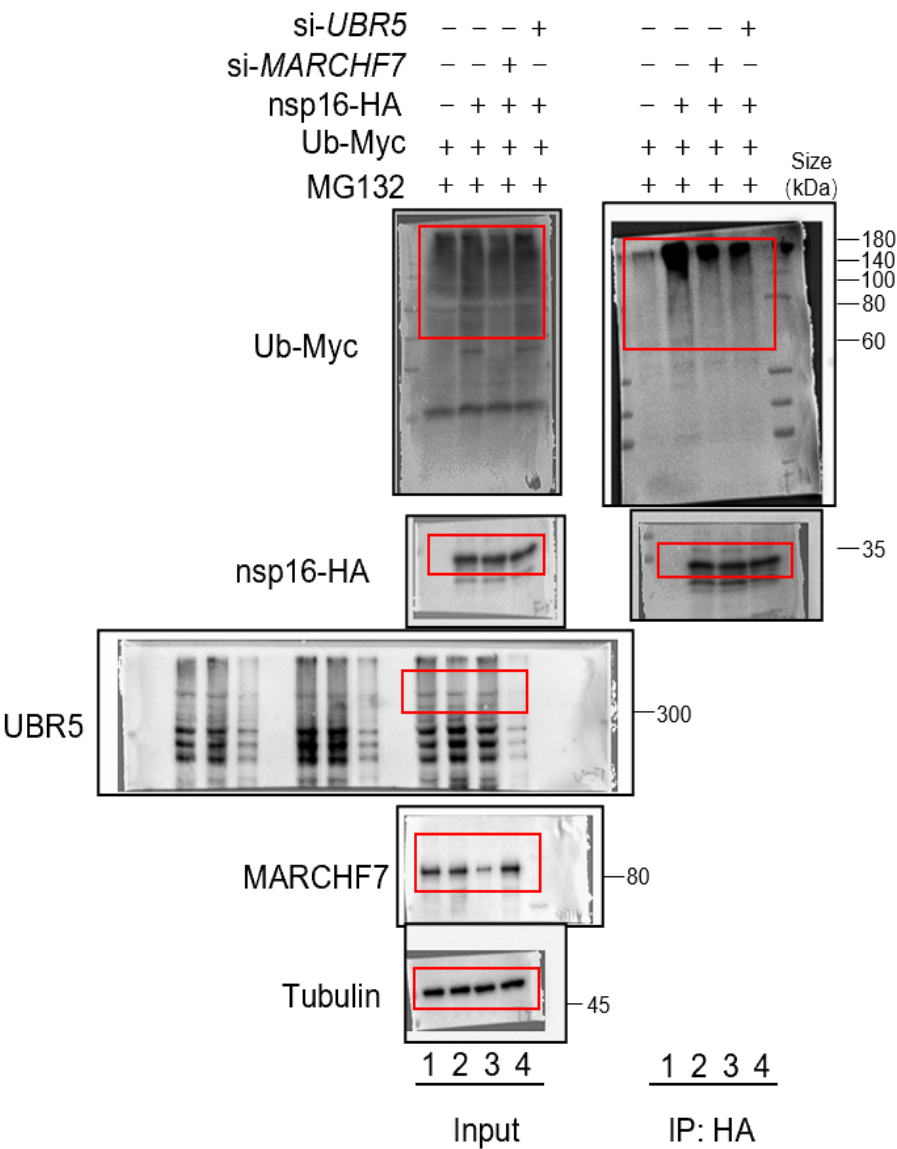

Supplement: Figure 3—source data 1. [file elife-102277-fig3-data1.zip › Figure 3-source data 1/Figure 3C-source data 1.pdf]

Figure 3D

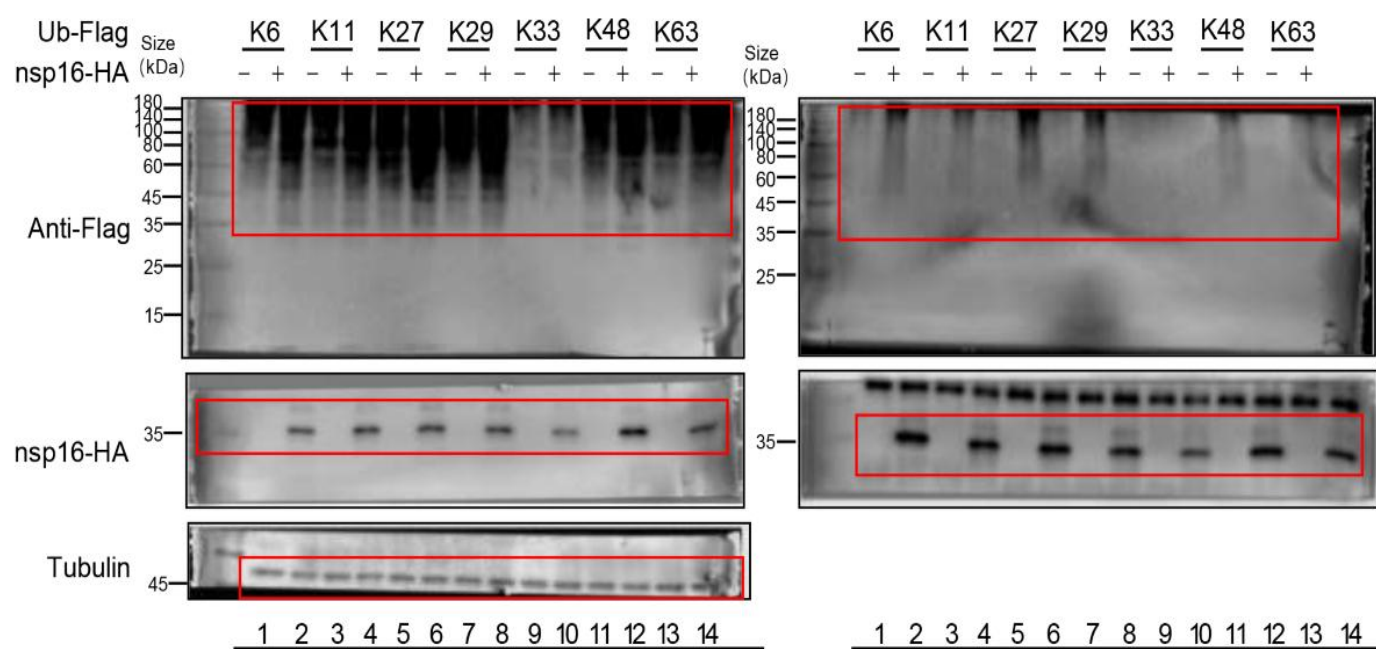

Supplement: Figure 3—source data 1. [file elife-102277-fig3-data1.zip › Figure 3-source data 1/Figure 3D-source data 1.pdf]

Figure.3E

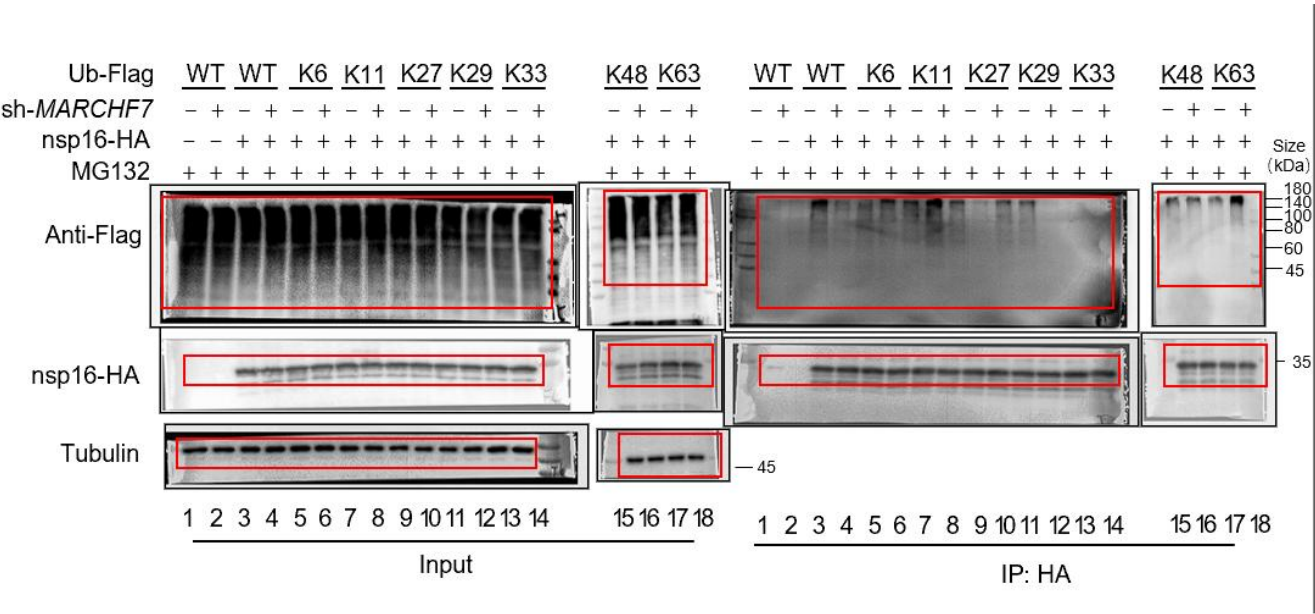

Supplement: Figure 3—source data 1. [file elife-102277-fig3-data1.zip › Figure 3-source data 1/Figure 3E-source data 1.pdf]

Figure.3F

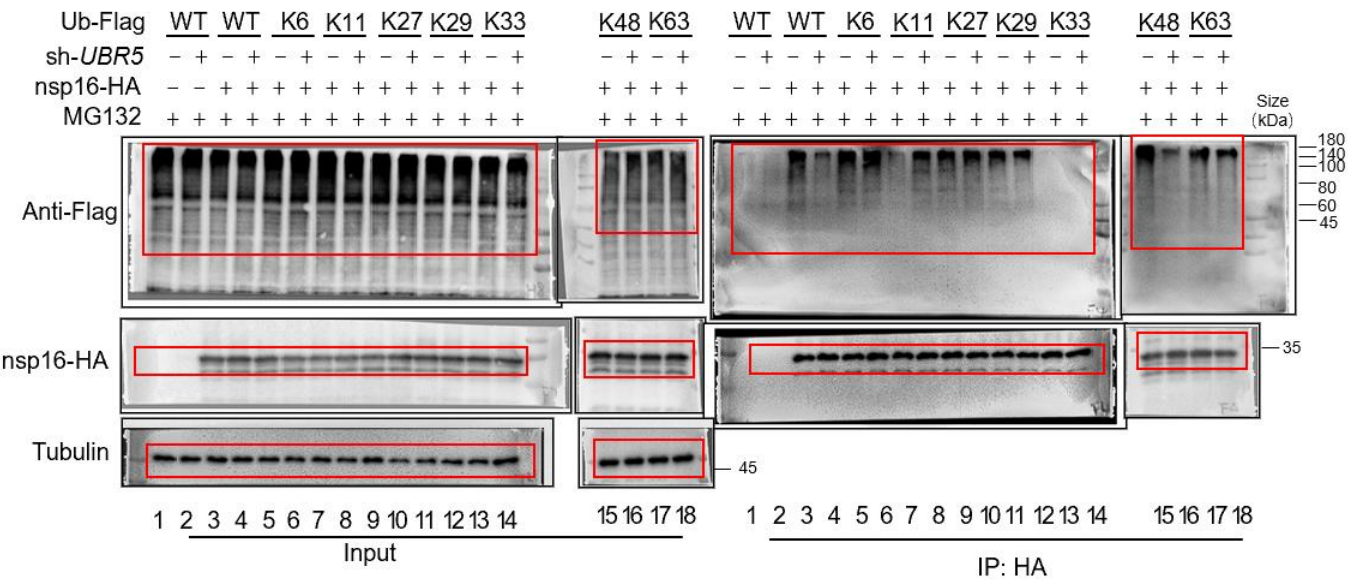

Supplement: Figure 3—source data 1. [file elife-102277-fig3-data1.zip › Figure 3-source data 1/Figure 3F-source data 1.pdf]

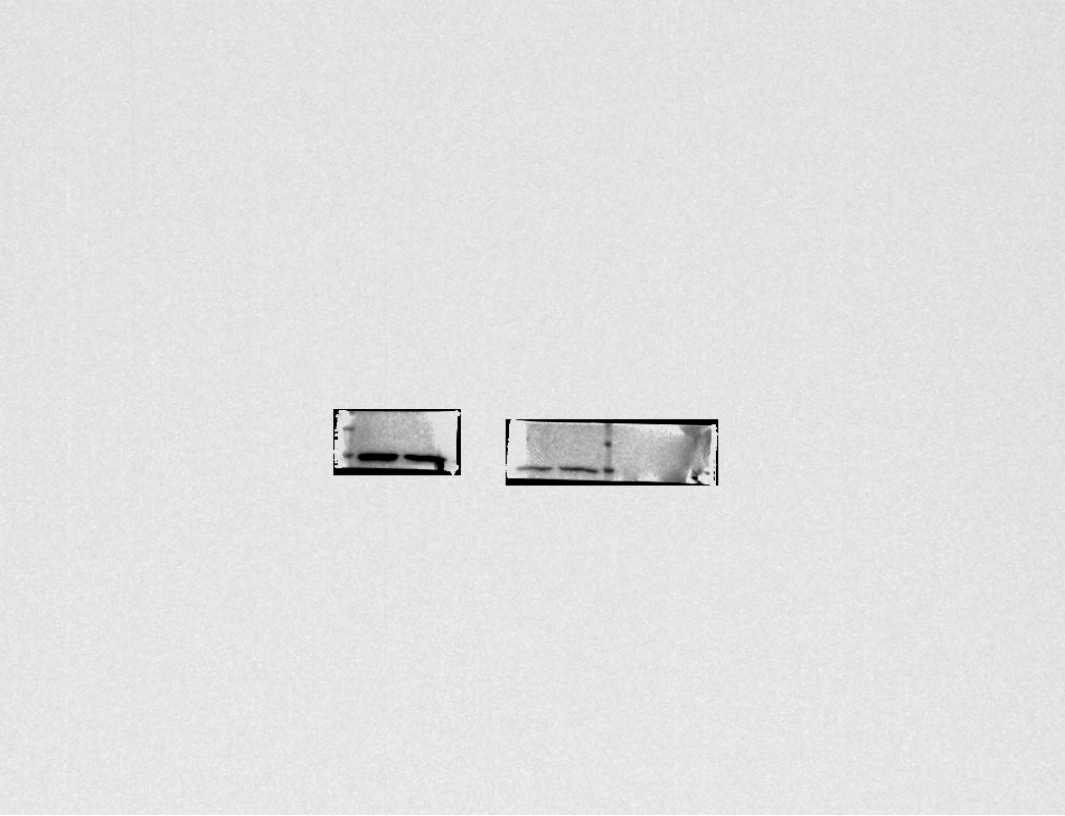

Supplement: Figure 3—source data 2. [file elife-102277-fig3-data2.zip › Figure 3-source data 2/Figure 3A-source data 2/Actin.tif]

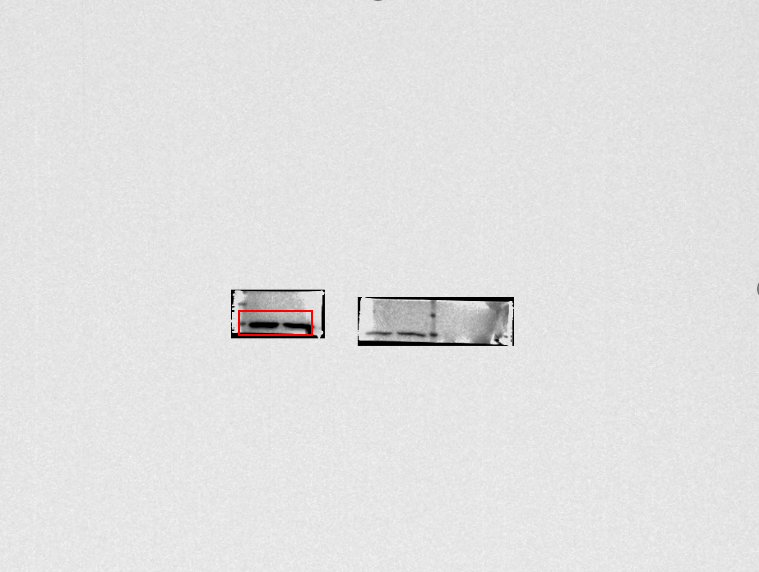

Supplement: Figure 3—source data 2. [file elife-102277-fig3-data2.zip › Figure 3-source data 2/Figure 3A-source data 2/Actin_2.tif]

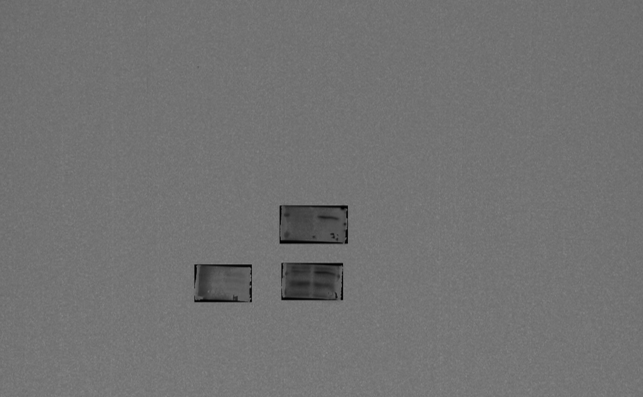

Supplement: Figure 3—source data 2. [file elife-102277-fig3-data2.zip › Figure 3-source data 2/Figure 3A-source data 2/nsp16-input.tif]

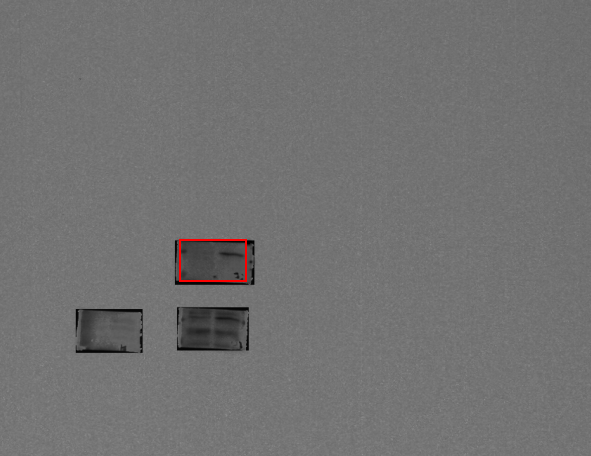

Supplement: Figure 3—source data 2. [file elife-102277-fig3-data2.zip › Figure 3-source data 2/Figure 3A-source data 2/nsp16-input_2.tif]

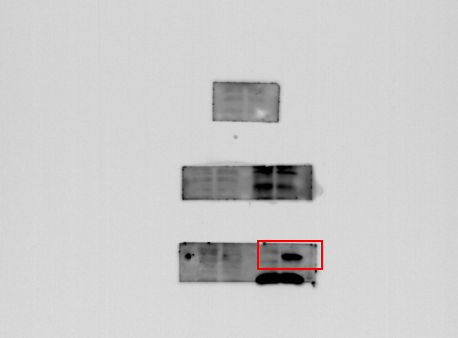

Supplement: Figure 3—source data 2. [file elife-102277-fig3-data2.zip › Figure 3-source data 2/Figure 3A-source data 2/nsp16-IP-2.tif]

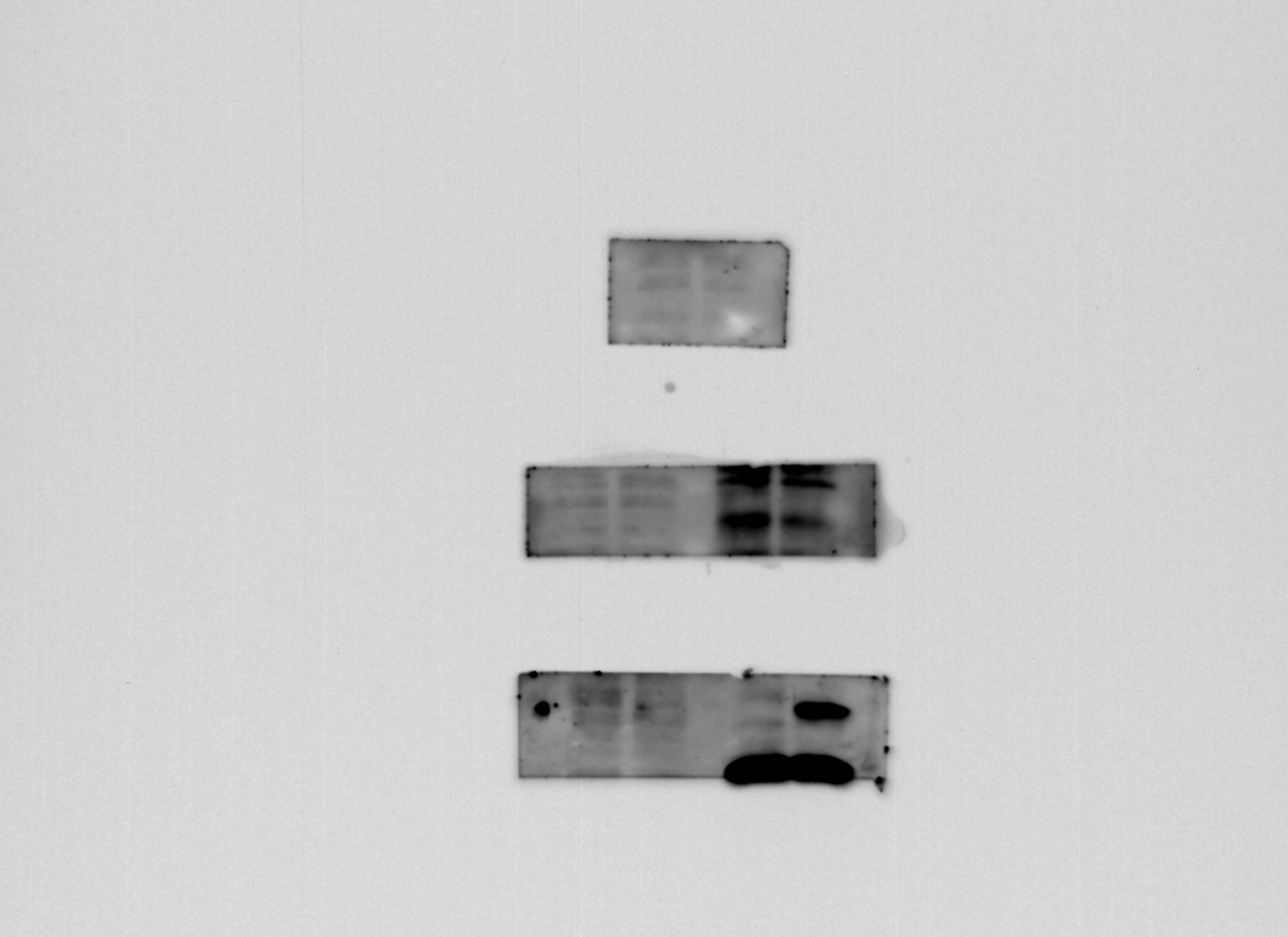

Supplement: Figure 3—source data 2. [file elife-102277-fig3-data2.zip › Figure 3-source data 2/Figure 3A-source data 2/nsp16-IP.tif]

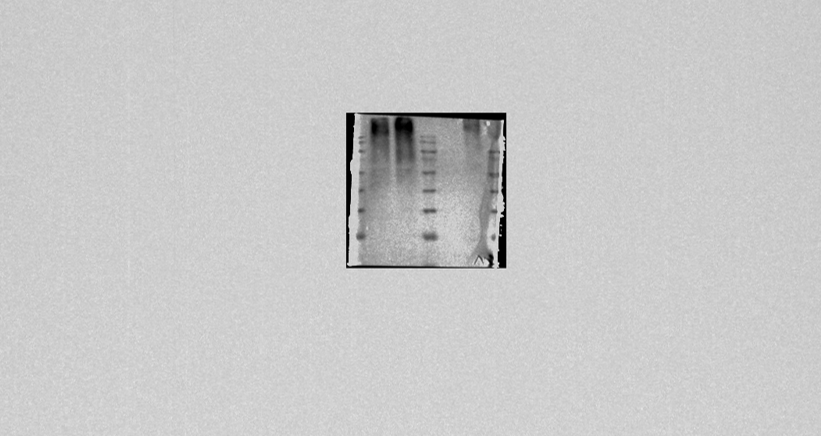

Supplement: Figure 3—source data 2. [file elife-102277-fig3-data2.zip › Figure 3-source data 2/Figure 3A-source data 2/Ub-Myc.tif]

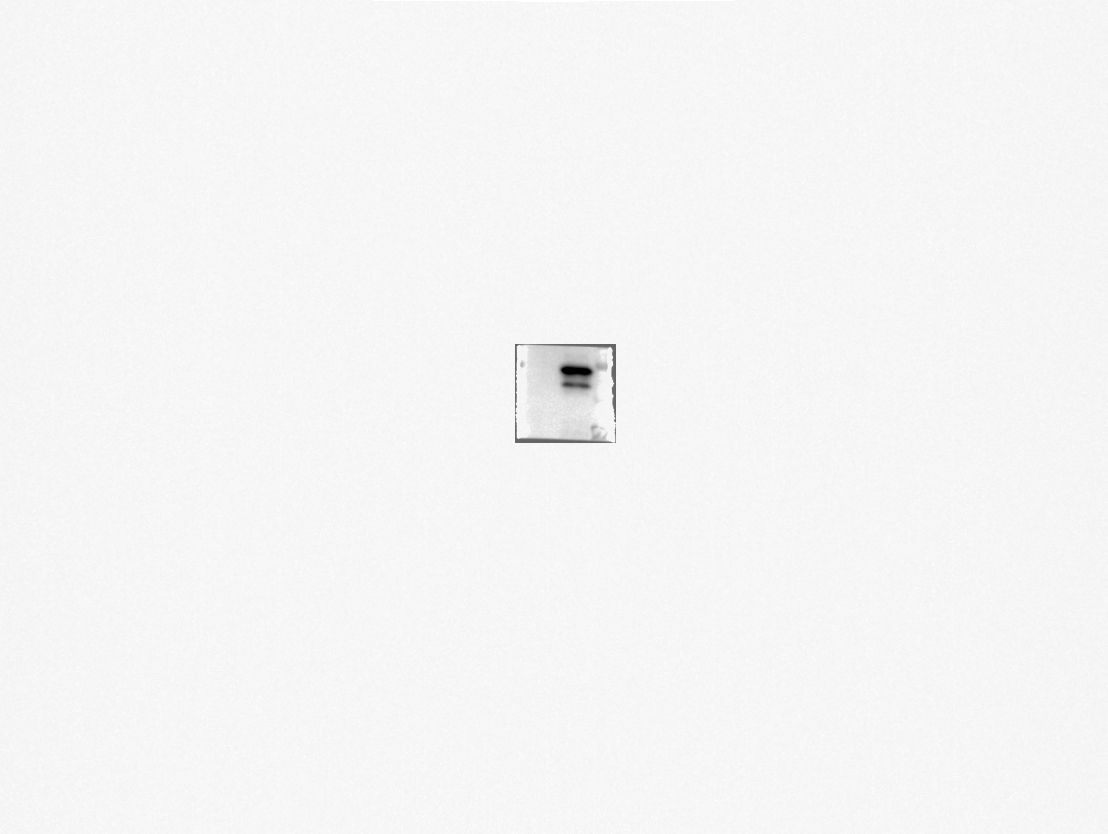

Supplement: Figure 3—source data 2. [file elife-102277-fig3-data2.zip › Figure 3-source data 2/Figure 3B-source data 2/nsp16-input.tif]

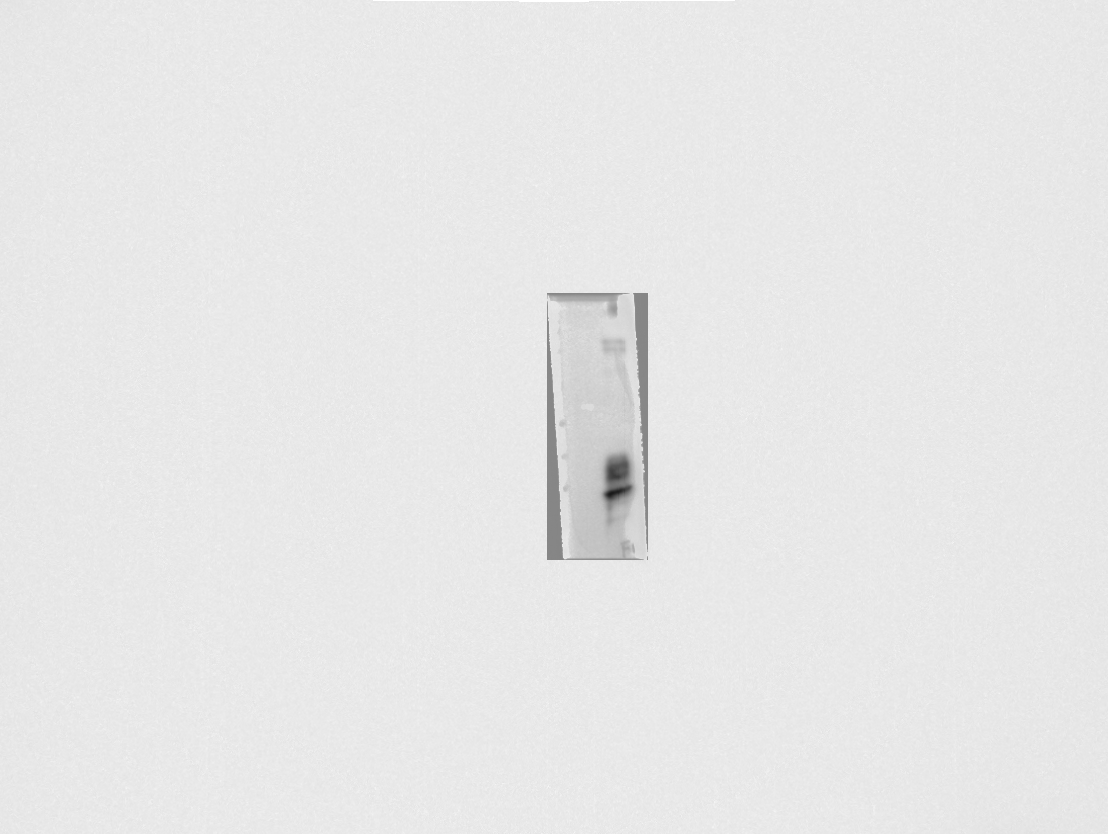

Supplement: Figure 3—source data 2. [file elife-102277-fig3-data2.zip › Figure 3-source data 2/Figure 3B-source data 2/nsp16-IP.tif]

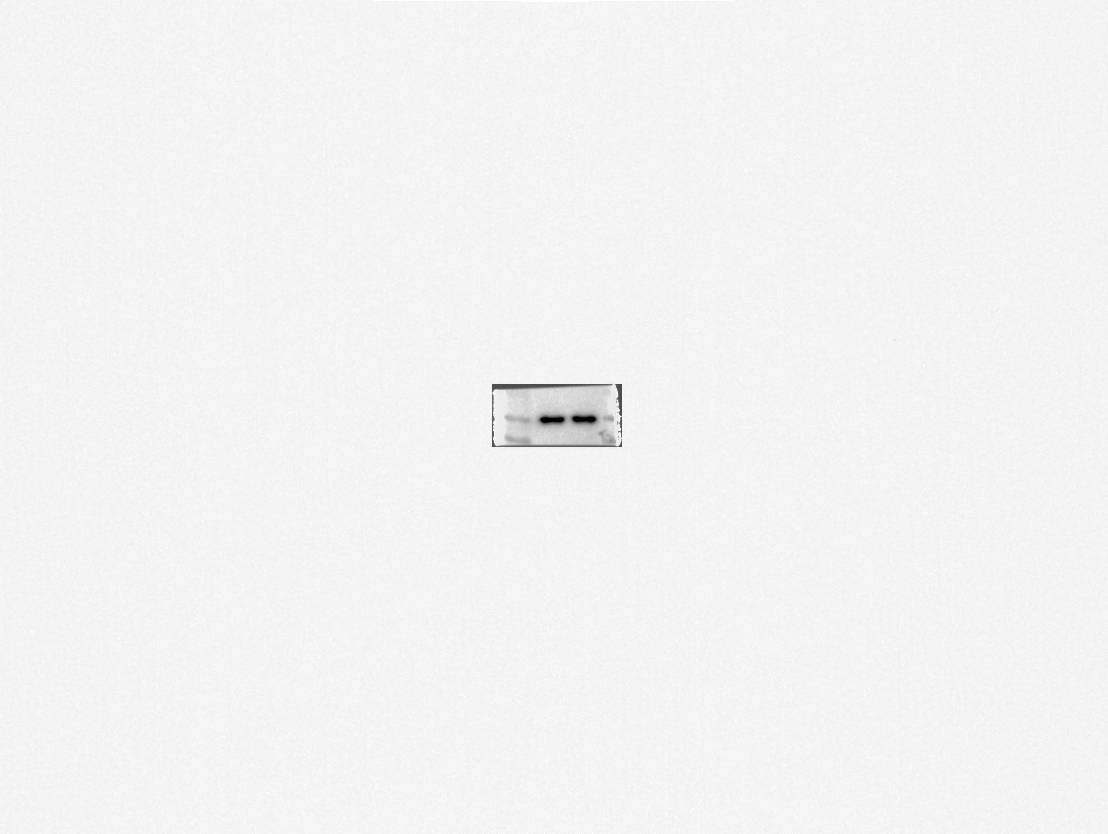

Supplement: Figure 3—source data 2. [file elife-102277-fig3-data2.zip › Figure 3-source data 2/Figure 3B-source data 2/Tubulin.tif]

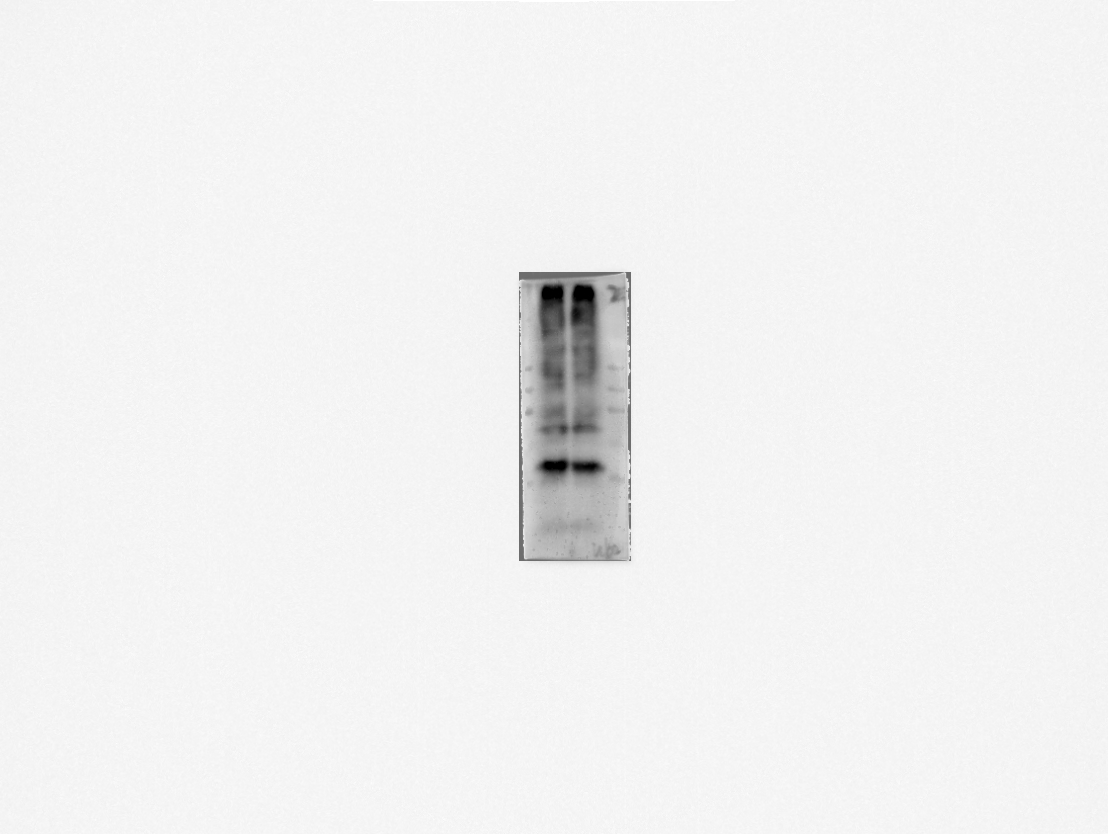

Supplement: Figure 3—source data 2. [file elife-102277-fig3-data2.zip › Figure 3-source data 2/Figure 3B-source data 2/Ub-input.tif]

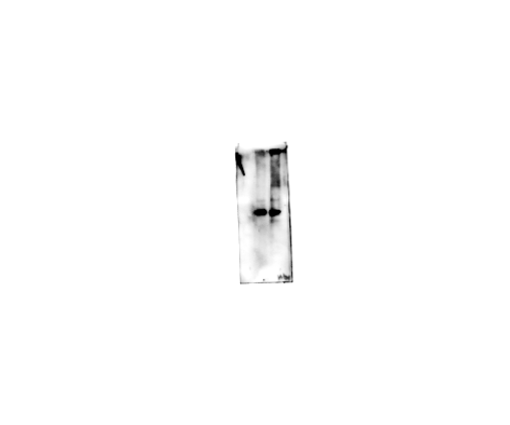

Supplement: Figure 3—source data 2. [file elife-102277-fig3-data2.zip › Figure 3-source data 2/Figure 3B-source data 2/Ub-IP.tif]

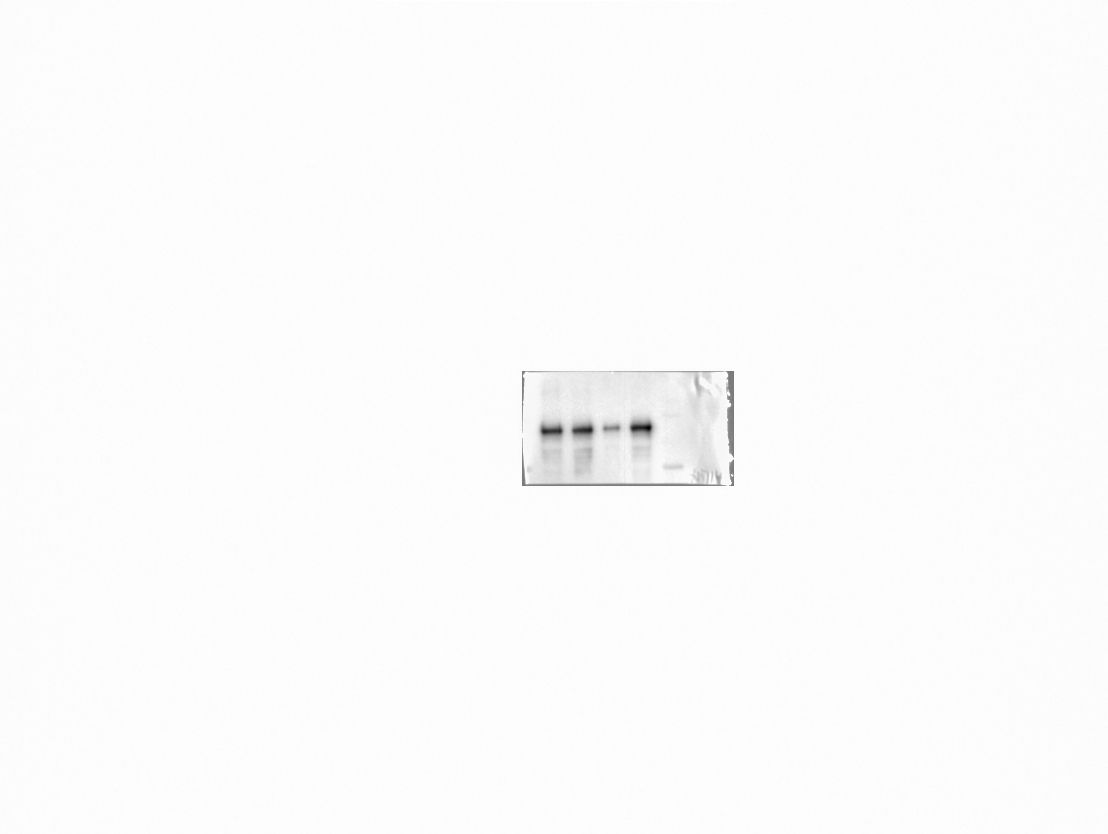

Supplement: Figure 3—source data 2. [file elife-102277-fig3-data2.zip › Figure 3-source data 2/Figure 3C-source data 2/MARCHF7.tif]

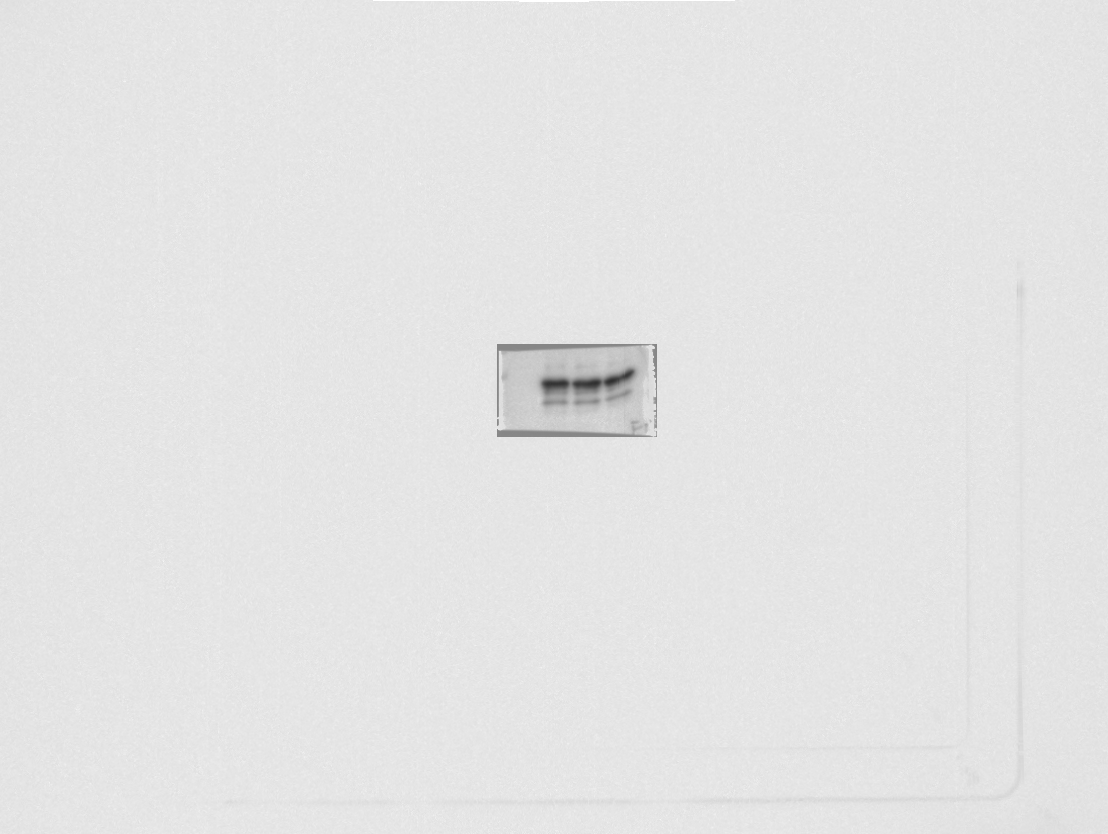

Supplement: Figure 3—source data 2. [file elife-102277-fig3-data2.zip › Figure 3-source data 2/Figure 3C-source data 2/nsp16-input.tif]

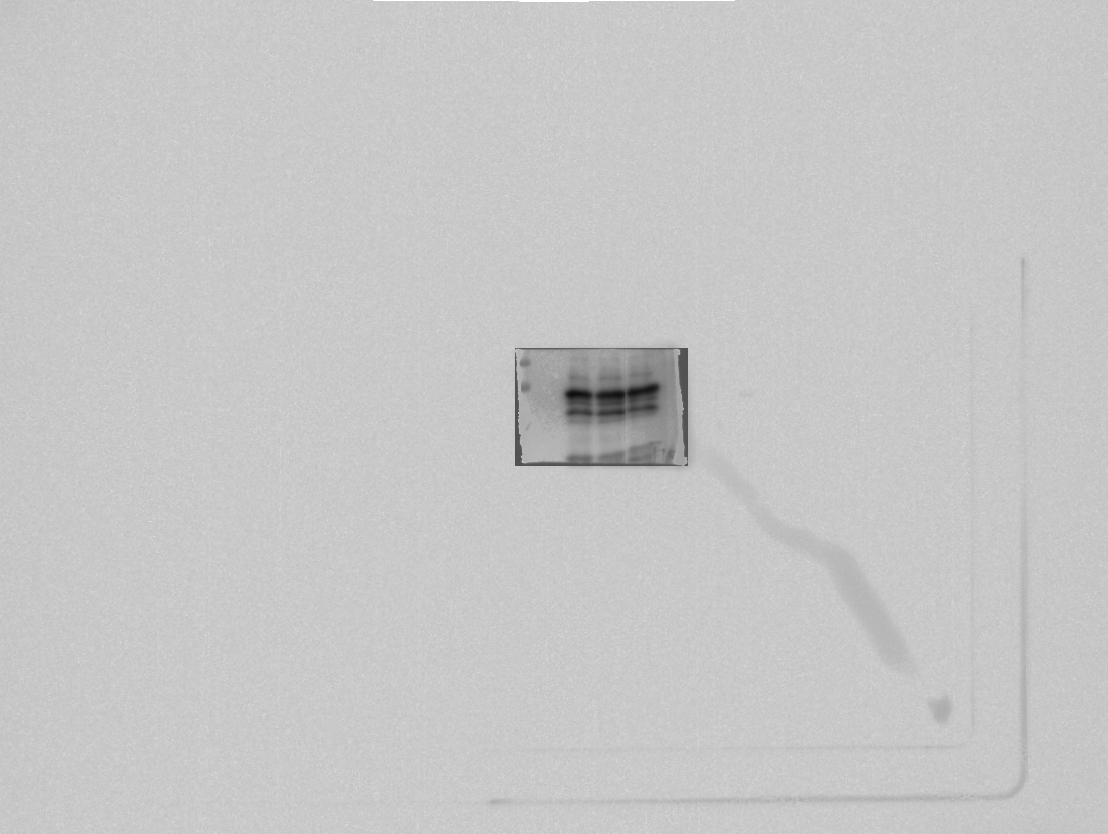

Supplement: Figure 3—source data 2. [file elife-102277-fig3-data2.zip › Figure 3-source data 2/Figure 3C-source data 2/nsp16-IP.tif]

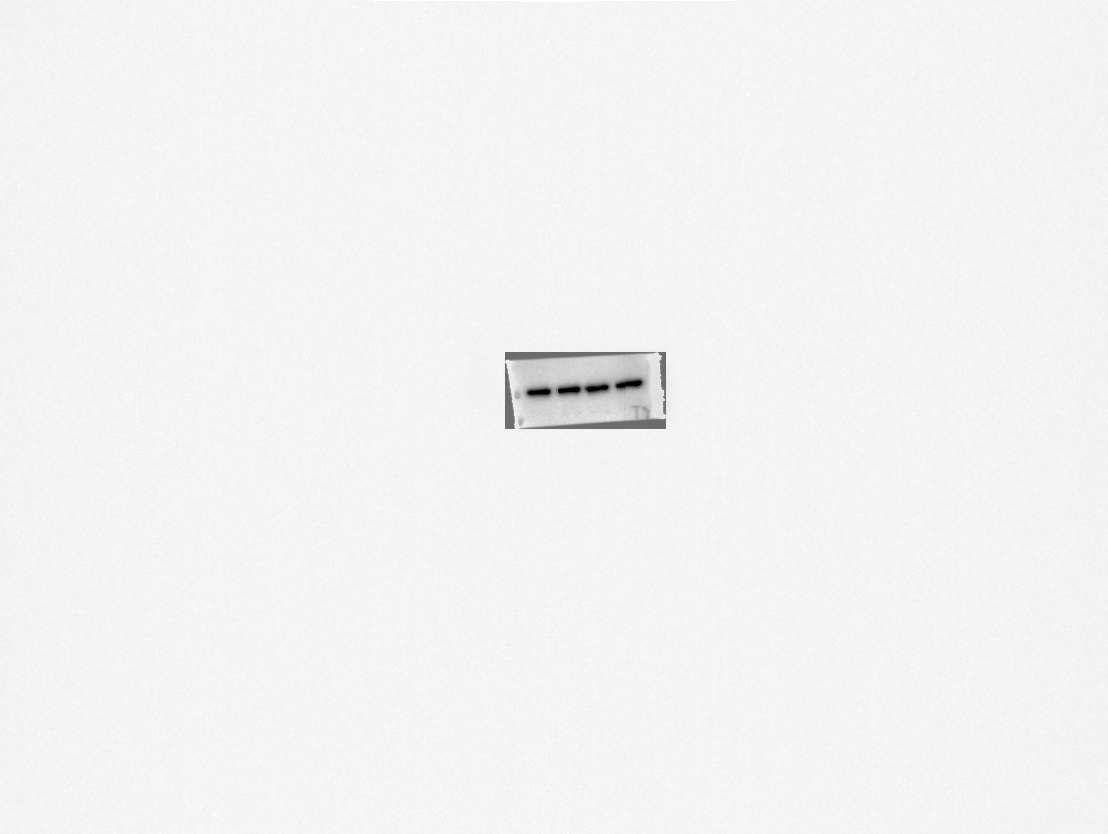

Supplement: Figure 3—source data 2. [file elife-102277-fig3-data2.zip › Figure 3-source data 2/Figure 3C-source data 2/Tubulin.tif]

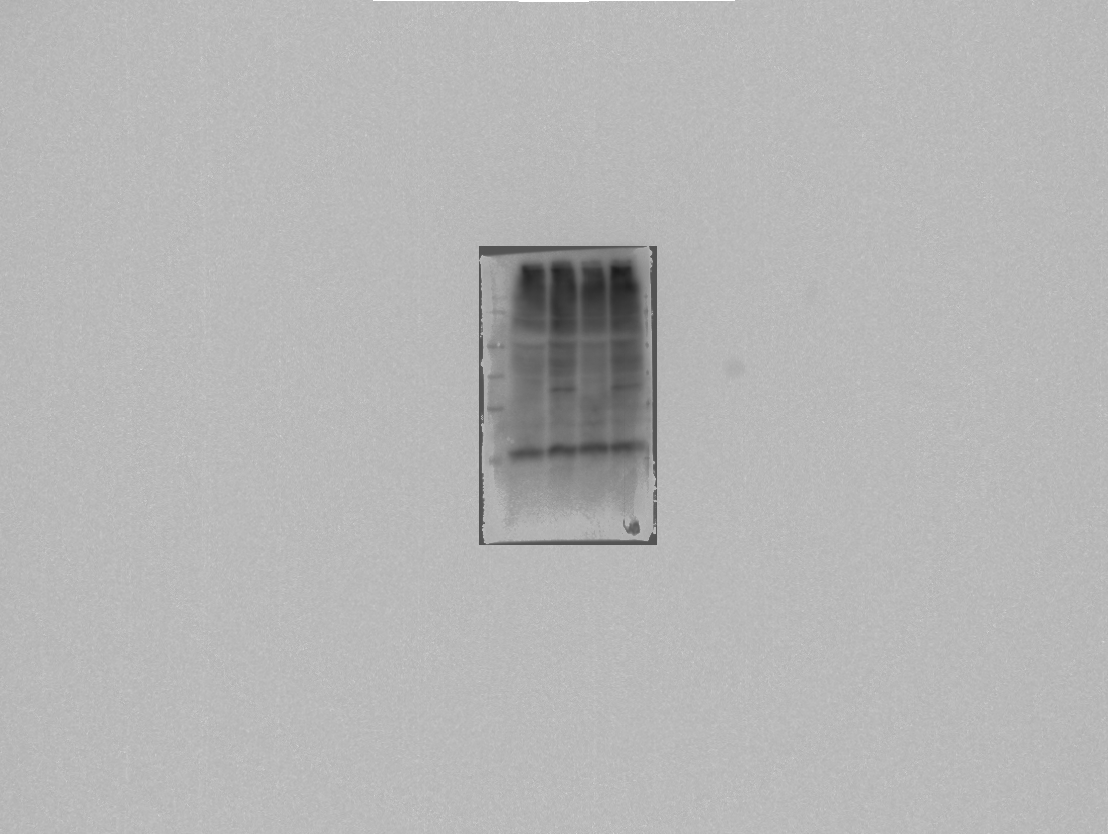

Supplement: Figure 3—source data 2. [file elife-102277-fig3-data2.zip › Figure 3-source data 2/Figure 3C-source data 2/Ub-Myc-input.tif]

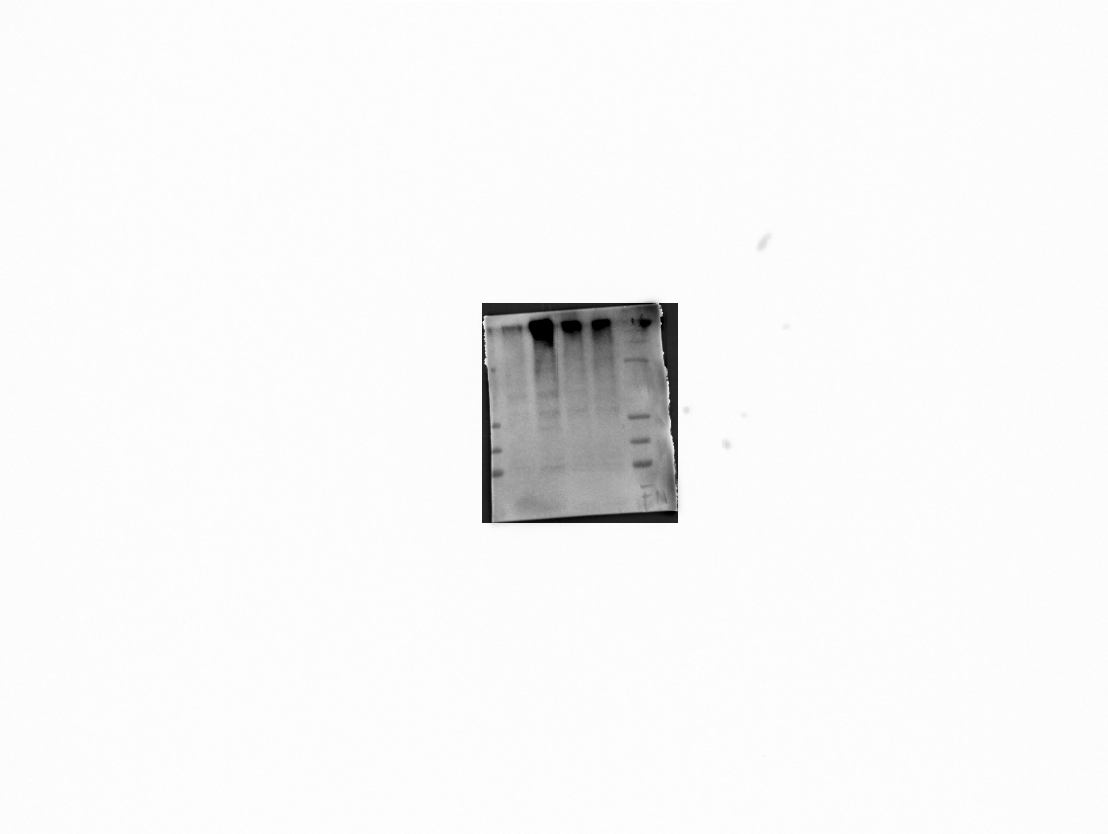

Supplement: Figure 3—source data 2. [file elife-102277-fig3-data2.zip › Figure 3-source data 2/Figure 3C-source data 2/Ub-Myc-IP.tif]

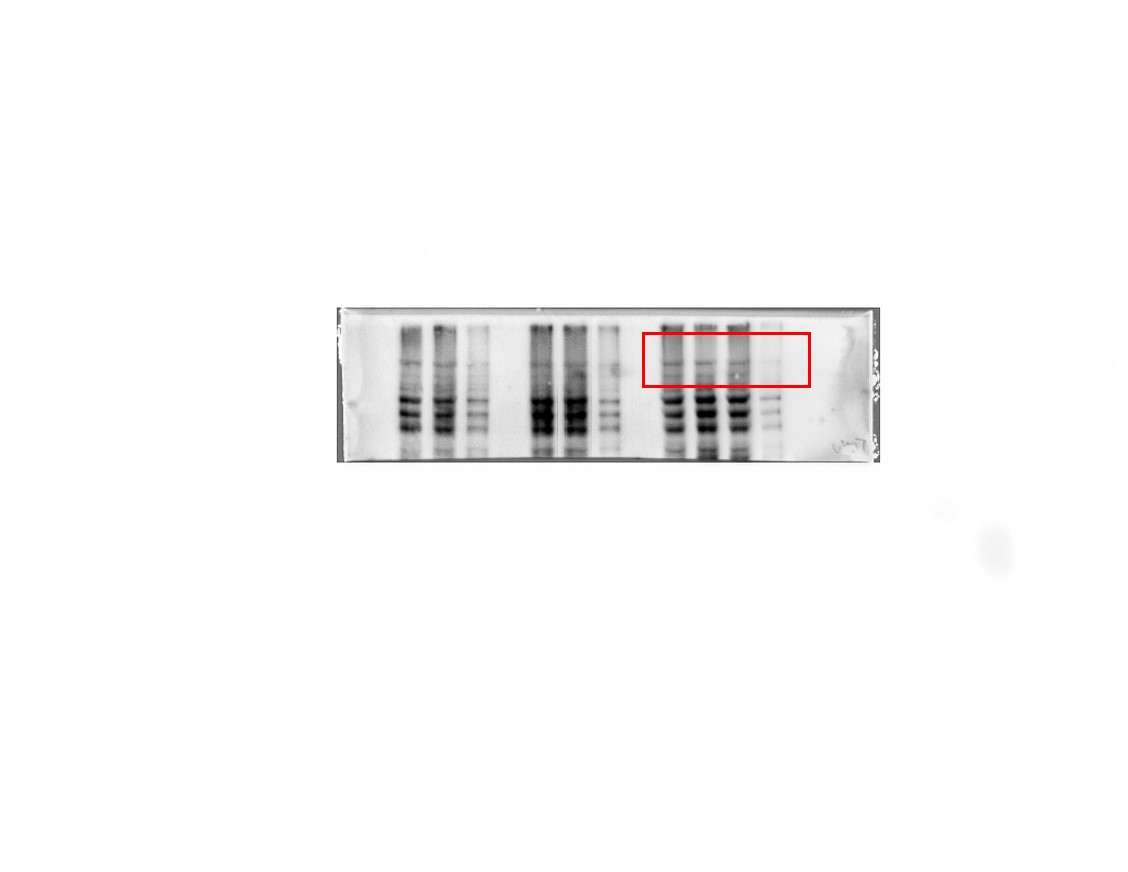

Supplement: Figure 3—source data 2. [file elife-102277-fig3-data2.zip › Figure 3-source data 2/Figure 3C-source data 2/UBR5-1.tif]

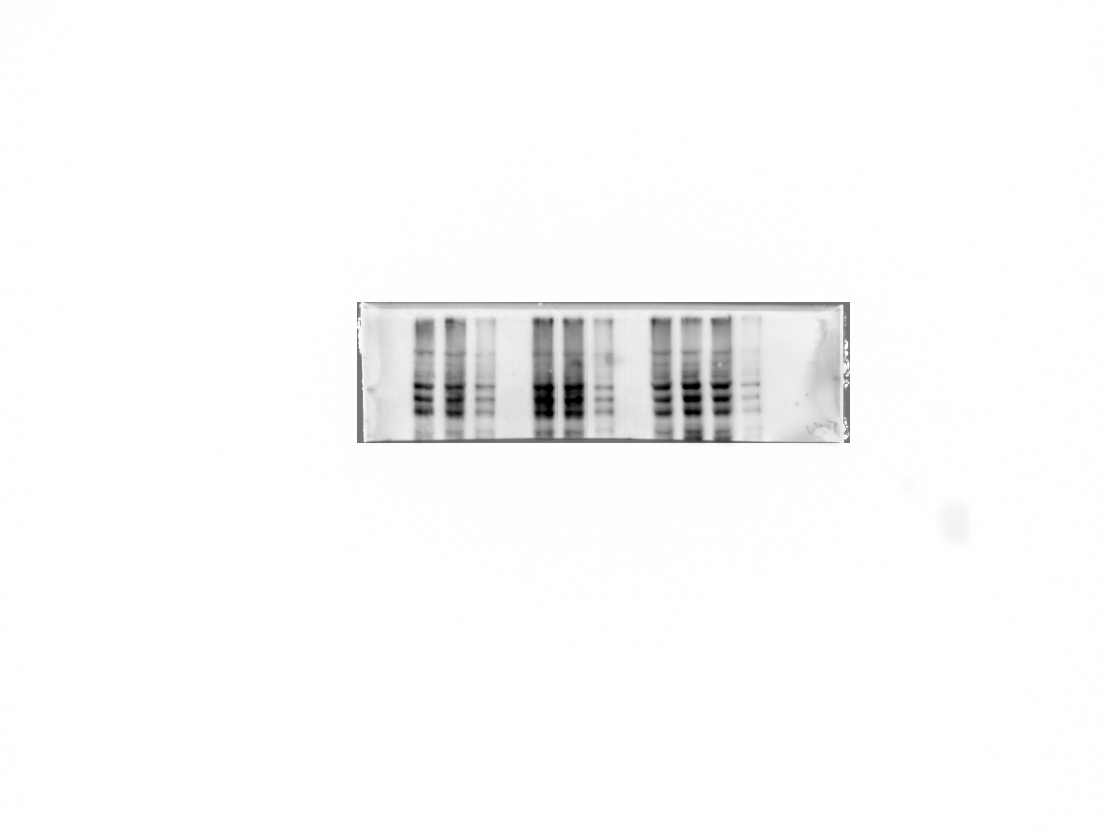

Supplement: Figure 3—source data 2. [file elife-102277-fig3-data2.zip › Figure 3-source data 2/Figure 3C-source data 2/UBR5.tif]

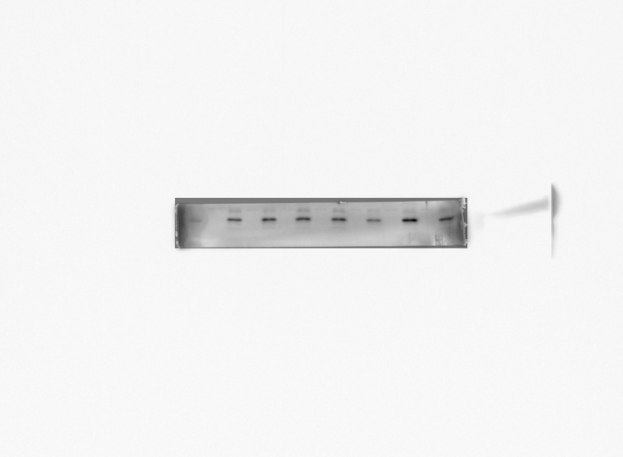

Supplement: Figure 3—source data 2. [file elife-102277-fig3-data2.zip › Figure 3-source data 2/Figure 3D-source data 2/nsp16-input.tif]

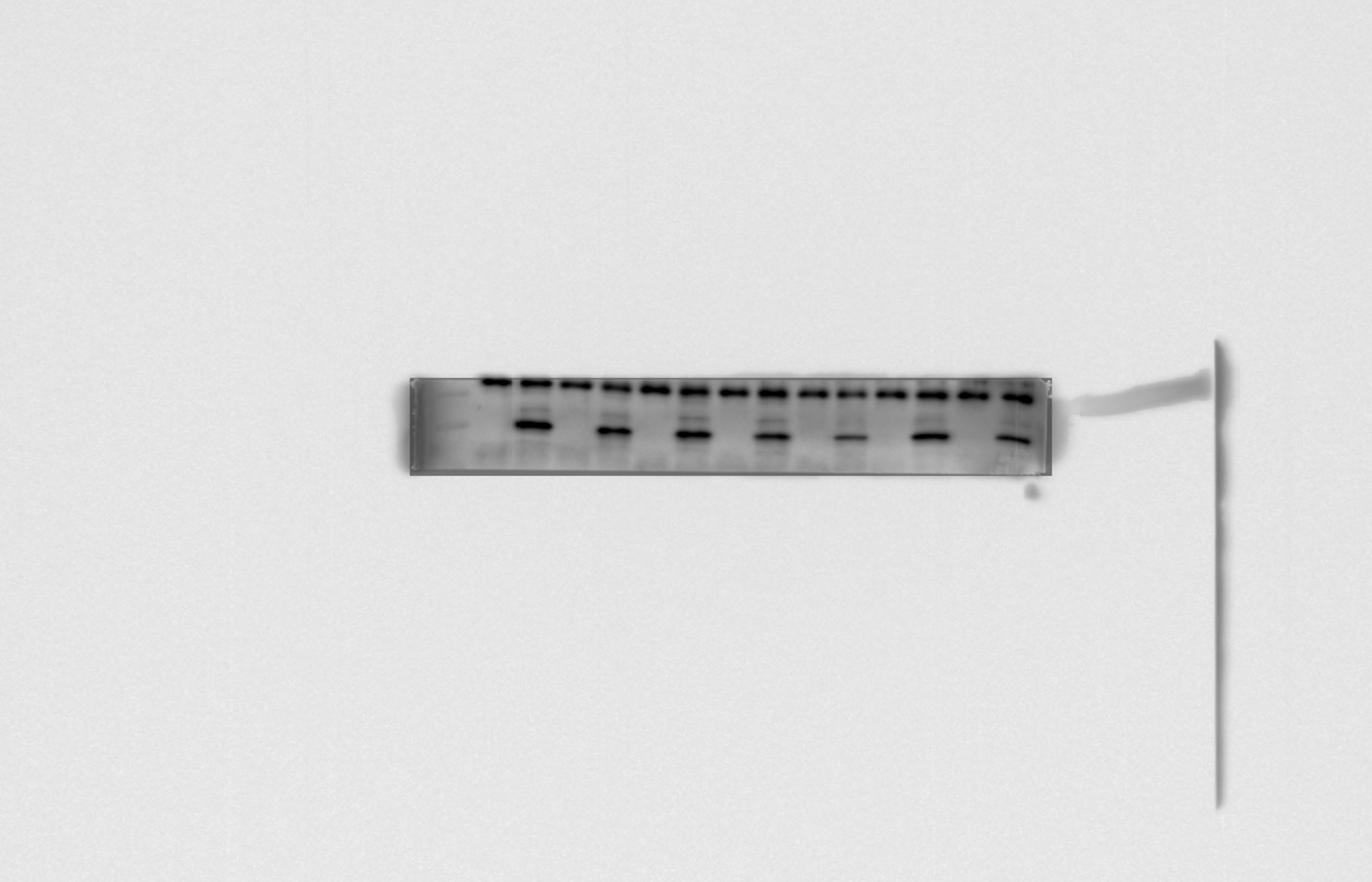

Supplement: Figure 3—source data 2. [file elife-102277-fig3-data2.zip › Figure 3-source data 2/Figure 3D-source data 2/nsp16-IP.tif]

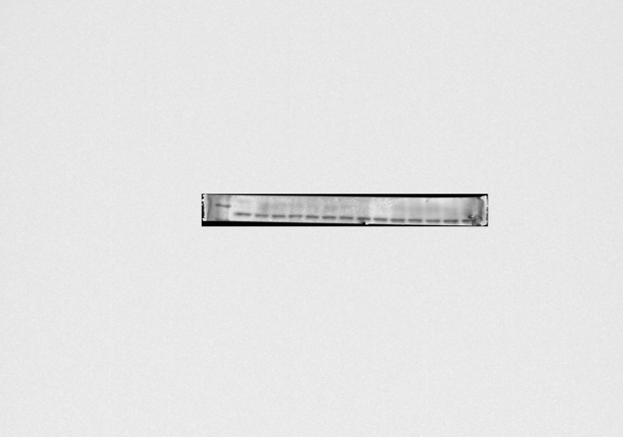

Supplement: Figure 3—source data 2. [file elife-102277-fig3-data2.zip › Figure 3-source data 2/Figure 3D-source data 2/Tubulin-input.tif]

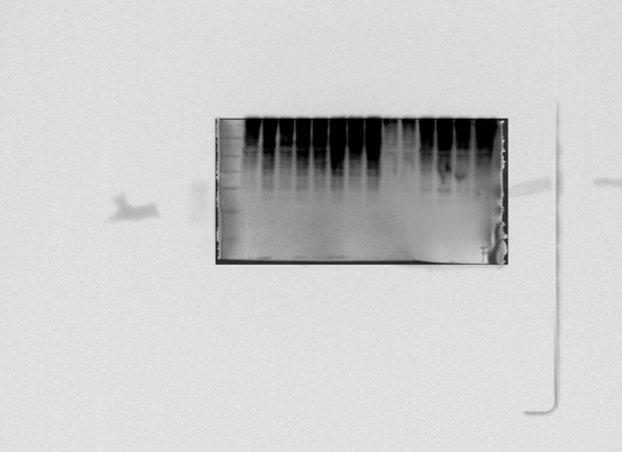

Supplement: Figure 3—source data 2. [file elife-102277-fig3-data2.zip › Figure 3-source data 2/Figure 3D-source data 2/Ub-input.tif]

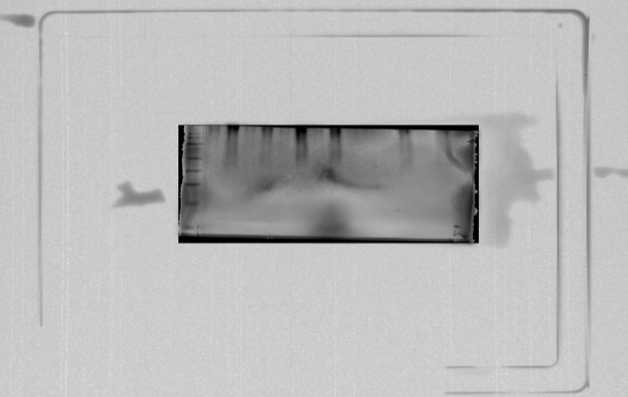

Supplement: Figure 3—source data 2. [file elife-102277-fig3-data2.zip › Figure 3-source data 2/Figure 3D-source data 2/Ub-IP.tif]

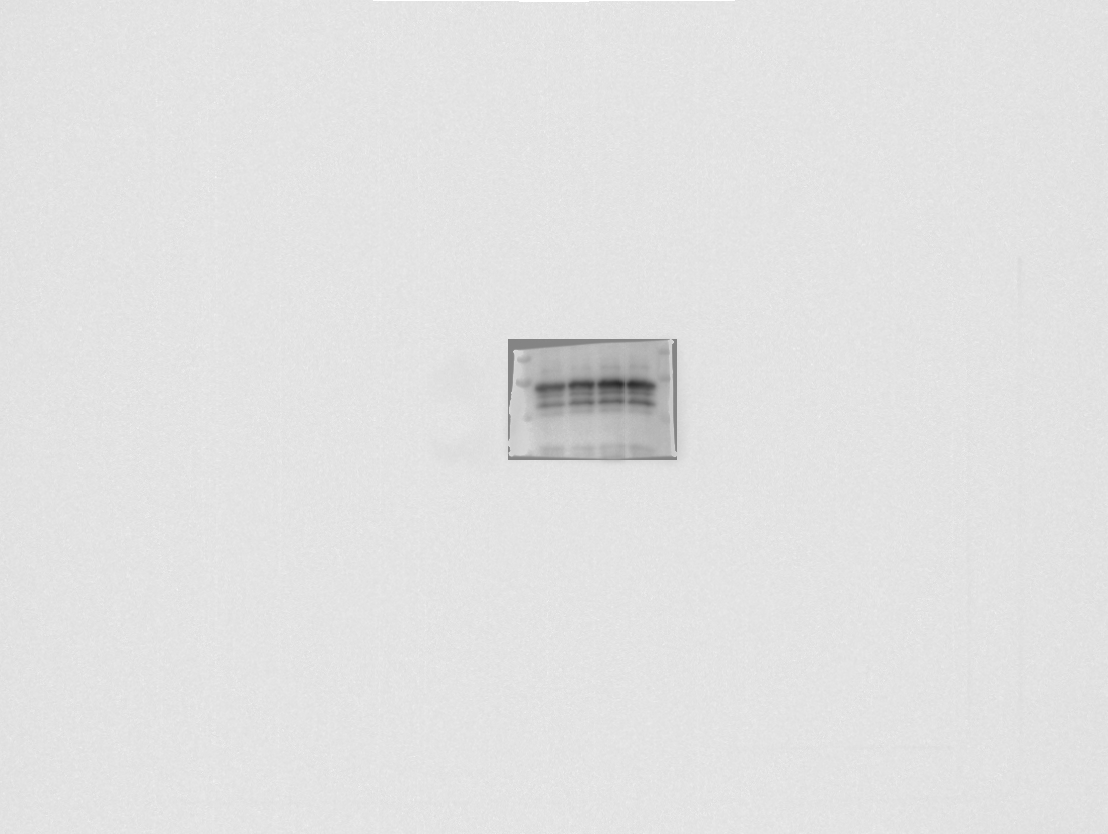

Supplement: Figure 3—source data 2. [file elife-102277-fig3-data2.zip › Figure 3-source data 2/Figure 3E-source data 2/nsp16-input-2.tif]

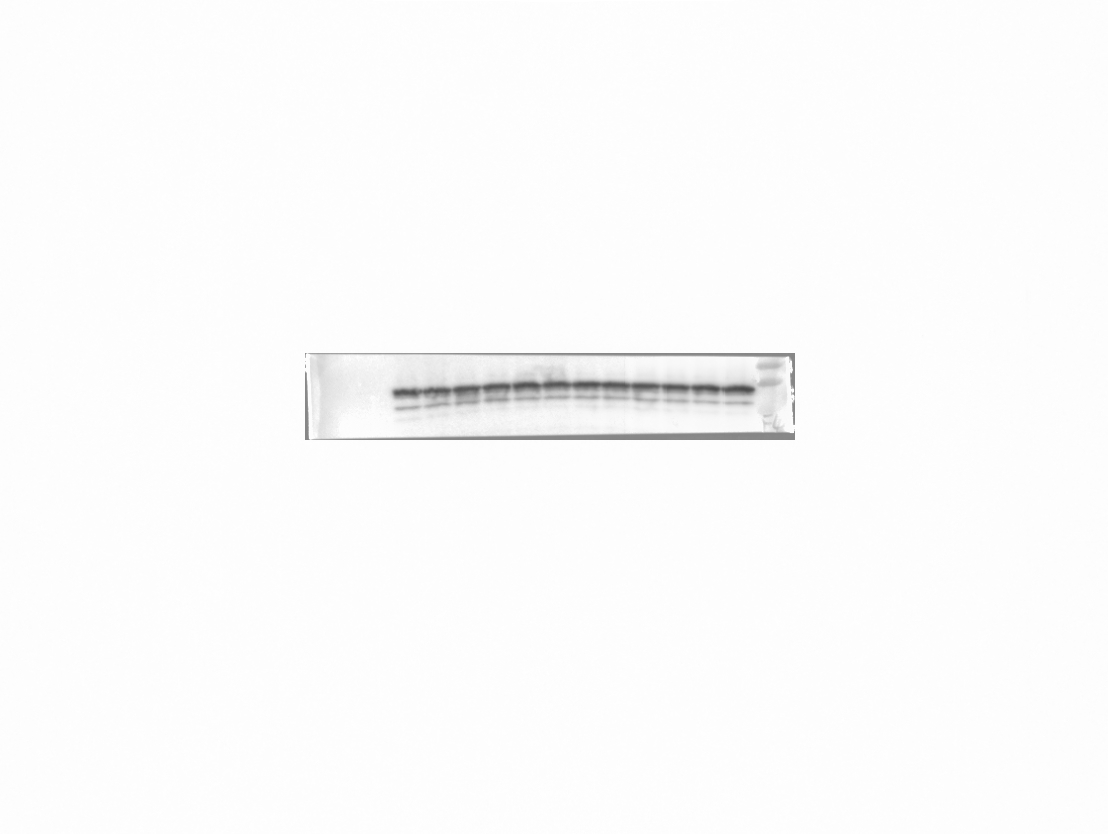

Supplement: Figure 3—source data 2. [file elife-102277-fig3-data2.zip › Figure 3-source data 2/Figure 3E-source data 2/nsp16-input.tif]

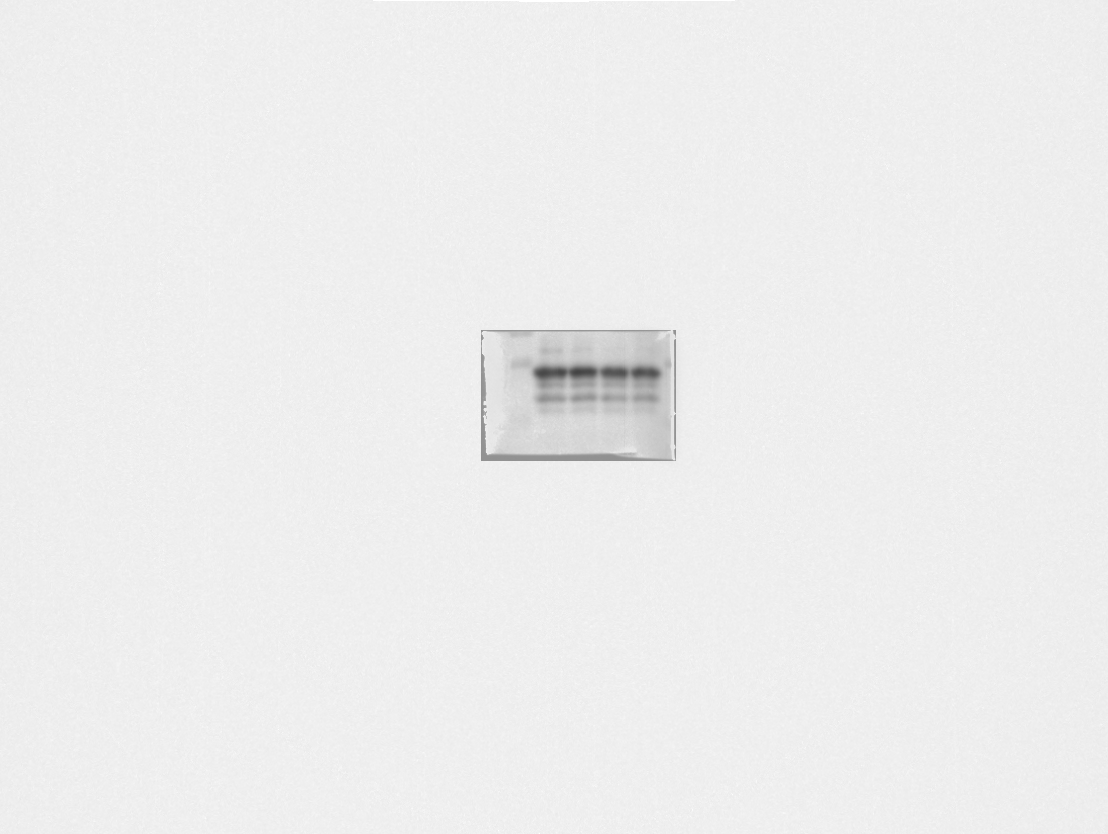

Supplement: Figure 3—source data 2. [file elife-102277-fig3-data2.zip › Figure 3-source data 2/Figure 3E-source data 2/nsp16-IP-2.tif]

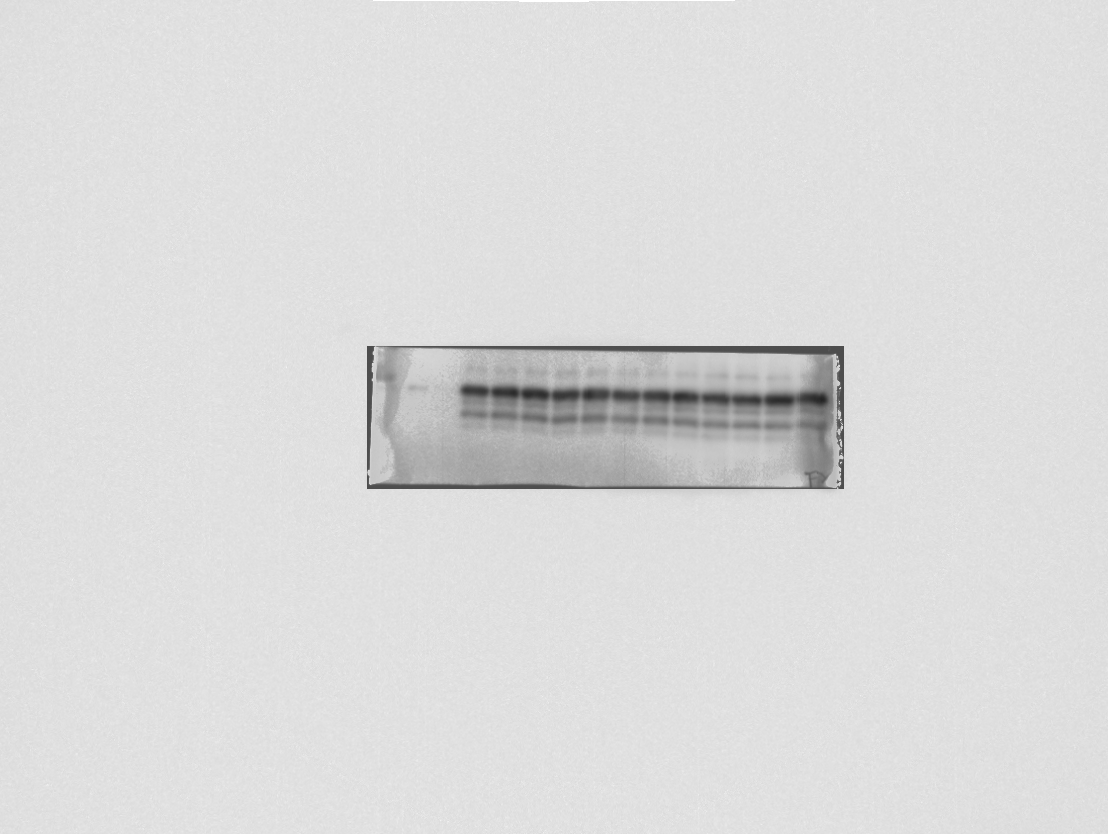

Supplement: Figure 3—source data 2. [file elife-102277-fig3-data2.zip › Figure 3-source data 2/Figure 3E-source data 2/nsp16-IP.tif]

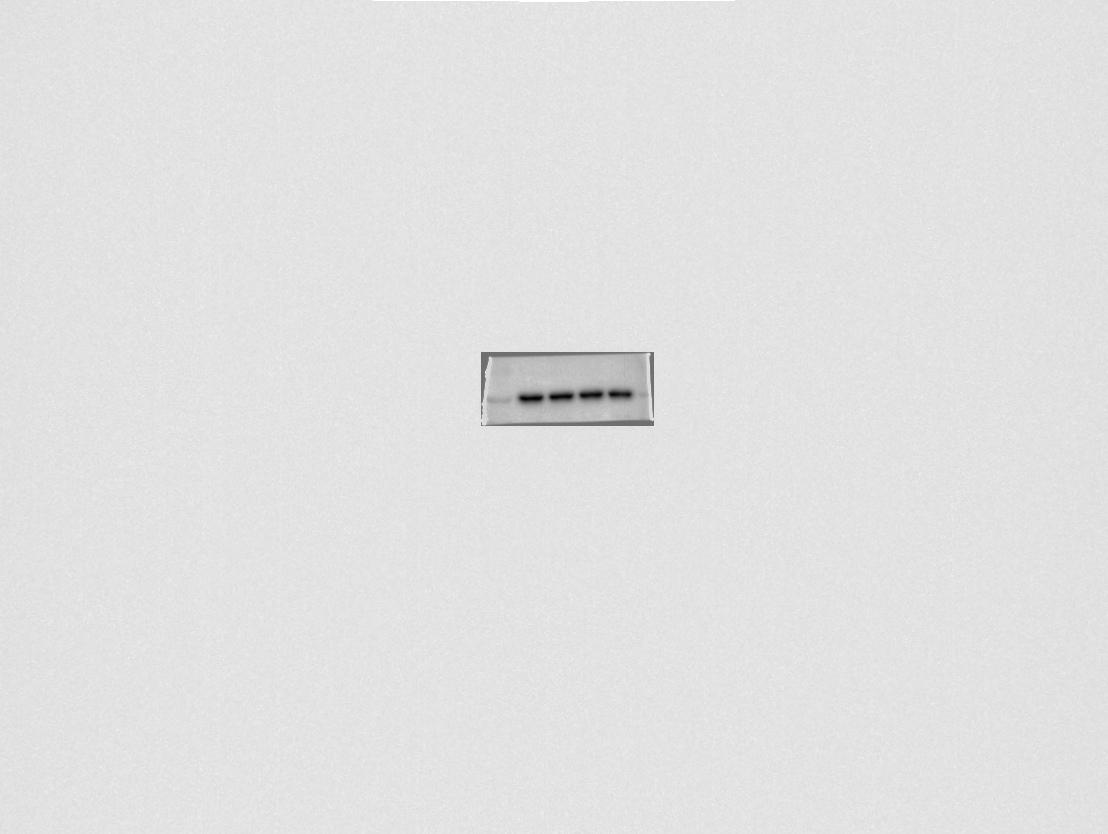

Supplement: Figure 3—source data 2. [file elife-102277-fig3-data2.zip › Figure 3-source data 2/Figure 3E-source data 2/Tubulin-input-2.tif]

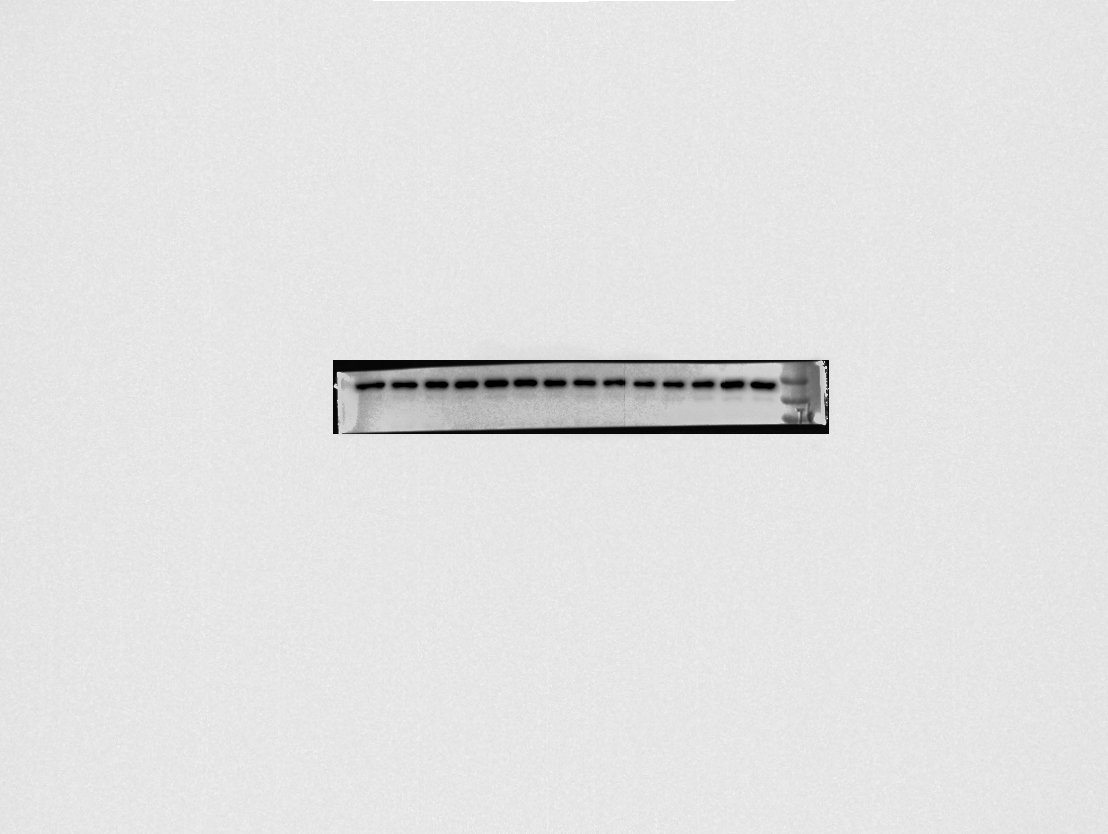

Supplement: Figure 3—source data 2. [file elife-102277-fig3-data2.zip › Figure 3-source data 2/Figure 3E-source data 2/Tubulin-input.tif]

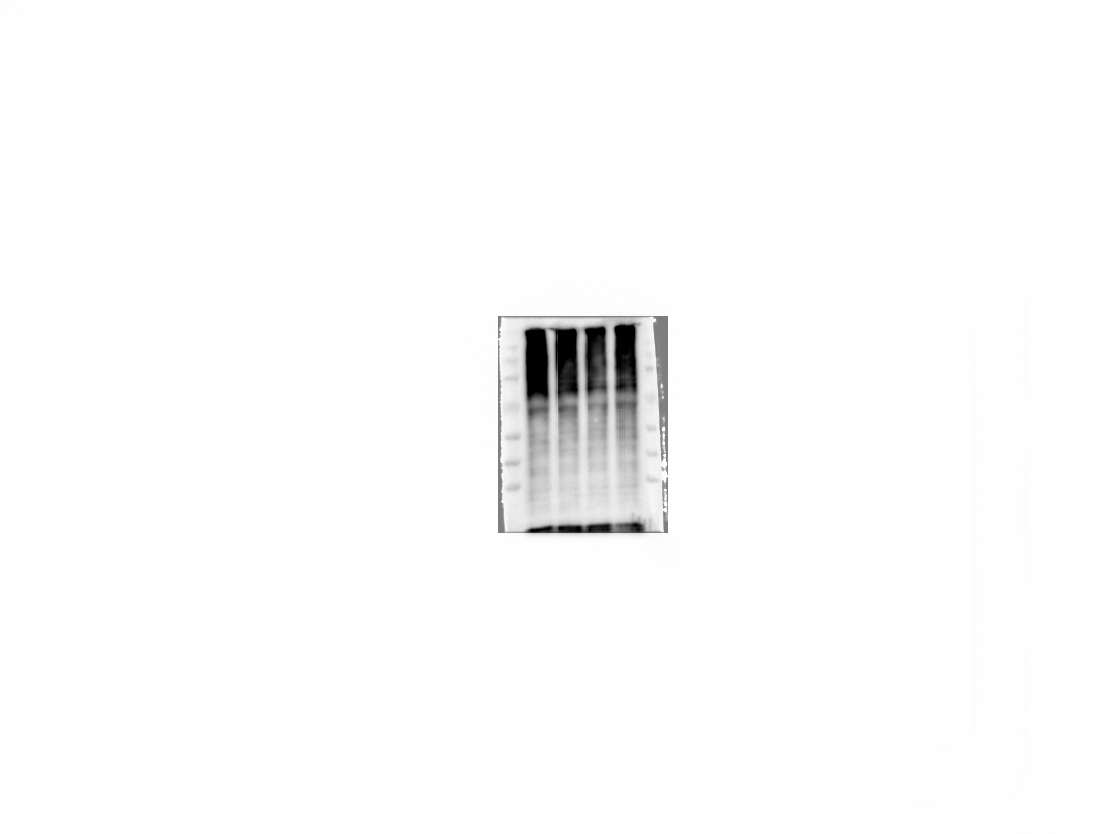

Supplement: Figure 3—source data 2. [file elife-102277-fig3-data2.zip › Figure 3-source data 2/Figure 3E-source data 2/Ub-input-2.tif]

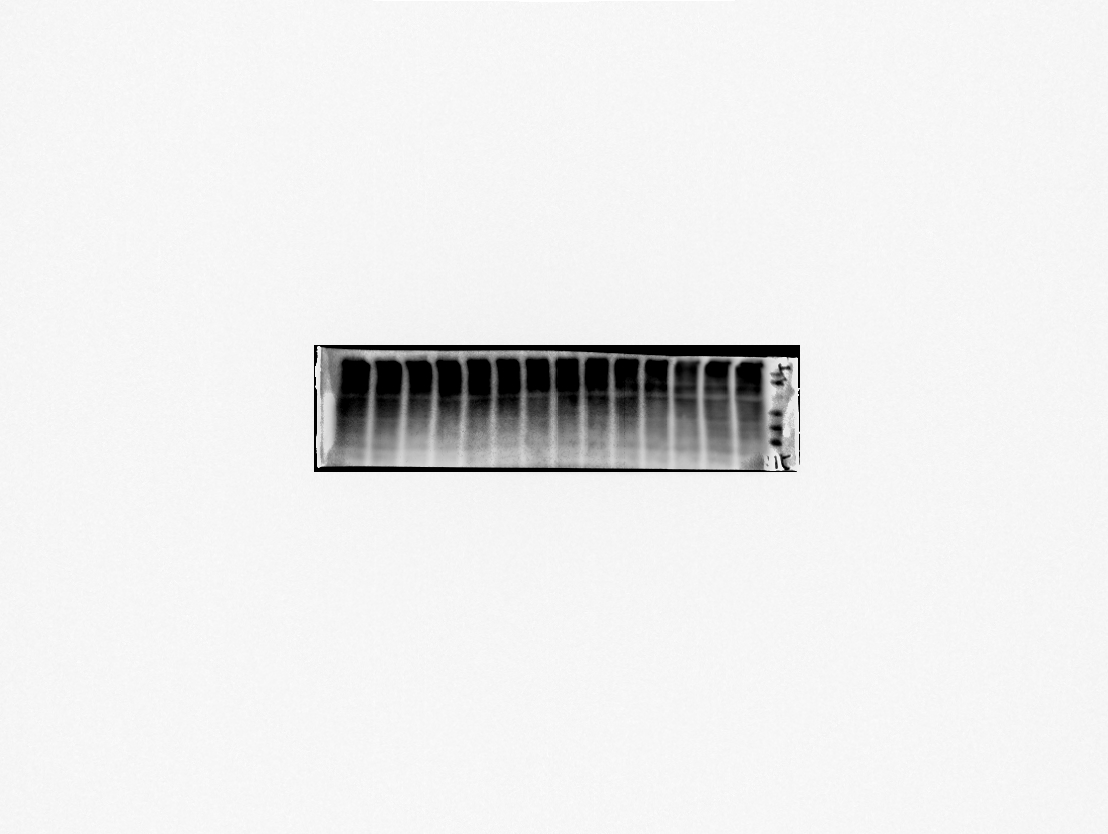

Supplement: Figure 3—source data 2. [file elife-102277-fig3-data2.zip › Figure 3-source data 2/Figure 3E-source data 2/Ub-input.tif]

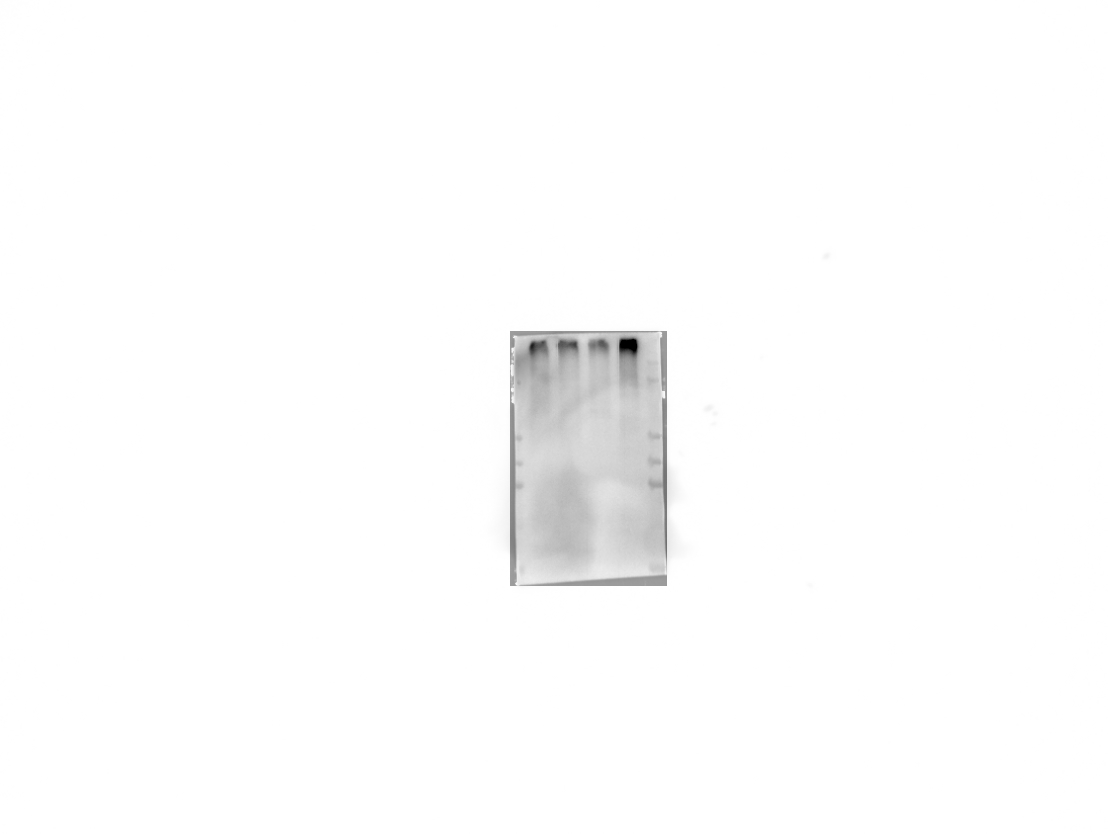

Supplement: Figure 3—source data 2. [file elife-102277-fig3-data2.zip › Figure 3-source data 2/Figure 3E-source data 2/Ub-IP-2.tif]

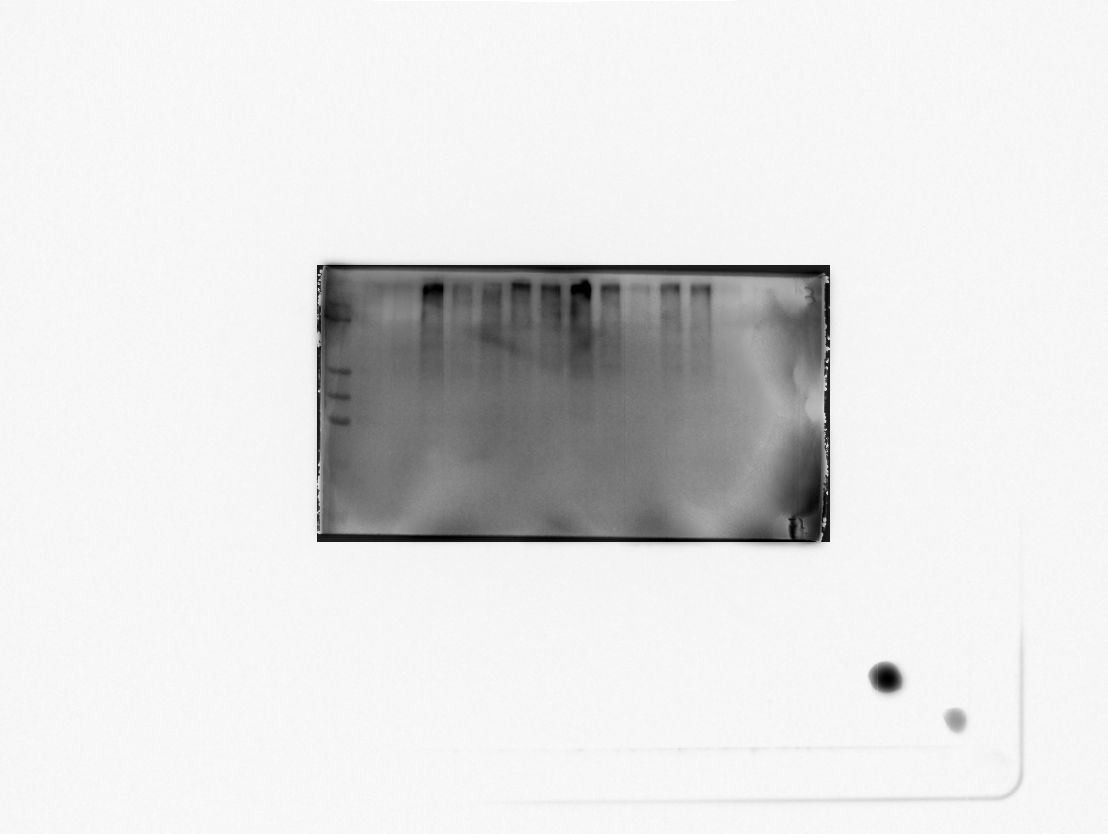

Supplement: Figure 3—source data 2. [file elife-102277-fig3-data2.zip › Figure 3-source data 2/Figure 3E-source data 2/Ub-IP.tif]

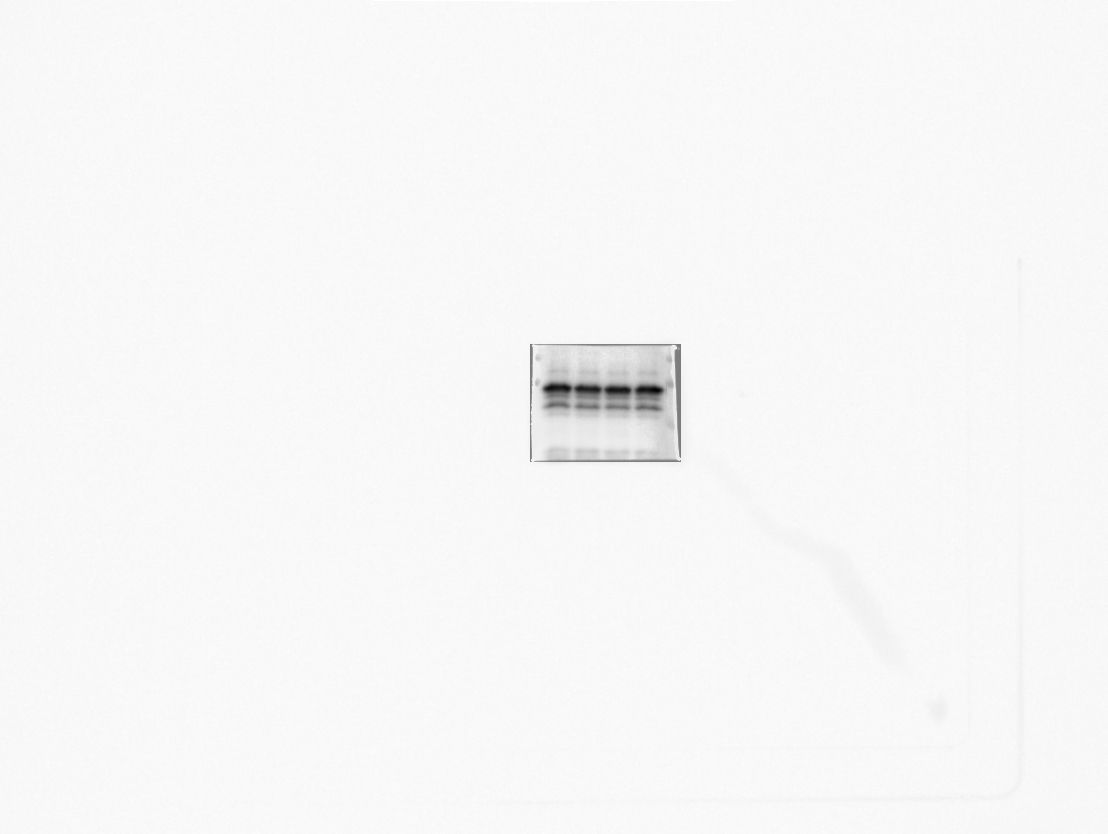

Supplement: Figure 3—source data 2. [file elife-102277-fig3-data2.zip › Figure 3-source data 2/Figure 3F-source data 2/nsp16-input-2.tif]

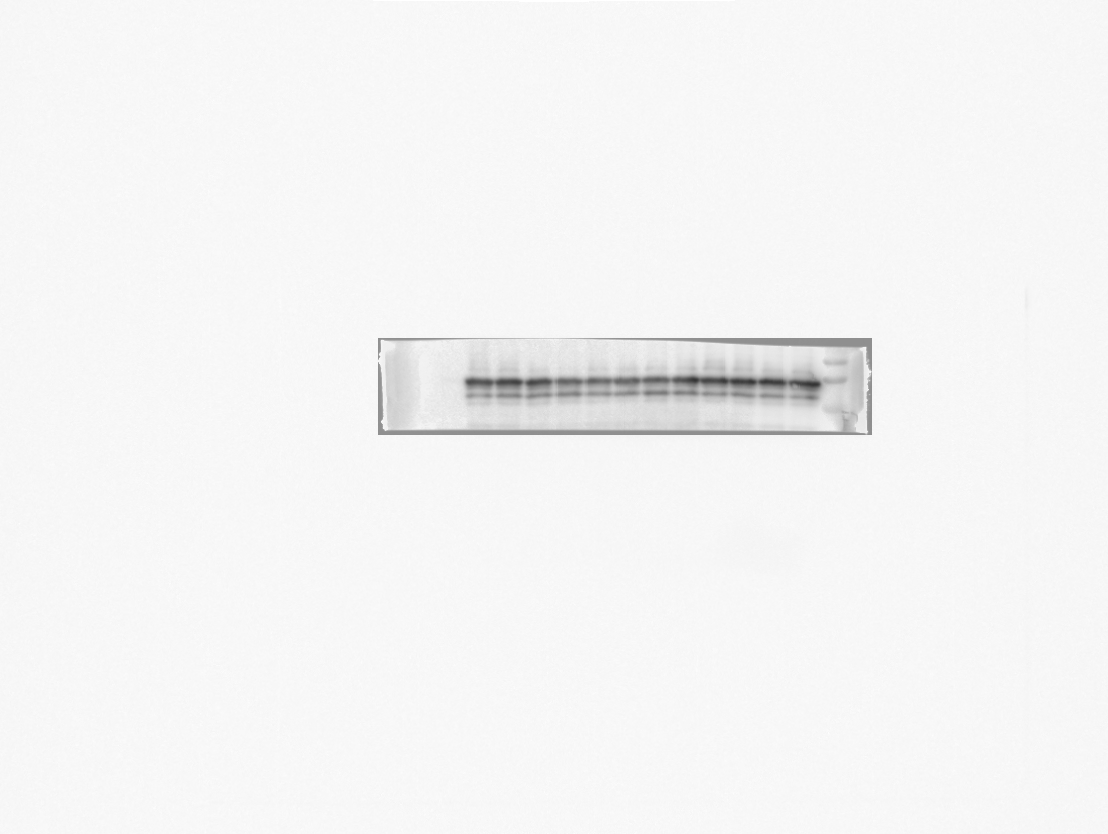

Supplement: Figure 3—source data 2. [file elife-102277-fig3-data2.zip › Figure 3-source data 2/Figure 3F-source data 2/nsp16-input.tif]

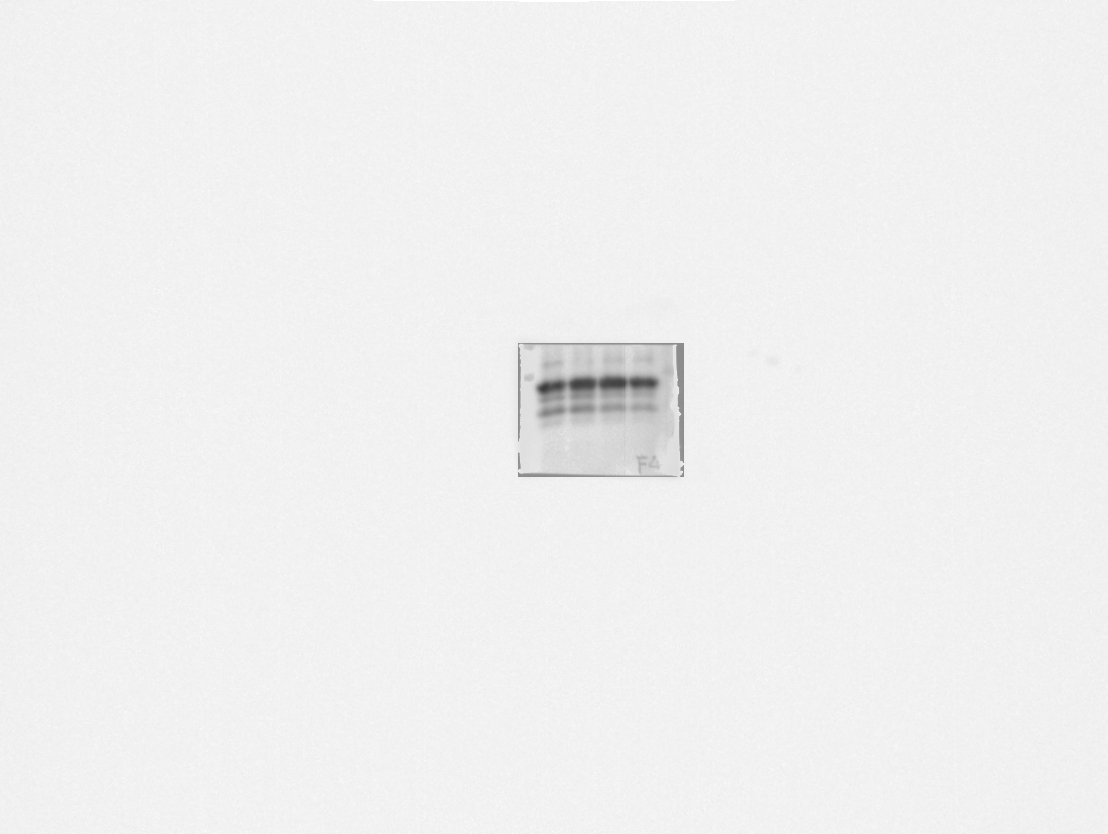

Supplement: Figure 3—source data 2. [file elife-102277-fig3-data2.zip › Figure 3-source data 2/Figure 3F-source data 2/nsp16-IP-2.tif]

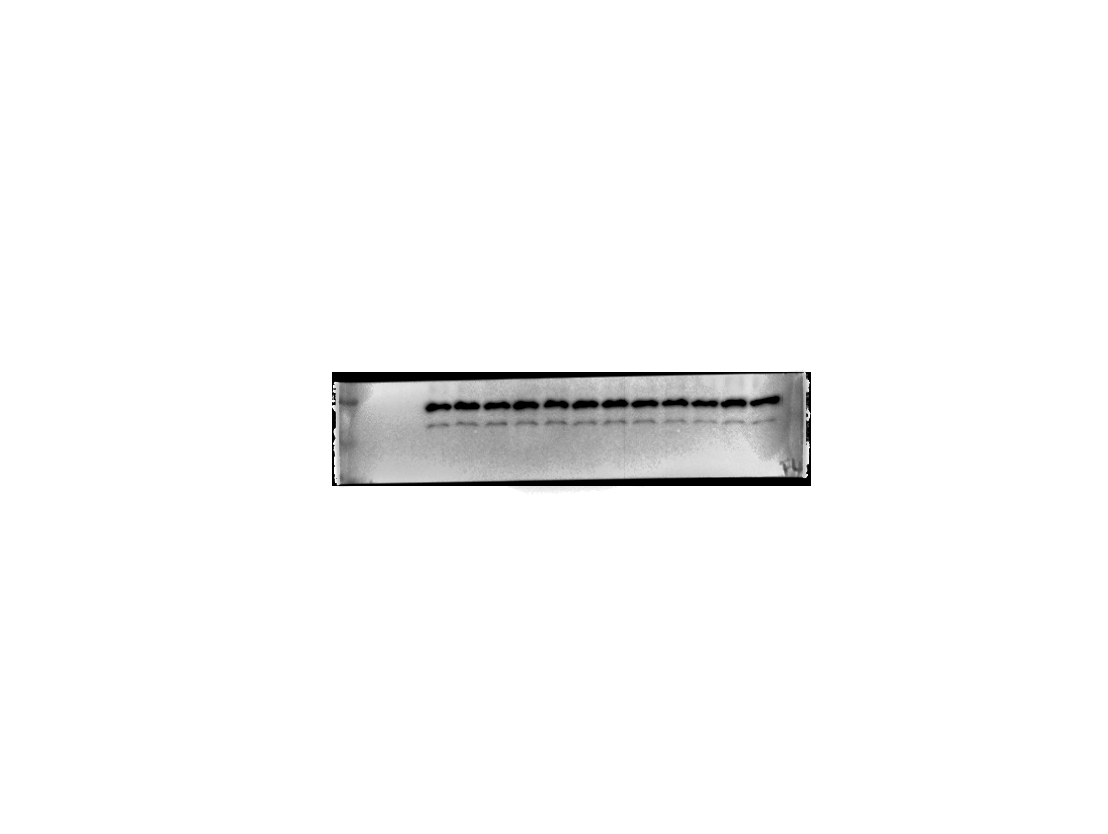

Supplement: Figure 3—source data 2. [file elife-102277-fig3-data2.zip › Figure 3-source data 2/Figure 3F-source data 2/nsp16-IP.tif]

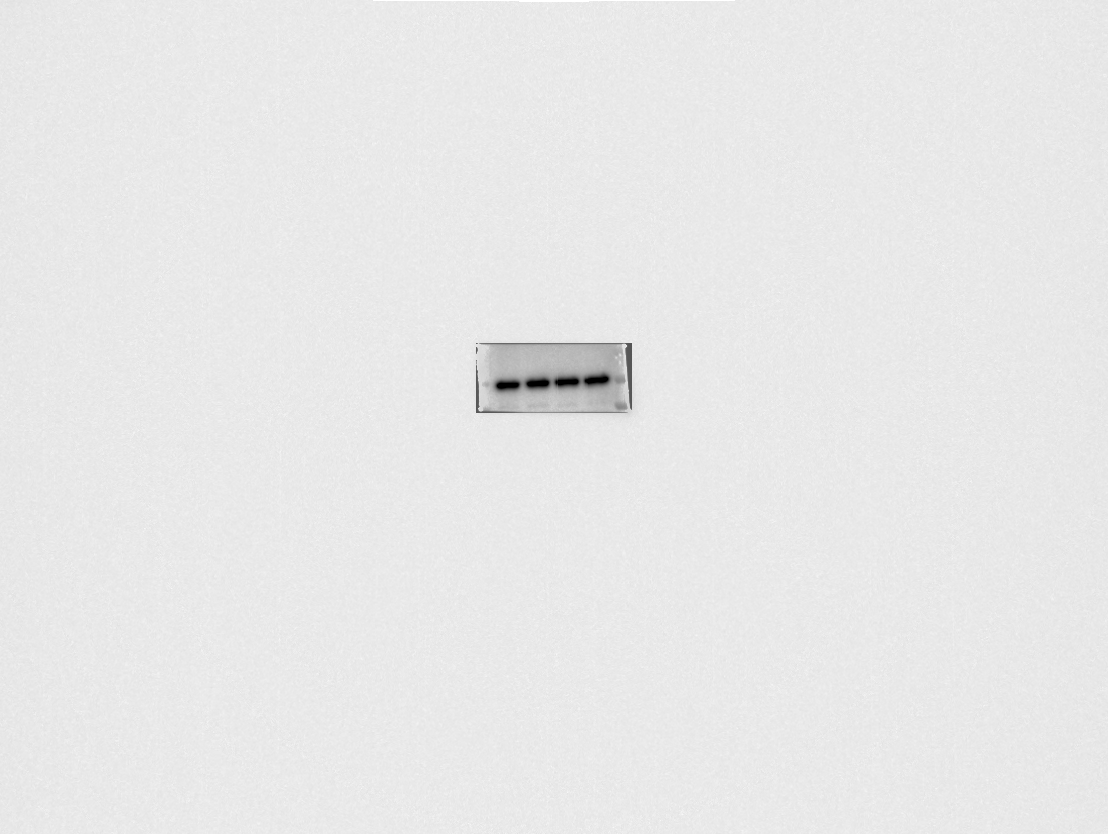

Supplement: Figure 3—source data 2. [file elife-102277-fig3-data2.zip › Figure 3-source data 2/Figure 3F-source data 2/Tubulin-input-2.tif]

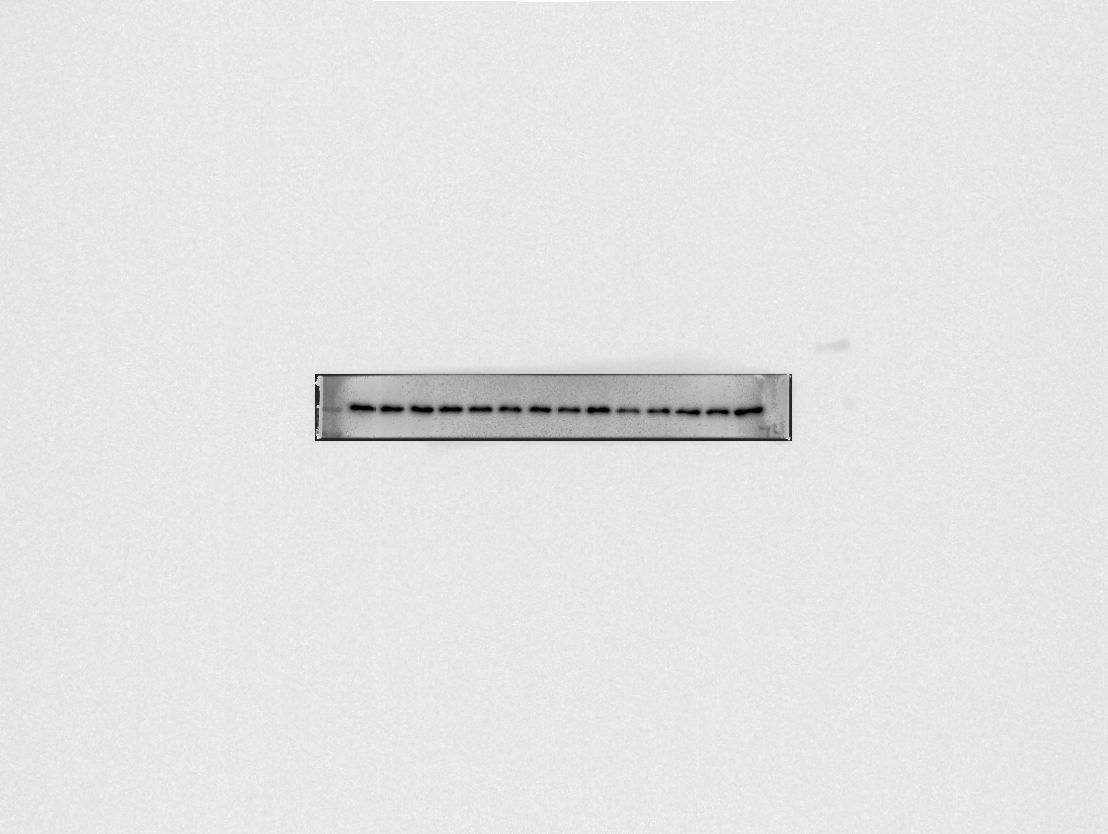

Supplement: Figure 3—source data 2. [file elife-102277-fig3-data2.zip › Figure 3-source data 2/Figure 3F-source data 2/Tubulin-input.tif]

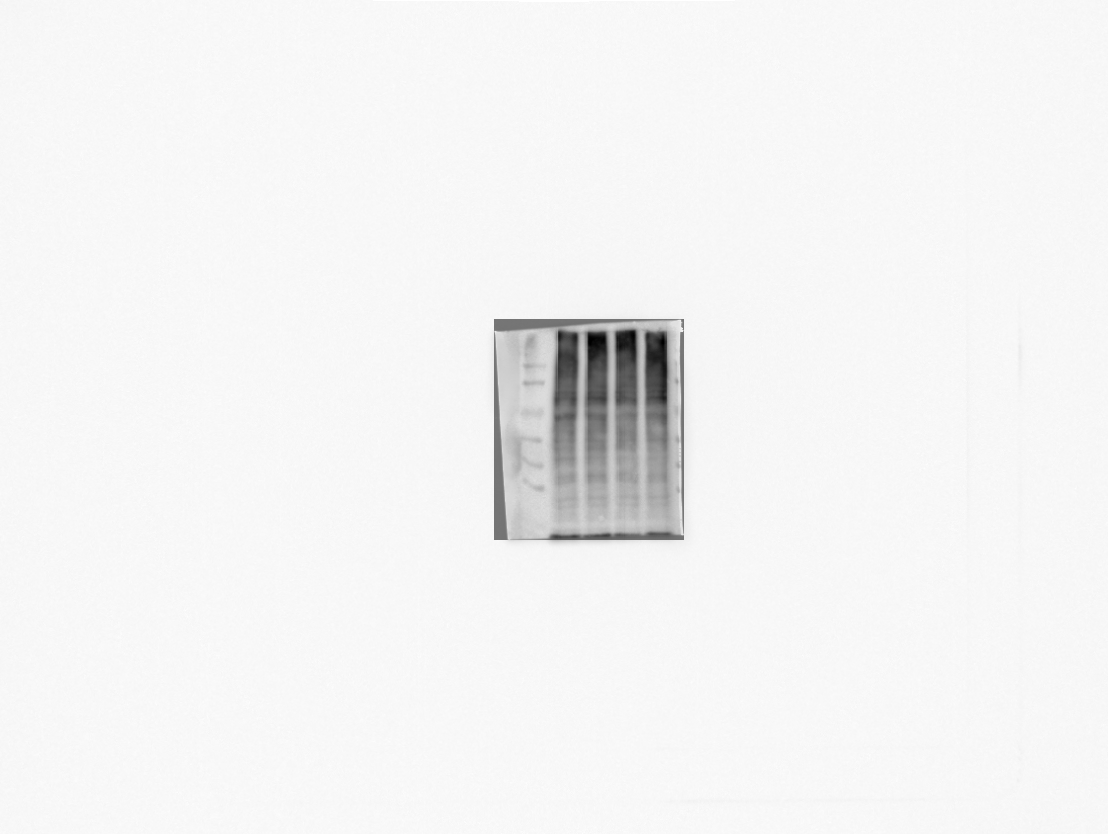

Supplement: Figure 3—source data 2. [file elife-102277-fig3-data2.zip › Figure 3-source data 2/Figure 3F-source data 2/Ub-input-2.tif]

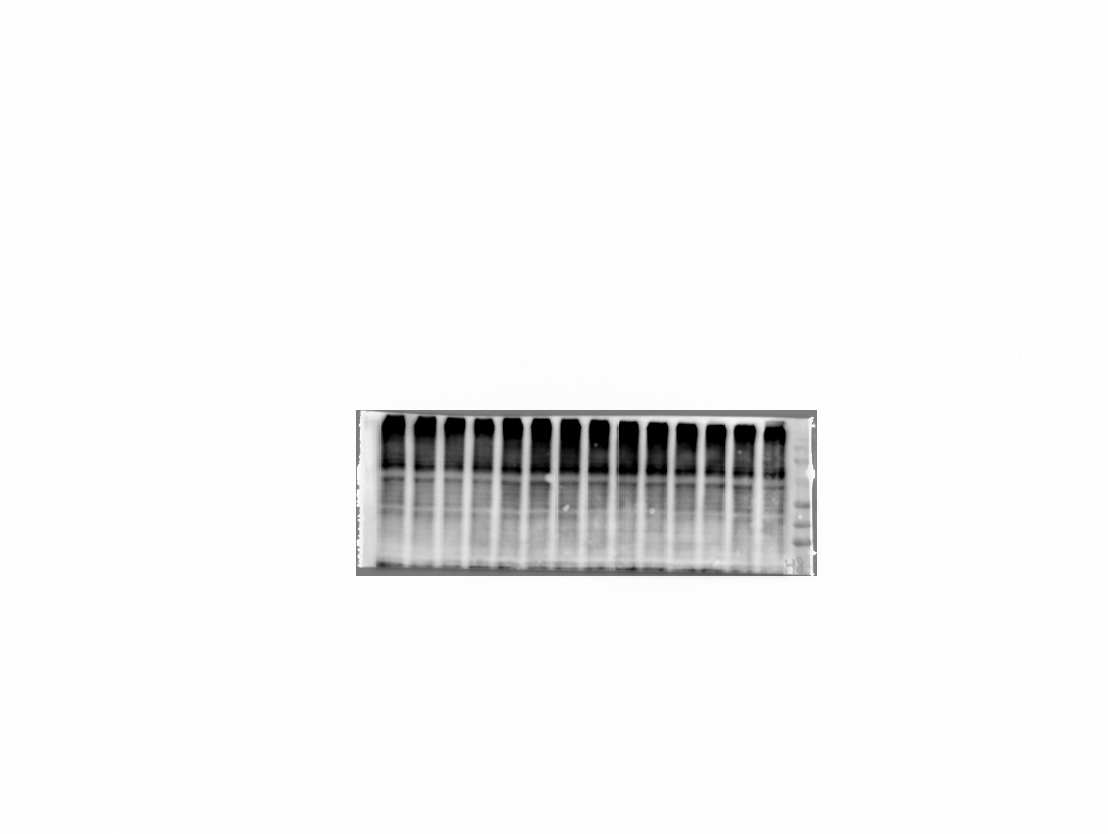

Supplement: Figure 3—source data 2. [file elife-102277-fig3-data2.zip › Figure 3-source data 2/Figure 3F-source data 2/Ub-input.tif]

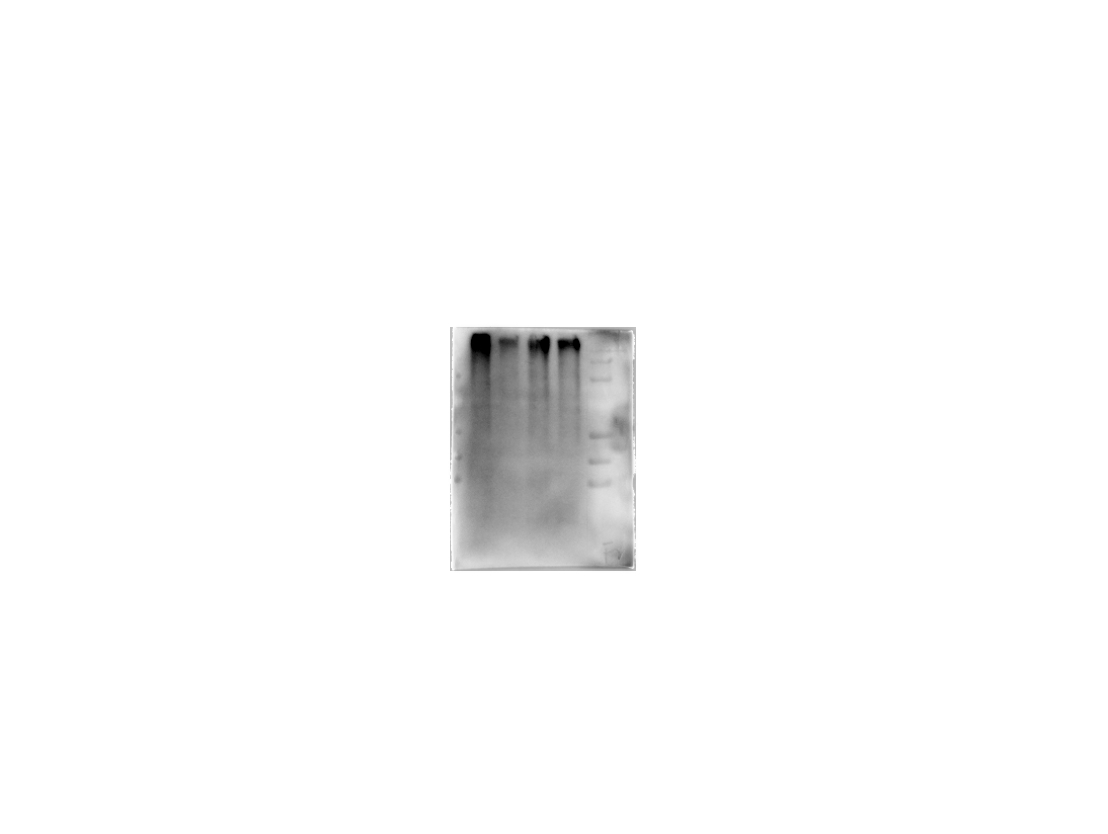

Supplement: Figure 3—source data 2. [file elife-102277-fig3-data2.zip › Figure 3-source data 2/Figure 3F-source data 2/Ub-IP-2.tif]

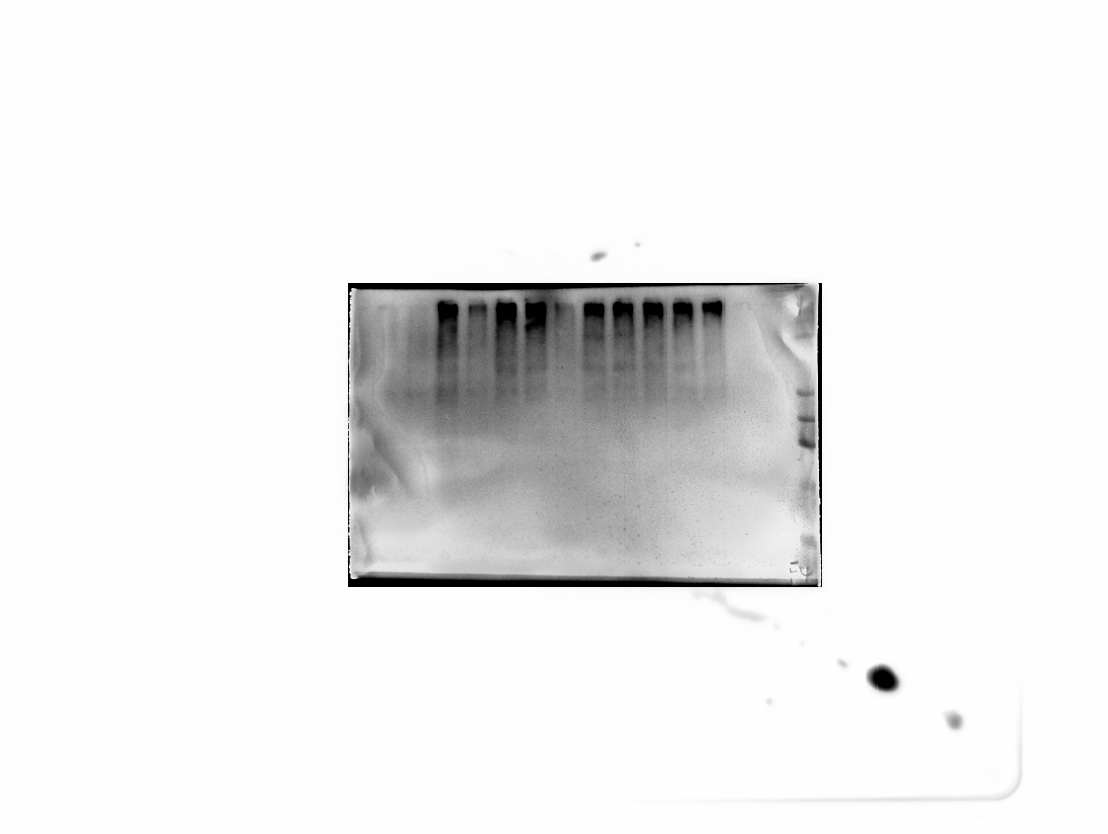

Supplement: Figure 3—source data 2. [file elife-102277-fig3-data2.zip › Figure 3-source data 2/Figure 3F-source data 2/Ub-IP.tif]

### Figure.4A

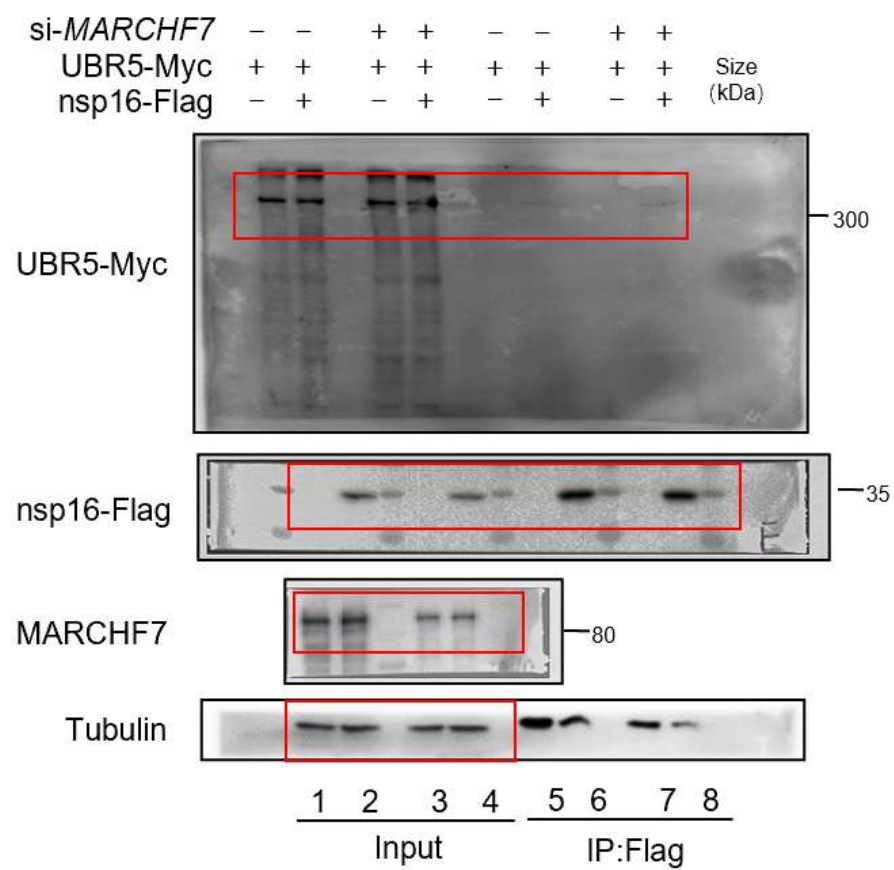

Supplement: Figure 4—source data 1. [file elife-102277-fig4-data1.zip › Figure 4-source data 1/Figure 4A-source data 1.pdf]

Figure 4B

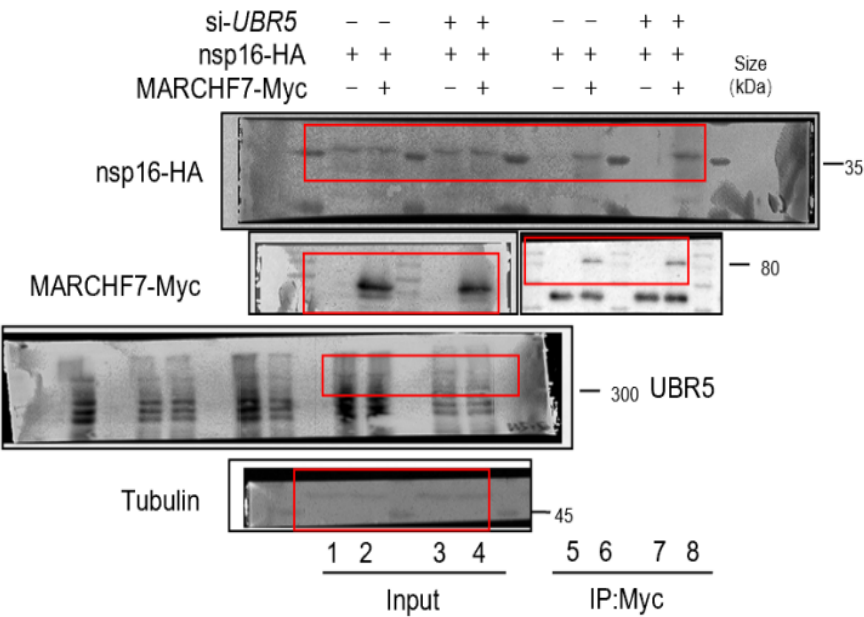

Supplement: Figure 4—source data 1. [file elife-102277-fig4-data1.zip › Figure 4-source data 1/Figure 4B-source data 1.pdf]

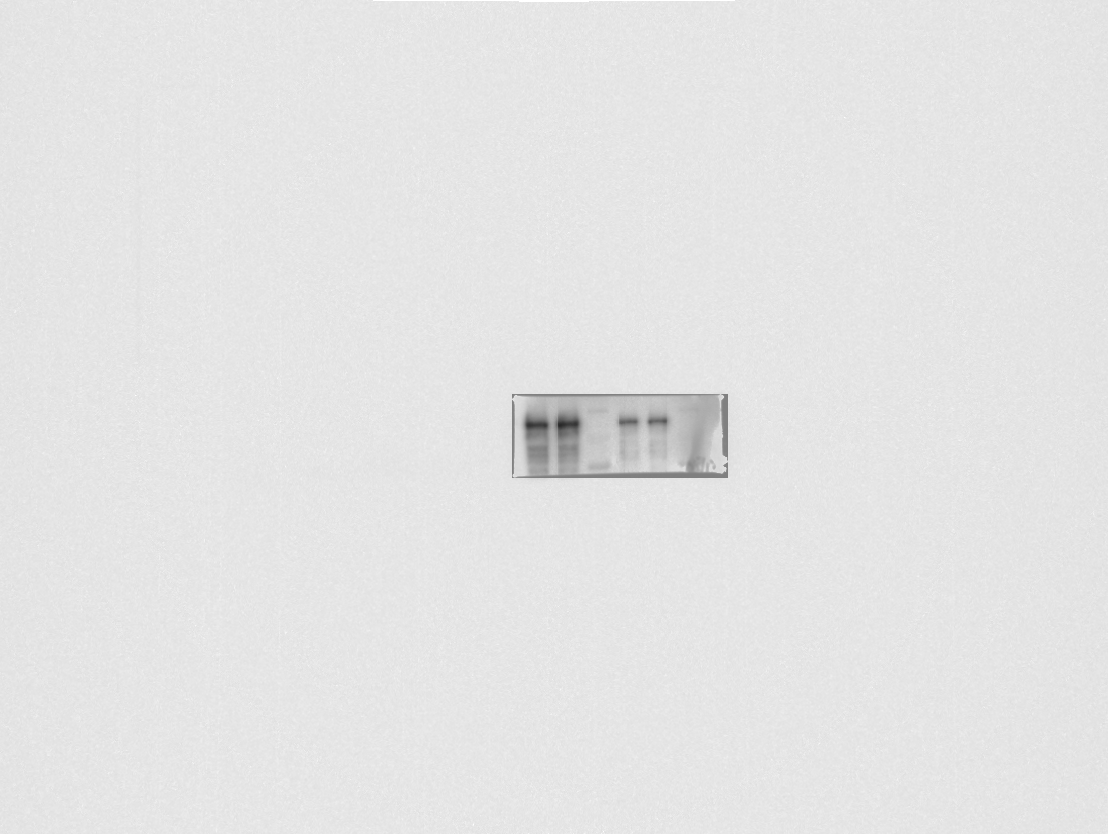

Supplement: Figure 4—source data 2. [file elife-102277-fig4-data2.zip › Figure 4-source data 2/Figure 4A-source data 2/MARCHF7tif.tif]

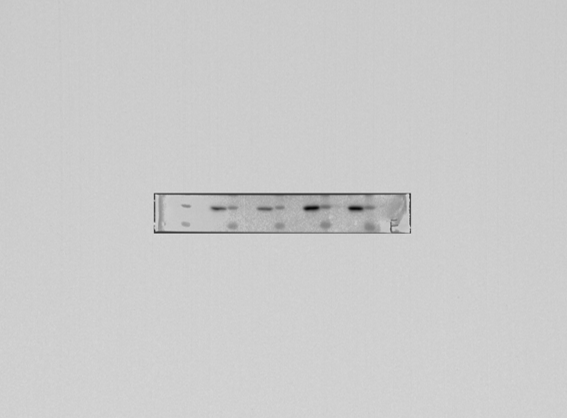

Supplement: Figure 4—source data 2. [file elife-102277-fig4-data2.zip › Figure 4-source data 2/Figure 4A-source data 2/nsp16-Flag.tif]

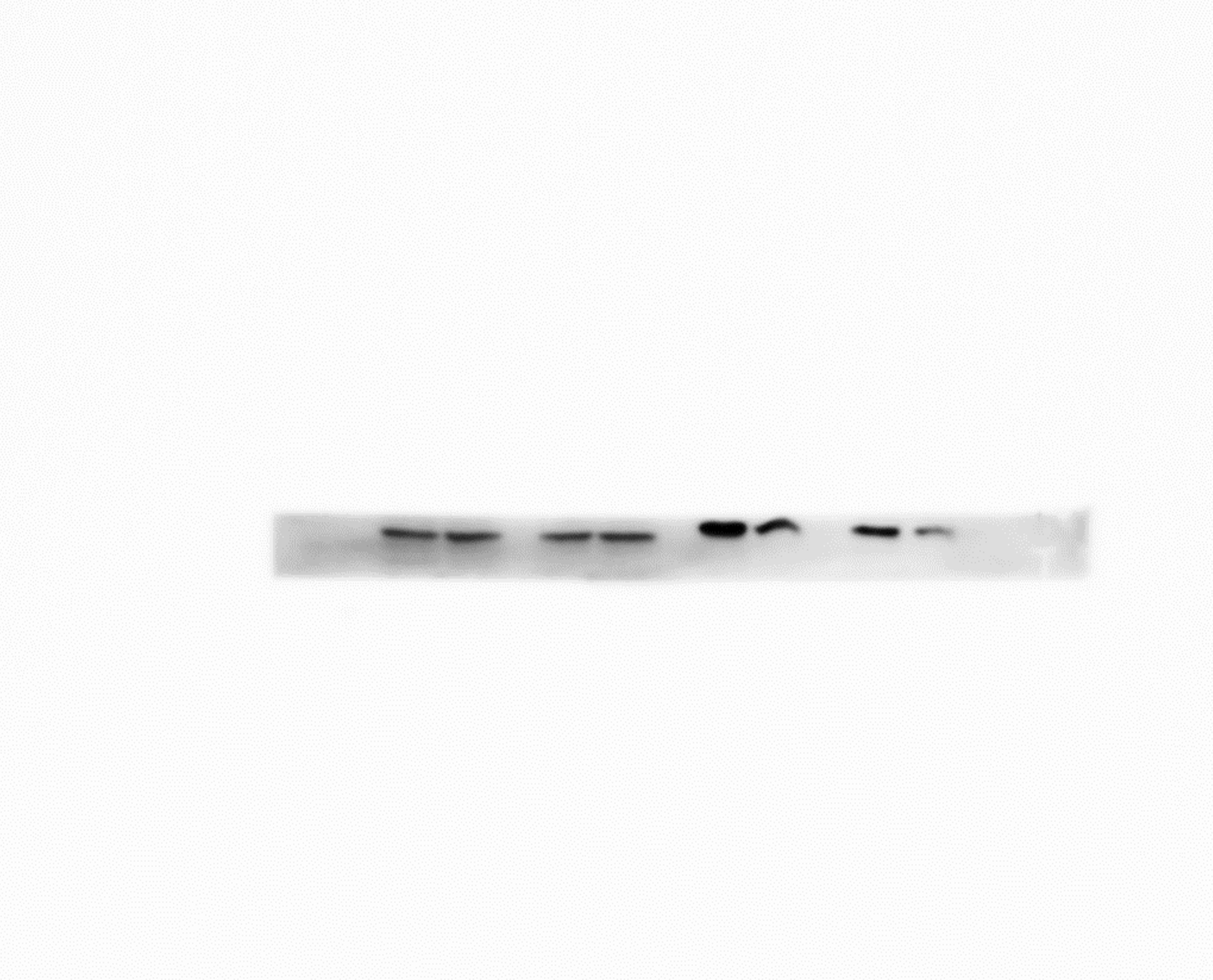

Supplement: Figure 4—source data 2. [file elife-102277-fig4-data2.zip › Figure 4-source data 2/Figure 4A-source data 2/Tubulin.tif]

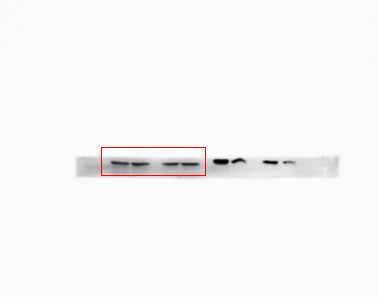

Supplement: Figure 4—source data 2. [file elife-102277-fig4-data2.zip › Figure 4-source data 2/Figure 4A-source data 2/Tubulin_2.tif]

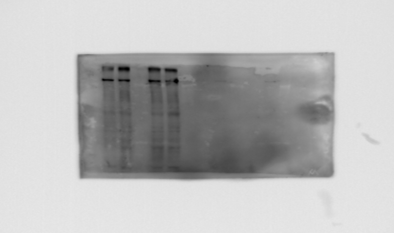

Supplement: Figure 4—source data 2. [file elife-102277-fig4-data2.zip › Figure 4-source data 2/Figure 4A-source data 2/UBR5.tif]

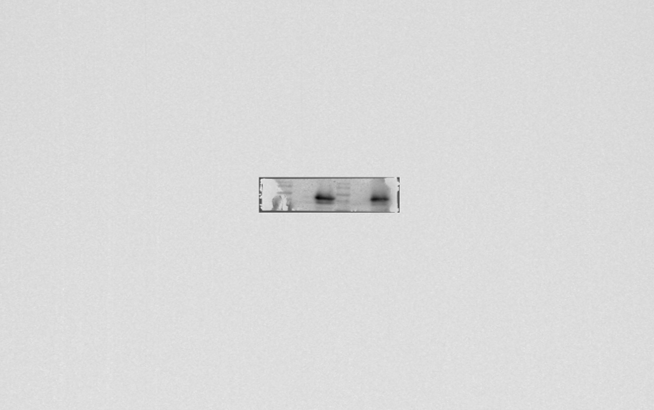

Supplement: Figure 4—source data 2. [file elife-102277-fig4-data2.zip › Figure 4-source data 2/Figure 4B-source data 2/MARCHF7 input.tif]

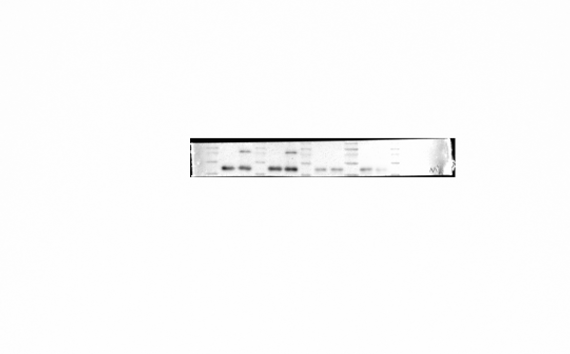

Supplement: Figure 4—source data 2. [file elife-102277-fig4-data2.zip › Figure 4-source data 2/Figure 4B-source data 2/MARCHF7 IP.tif]
