# Supplementary figures and images for: SARS-CoV-2 nsp16 is regulated by host E3 ubiquitin ligases, UBR5 and MARCHF7 (part 2 of 3)
Source: eLife. 2025 May 13;13:RP102277. doi: 10.7554/eLife.102277 (PMC12074641; doi:10.7554/eLife.102277)

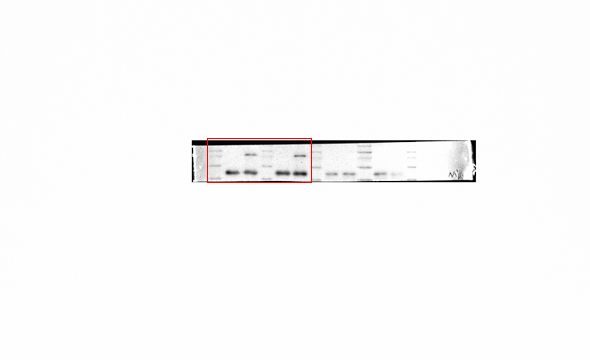

Supplement: Figure 4—source data 2. [file elife-102277-fig4-data2.zip › Figure 4-source data 2/Figure 4B-source data 2/MARCHF7 IP_2.tif]

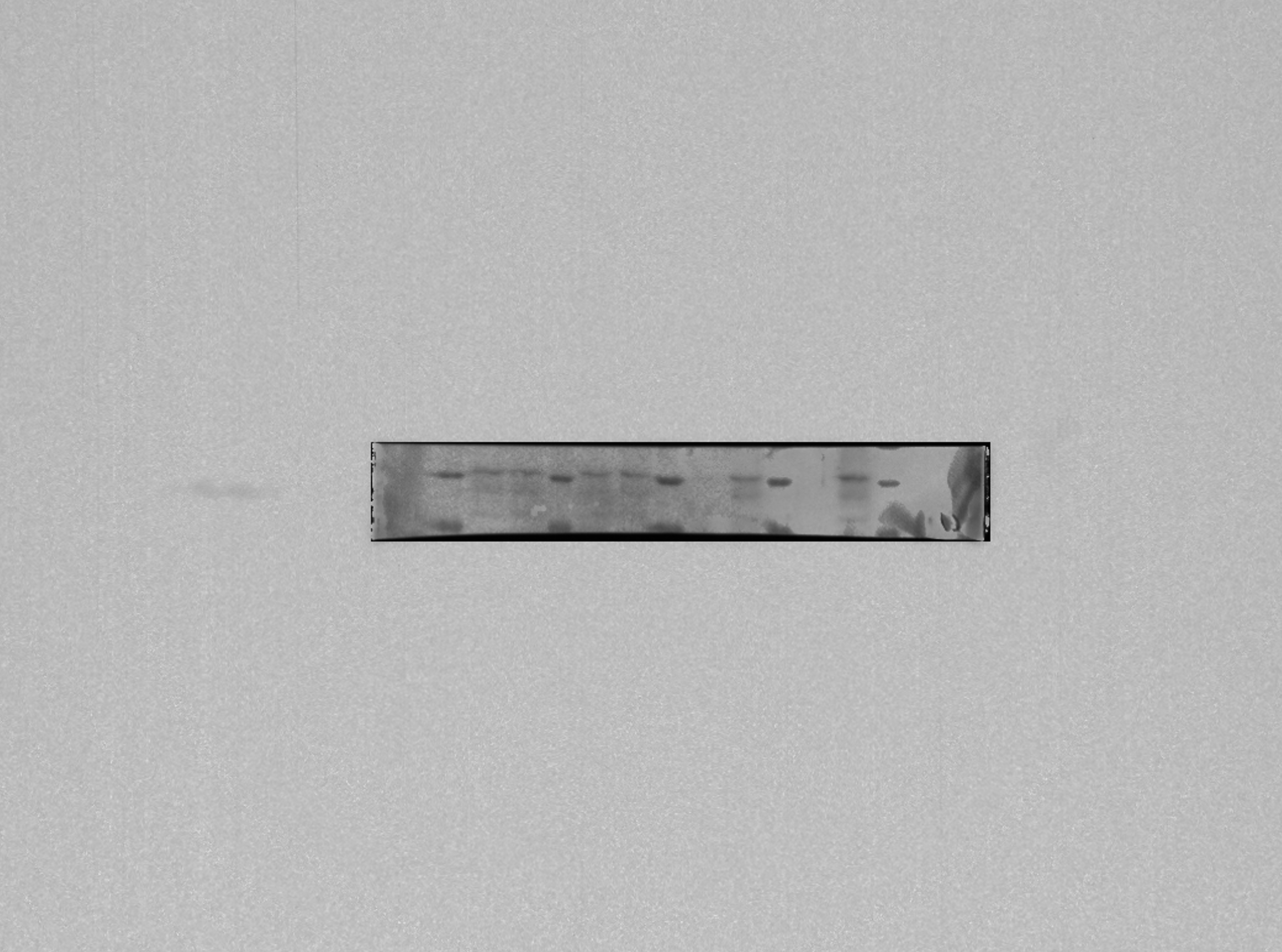

Supplement: Figure 4—source data 2. [file elife-102277-fig4-data2.zip › Figure 4-source data 2/Figure 4B-source data 2/nsp16-HA.tif]

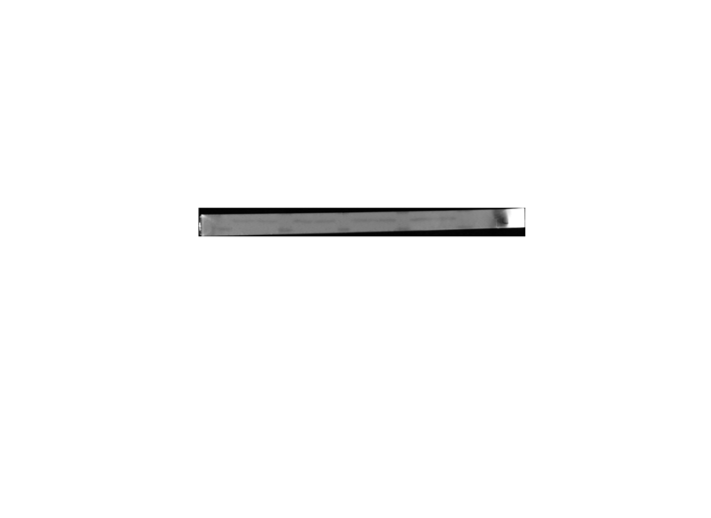

Supplement: Figure 4—source data 2. [file elife-102277-fig4-data2.zip › Figure 4-source data 2/Figure 4B-source data 2/Tubulin.tif]

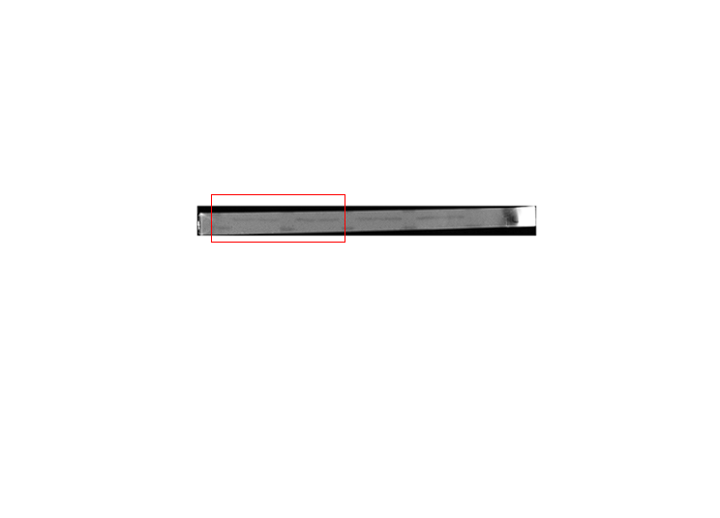

Supplement: Figure 4—source data 2. [file elife-102277-fig4-data2.zip › Figure 4-source data 2/Figure 4B-source data 2/Tubulin_2.tif]

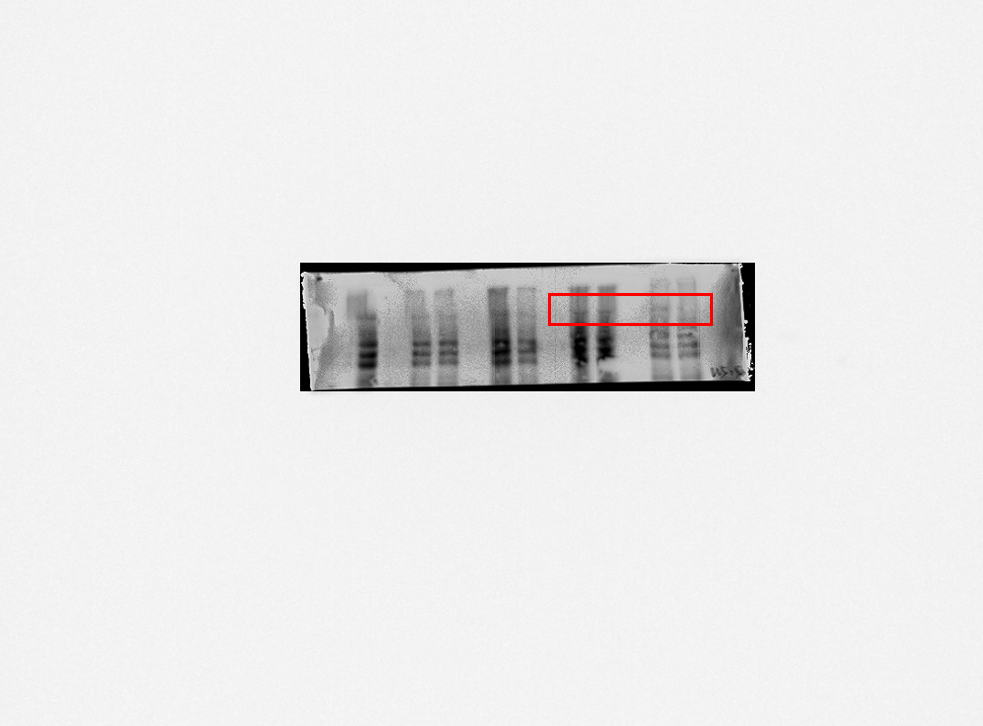

Supplement: Figure 4—source data 2. [file elife-102277-fig4-data2.zip › Figure 4-source data 2/Figure 4B-source data 2/UBR5-1.tif]

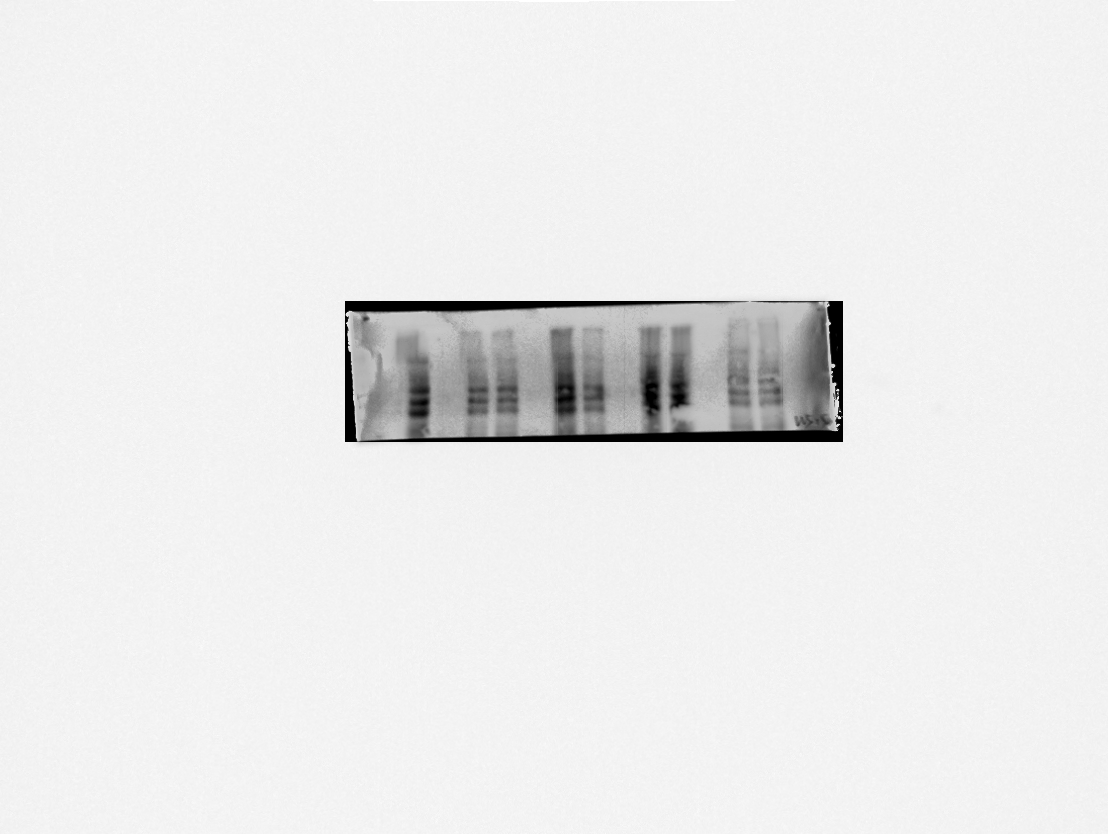

Supplement: Figure 4—source data 2. [file elife-102277-fig4-data2.zip › Figure 4-source data 2/Figure 4B-source data 2/UBR5.tif]

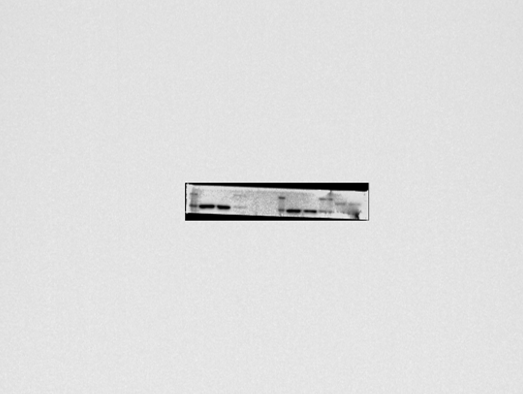

Supplement: Figure 4—figure supplement 1—source data 2. [file elife-102277-fig4-figsupp1-data2.zip › Figure 4—figure supplement 1-source data 2/Figure 4—figure supplement 1A-source data 2/ACTIN.tif]

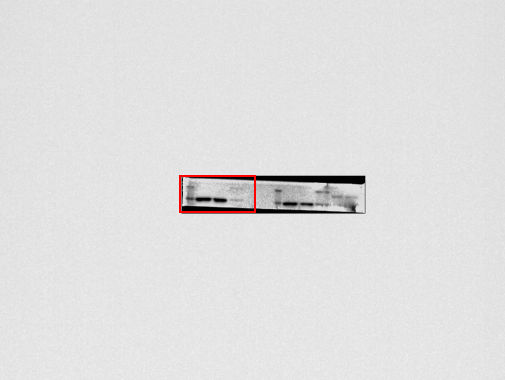

Supplement: Figure 4—figure supplement 1—source data 2. [file elife-102277-fig4-figsupp1-data2.zip › Figure 4—figure supplement 1-source data 2/Figure 4—figure supplement 1A-source data 2/ACTIN_2.tif]

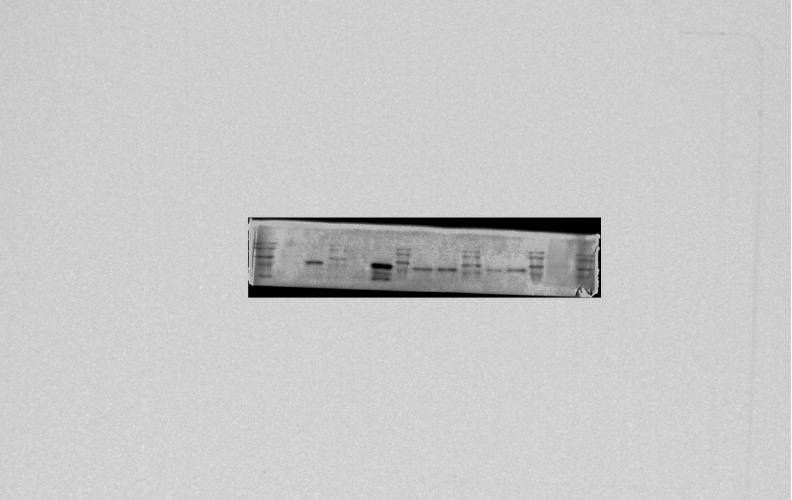

Supplement: Figure 4—figure supplement 1—source data 2. [file elife-102277-fig4-figsupp1-data2.zip › Figure 4—figure supplement 1-source data 2/Figure 4—figure supplement 1A-source data 2/MARCHF7.tif]

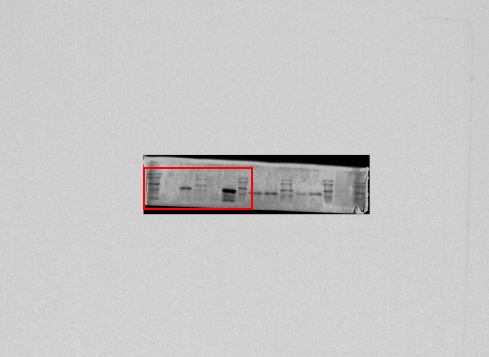

Supplement: Figure 4—figure supplement 1—source data 2. [file elife-102277-fig4-figsupp1-data2.zip › Figure 4—figure supplement 1-source data 2/Figure 4—figure supplement 1A-source data 2/MARCHF7_2.tif]

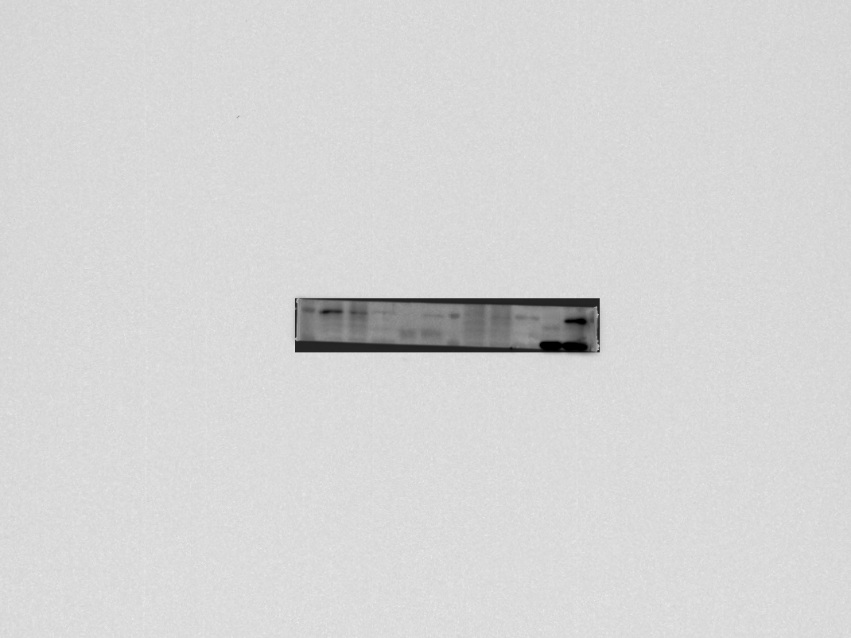

Supplement: Figure 4—figure supplement 1—source data 2. [file elife-102277-fig4-figsupp1-data2.zip › Figure 4—figure supplement 1-source data 2/Figure 4—figure supplement 1A-source data 2/nsp16-Flag.tif]

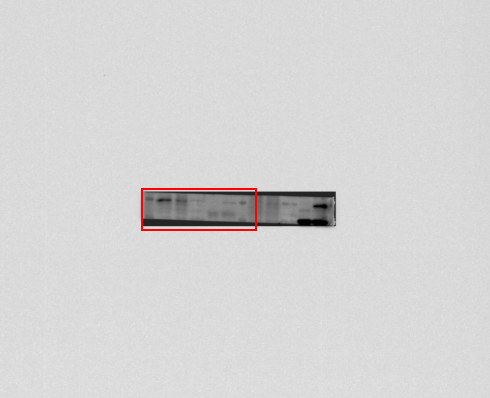

Supplement: Figure 4—figure supplement 1—source data 2. [file elife-102277-fig4-figsupp1-data2.zip › Figure 4—figure supplement 1-source data 2/Figure 4—figure supplement 1A-source data 2/nsp16-Flag_2.tif]

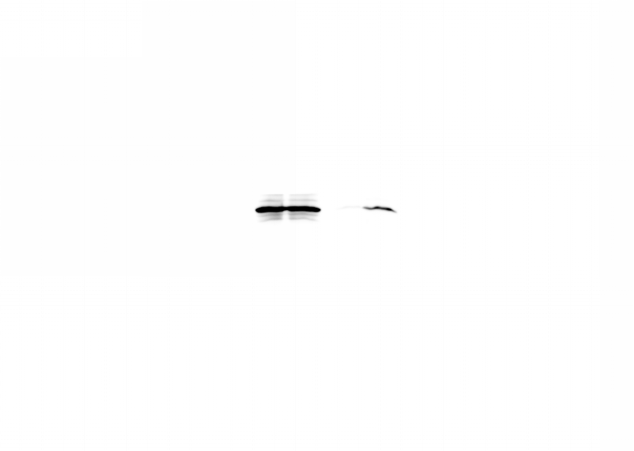

Supplement: Figure 4—figure supplement 1—source data 2. [file elife-102277-fig4-figsupp1-data2.zip › Figure 4—figure supplement 1-source data 2/Figure 4—figure supplement 1B-source data 2/nsp16-Flag.tif]

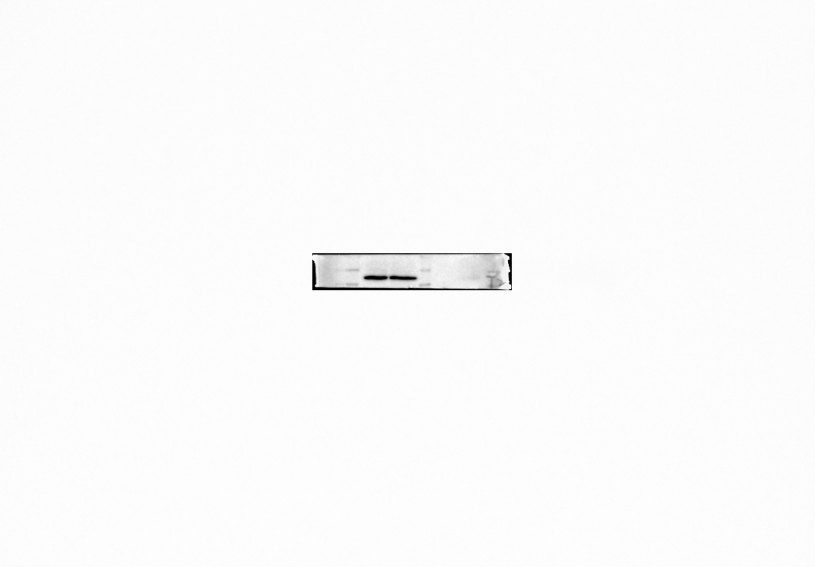

Supplement: Figure 4—figure supplement 1—source data 2. [file elife-102277-fig4-figsupp1-data2.zip › Figure 4—figure supplement 1-source data 2/Figure 4—figure supplement 1B-source data 2/Tubulin.tif]

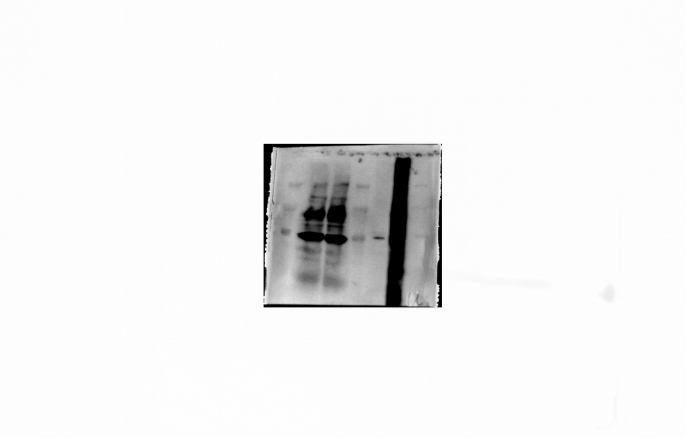

Supplement: Figure 4—figure supplement 1—source data 2. [file elife-102277-fig4-figsupp1-data2.zip › Figure 4—figure supplement 1-source data 2/Figure 4—figure supplement 1B-source data 2/UBR5.tif]

Figure 4—figure supplement 2B

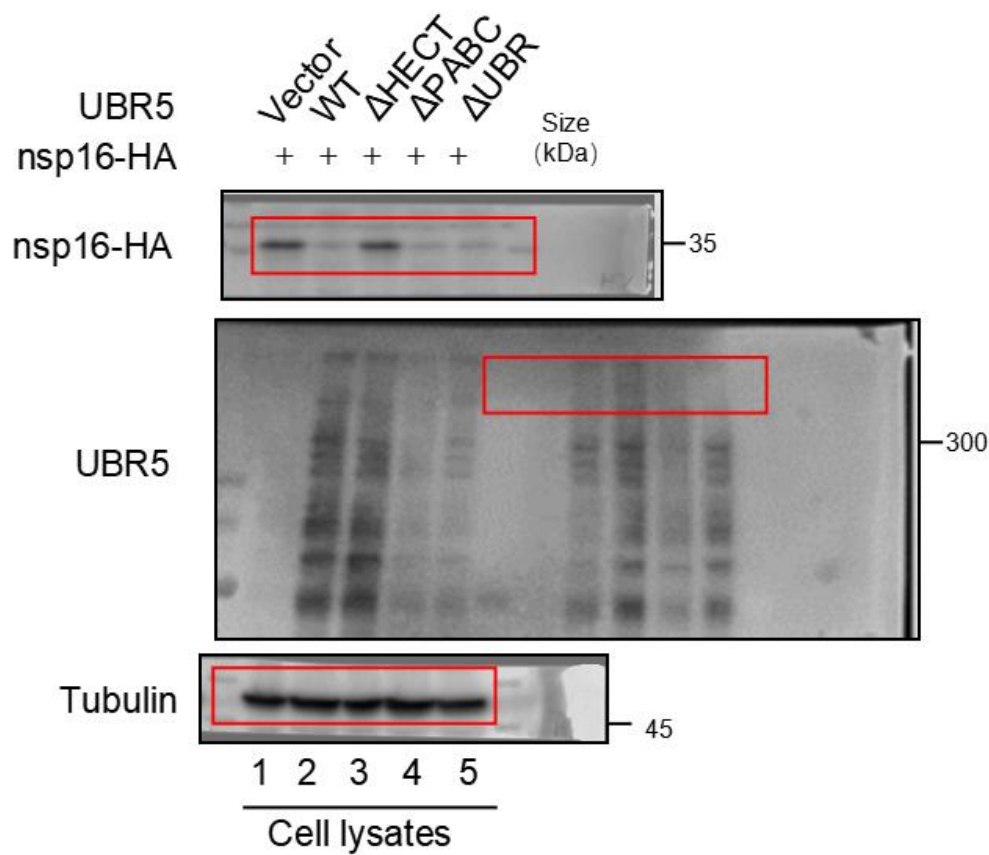

Supplement: Figure 4—figure supplement 2—source data 1. [file elife-102277-fig4-figsupp2-data1.zip › Figure 4—figure supplement 2-source data 1/Figure 4—figure supplement 2B-source data 1.pdf]

Figure 4—figure supplement 2C

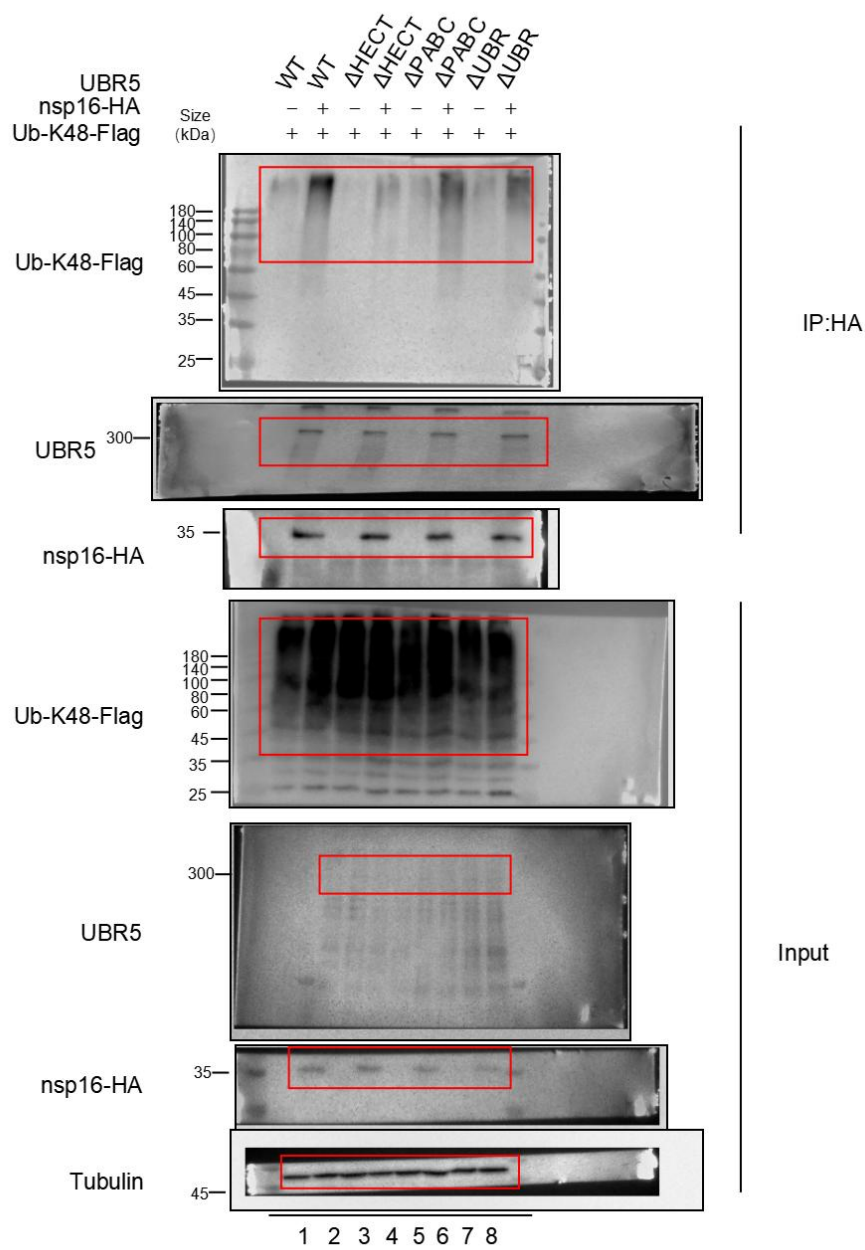

Supplement: Figure 4—figure supplement 2—source data 1. [file elife-102277-fig4-figsupp2-data1.zip › Figure 4—figure supplement 2-source data 1/Figure 4—figure supplement 2C-source data 1.pdf]

Figure 4—figure supplement 2E

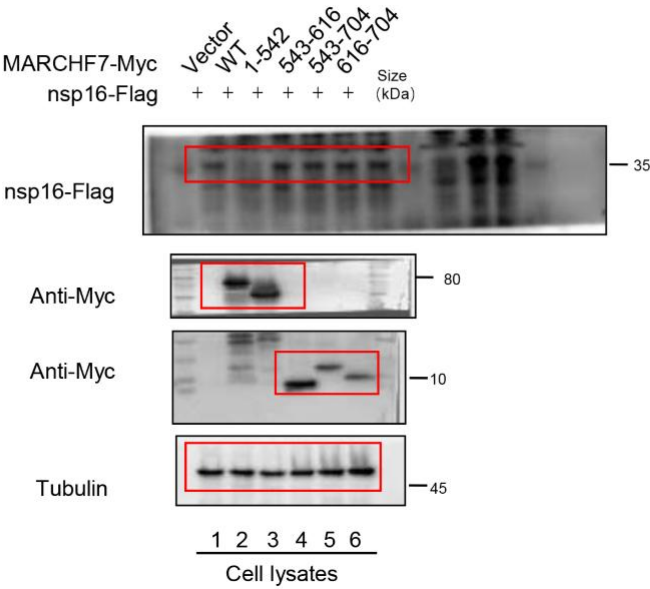

Supplement: Figure 4—figure supplement 2—source data 1. [file elife-102277-fig4-figsupp2-data1.zip › Figure 4—figure supplement 2-source data 1/Figure 4—figure supplement 2E-source data 1.pdf]

Figure 4—figure supplement 2F

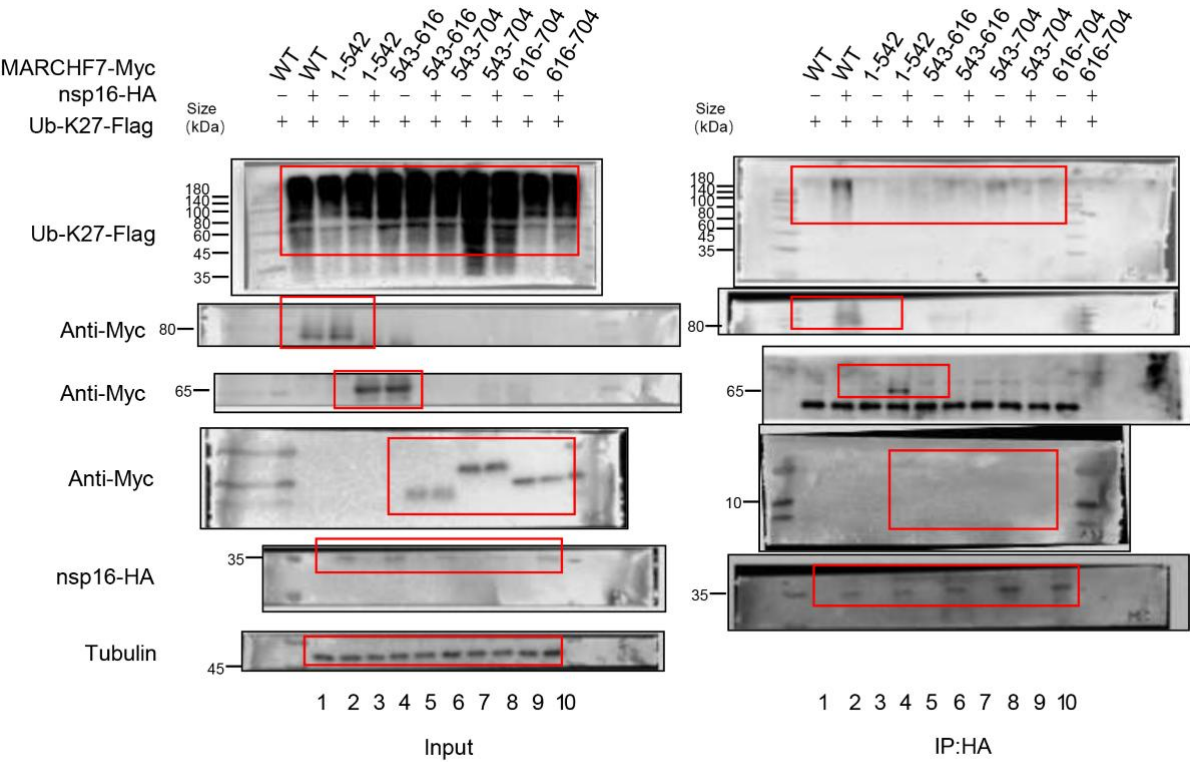

Supplement: Figure 4—figure supplement 2—source data 1. [file elife-102277-fig4-figsupp2-data1.zip › Figure 4—figure supplement 2-source data 1/Figure 4—figure supplement 2F-source data 1.pdf]

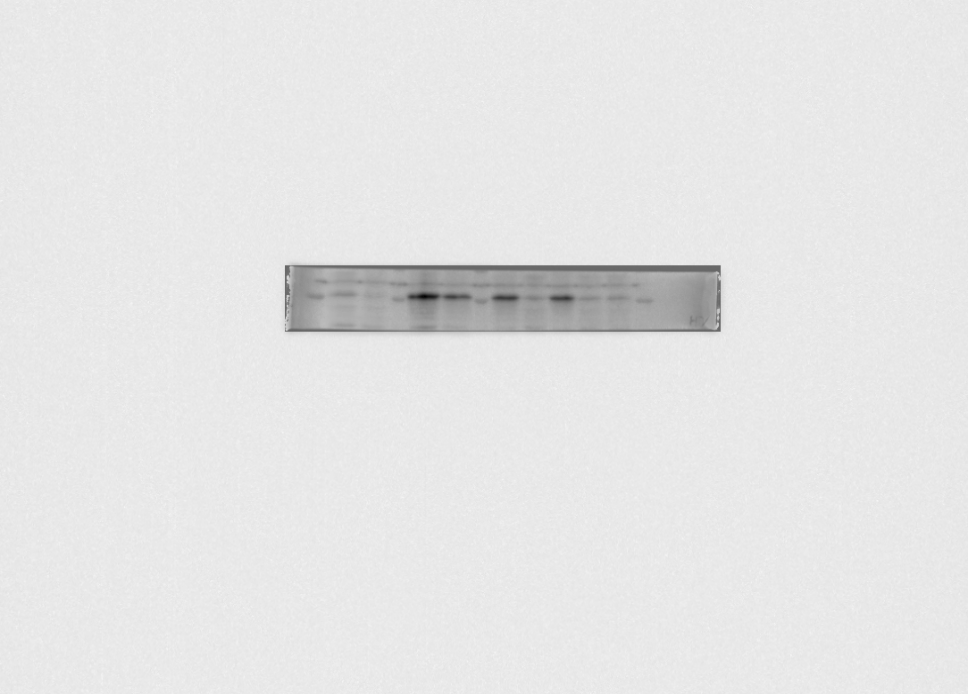

Supplement: Figure 4—figure supplement 2—source data 2. [file elife-102277-fig4-figsupp2-data2.zip › Figure 4—figure supplement 2-source data 2/Figure 4—figure supplement 2B-source data 2/nsp16-HA.tif]

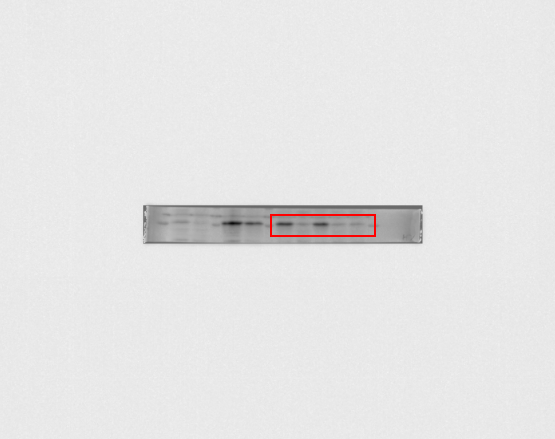

Supplement: Figure 4—figure supplement 2—source data 2. [file elife-102277-fig4-figsupp2-data2.zip › Figure 4—figure supplement 2-source data 2/Figure 4—figure supplement 2B-source data 2/nsp16-HA_2.tif]

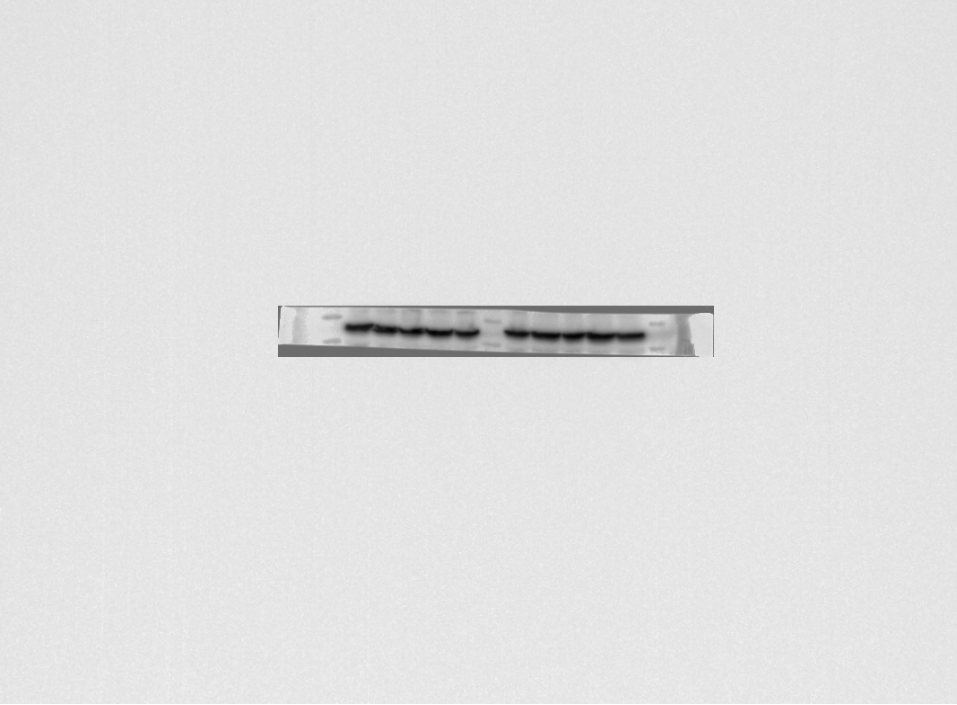

Supplement: Figure 4—figure supplement 2—source data 2. [file elife-102277-fig4-figsupp2-data2.zip › Figure 4—figure supplement 2-source data 2/Figure 4—figure supplement 2B-source data 2/Tubulin.tif]

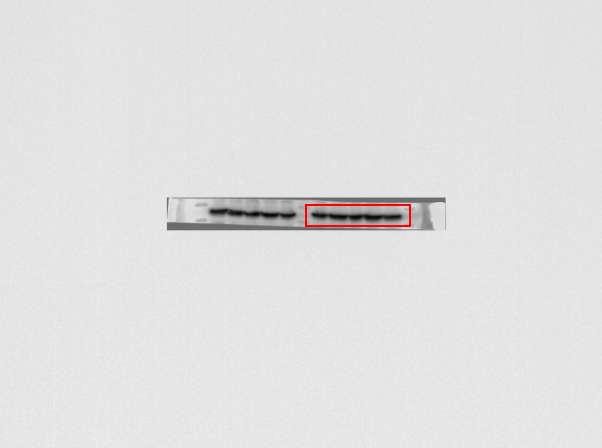

Supplement: Figure 4—figure supplement 2—source data 2. [file elife-102277-fig4-figsupp2-data2.zip › Figure 4—figure supplement 2-source data 2/Figure 4—figure supplement 2B-source data 2/Tubulin_2.tif]

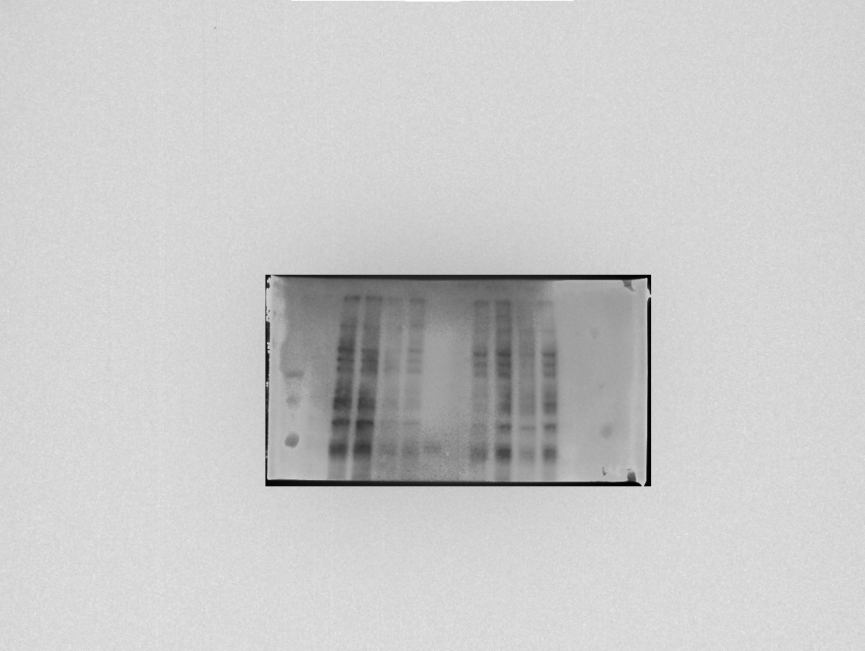

Supplement: Figure 4—figure supplement 2—source data 2. [file elife-102277-fig4-figsupp2-data2.zip › Figure 4—figure supplement 2-source data 2/Figure 4—figure supplement 2B-source data 2/UBR5.tif]

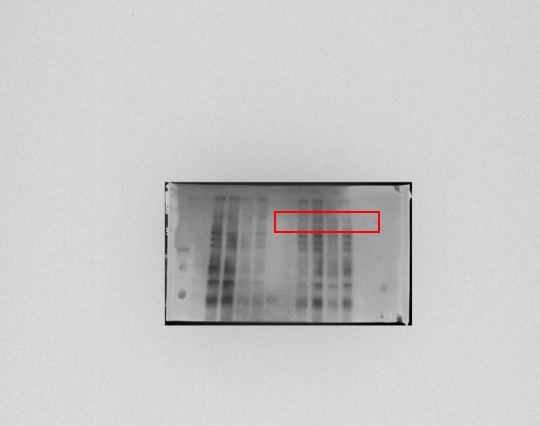

Supplement: Figure 4—figure supplement 2—source data 2. [file elife-102277-fig4-figsupp2-data2.zip › Figure 4—figure supplement 2-source data 2/Figure 4—figure supplement 2B-source data 2/UBR5_2.tif]

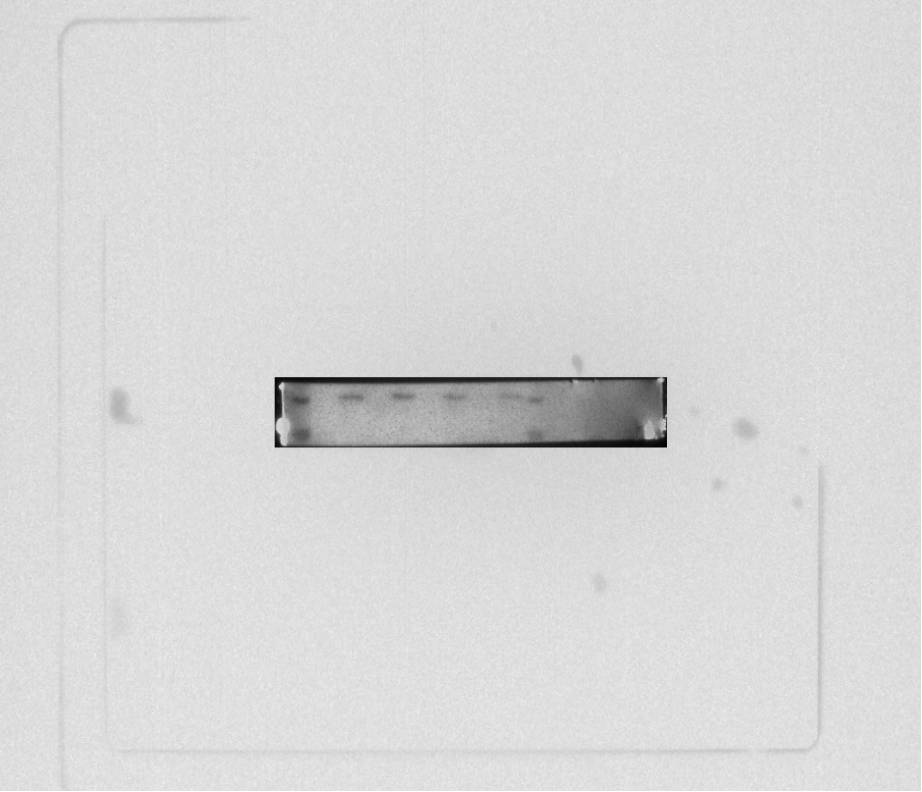

Supplement: Figure 4—figure supplement 2—source data 2. [file elife-102277-fig4-figsupp2-data2.zip › Figure 4—figure supplement 2-source data 2/Figure 4—figure supplement 2C-source data 2/nsp16-HA input.tif]

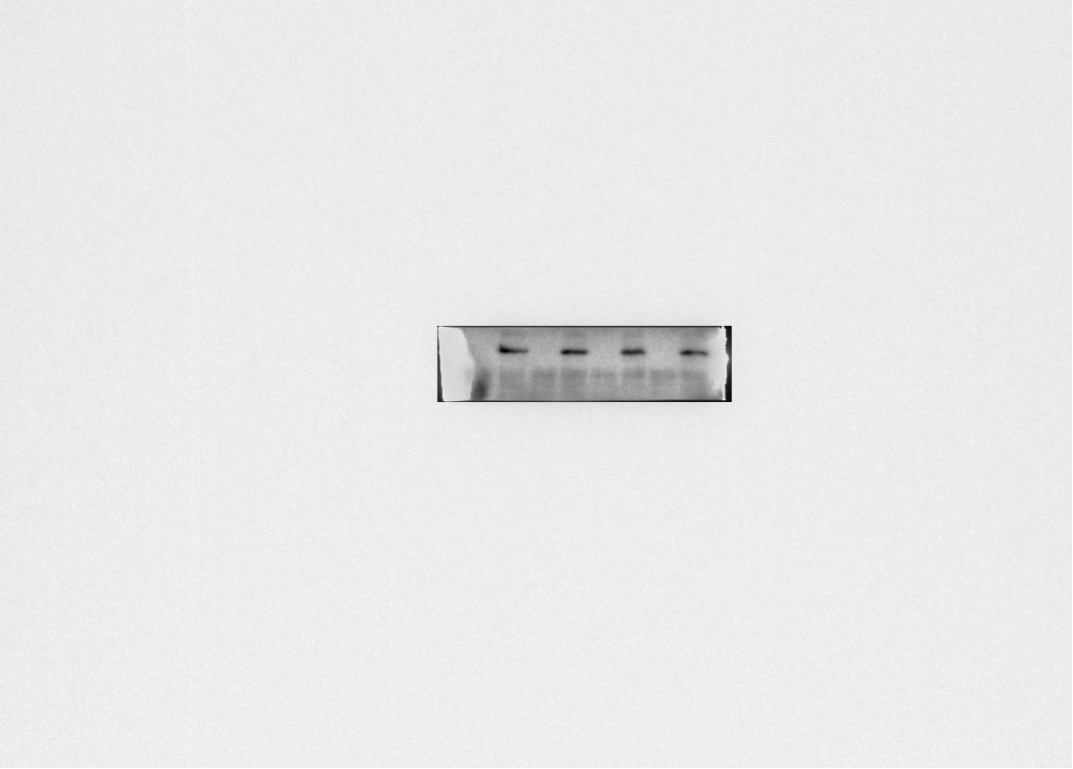

Supplement: Figure 4—figure supplement 2—source data 2. [file elife-102277-fig4-figsupp2-data2.zip › Figure 4—figure supplement 2-source data 2/Figure 4—figure supplement 2C-source data 2/nsp16-HA IP.tif]

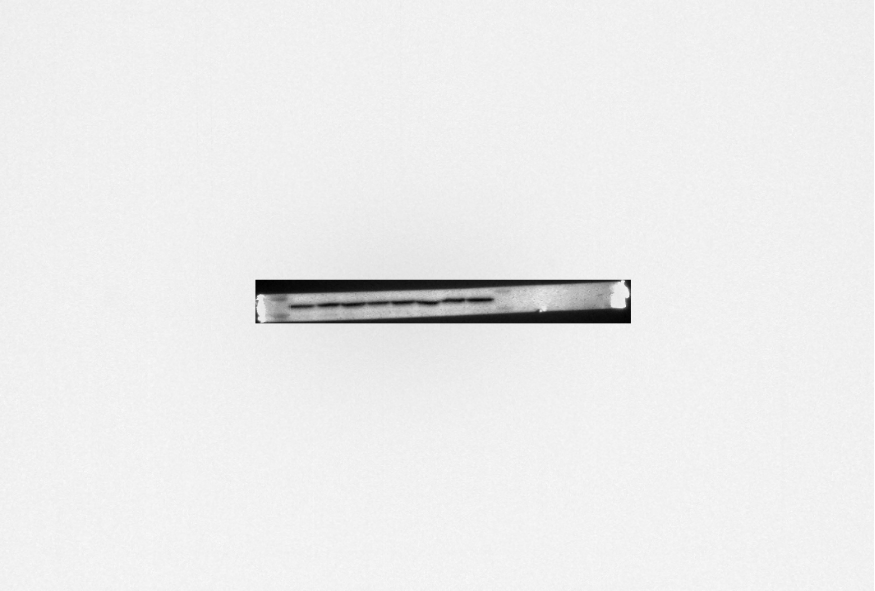

Supplement: Figure 4—figure supplement 2—source data 2. [file elife-102277-fig4-figsupp2-data2.zip › Figure 4—figure supplement 2-source data 2/Figure 4—figure supplement 2C-source data 2/Tubulin input.tif]

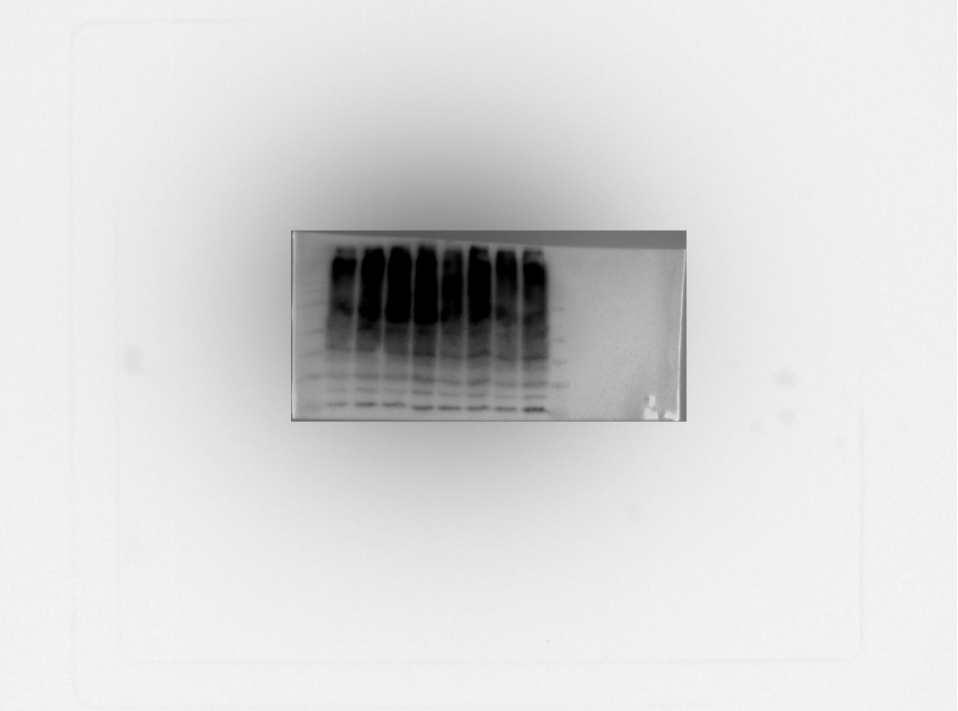

Supplement: Figure 4—figure supplement 2—source data 2. [file elife-102277-fig4-figsupp2-data2.zip › Figure 4—figure supplement 2-source data 2/Figure 4—figure supplement 2C-source data 2/Ub-Flag input.tif]

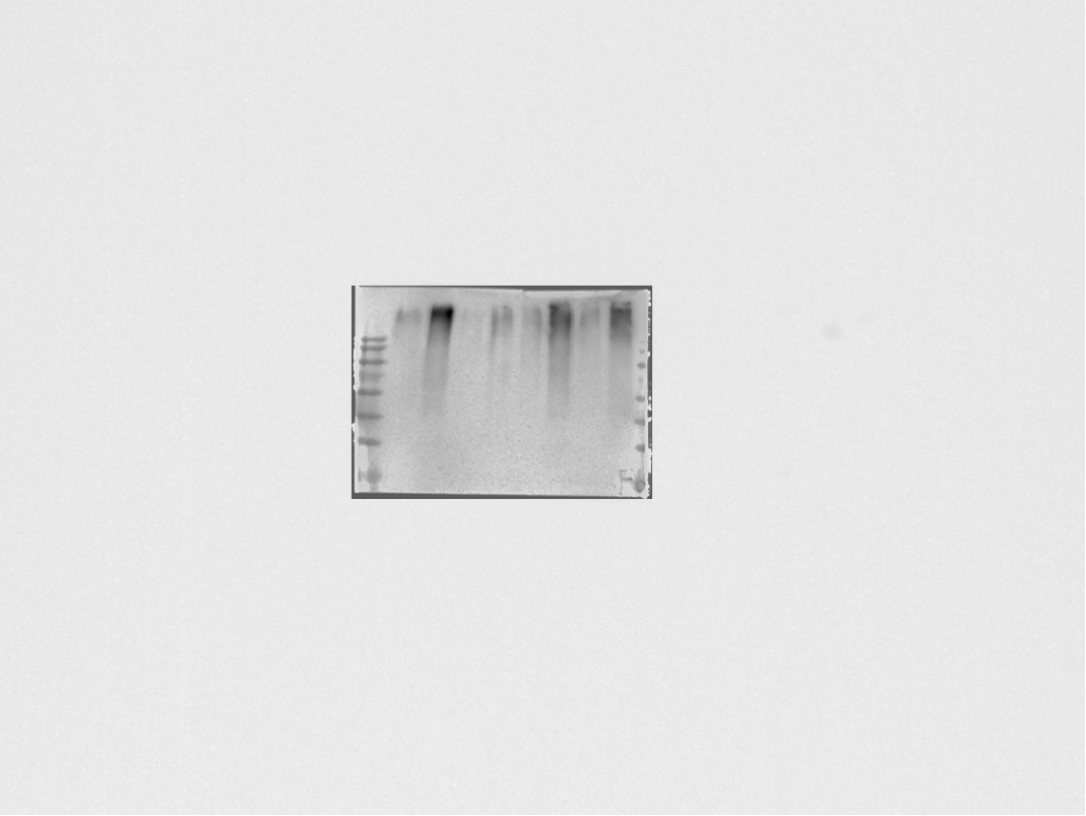

Supplement: Figure 4—figure supplement 2—source data 2. [file elife-102277-fig4-figsupp2-data2.zip › Figure 4—figure supplement 2-source data 2/Figure 4—figure supplement 2C-source data 2/Ub-Flag IP.tif]

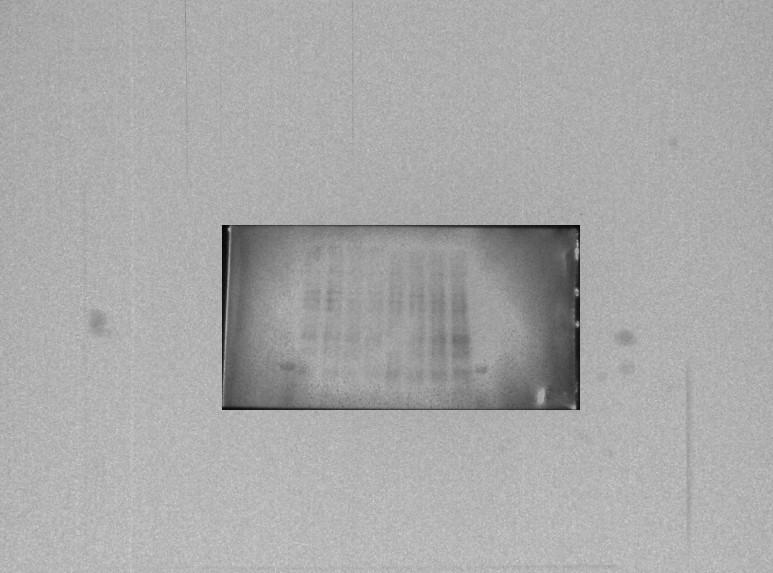

Supplement: Figure 4—figure supplement 2—source data 2. [file elife-102277-fig4-figsupp2-data2.zip › Figure 4—figure supplement 2-source data 2/Figure 4—figure supplement 2C-source data 2/UBR5 input.tif]

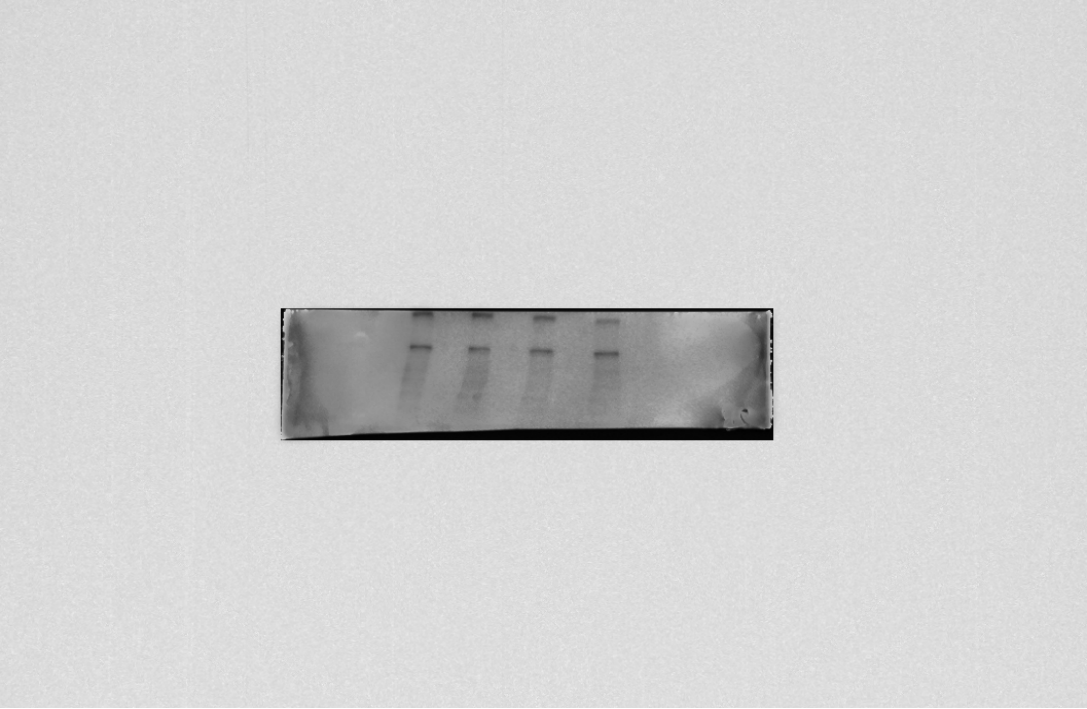

Supplement: Figure 4—figure supplement 2—source data 2. [file elife-102277-fig4-figsupp2-data2.zip › Figure 4—figure supplement 2-source data 2/Figure 4—figure supplement 2C-source data 2/UBR5 IP.tif]

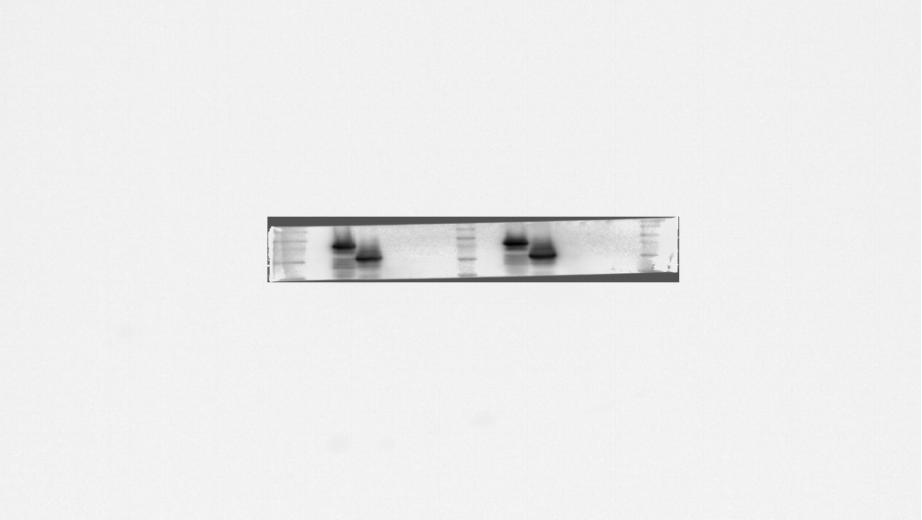

Supplement: Figure 4—figure supplement 2—source data 2. [file elife-102277-fig4-figsupp2-data2.zip › Figure 4—figure supplement 2-source data 2/Figure 4—figure supplement 2E-source data 2/anti-Myc-1.tif]

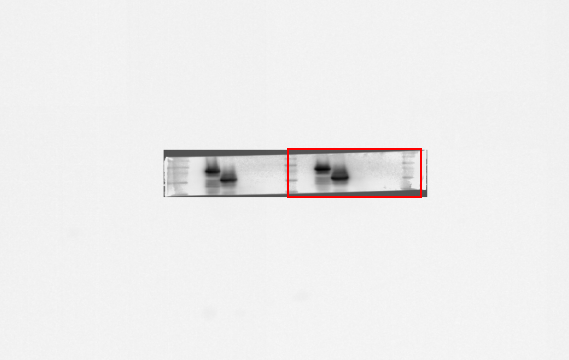

Supplement: Figure 4—figure supplement 2—source data 2. [file elife-102277-fig4-figsupp2-data2.zip › Figure 4—figure supplement 2-source data 2/Figure 4—figure supplement 2E-source data 2/anti-Myc-1_2.tif]

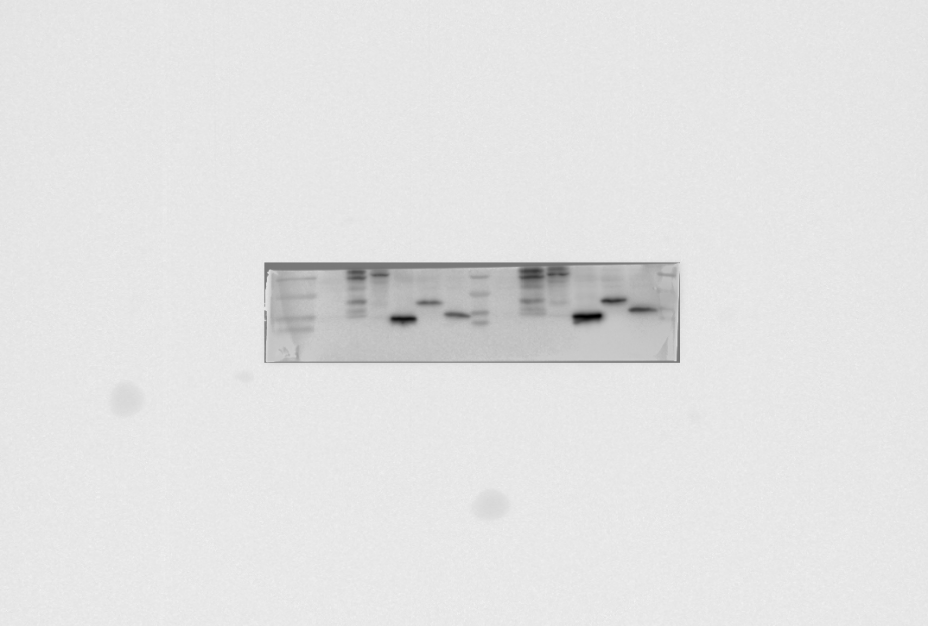

Supplement: Figure 4—figure supplement 2—source data 2. [file elife-102277-fig4-figsupp2-data2.zip › Figure 4—figure supplement 2-source data 2/Figure 4—figure supplement 2E-source data 2/anti-Myc-2.tif]

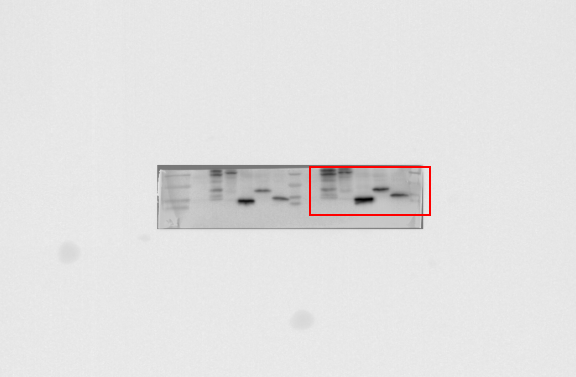

Supplement: Figure 4—figure supplement 2—source data 2. [file elife-102277-fig4-figsupp2-data2.zip › Figure 4—figure supplement 2-source data 2/Figure 4—figure supplement 2E-source data 2/anti-Myc-2_2.tif]

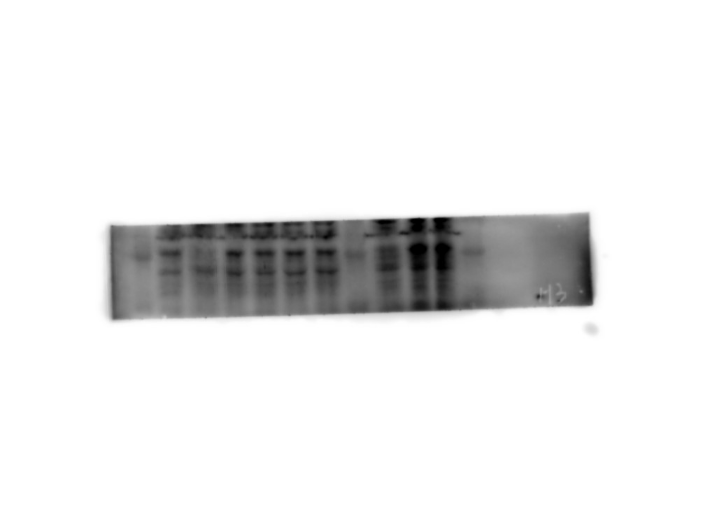

Supplement: Figure 4—figure supplement 2—source data 2. [file elife-102277-fig4-figsupp2-data2.zip › Figure 4—figure supplement 2-source data 2/Figure 4—figure supplement 2E-source data 2/nsp16.tif]

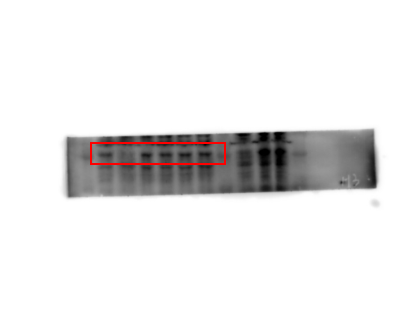

Supplement: Figure 4—figure supplement 2—source data 2. [file elife-102277-fig4-figsupp2-data2.zip › Figure 4—figure supplement 2-source data 2/Figure 4—figure supplement 2E-source data 2/nsp16_2.tif]

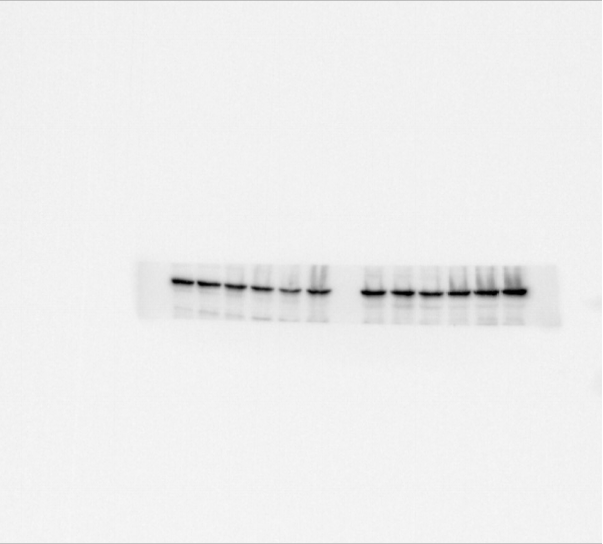

Supplement: Figure 4—figure supplement 2—source data 2. [file elife-102277-fig4-figsupp2-data2.zip › Figure 4—figure supplement 2-source data 2/Figure 4—figure supplement 2E-source data 2/Tubulin.tif]

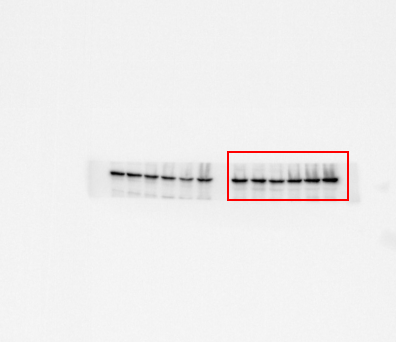

Supplement: Figure 4—figure supplement 2—source data 2. [file elife-102277-fig4-figsupp2-data2.zip › Figure 4—figure supplement 2-source data 2/Figure 4—figure supplement 2E-source data 2/Tubulin_2.tif]

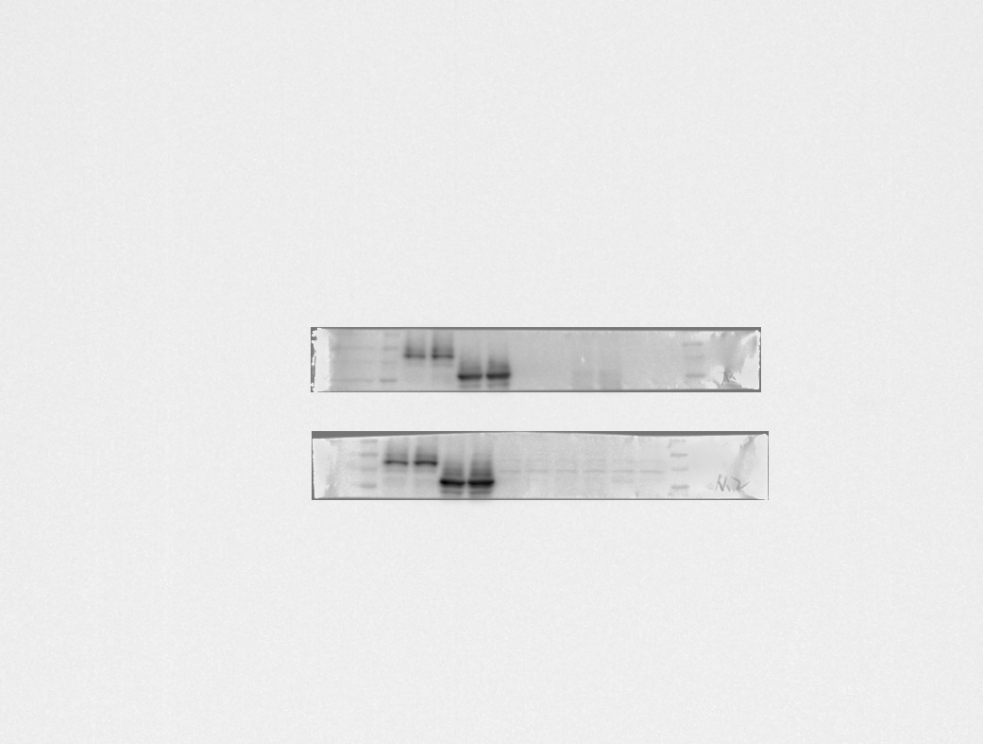

Supplement: Figure 4—figure supplement 2—source data 2. [file elife-102277-fig4-figsupp2-data2.zip › Figure 4—figure supplement 2-source data 2/Figure 4—figure supplement 2F-source data 2/anti-Myc input.tif]

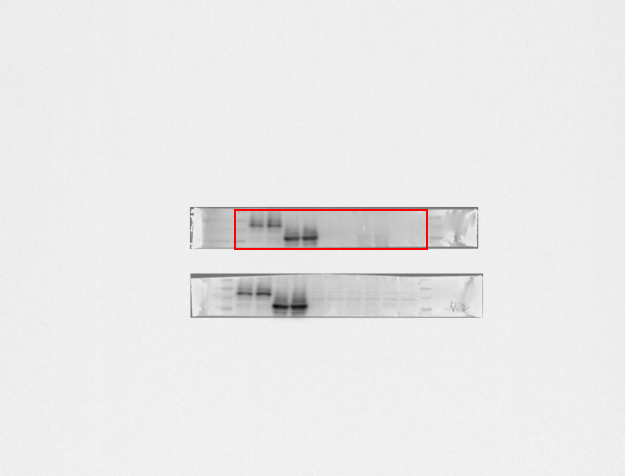

Supplement: Figure 4—figure supplement 2—source data 2. [file elife-102277-fig4-figsupp2-data2.zip › Figure 4—figure supplement 2-source data 2/Figure 4—figure supplement 2F-source data 2/anti-Myc input_2.tif]

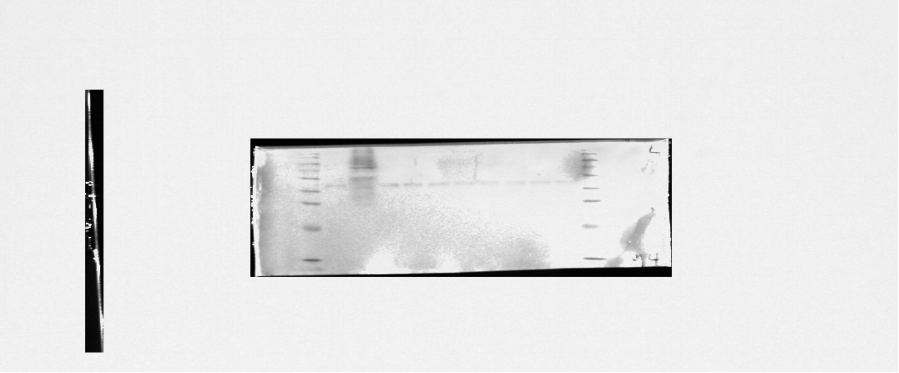

Supplement: Figure 4—figure supplement 2—source data 2. [file elife-102277-fig4-figsupp2-data2.zip › Figure 4—figure supplement 2-source data 2/Figure 4—figure supplement 2F-source data 2/anti-Myc-2 IP.tif]

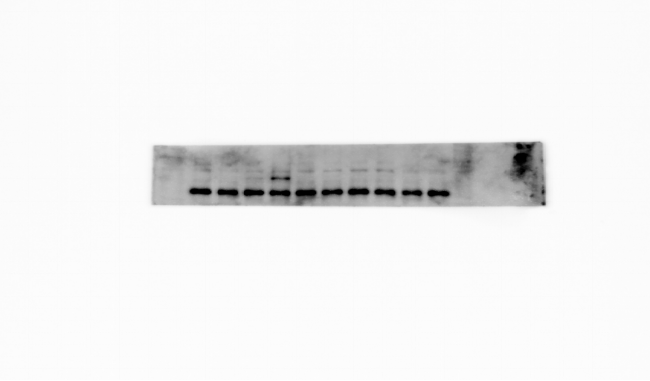

Supplement: Figure 4—figure supplement 2—source data 2. [file elife-102277-fig4-figsupp2-data2.zip › Figure 4—figure supplement 2-source data 2/Figure 4—figure supplement 2F-source data 2/anti-Myc-3 IP.tif]

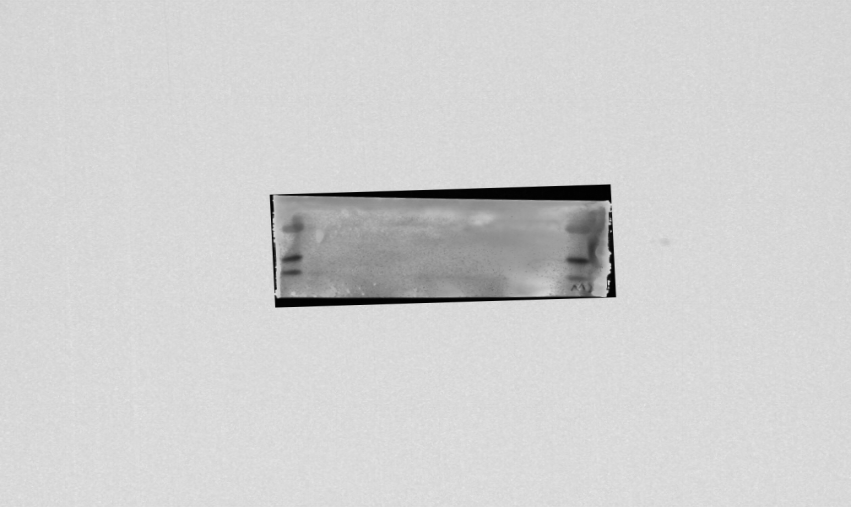

Supplement: Figure 4—figure supplement 2—source data 2. [file elife-102277-fig4-figsupp2-data2.zip › Figure 4—figure supplement 2-source data 2/Figure 4—figure supplement 2F-source data 2/anti-Myc-4 IP.tif]

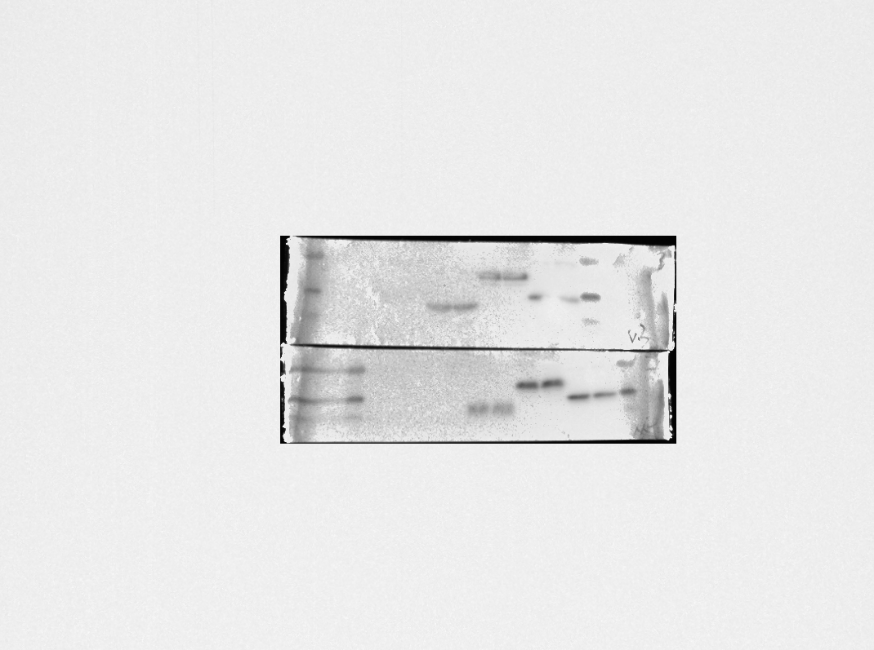

Supplement: Figure 4—figure supplement 2—source data 2. [file elife-102277-fig4-figsupp2-data2.zip › Figure 4—figure supplement 2-source data 2/Figure 4—figure supplement 2F-source data 2/Myc input 2.tif]

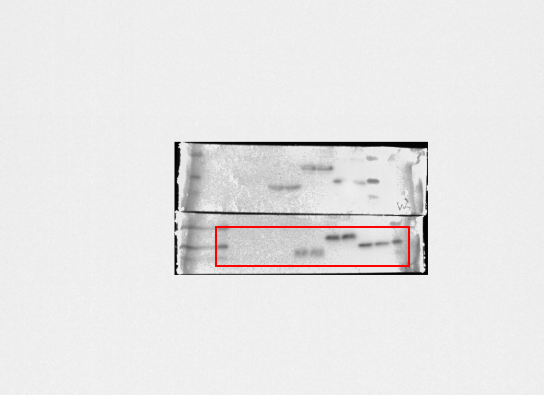

Supplement: Figure 4—figure supplement 2—source data 2. [file elife-102277-fig4-figsupp2-data2.zip › Figure 4—figure supplement 2-source data 2/Figure 4—figure supplement 2F-source data 2/Myc input 2_2.tif]

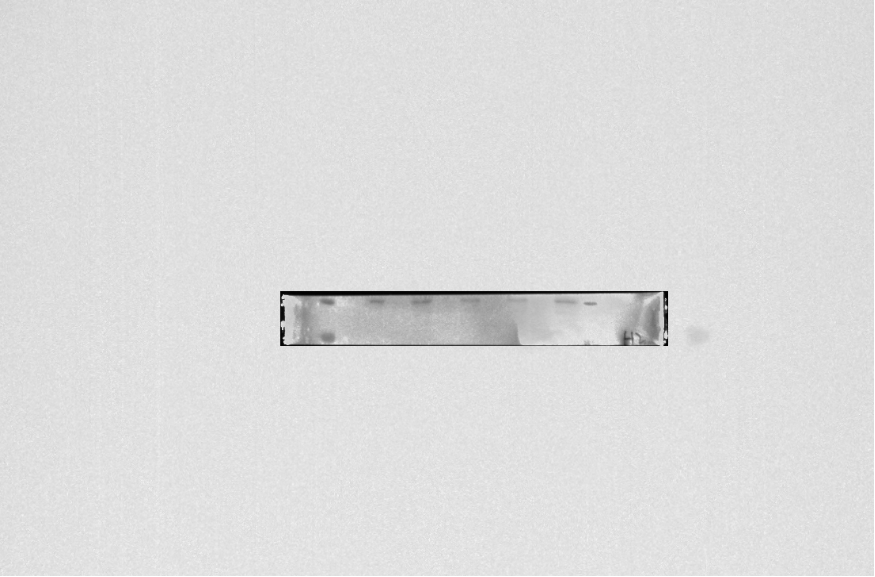

Supplement: Figure 4—figure supplement 2—source data 2. [file elife-102277-fig4-figsupp2-data2.zip › Figure 4—figure supplement 2-source data 2/Figure 4—figure supplement 2F-source data 2/nsp16-input.tif]

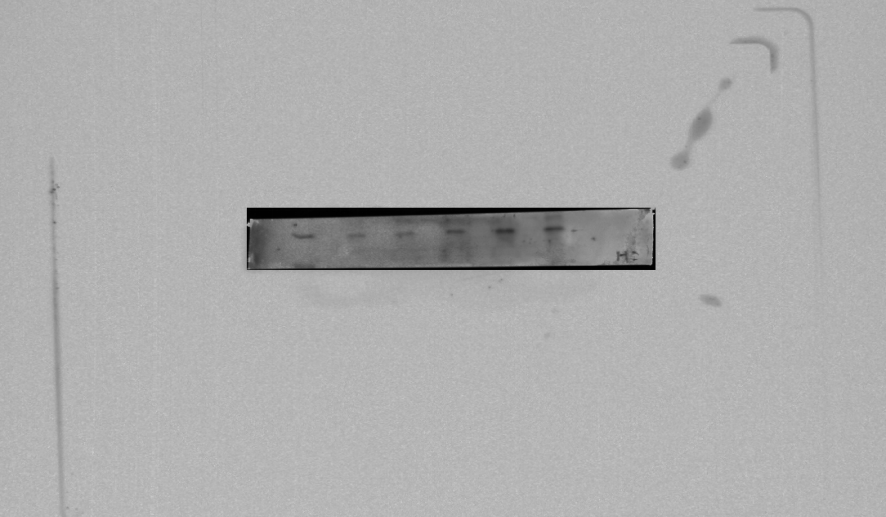

Supplement: Figure 4—figure supplement 2—source data 2. [file elife-102277-fig4-figsupp2-data2.zip › Figure 4—figure supplement 2-source data 2/Figure 4—figure supplement 2F-source data 2/nsp16-IP.tif]

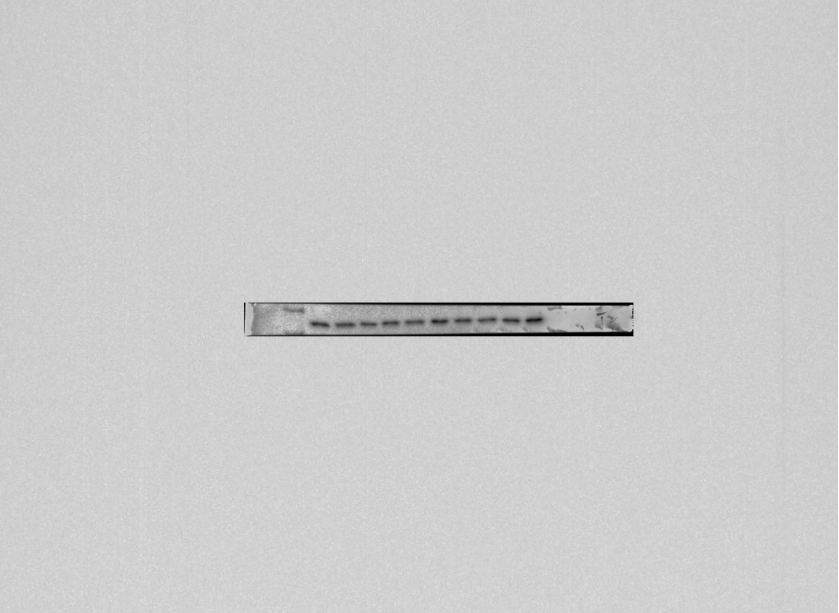

Supplement: Figure 4—figure supplement 2—source data 2. [file elife-102277-fig4-figsupp2-data2.zip › Figure 4—figure supplement 2-source data 2/Figure 4—figure supplement 2F-source data 2/Tubulin input.tif]

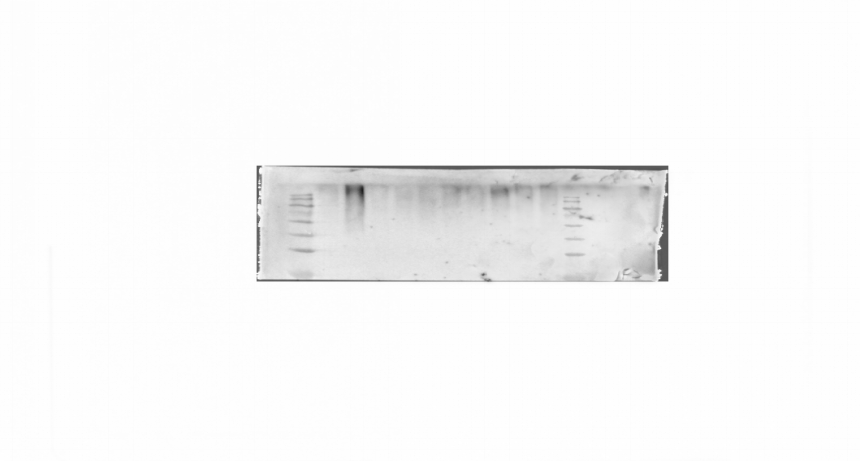

Supplement: Figure 4—figure supplement 2—source data 2. [file elife-102277-fig4-figsupp2-data2.zip › Figure 4—figure supplement 2-source data 2/Figure 4—figure supplement 2F-source data 2/Ub IP.tif]

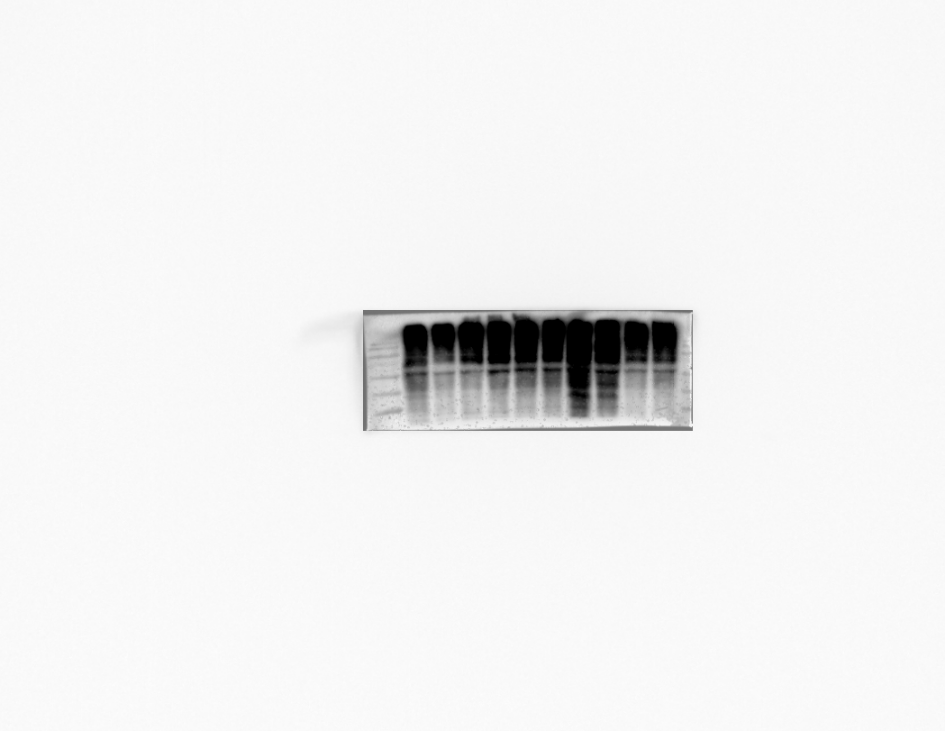

Supplement: Figure 4—figure supplement 2—source data 2. [file elife-102277-fig4-figsupp2-data2.zip › Figure 4—figure supplement 2-source data 2/Figure 4—figure supplement 2F-source data 2/Ub-input.tif]

Figure 5A

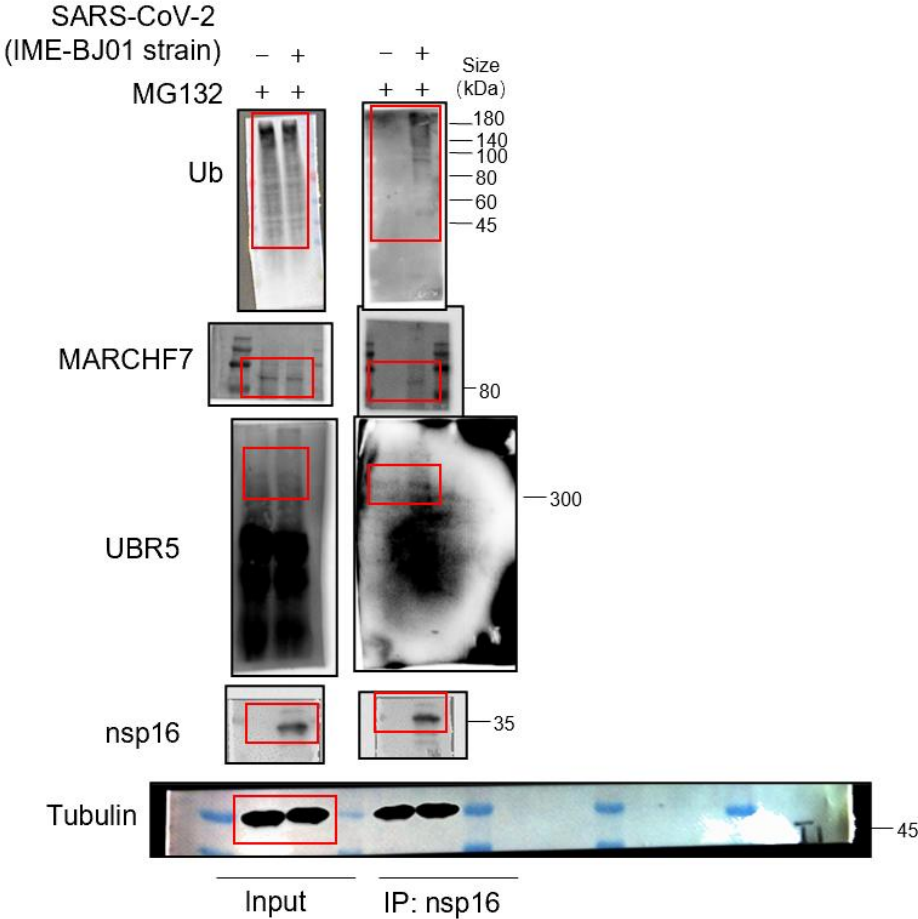

Supplement: Figure 5—source data 1. [file elife-102277-fig5-data1.zip › Figure 5-source data 1/Figure 5A-source data 1 .pdf]

Figure 5I

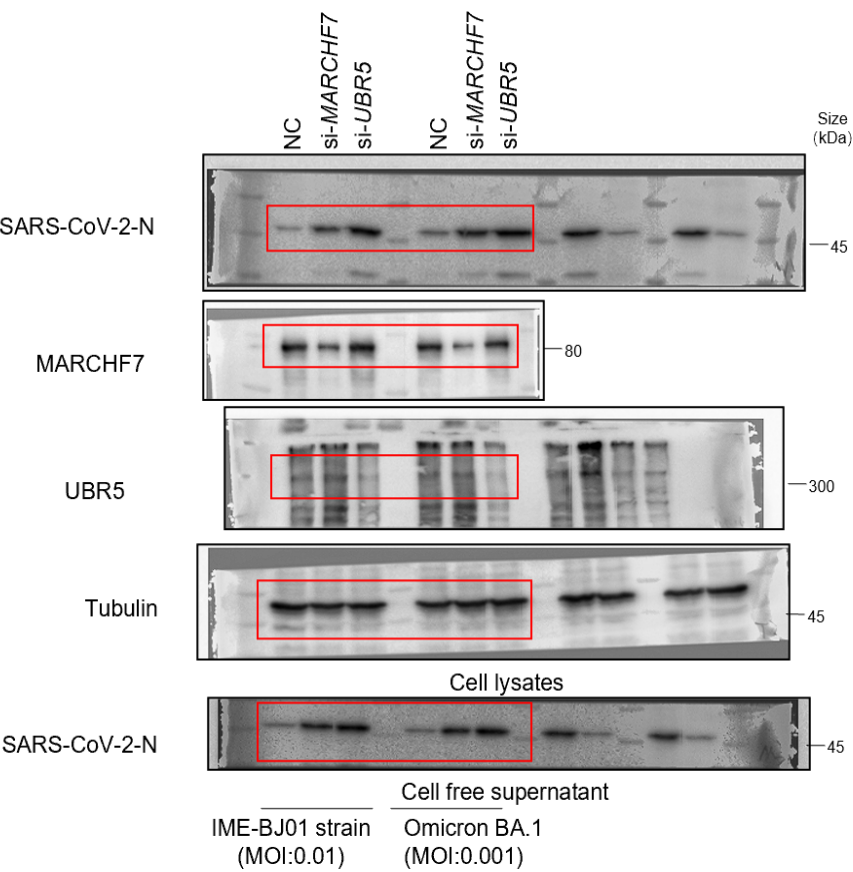

Supplement: Figure 5—source data 1. [file elife-102277-fig5-data1.zip › Figure 5-source data 1/Figure 5I-source data 1.pdf]

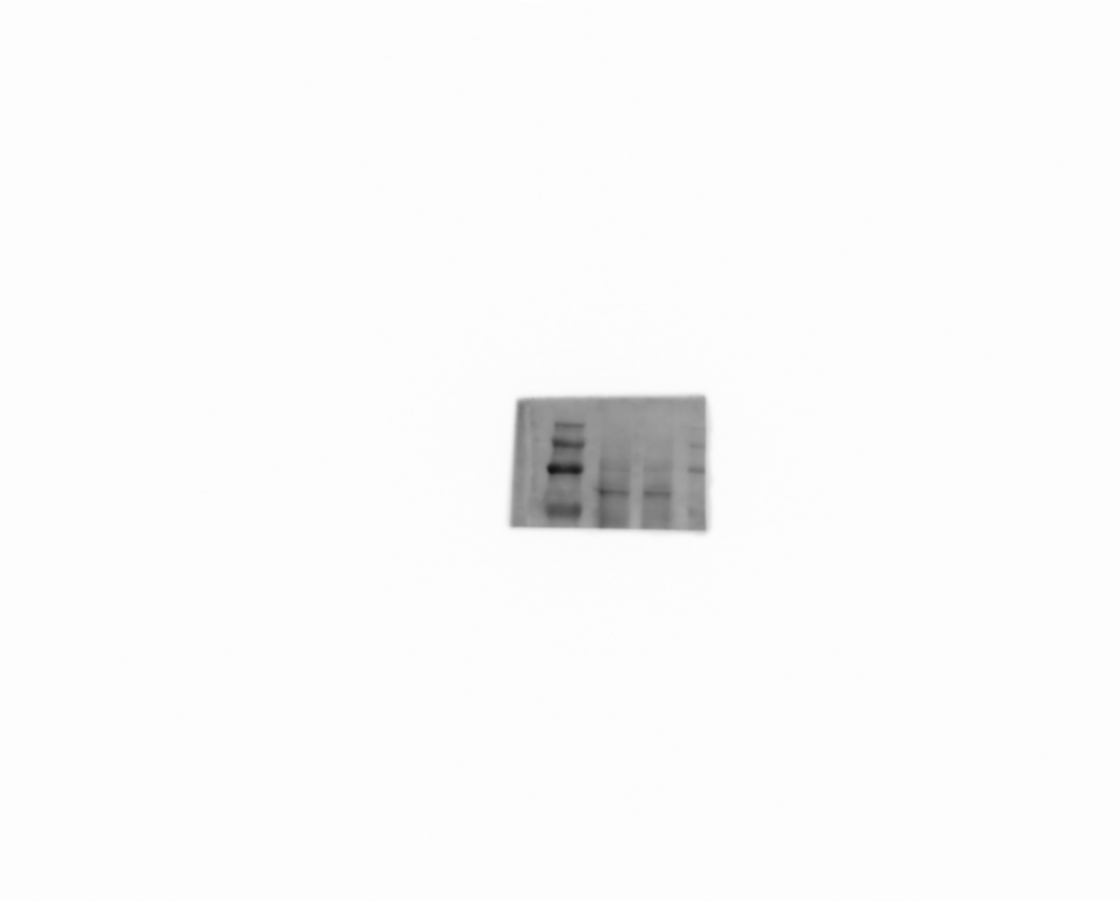

Supplement: Figure 5—source data 2. [file elife-102277-fig5-data2.zip › Figure 5-source data 2/Figure 5A-source data 2/MARCHF7-input.tif]

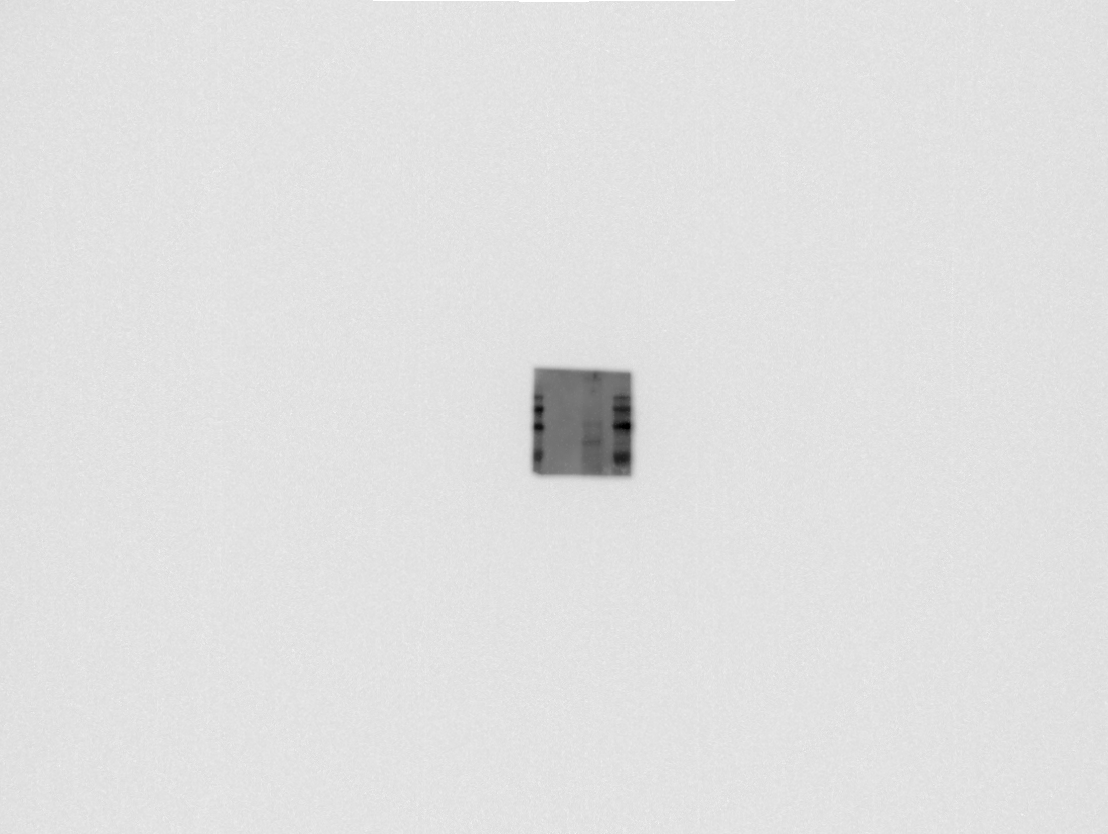

Supplement: Figure 5—source data 2. [file elife-102277-fig5-data2.zip › Figure 5-source data 2/Figure 5A-source data 2/MARCHF7-IP.tif]

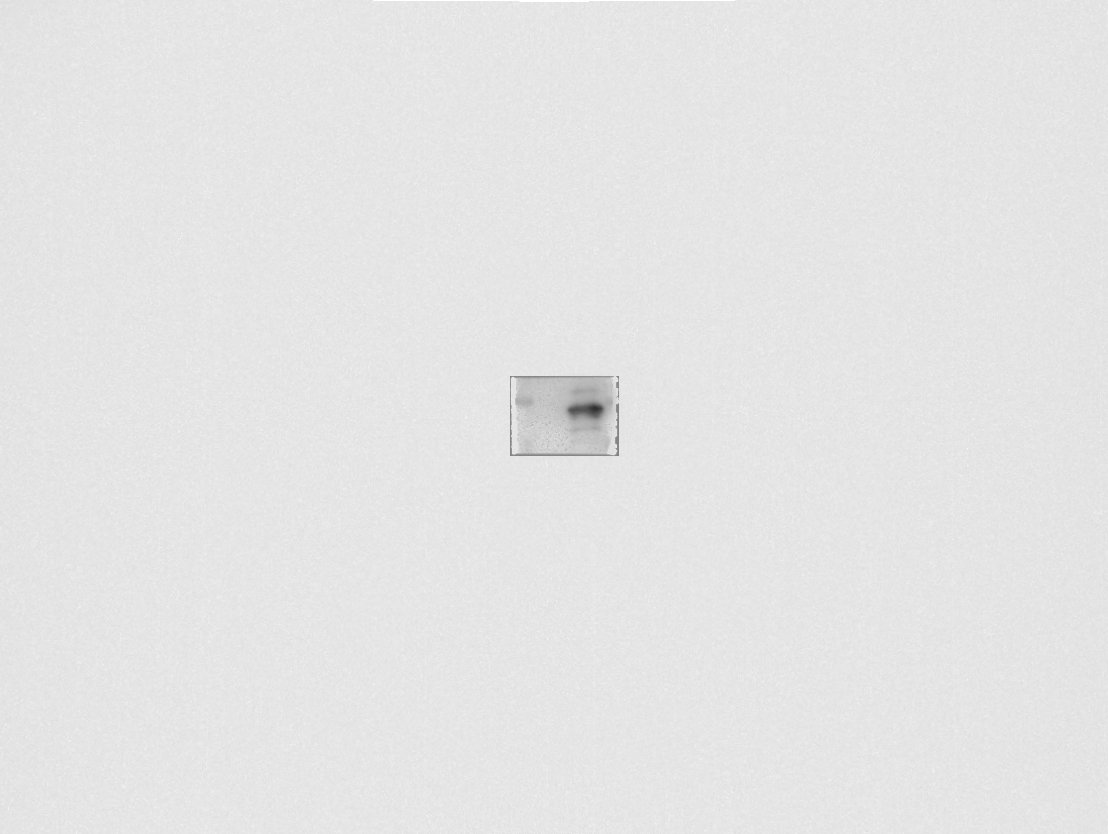

Supplement: Figure 5—source data 2. [file elife-102277-fig5-data2.zip › Figure 5-source data 2/Figure 5A-source data 2/nsp16-input.tif]

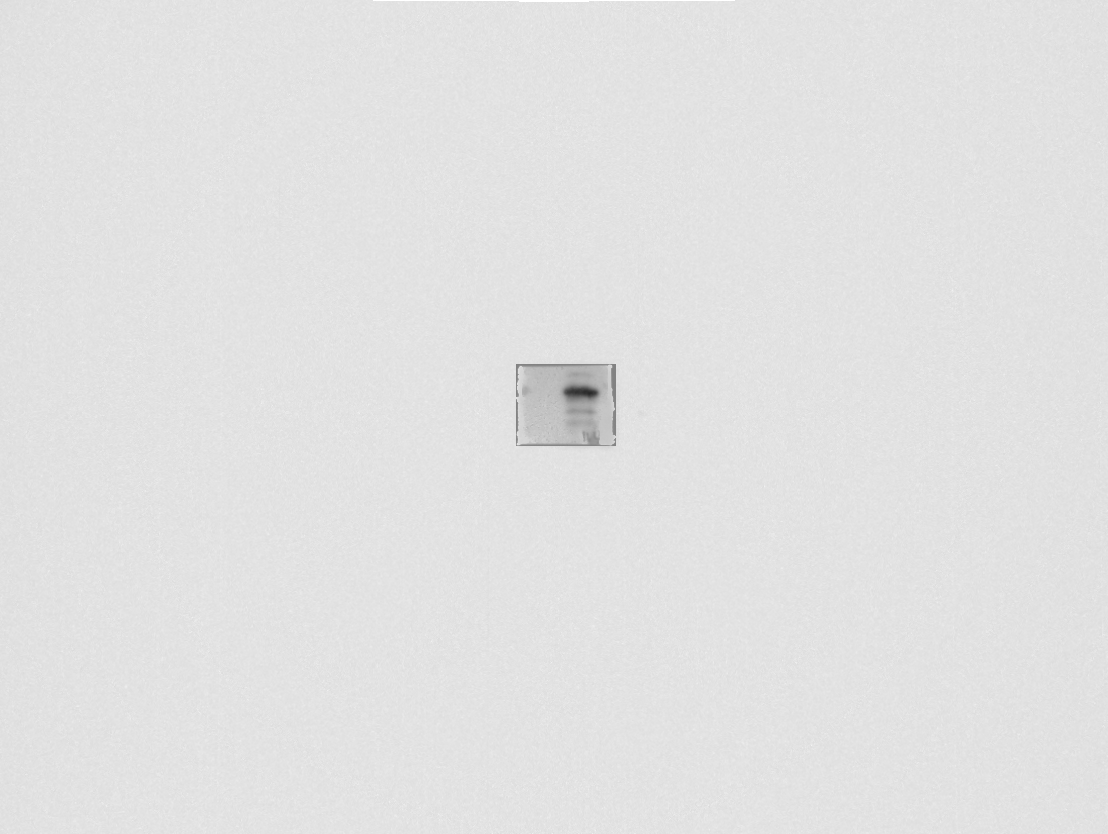

Supplement: Figure 5—source data 2. [file elife-102277-fig5-data2.zip › Figure 5-source data 2/Figure 5A-source data 2/nsp16-IP.tif]

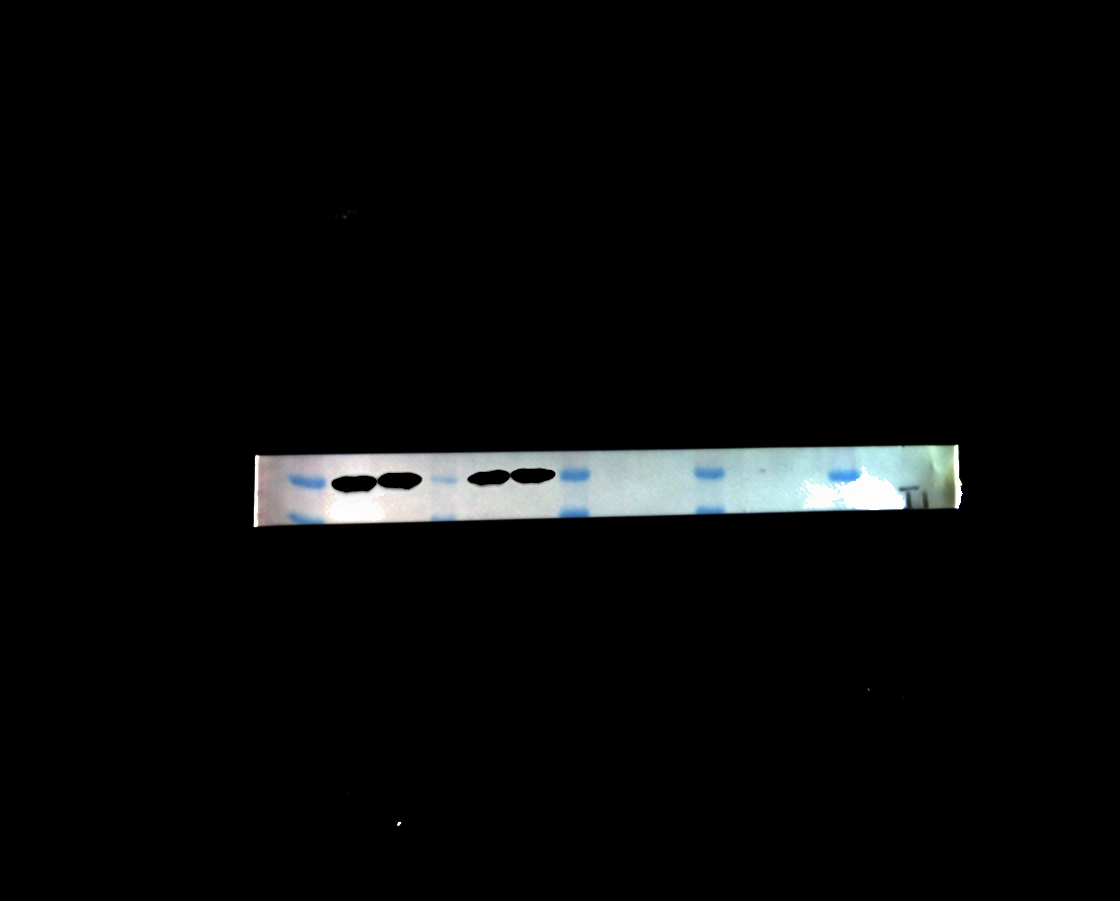

Supplement: Figure 5—source data 2. [file elife-102277-fig5-data2.zip › Figure 5-source data 2/Figure 5A-source data 2/Tubulin-input.tif]

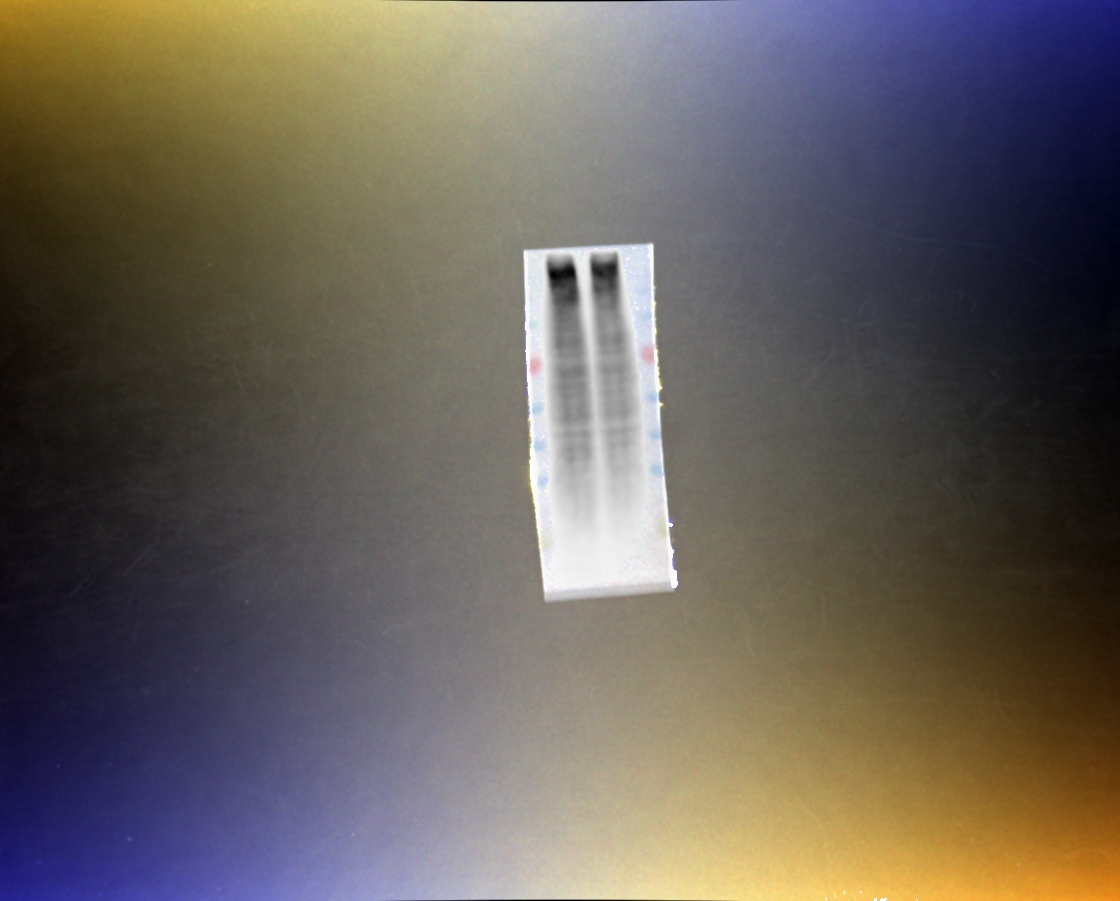

Supplement: Figure 5—source data 2. [file elife-102277-fig5-data2.zip › Figure 5-source data 2/Figure 5A-source data 2/Ub-input.tif]

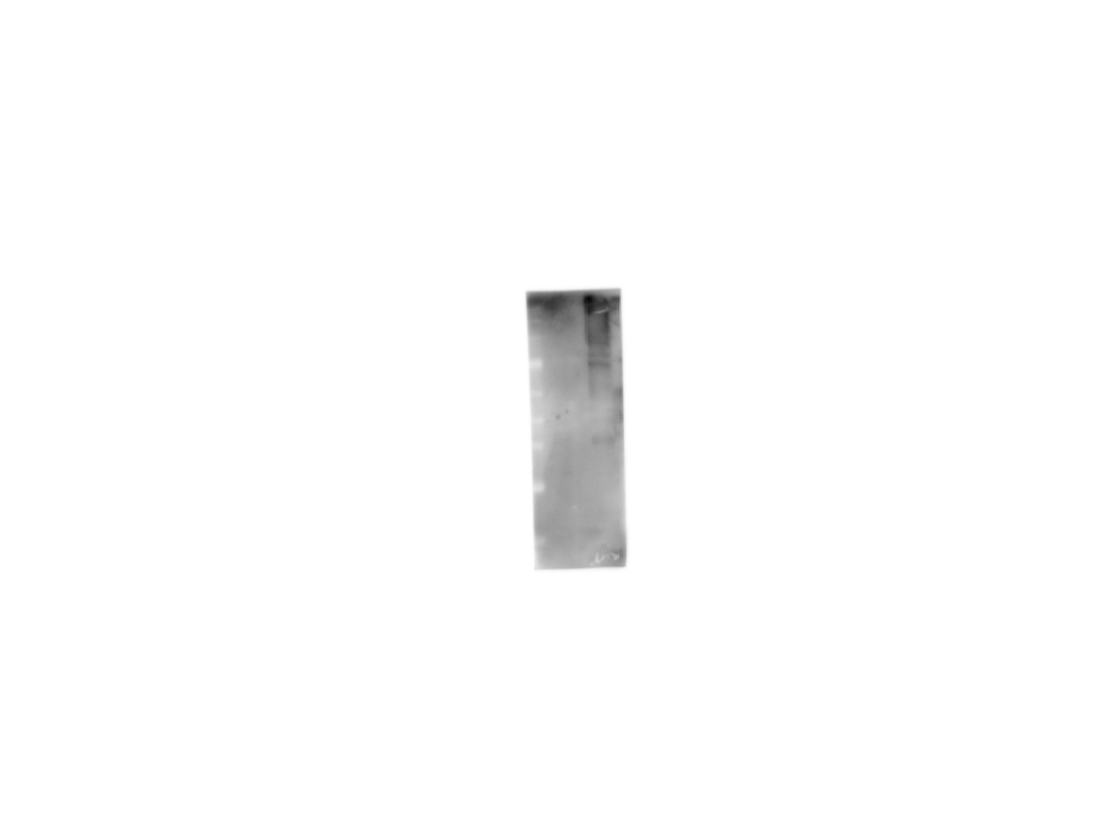

Supplement: Figure 5—source data 2. [file elife-102277-fig5-data2.zip › Figure 5-source data 2/Figure 5A-source data 2/Ub-IP.tif]

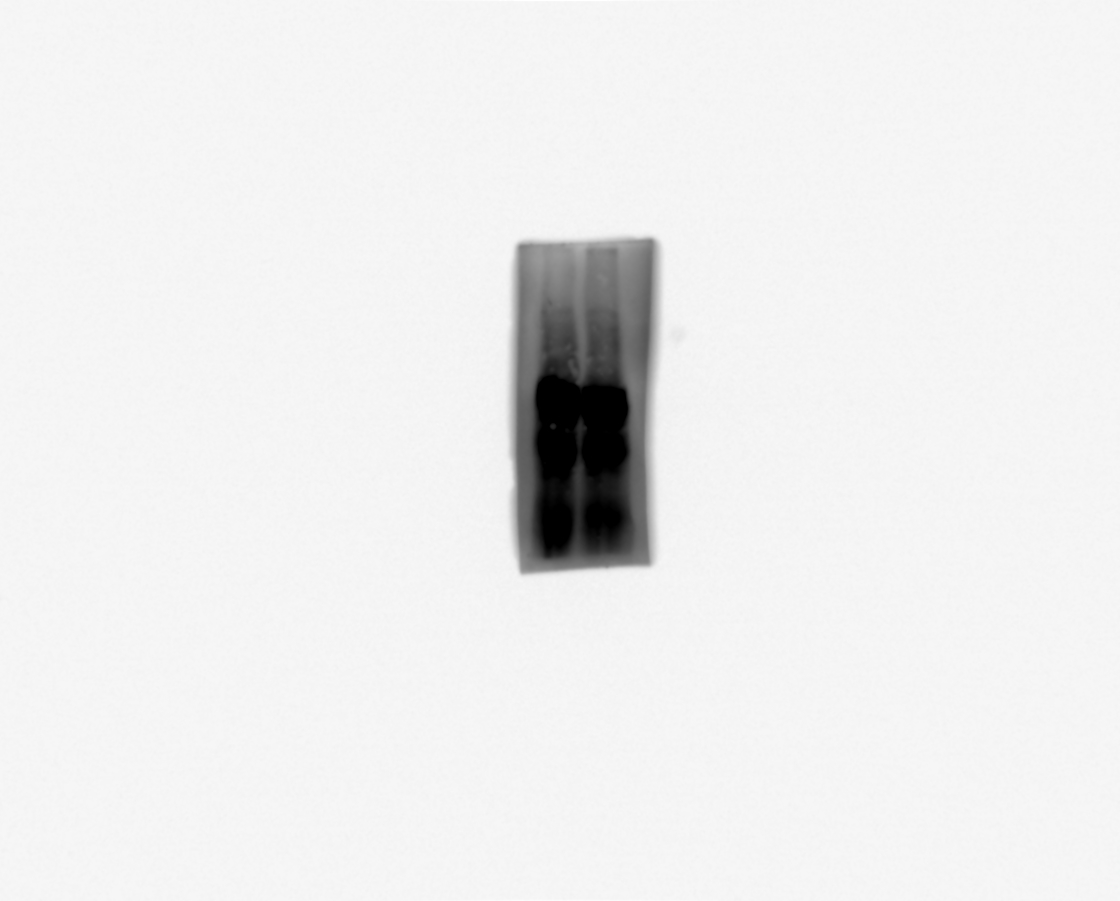

Supplement: Figure 5—source data 2. [file elife-102277-fig5-data2.zip › Figure 5-source data 2/Figure 5A-source data 2/UBR5-input.tif]

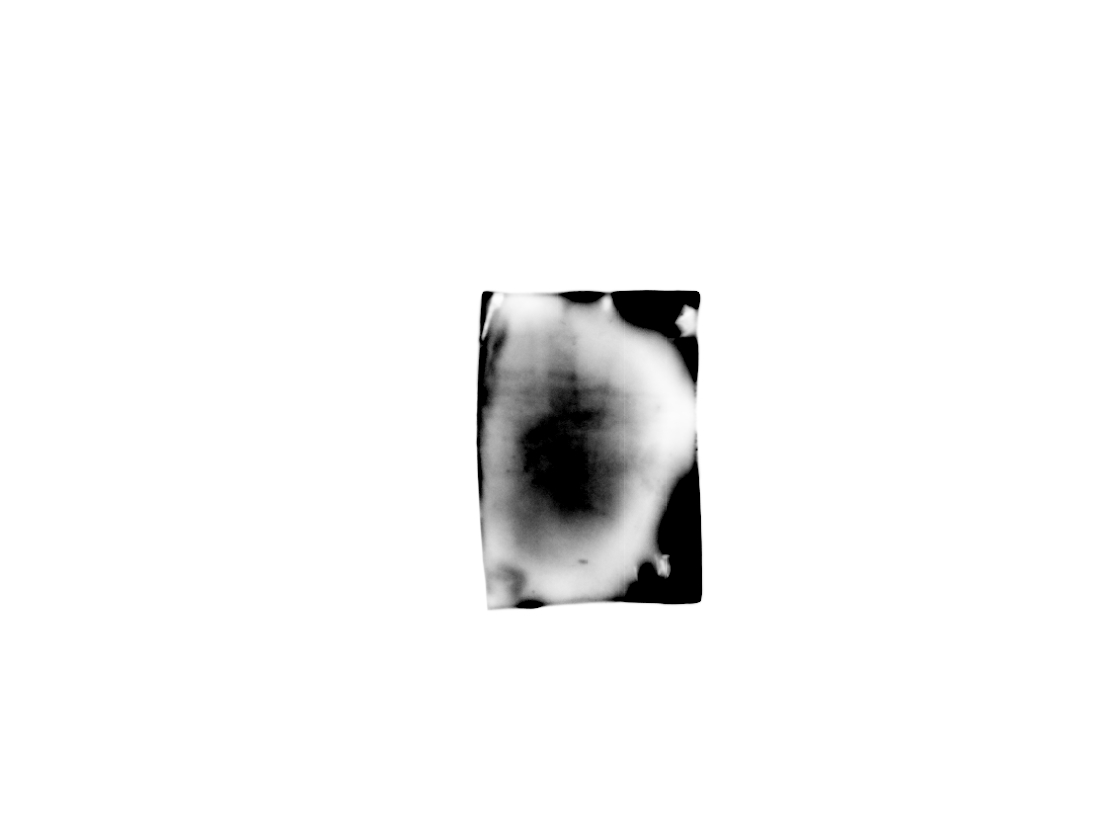

Supplement: Figure 5—source data 2. [file elife-102277-fig5-data2.zip › Figure 5-source data 2/Figure 5A-source data 2/UBR5-IP.tif]

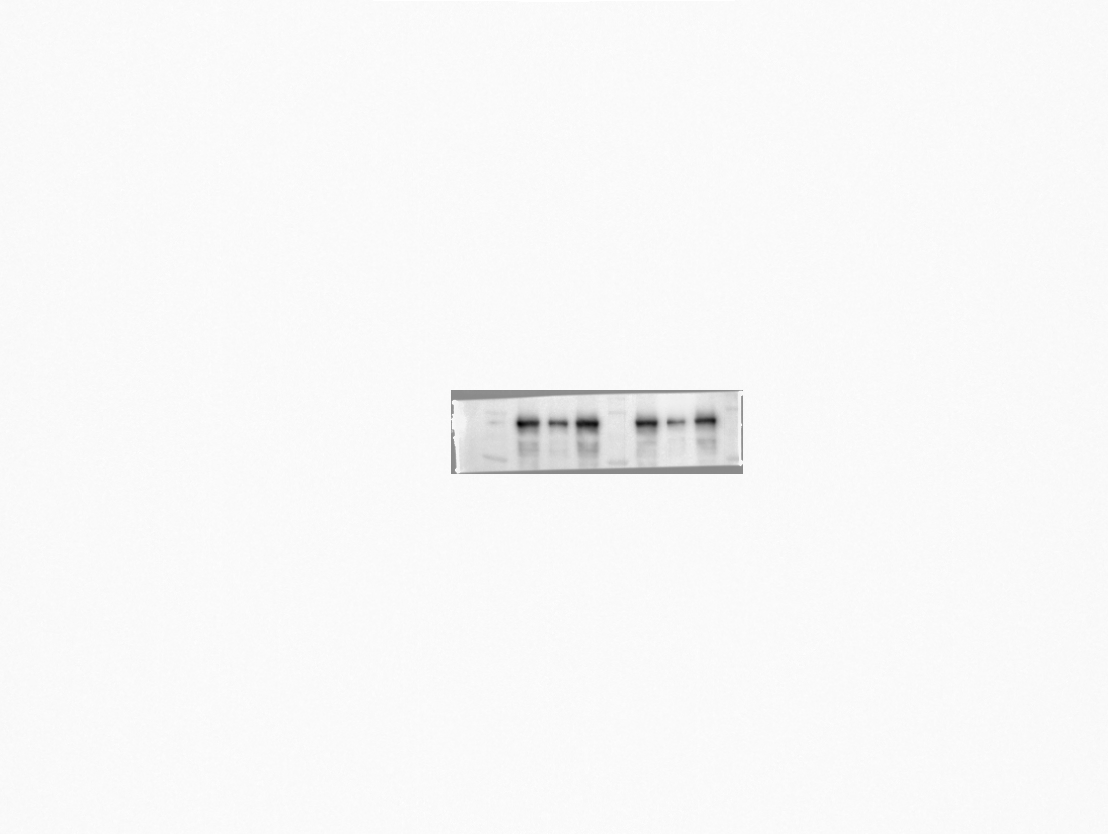

Supplement: Figure 5—source data 2. [file elife-102277-fig5-data2.zip › Figure 5-source data 2/Figure 5I-source data 2/MARCHF7.tif]

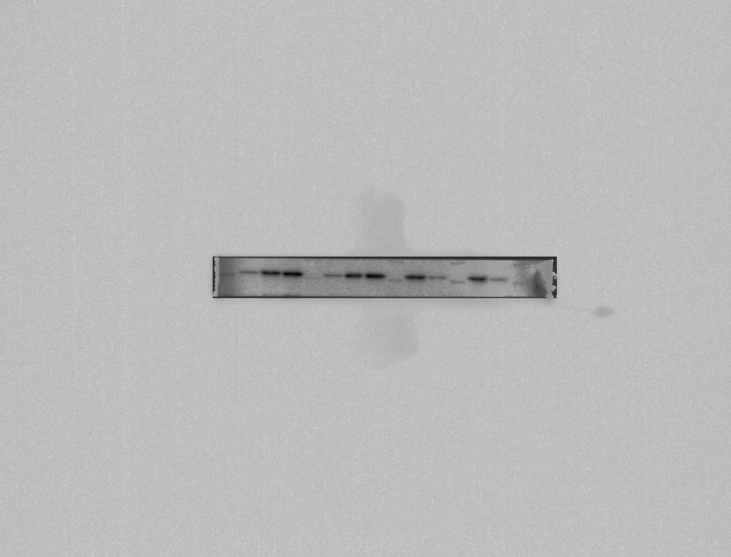

Supplement: Figure 5—source data 2. [file elife-102277-fig5-data2.zip › Figure 5-source data 2/Figure 5I-source data 2/SARS-CoV-2-N in supernatant.tif]

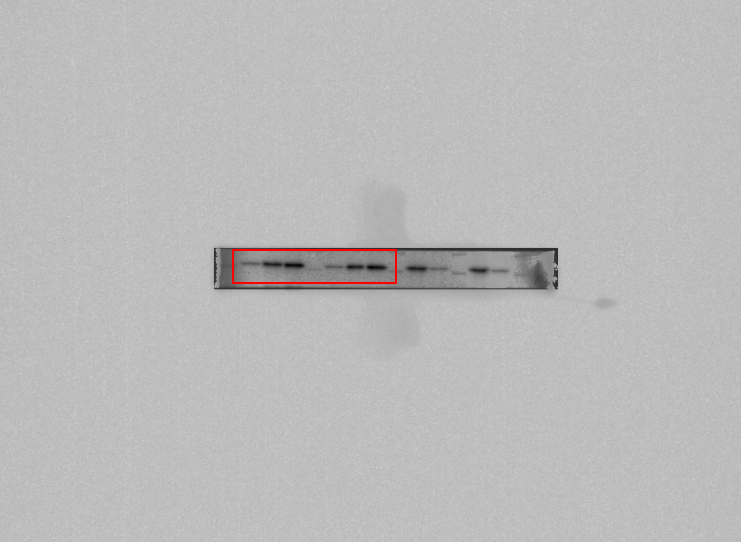

Supplement: Figure 5—source data 2. [file elife-102277-fig5-data2.zip › Figure 5-source data 2/Figure 5I-source data 2/SARS-CoV-2-N in supernatant_2.tif]

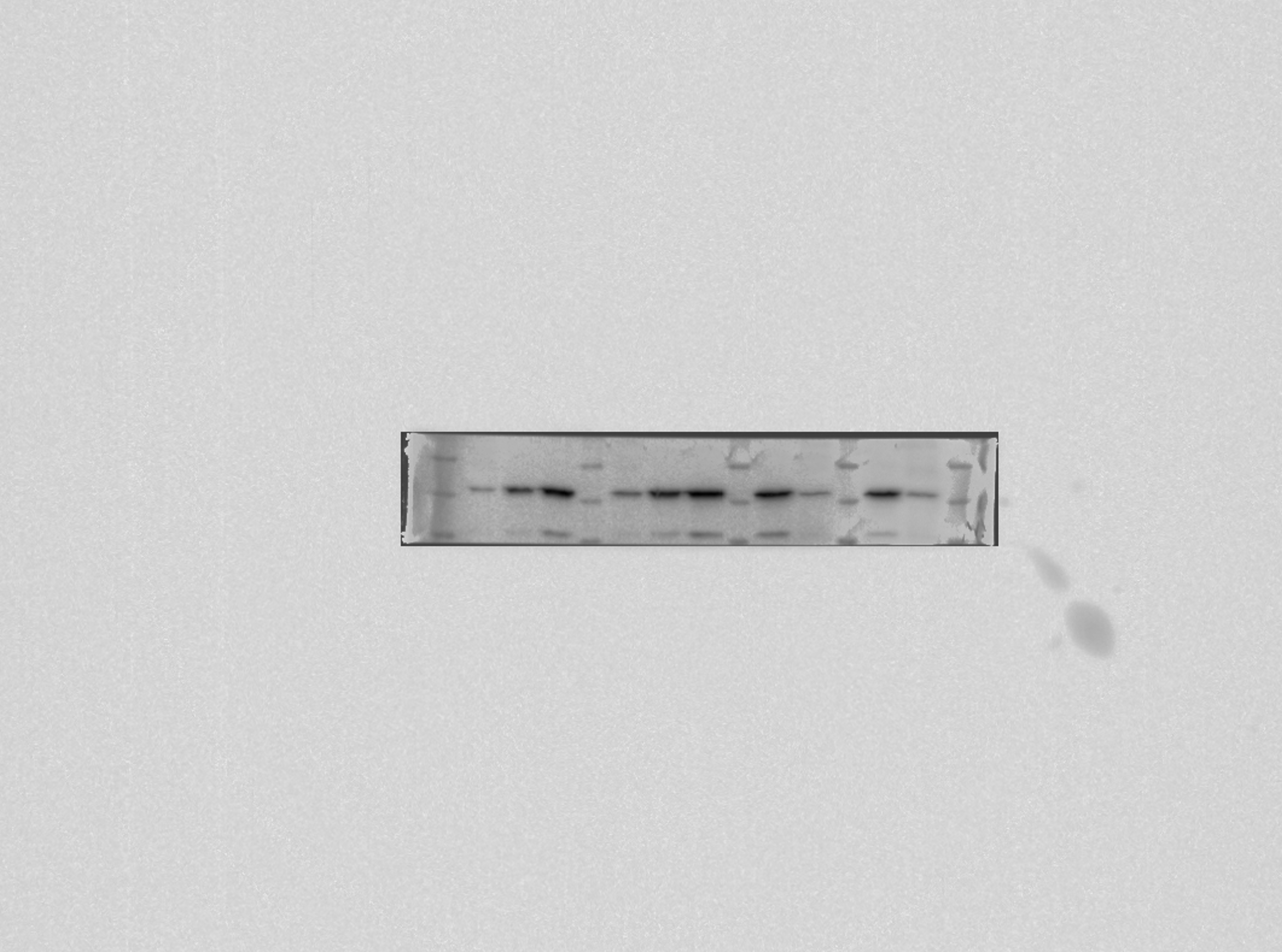

Supplement: Figure 5—source data 2. [file elife-102277-fig5-data2.zip › Figure 5-source data 2/Figure 5I-source data 2/SARS-CoV-2-N.tif]

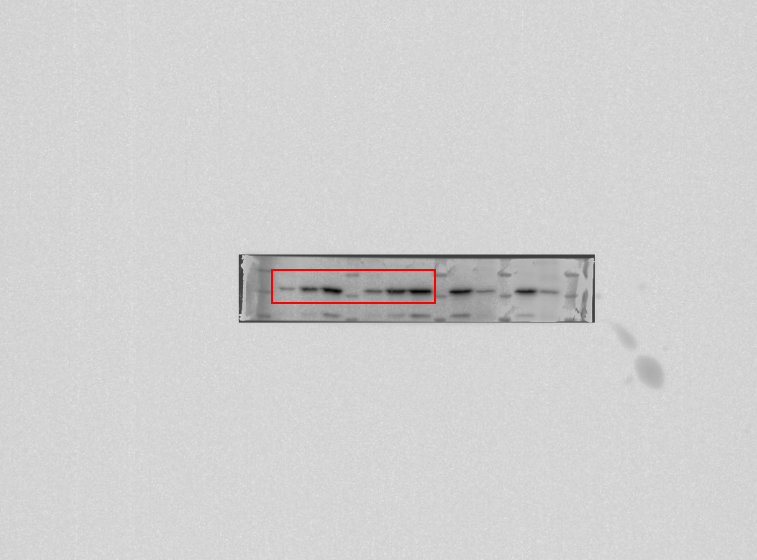

Supplement: Figure 5—source data 2. [file elife-102277-fig5-data2.zip › Figure 5-source data 2/Figure 5I-source data 2/SARS-CoV-2-N_2.tif]

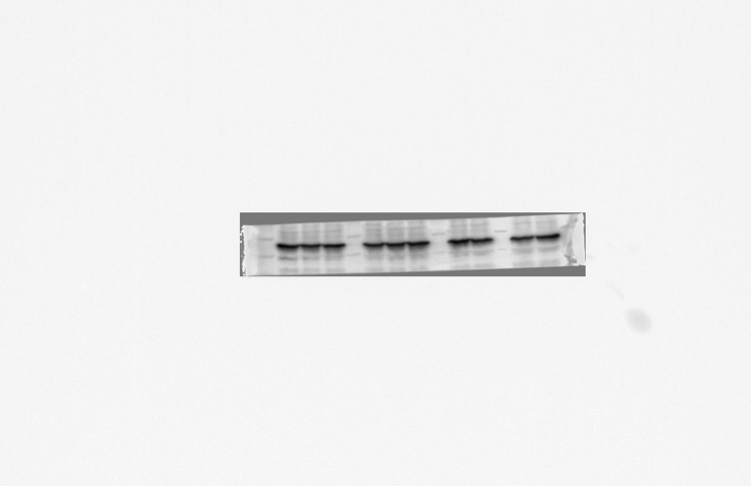

Supplement: Figure 5—source data 2. [file elife-102277-fig5-data2.zip › Figure 5-source data 2/Figure 5I-source data 2/Tubulin.tif]

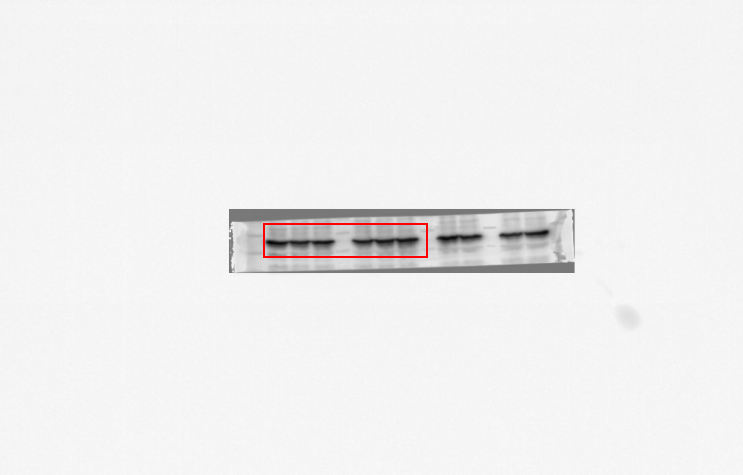

Supplement: Figure 5—source data 2. [file elife-102277-fig5-data2.zip › Figure 5-source data 2/Figure 5I-source data 2/Tubulin_2.tif]

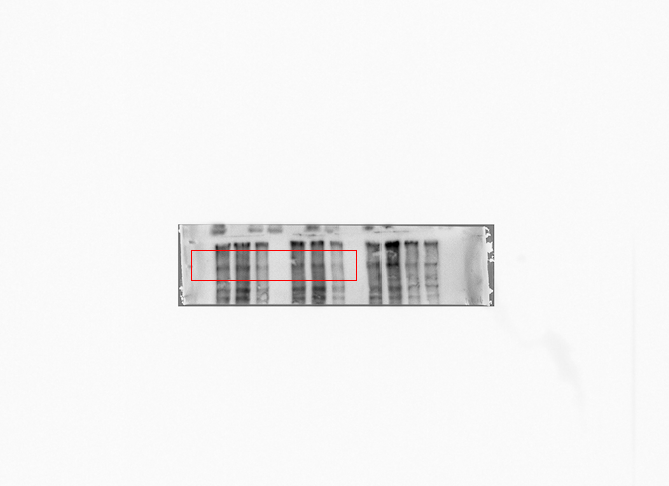

Supplement: Figure 5—source data 2. [file elife-102277-fig5-data2.zip › Figure 5-source data 2/Figure 5I-source data 2/UBR5-2.tif]

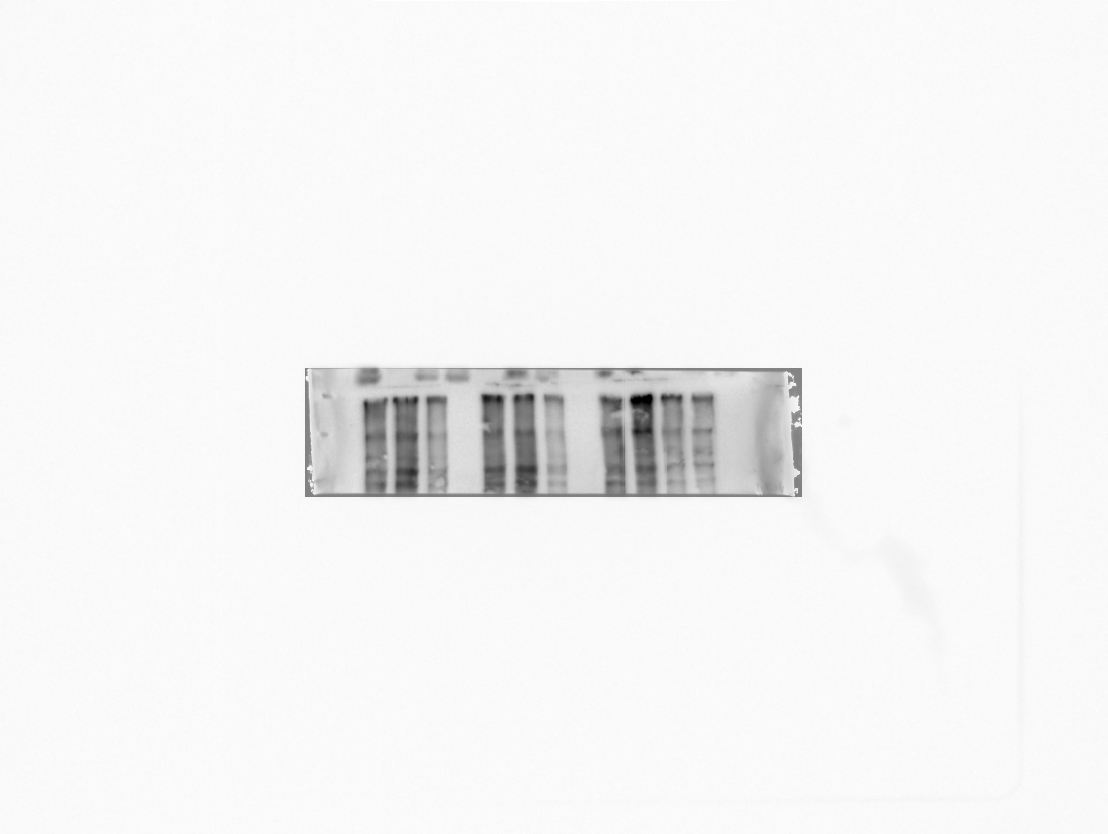

Supplement: Figure 5—source data 2. [file elife-102277-fig5-data2.zip › Figure 5-source data 2/Figure 5I-source data 2/UBR5.tif]

Figure 6G-H

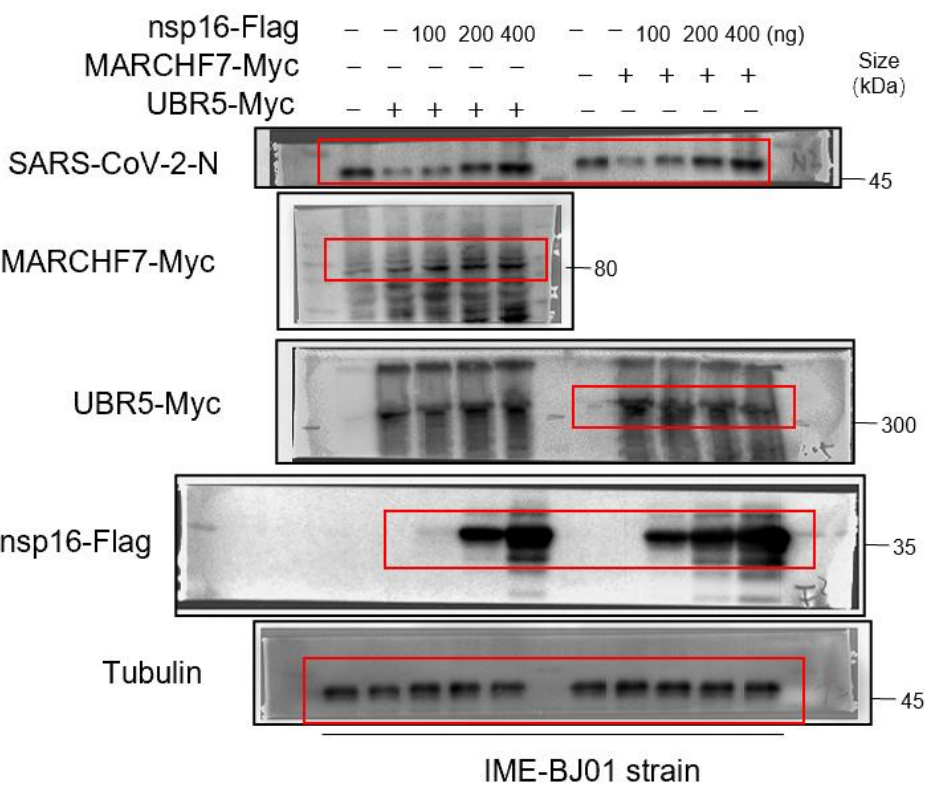

Supplement: Figure 6—source data 1. [file elife-102277-fig6-data1.zip › Figure 6-source data 1/Figure 6G-H-source data 1.pdf]

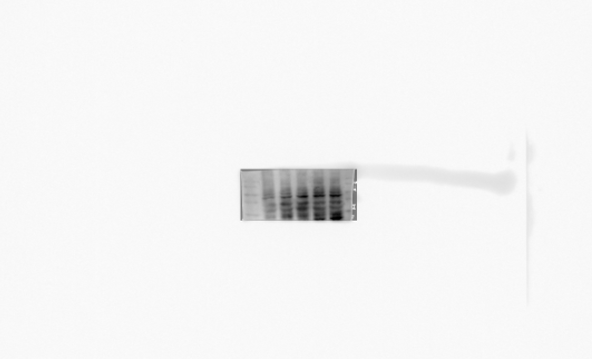

Supplement: Figure 6—source data 2. [file elife-102277-fig6-data2.zip › Figure 6-source data 2/Figure 6G-H-source data 2/MARCHF7.tif]

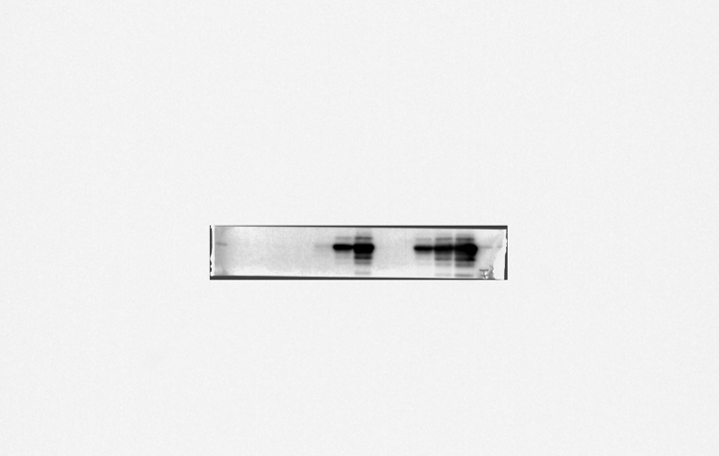

Supplement: Figure 6—source data 2. [file elife-102277-fig6-data2.zip › Figure 6-source data 2/Figure 6G-H-source data 2/nsp16.tif]

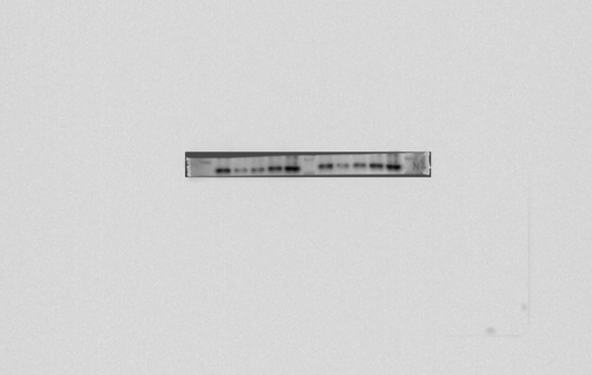

Supplement: Figure 6—source data 2. [file elife-102277-fig6-data2.zip › Figure 6-source data 2/Figure 6G-H-source data 2/SARS-CoV-2-N.tif]

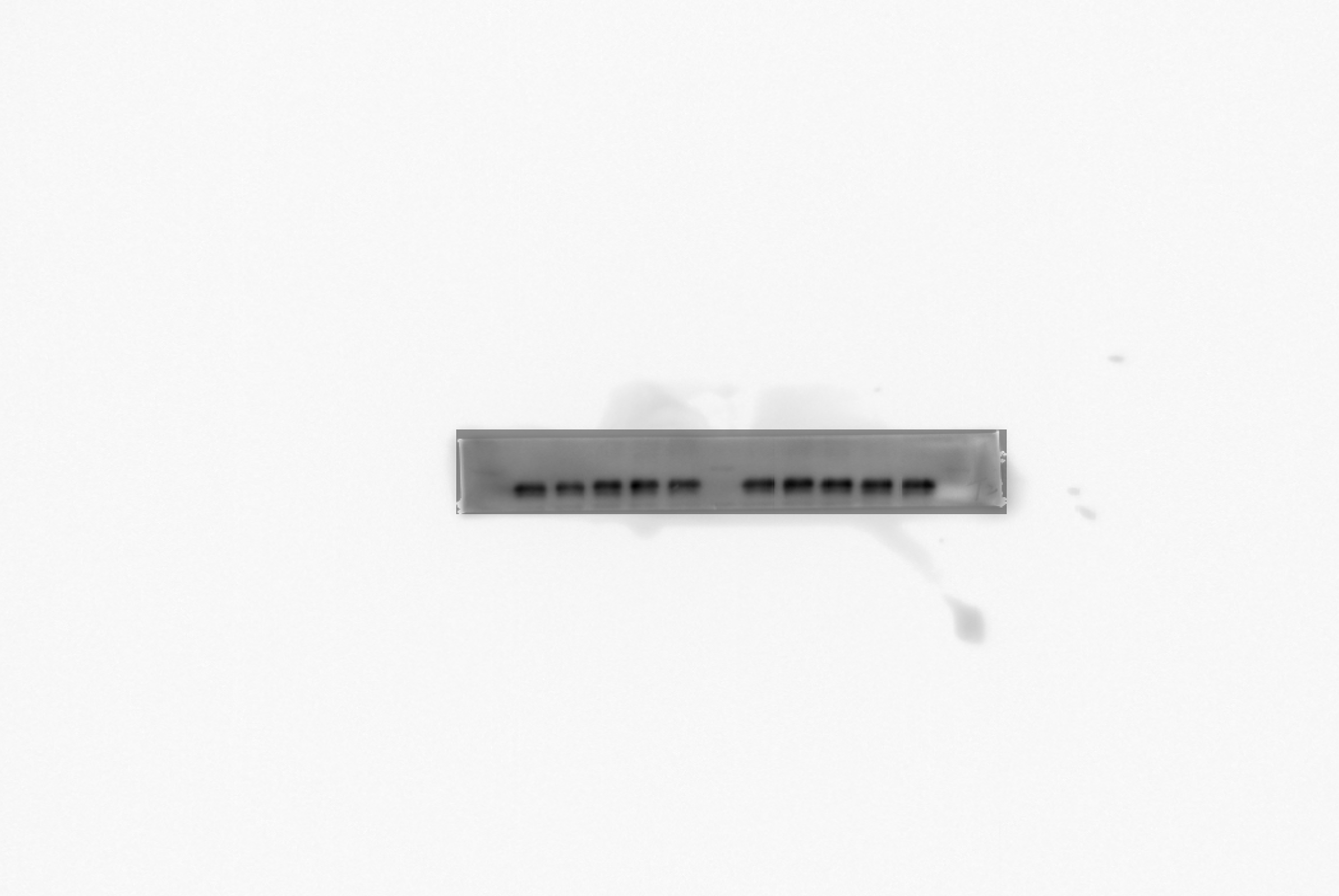

Supplement: Figure 6—source data 2. [file elife-102277-fig6-data2.zip › Figure 6-source data 2/Figure 6G-H-source data 2/Tubulin.tif]

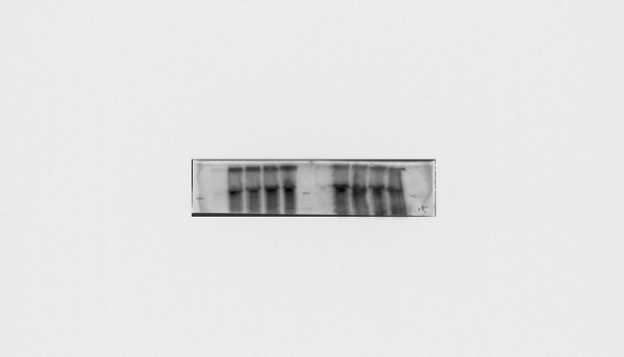

Supplement: Figure 6—source data 2. [file elife-102277-fig6-data2.zip › Figure 6-source data 2/Figure 6G-H-source data 2/UBR5.tif]

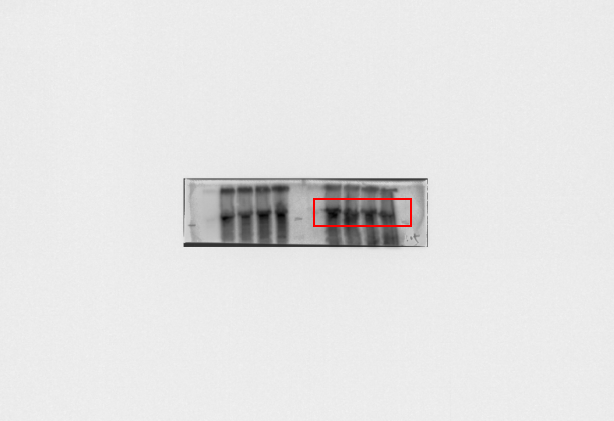

Supplement: Figure 6—source data 2. [file elife-102277-fig6-data2.zip › Figure 6-source data 2/Figure 6G-H-source data 2/UBR5_2.tif]

Figure 6—figure supplement 1G-H

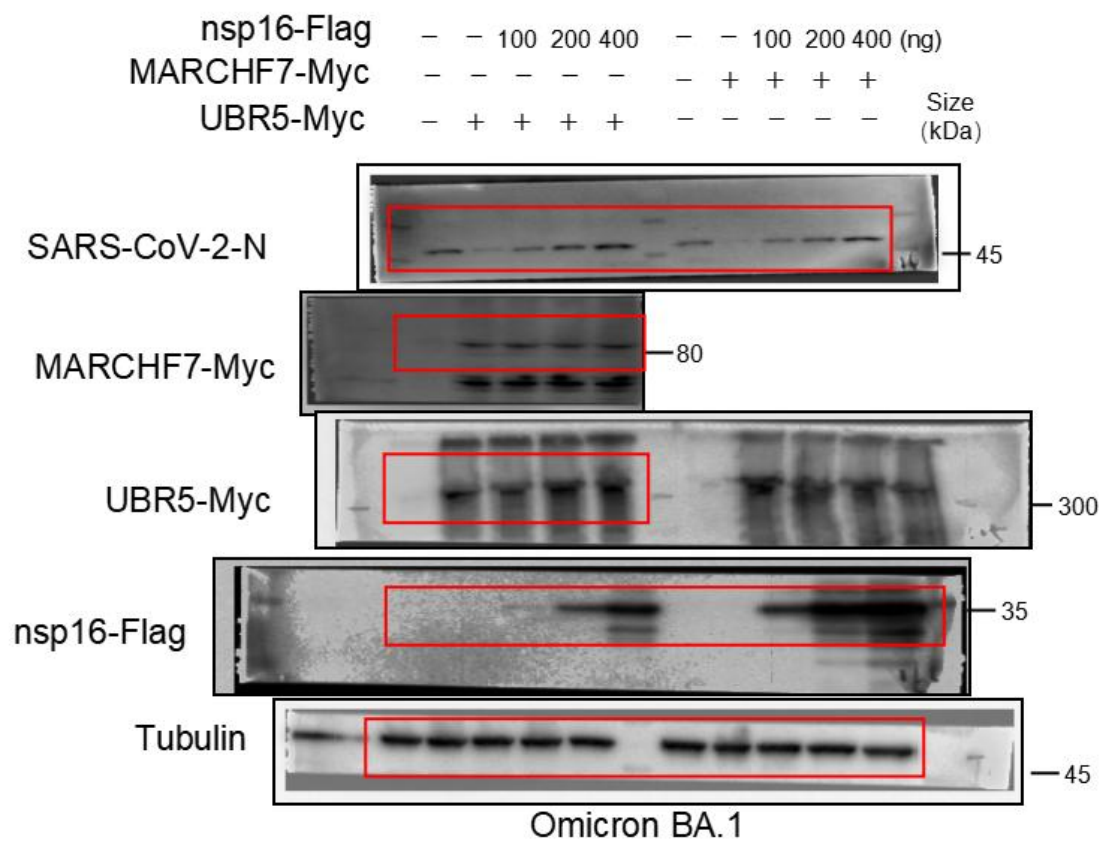

Supplement: Figure 6—figure supplement 1—source data 1. [file elife-102277-fig6-figsupp1-data1.zip › Figure 6—figure supplement 1-source data 1/Figure 6—figure supplement 1G-H-source data 1.pdf]

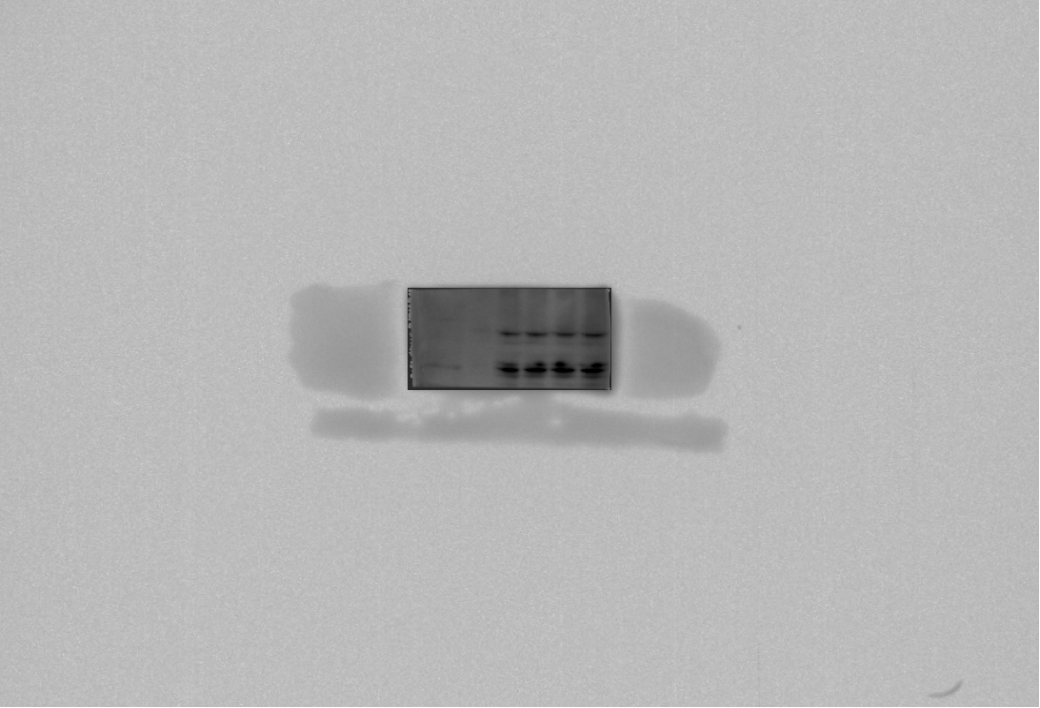

Supplement: Figure 6—figure supplement 1—source data 2. [file elife-102277-fig6-figsupp1-data2.zip › Figure 6—figure supplement 1-source data 2/Figure 6—figure supplement 1G-H-source data 2/MARCHF7-MYC.tif]

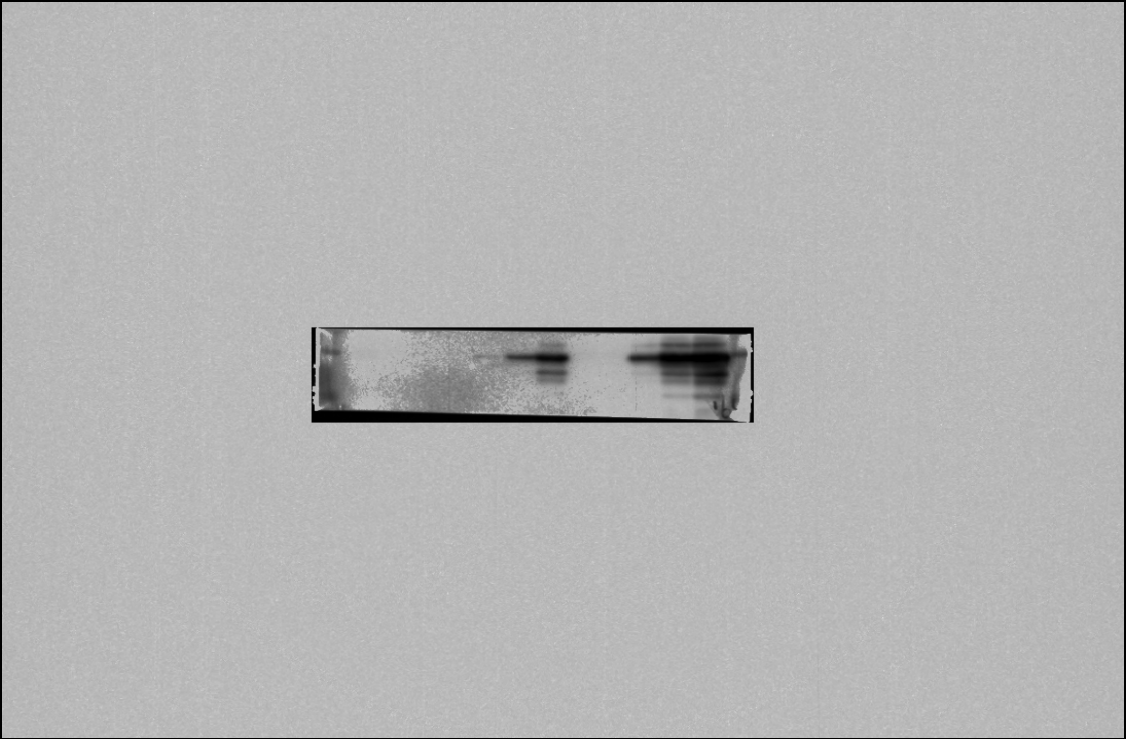

Supplement: Figure 6—figure supplement 1—source data 2. [file elife-102277-fig6-figsupp1-data2.zip › Figure 6—figure supplement 1-source data 2/Figure 6—figure supplement 1G-H-source data 2/nsp16-Flag.tif]

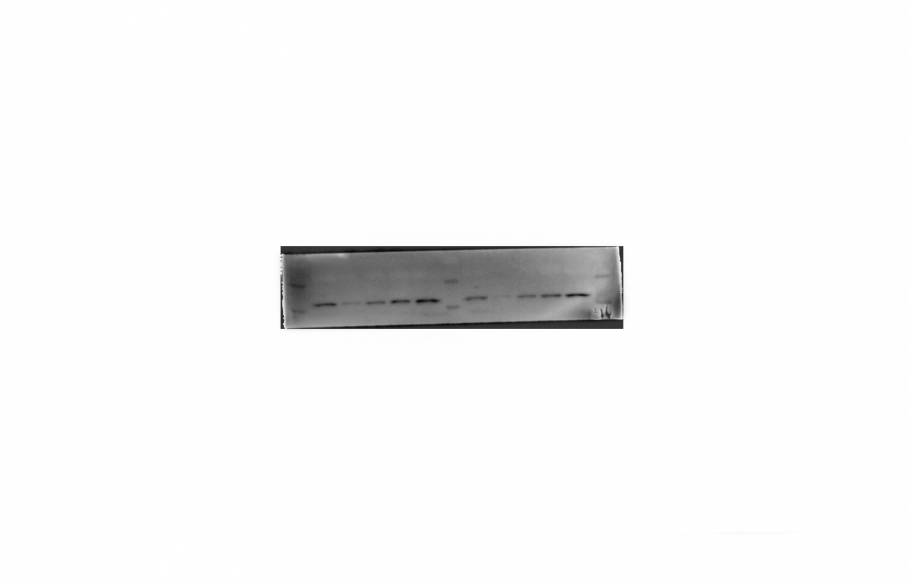

Supplement: Figure 6—figure supplement 1—source data 2. [file elife-102277-fig6-figsupp1-data2.zip › Figure 6—figure supplement 1-source data 2/Figure 6—figure supplement 1G-H-source data 2/SARS-COV-2-N.tif]

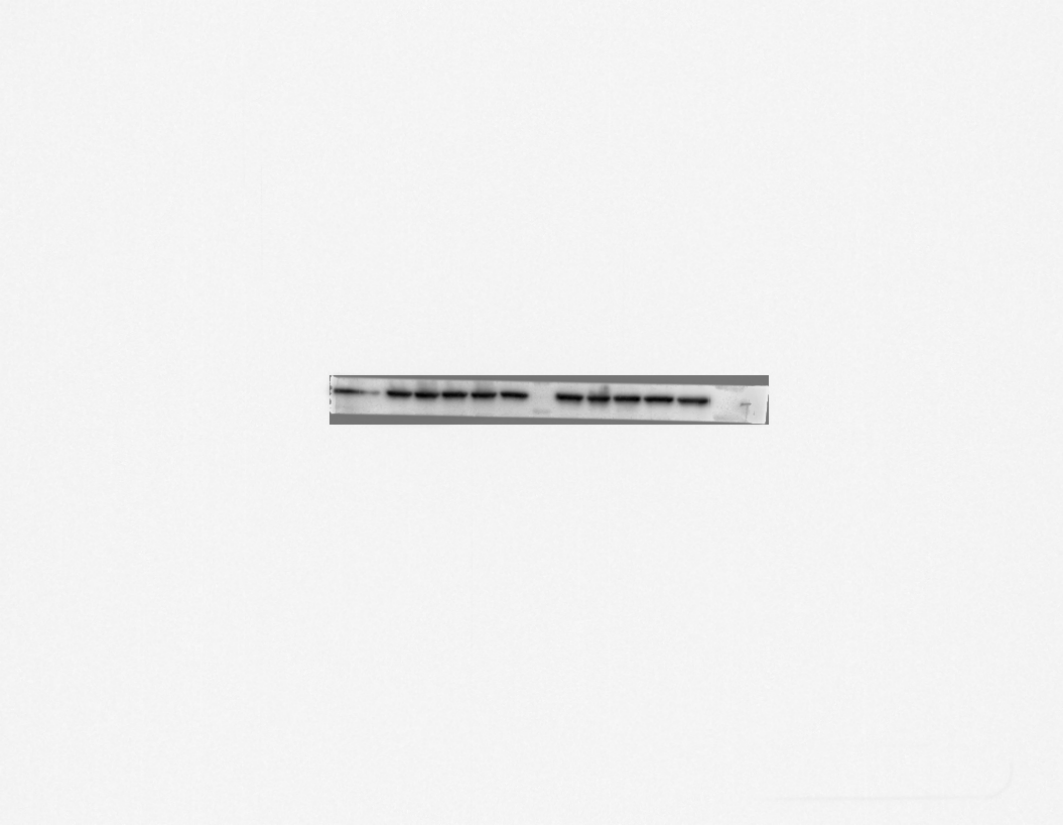

Supplement: Figure 6—figure supplement 1—source data 2. [file elife-102277-fig6-figsupp1-data2.zip › Figure 6—figure supplement 1-source data 2/Figure 6—figure supplement 1G-H-source data 2/Tubulin.tif]

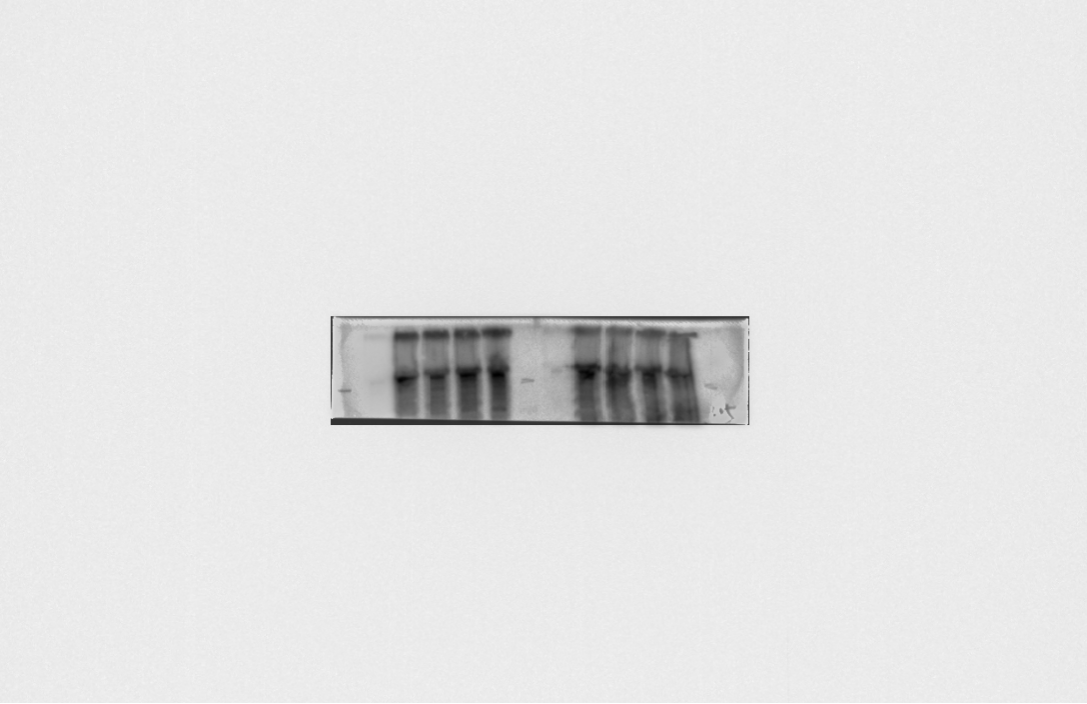

Supplement: Figure 6—figure supplement 1—source data 2. [file elife-102277-fig6-figsupp1-data2.zip › Figure 6—figure supplement 1-source data 2/Figure 6—figure supplement 1G-H-source data 2/UBR5.tif]

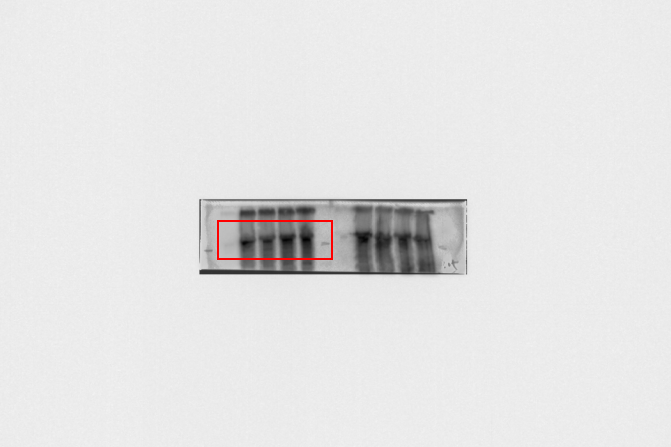

Supplement: Figure 6—figure supplement 1—source data 2. [file elife-102277-fig6-figsupp1-data2.zip › Figure 6—figure supplement 1-source data 2/Figure 6—figure supplement 1G-H-source data 2/UBR5_2.tif]

Figure 6—figure supplement 2G-H

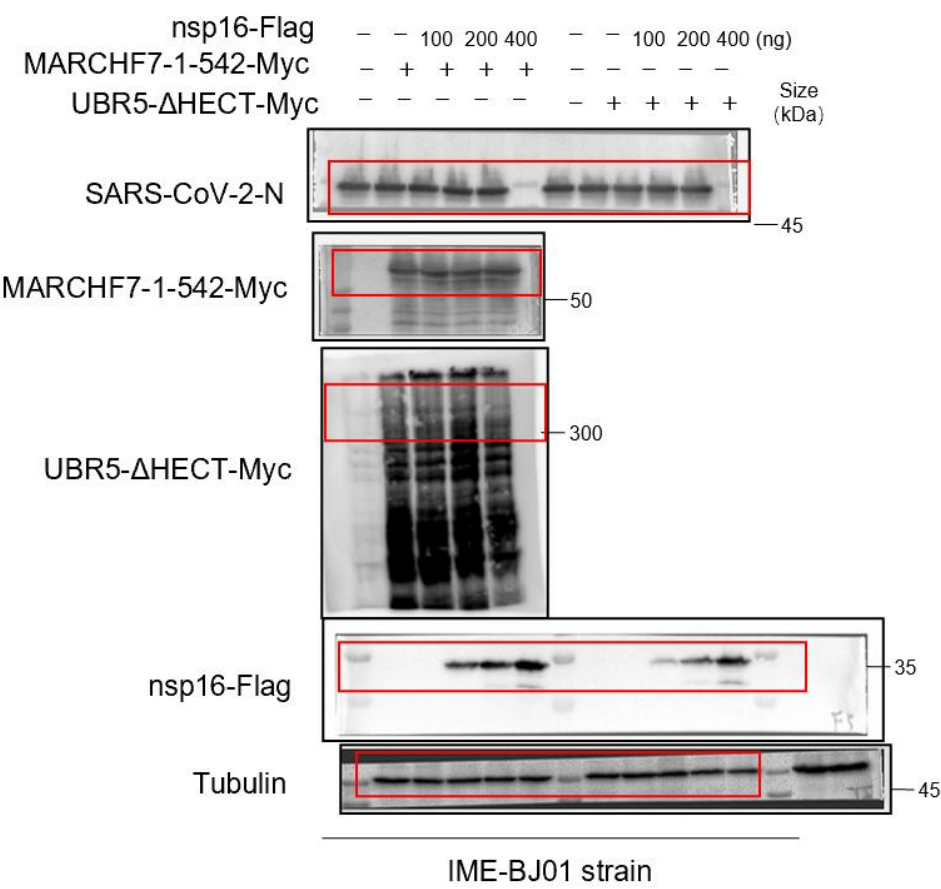

Supplement: Figure 6—figure supplement 2—source data 1. [file elife-102277-fig6-figsupp2-data1.zip › Figure 6—figure supplement 2-source data 1/Figure 6—figure supplement 2 G-H-source data 1 .pdf]

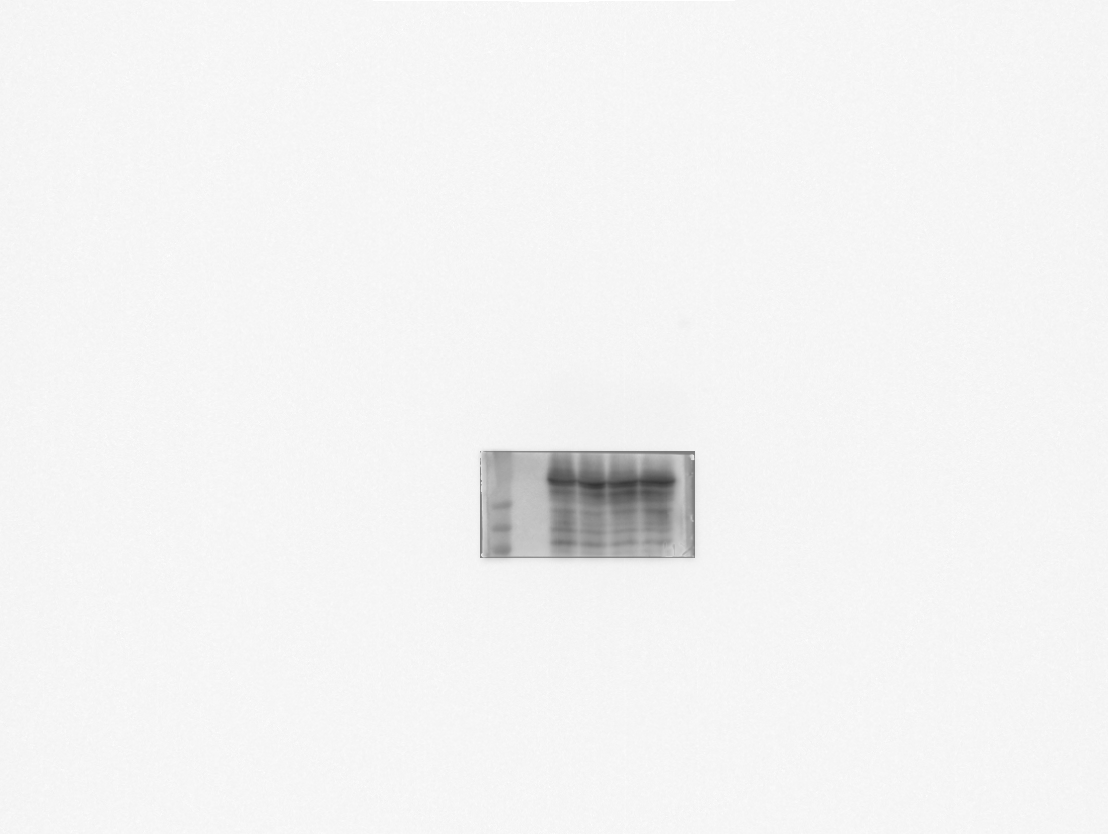

Supplement: Figure 6—figure supplement 2—source data 2. [file elife-102277-fig6-figsupp2-data2.zip › Figure 6—figure supplement 2-source data 2/Figure 6—figure supplement 2G-H-source data 2/1-542-MYC.tif]

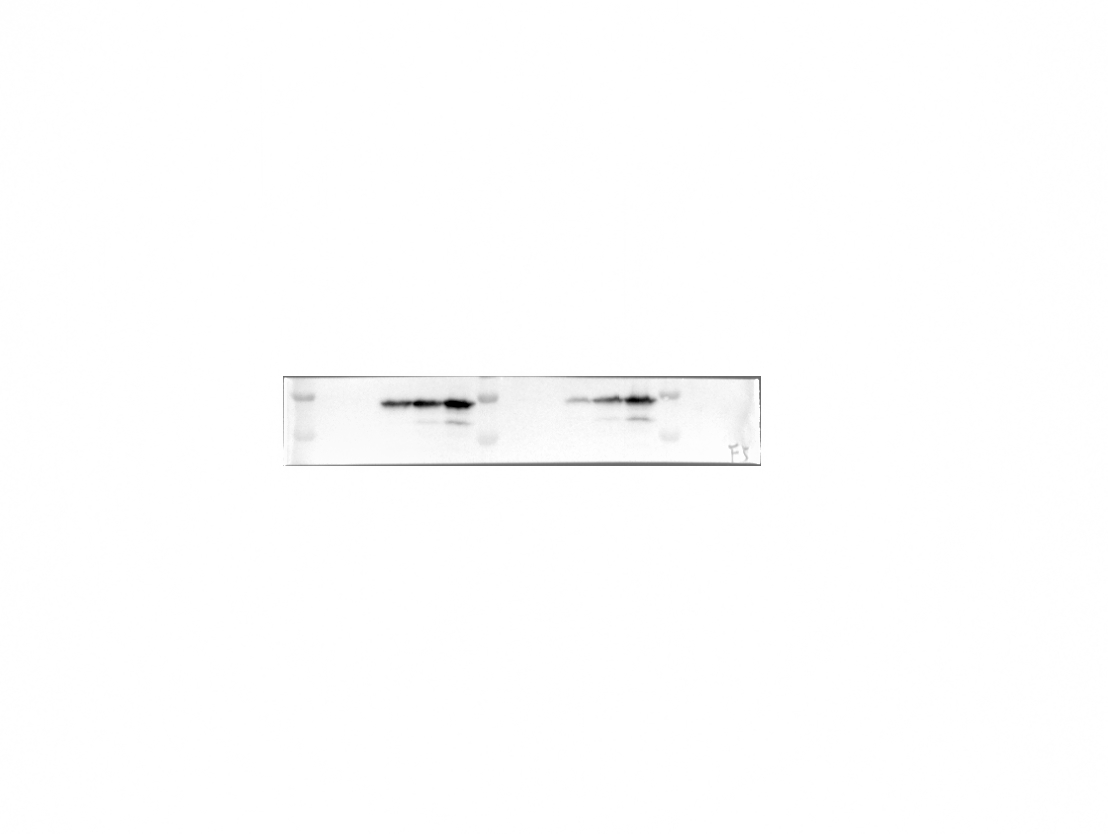

Supplement: Figure 6—figure supplement 2—source data 2. [file elife-102277-fig6-figsupp2-data2.zip › Figure 6—figure supplement 2-source data 2/Figure 6—figure supplement 2G-H-source data 2/nsp16-Flag.tif]

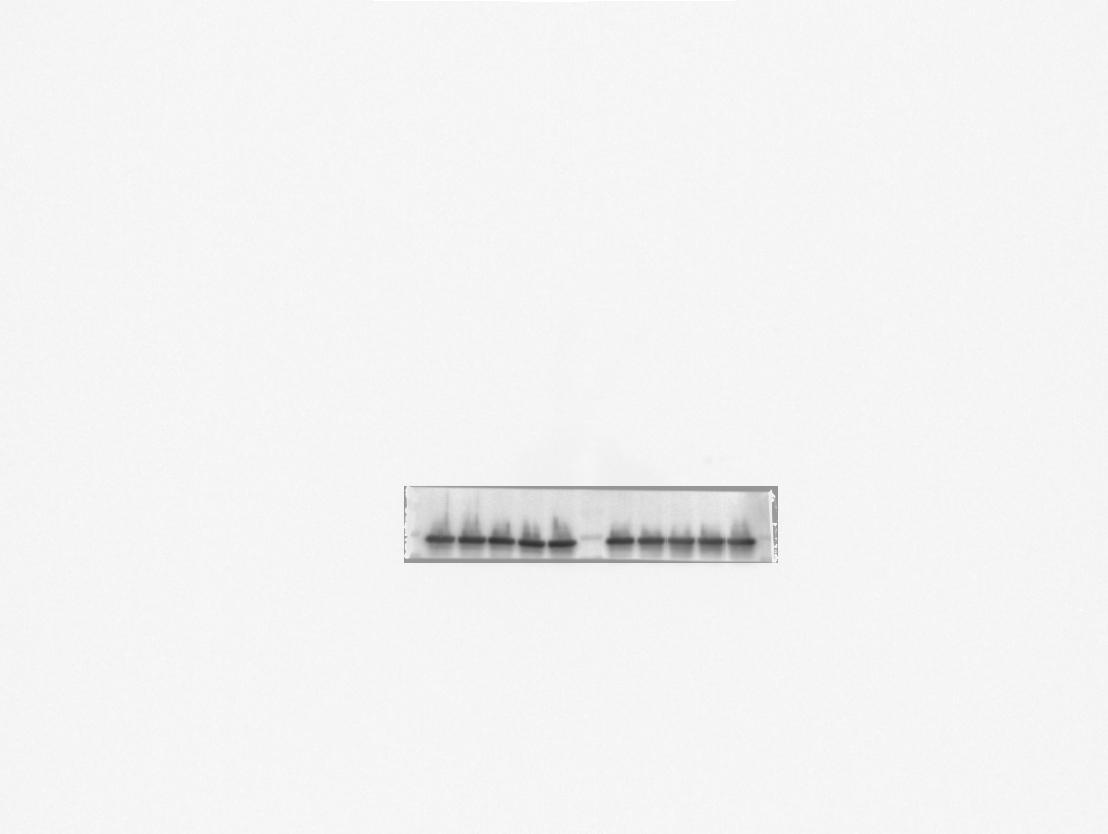

Supplement: Figure 6—figure supplement 2—source data 2. [file elife-102277-fig6-figsupp2-data2.zip › Figure 6—figure supplement 2-source data 2/Figure 6—figure supplement 2G-H-source data 2/SARS-COV-2-N.tif]

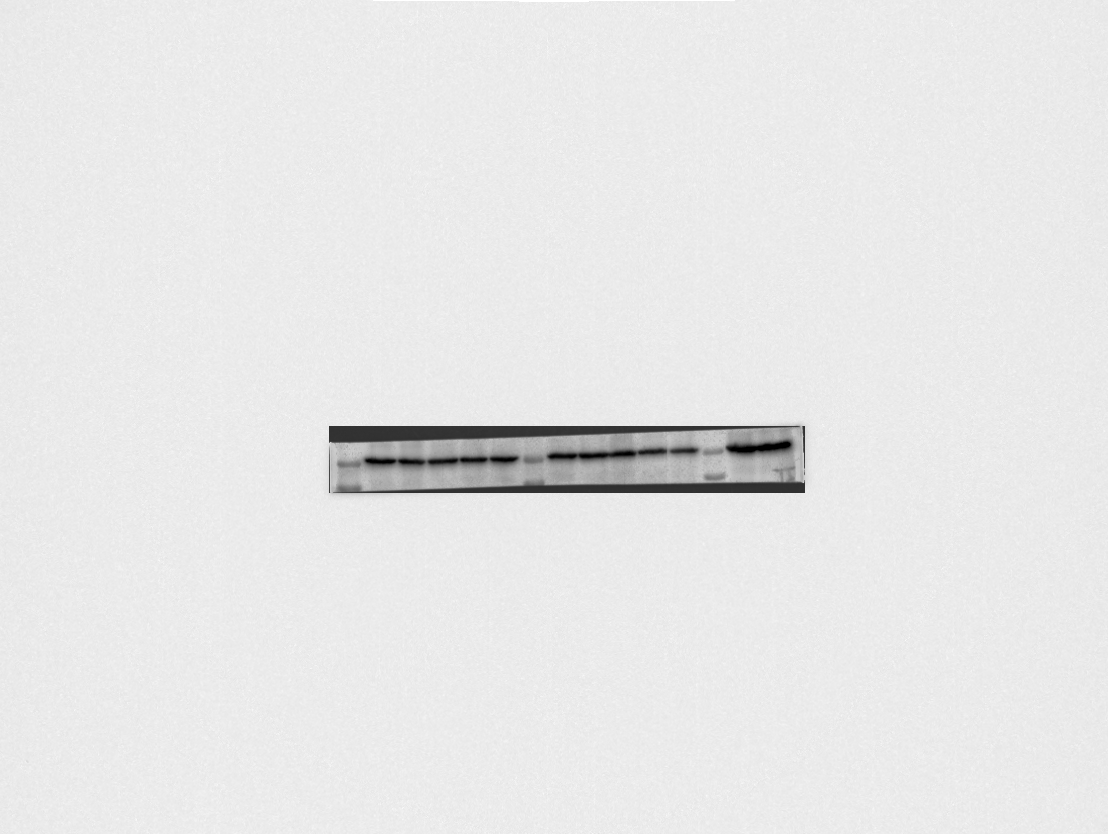

Supplement: Figure 6—figure supplement 2—source data 2. [file elife-102277-fig6-figsupp2-data2.zip › Figure 6—figure supplement 2-source data 2/Figure 6—figure supplement 2G-H-source data 2/Tubulin.tif]

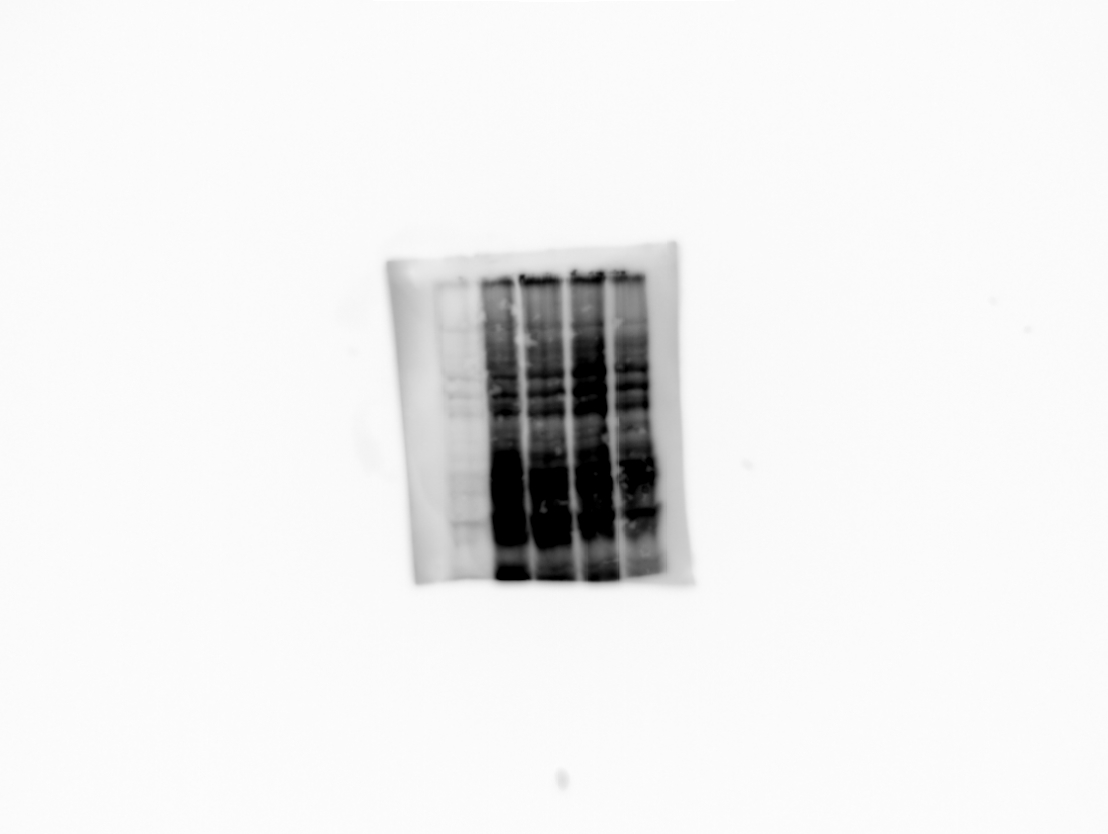

Supplement: Figure 6—figure supplement 2—source data 2. [file elife-102277-fig6-figsupp2-data2.zip › Figure 6—figure supplement 2-source data 2/Figure 6—figure supplement 2G-H-source data 2/ΔHECT.tif]

Figure 6—figure supplement 3B

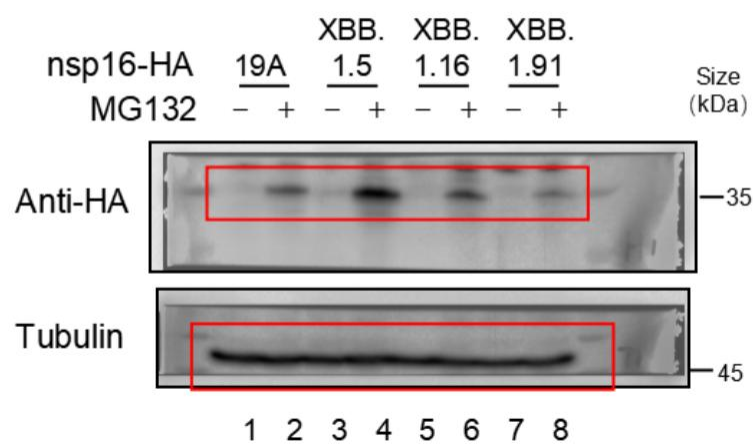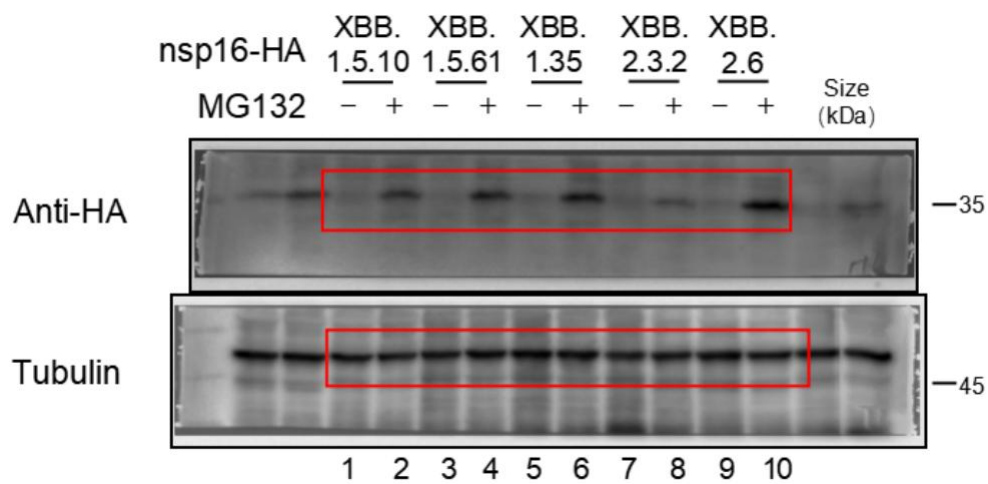

Supplement: Figure 6—figure supplement 3—source data 1. [file elife-102277-fig6-figsupp3-data1.zip › Figure 6—figure supplement 3-source data 1/Figure 6—figure supplement 3B-source data 1.pdf]

Figure 6—figure supplement 3C

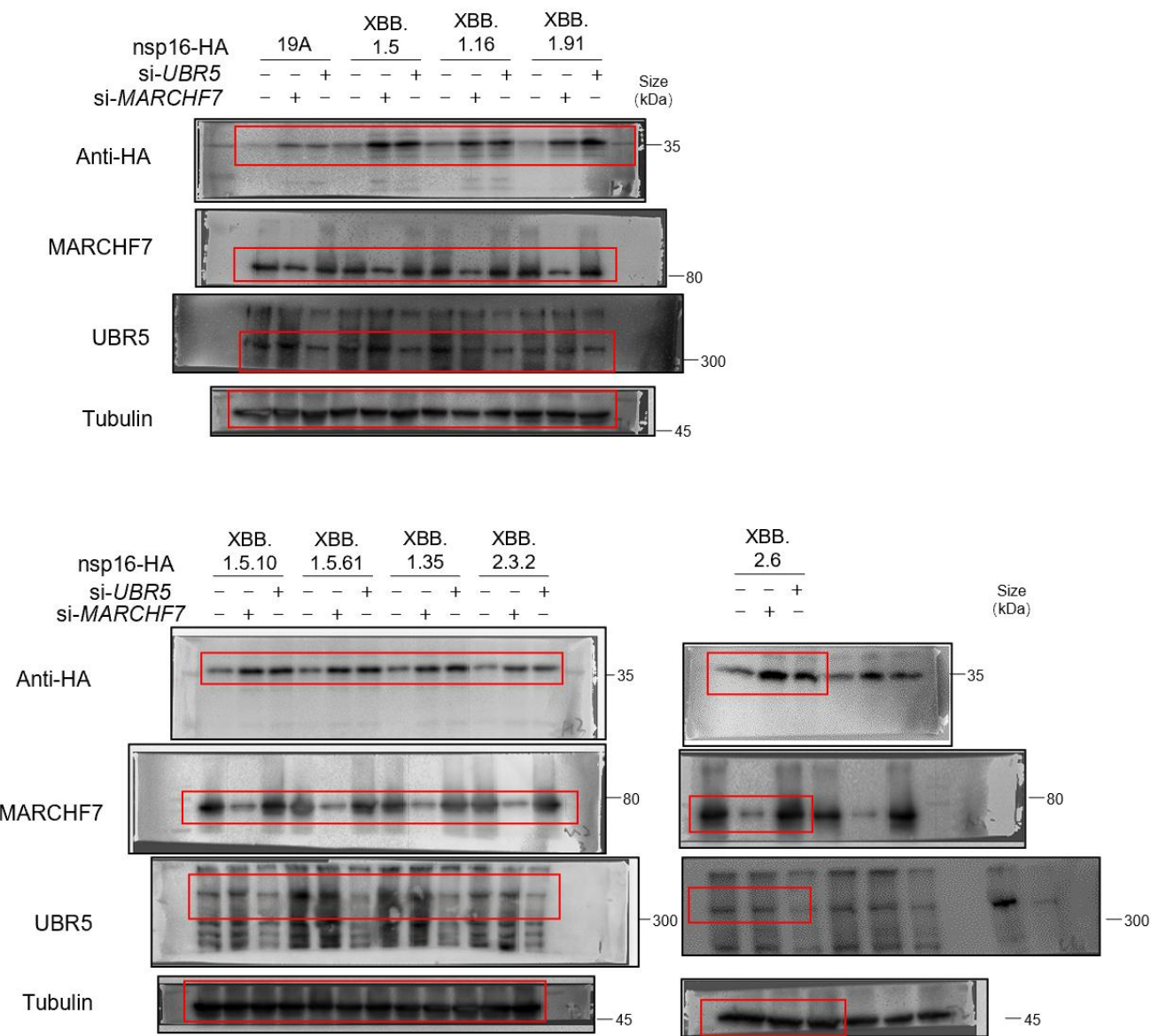

Supplement: Figure 6—figure supplement 3—source data 1. [file elife-102277-fig6-figsupp3-data1.zip › Figure 6—figure supplement 3-source data 1/Figure 6—figure supplement 3C-source data 1.pdf]

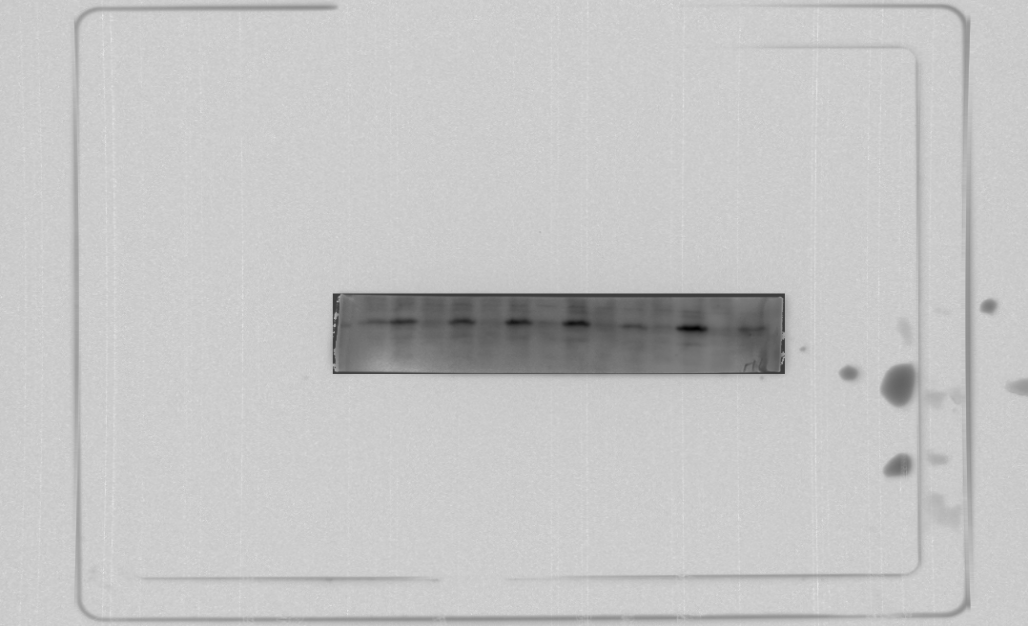

Supplement: Figure 6—figure supplement 3—source data 2. [file elife-102277-fig6-figsupp3-data2.zip › Figure 6—figure supplement 3-source data 2/Figure 6—figure supplement 3B-source data 2/nsp16-HA-2.tif]

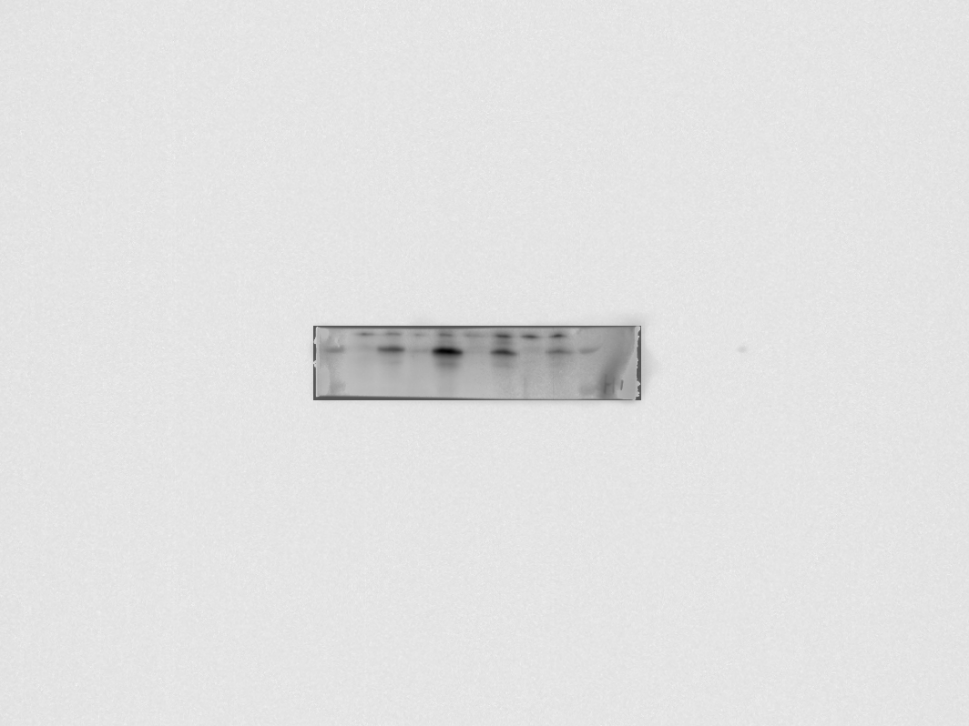

Supplement: Figure 6—figure supplement 3—source data 2. [file elife-102277-fig6-figsupp3-data2.zip › Figure 6—figure supplement 3-source data 2/Figure 6—figure supplement 3B-source data 2/nsp16-HA.tif]

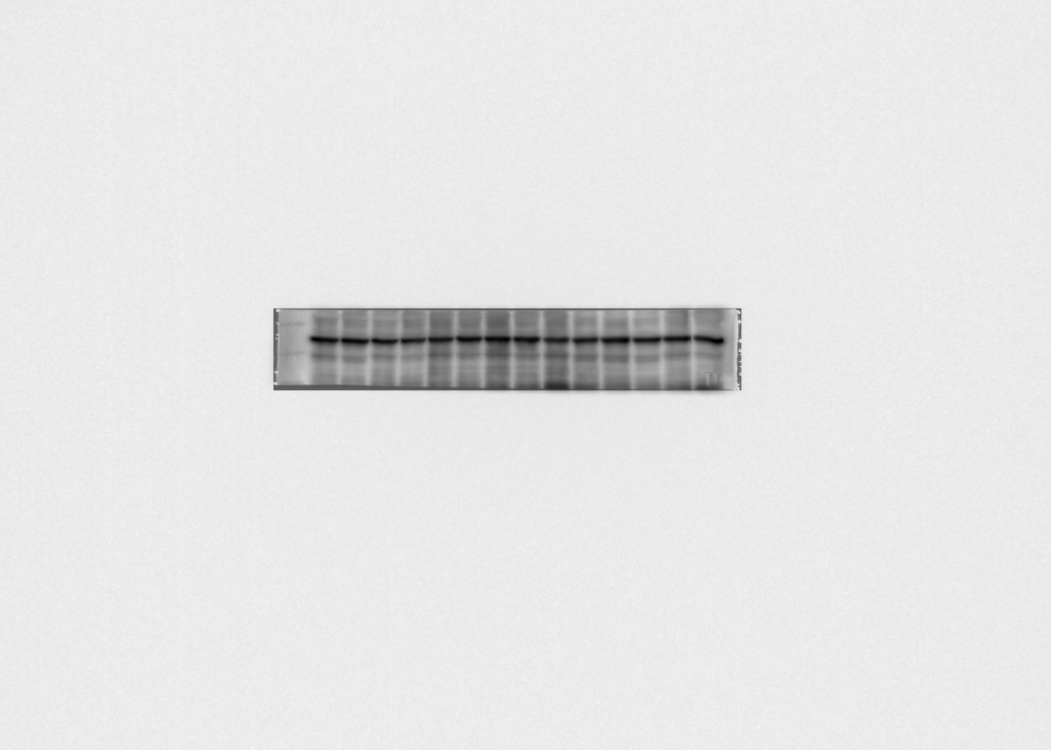

Supplement: Figure 6—figure supplement 3—source data 2. [file elife-102277-fig6-figsupp3-data2.zip › Figure 6—figure supplement 3-source data 2/Figure 6—figure supplement 3B-source data 2/Tubulin-2.tif]

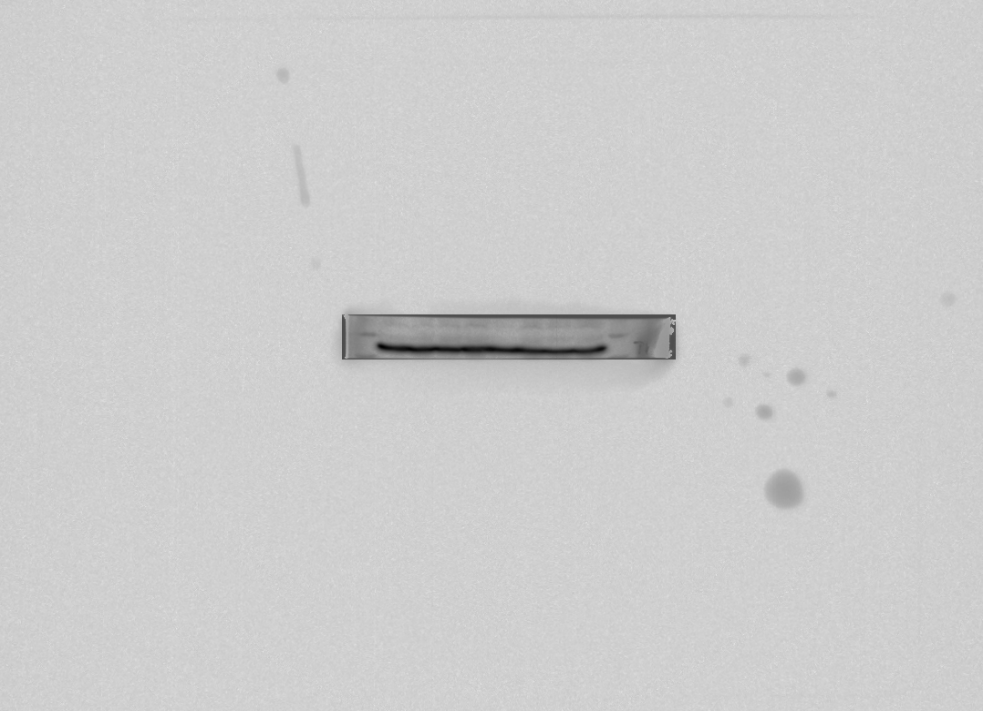

Supplement: Figure 6—figure supplement 3—source data 2. [file elife-102277-fig6-figsupp3-data2.zip › Figure 6—figure supplement 3-source data 2/Figure 6—figure supplement 3B-source data 2/Tubulin.tif]

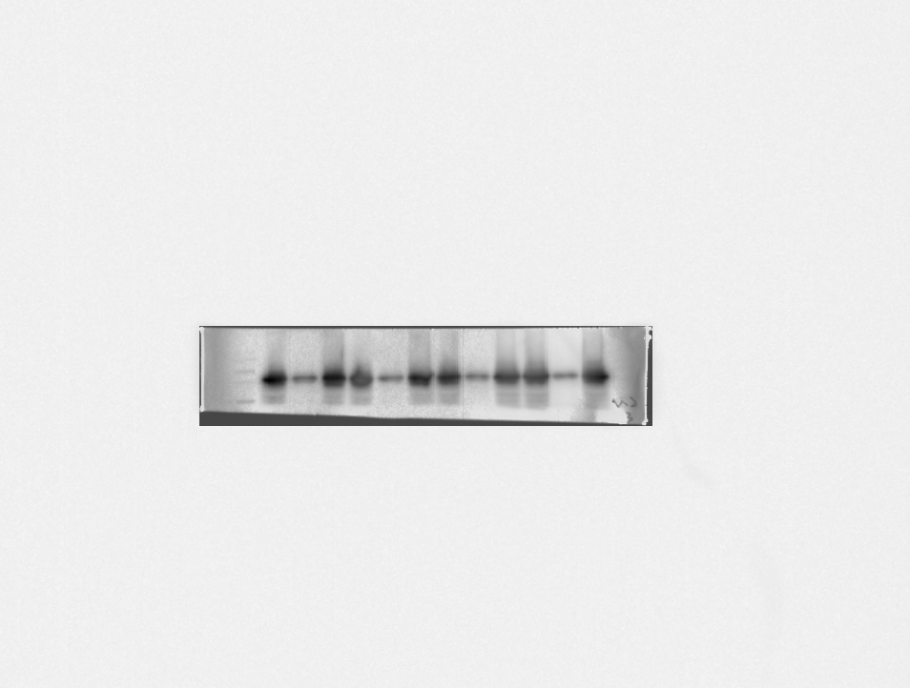

Supplement: Figure 6—figure supplement 3—source data 2. [file elife-102277-fig6-figsupp3-data2.zip › Figure 6—figure supplement 3-source data 2/Figure 6—figure supplement 3C-source data 2/MARCHF7-2.tif]

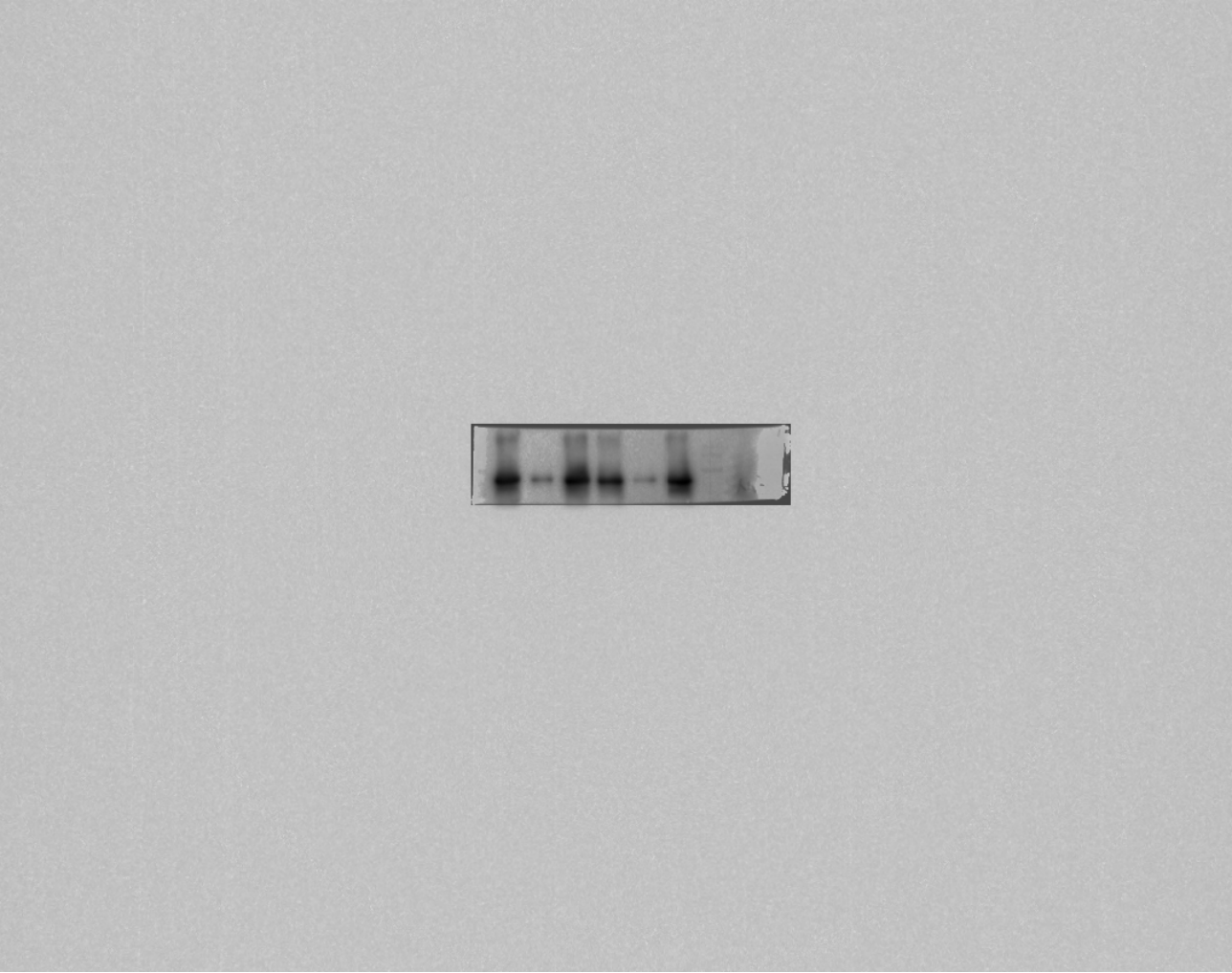

Supplement: Figure 6—figure supplement 3—source data 2. [file elife-102277-fig6-figsupp3-data2.zip › Figure 6—figure supplement 3-source data 2/Figure 6—figure supplement 3C-source data 2/MARCHF7-3.tif]
