# Supplementary figures and images for: SARS-CoV-2 nsp16 is regulated by host E3 ubiquitin ligases, UBR5 and MARCHF7 (part 3 of 3)
Source: eLife. 2025 May 13;13:RP102277. doi: 10.7554/eLife.102277 (PMC12074641; doi:10.7554/eLife.102277)

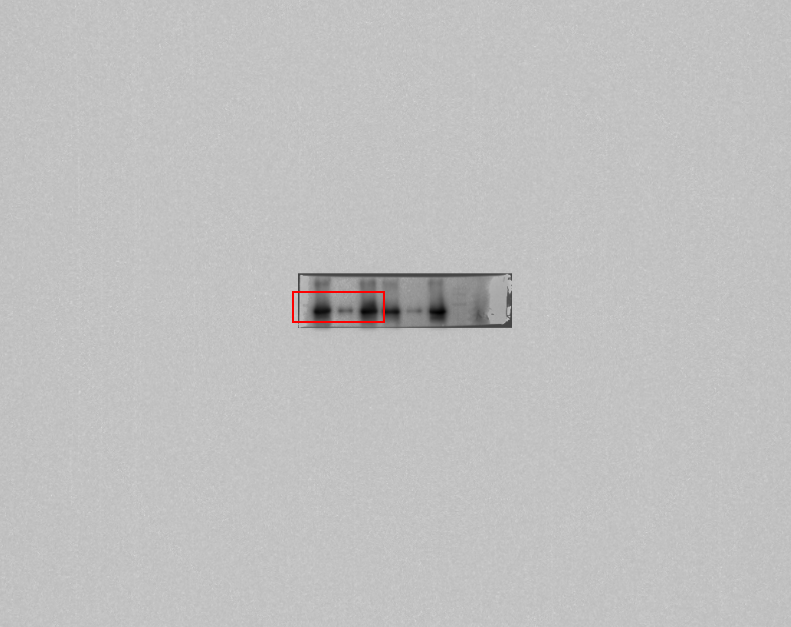

Supplement: Figure 6—figure supplement 3—source data 2. [file elife-102277-fig6-figsupp3-data2.zip › Figure 6—figure supplement 3-source data 2/Figure 6—figure supplement 3C-source data 2/MARCHF7-3_2.tif]

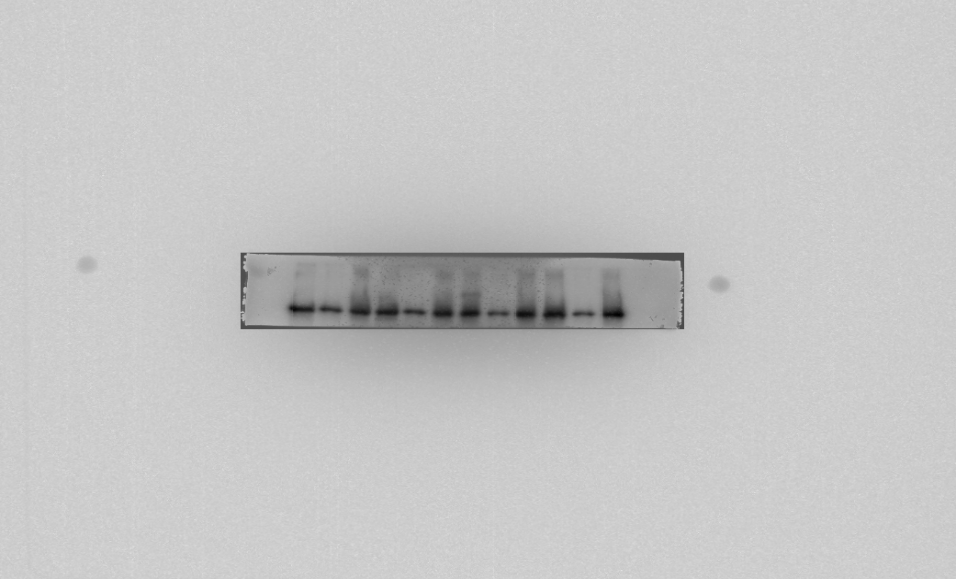

Supplement: Figure 6—figure supplement 3—source data 2. [file elife-102277-fig6-figsupp3-data2.zip › Figure 6—figure supplement 3-source data 2/Figure 6—figure supplement 3C-source data 2/MARCHF7.tif]

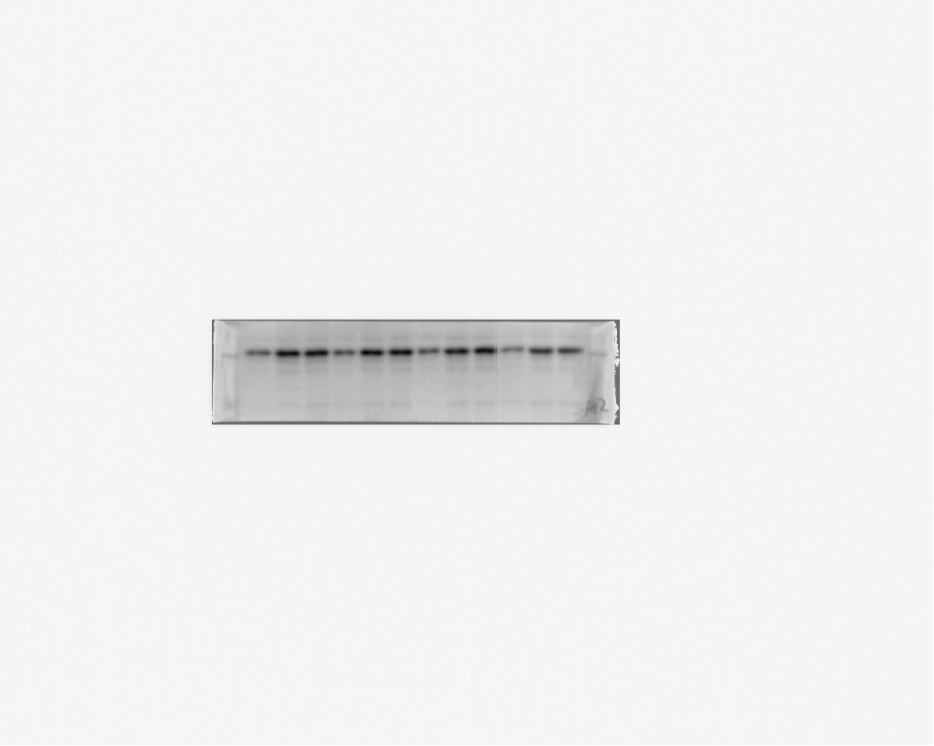

Supplement: Figure 6—figure supplement 3—source data 2. [file elife-102277-fig6-figsupp3-data2.zip › Figure 6—figure supplement 3-source data 2/Figure 6—figure supplement 3C-source data 2/nsp16-HA-2.tif]

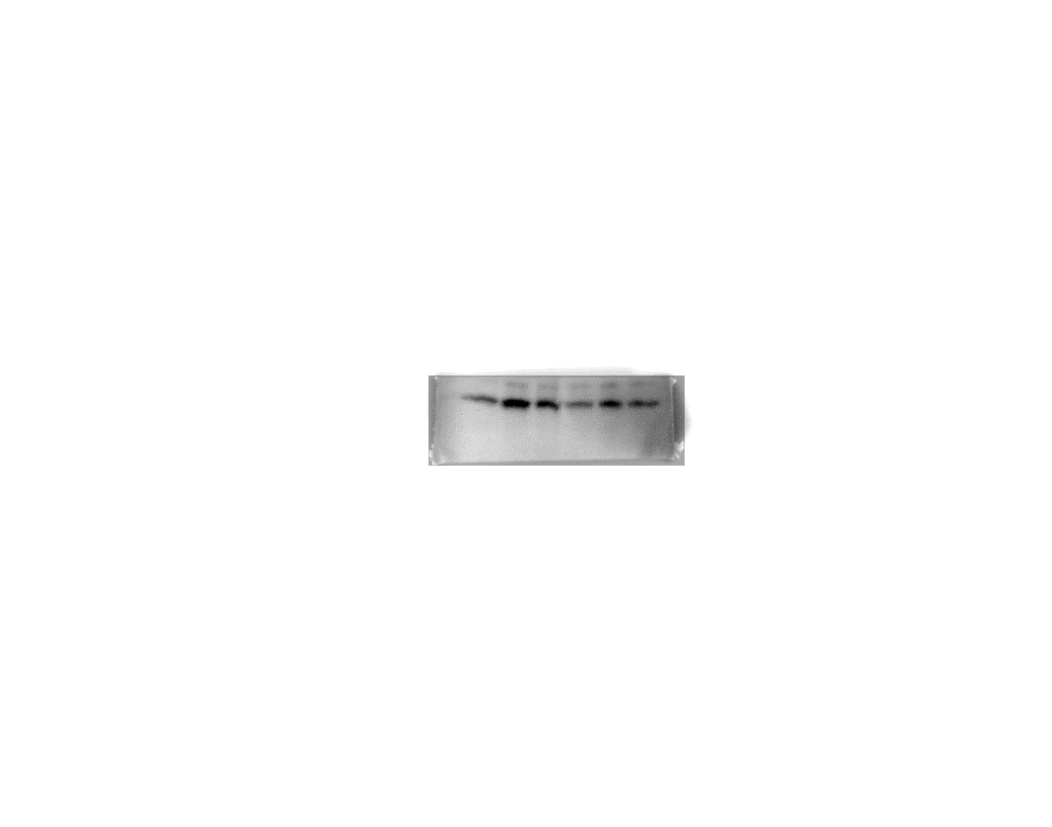

Supplement: Figure 6—figure supplement 3—source data 2. [file elife-102277-fig6-figsupp3-data2.zip › Figure 6—figure supplement 3-source data 2/Figure 6—figure supplement 3C-source data 2/nsp16-HA-3.tif]

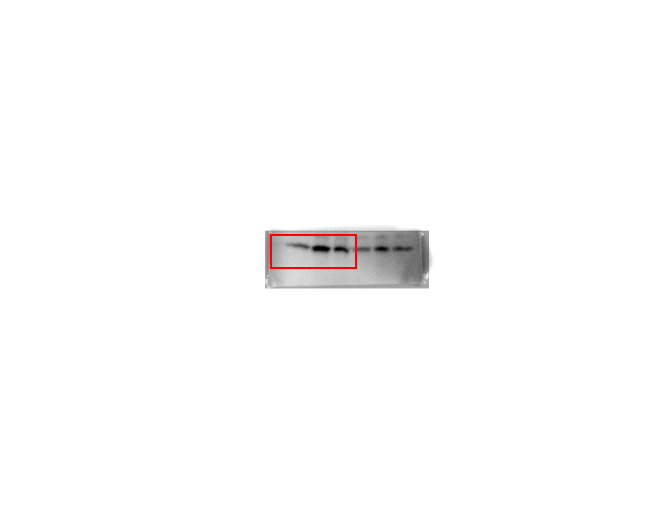

Supplement: Figure 6—figure supplement 3—source data 2. [file elife-102277-fig6-figsupp3-data2.zip › Figure 6—figure supplement 3-source data 2/Figure 6—figure supplement 3C-source data 2/nsp16-HA-3_2.tif]

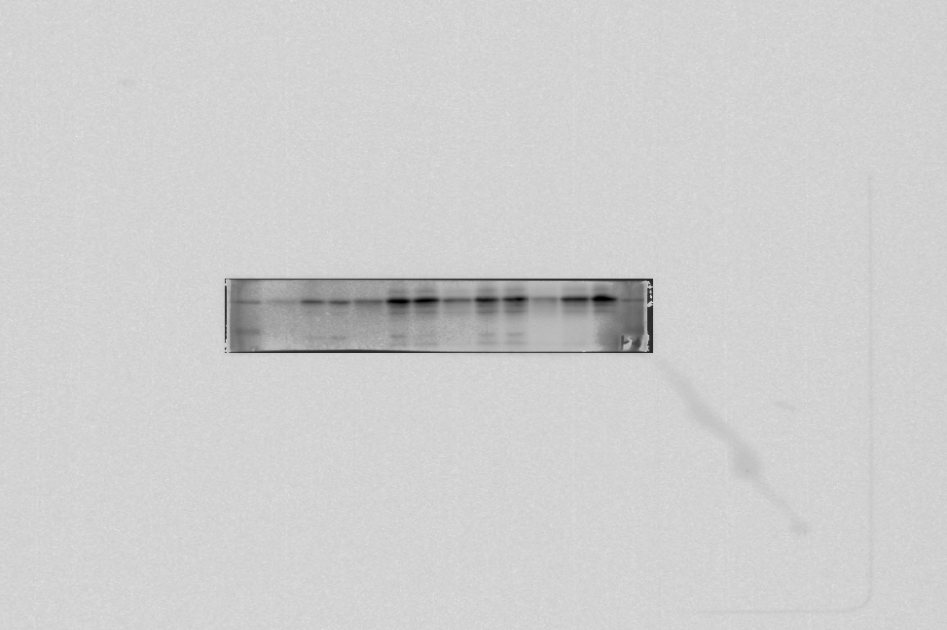

Supplement: Figure 6—figure supplement 3—source data 2. [file elife-102277-fig6-figsupp3-data2.zip › Figure 6—figure supplement 3-source data 2/Figure 6—figure supplement 3C-source data 2/nsp16-HA.tif]

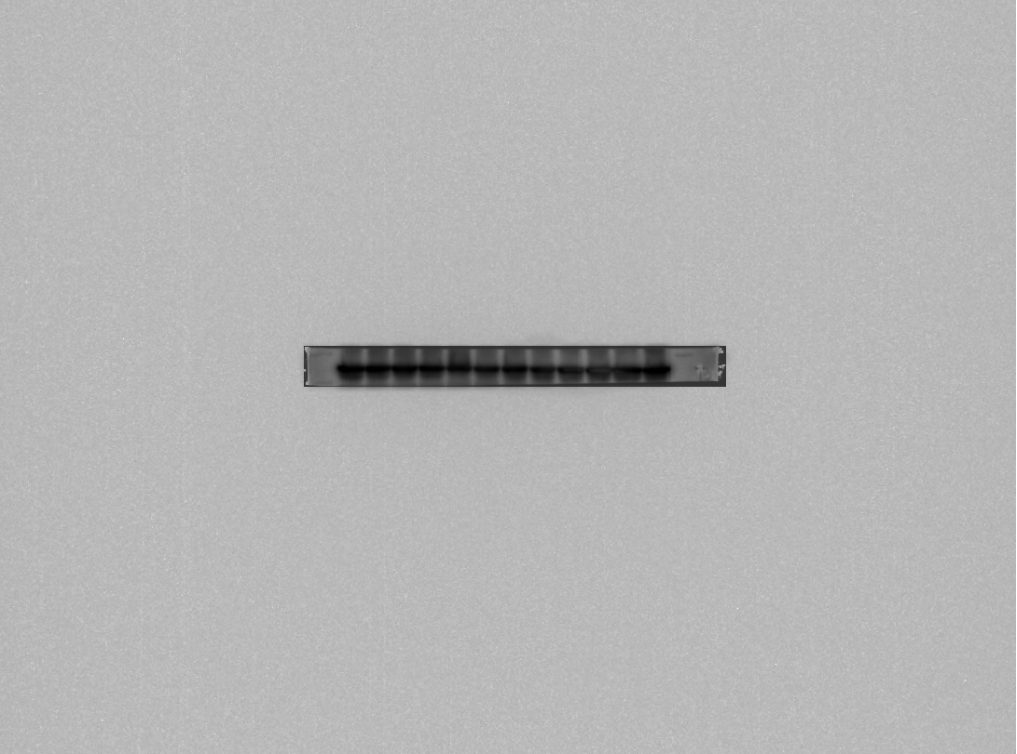

Supplement: Figure 6—figure supplement 3—source data 2. [file elife-102277-fig6-figsupp3-data2.zip › Figure 6—figure supplement 3-source data 2/Figure 6—figure supplement 3C-source data 2/Tubulin-2.tif]

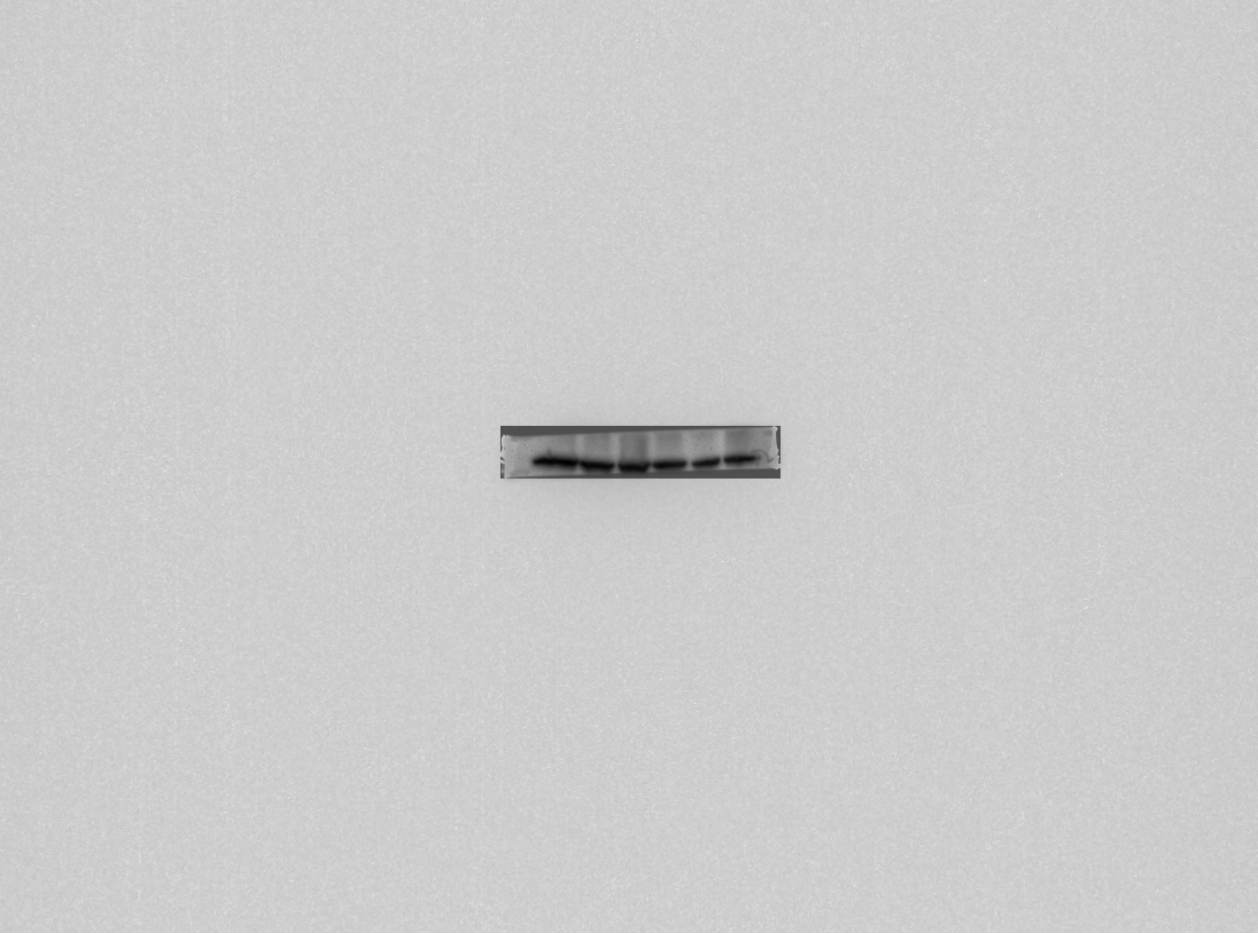

Supplement: Figure 6—figure supplement 3—source data 2. [file elife-102277-fig6-figsupp3-data2.zip › Figure 6—figure supplement 3-source data 2/Figure 6—figure supplement 3C-source data 2/Tubulin-3.tif]

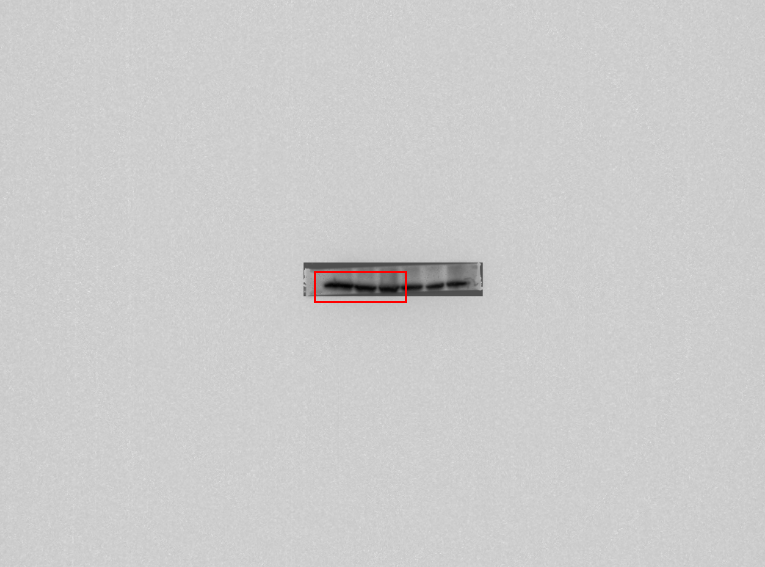

Supplement: Figure 6—figure supplement 3—source data 2. [file elife-102277-fig6-figsupp3-data2.zip › Figure 6—figure supplement 3-source data 2/Figure 6—figure supplement 3C-source data 2/Tubulin-3_2.tif]

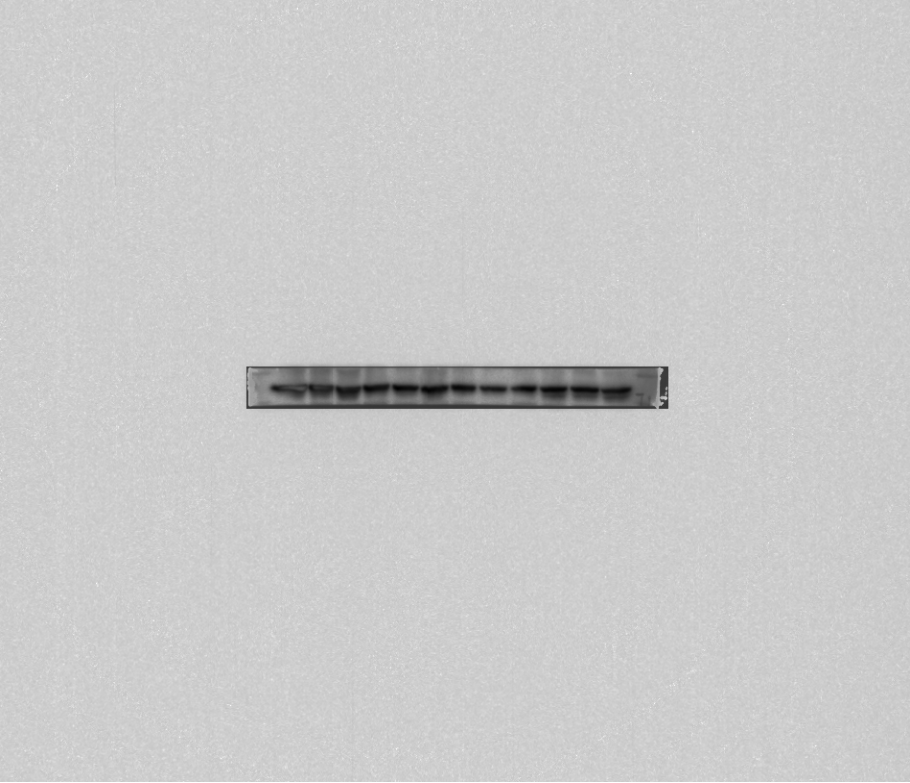

Supplement: Figure 6—figure supplement 3—source data 2. [file elife-102277-fig6-figsupp3-data2.zip › Figure 6—figure supplement 3-source data 2/Figure 6—figure supplement 3C-source data 2/Tubulin.tif]

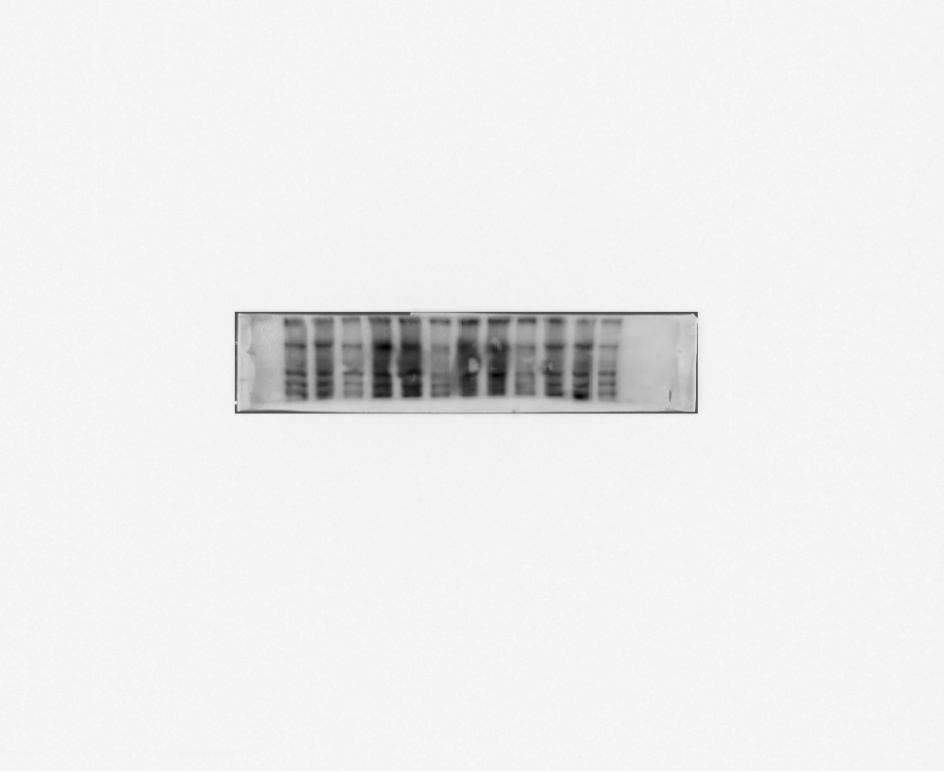

Supplement: Figure 6—figure supplement 3—source data 2. [file elife-102277-fig6-figsupp3-data2.zip › Figure 6—figure supplement 3-source data 2/Figure 6—figure supplement 3C-source data 2/UBR5-2.tif]

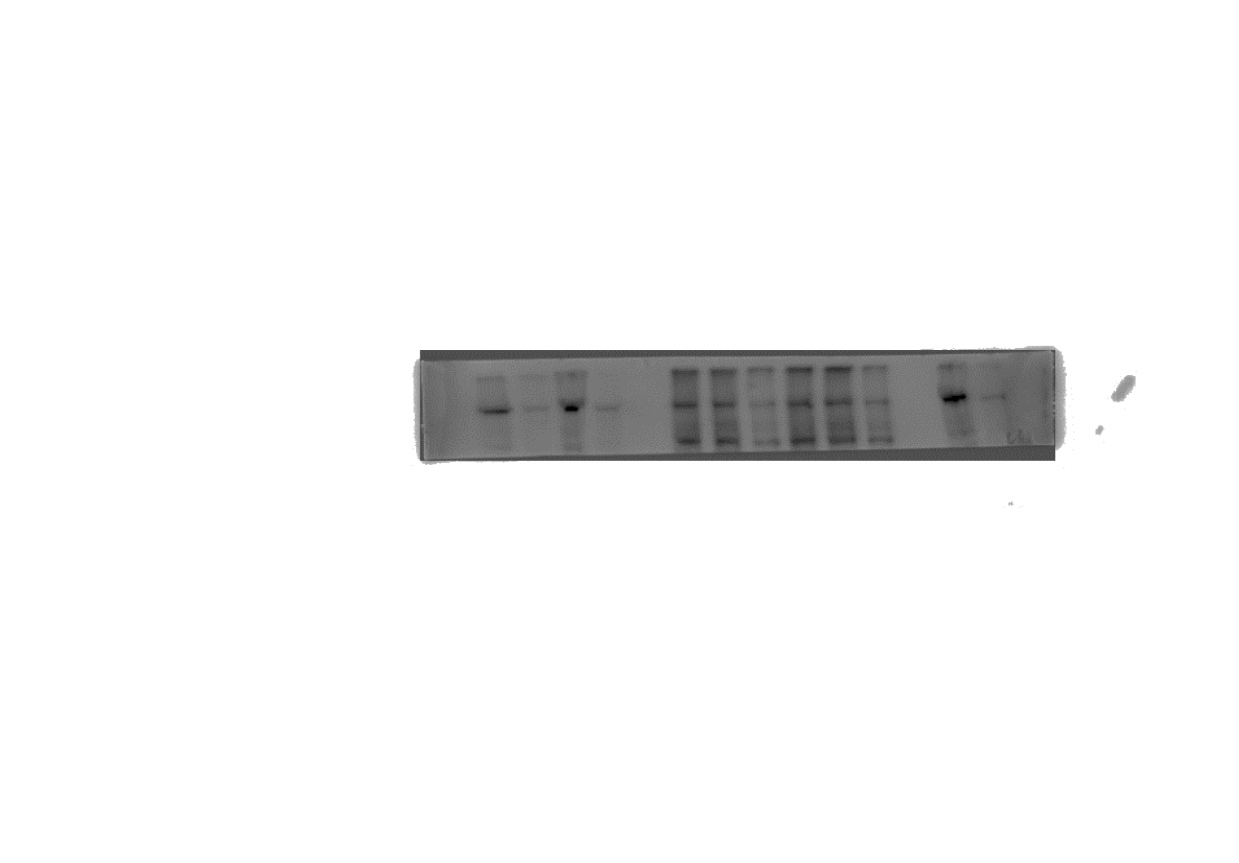

Supplement: Figure 6—figure supplement 3—source data 2. [file elife-102277-fig6-figsupp3-data2.zip › Figure 6—figure supplement 3-source data 2/Figure 6—figure supplement 3C-source data 2/UBR5-3.tif]

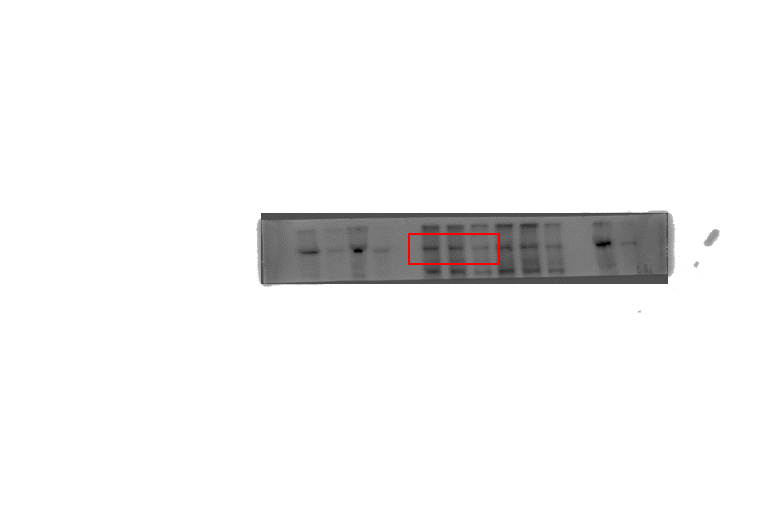

Supplement: Figure 6—figure supplement 3—source data 2. [file elife-102277-fig6-figsupp3-data2.zip › Figure 6—figure supplement 3-source data 2/Figure 6—figure supplement 3C-source data 2/UBR5-3_2.tif]

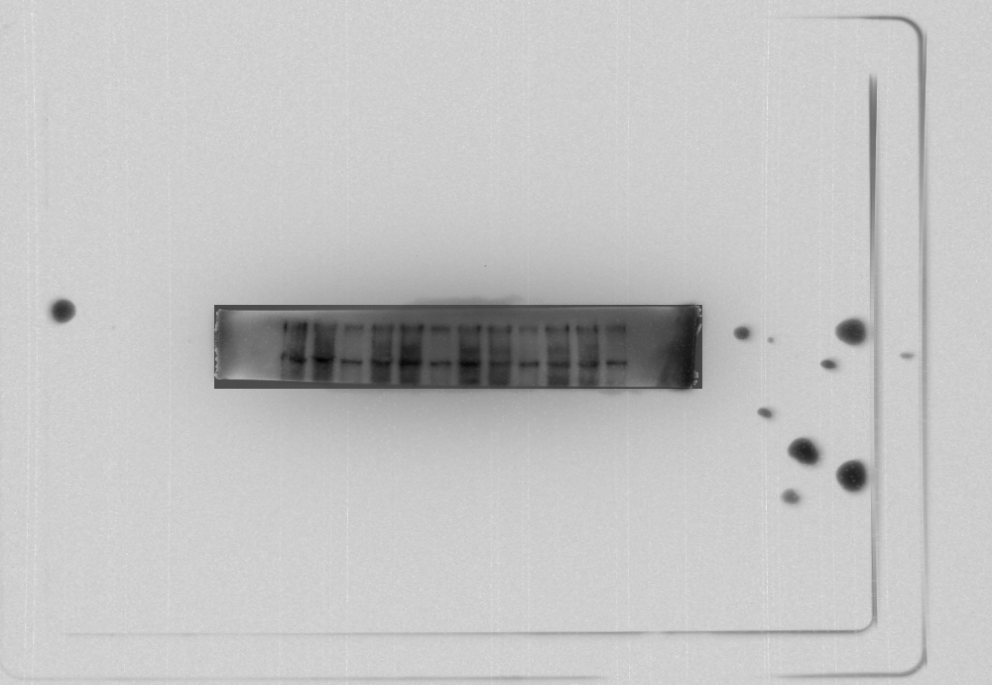

Supplement: Figure 6—figure supplement 3—source data 2. [file elife-102277-fig6-figsupp3-data2.zip › Figure 6—figure supplement 3-source data 2/Figure 6—figure supplement 3C-source data 2/UBR5.tif]

Figure 6—figure supplement 4C

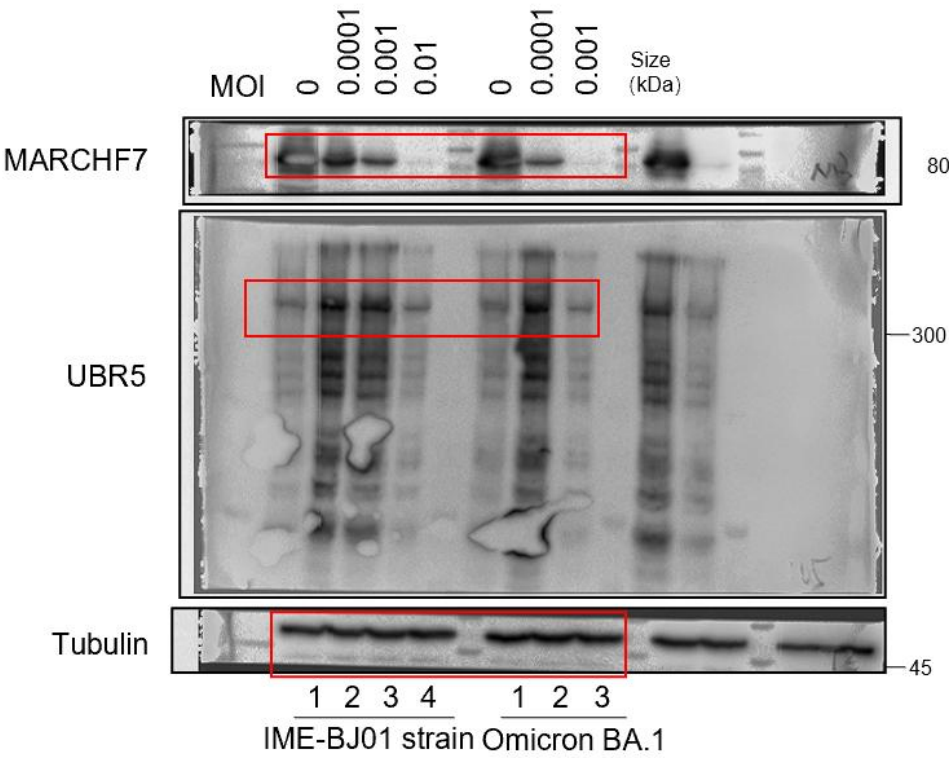

Supplement: Figure 6—figure supplement 4—source data 1. [file elife-102277-fig6-figsupp4-data1.zip › Figure 6—figure supplement 4-source data 1/Figure 6—figure supplement 4C-source data 1.pdf]

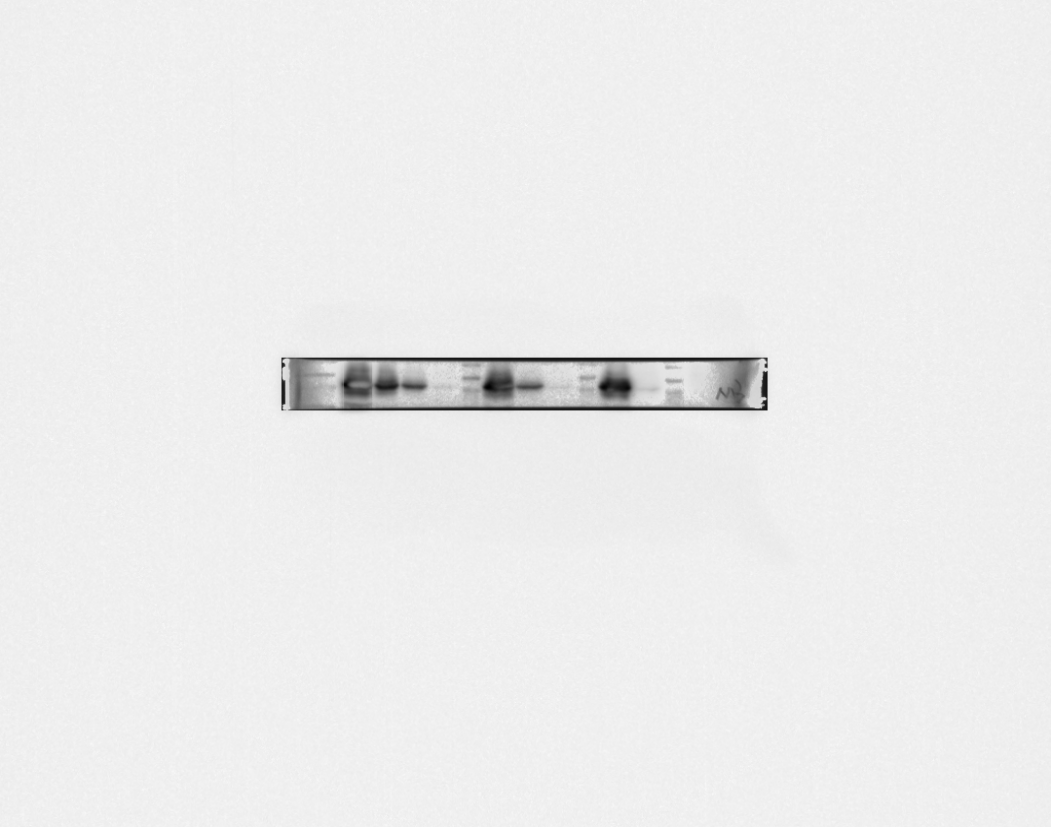

Supplement: Figure 6—figure supplement 4—source data 2. [file elife-102277-fig6-figsupp4-data2.zip › Figure 6—figure supplement 4-source data 2/Figure 6—figure supplement 4C-source data 2/MARCHF7.tif]

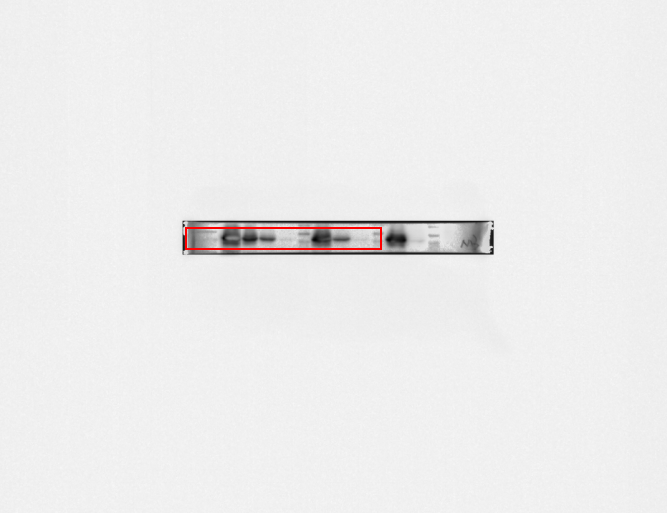

Supplement: Figure 6—figure supplement 4—source data 2. [file elife-102277-fig6-figsupp4-data2.zip › Figure 6—figure supplement 4-source data 2/Figure 6—figure supplement 4C-source data 2/MARCHF7_2.tif]

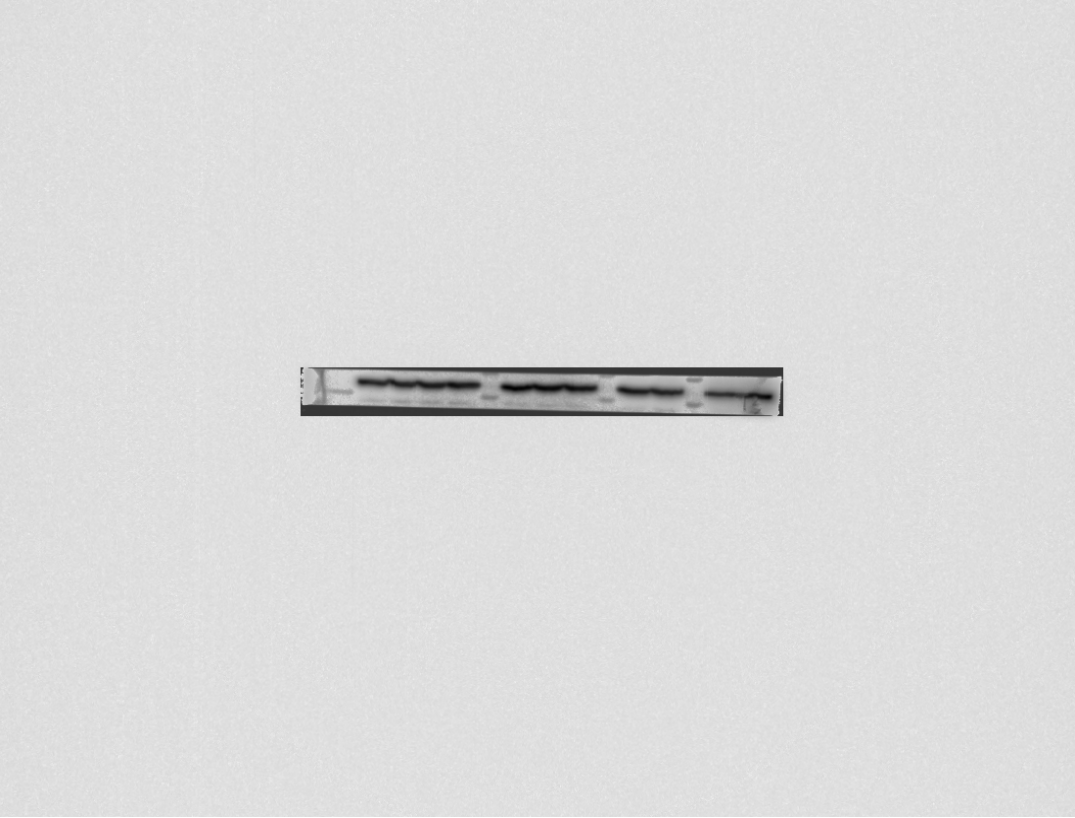

Supplement: Figure 6—figure supplement 4—source data 2. [file elife-102277-fig6-figsupp4-data2.zip › Figure 6—figure supplement 4-source data 2/Figure 6—figure supplement 4C-source data 2/Tubulin.tif]

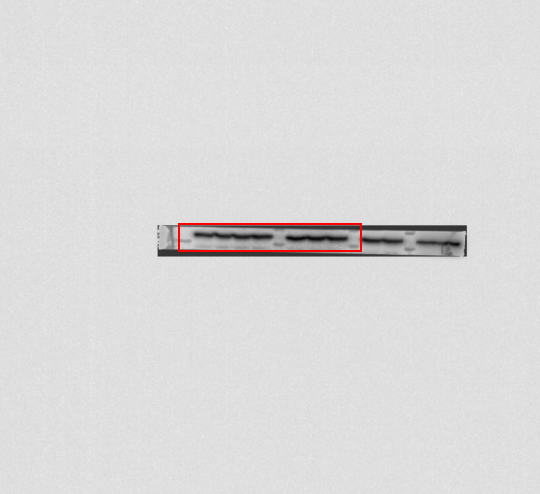

Supplement: Figure 6—figure supplement 4—source data 2. [file elife-102277-fig6-figsupp4-data2.zip › Figure 6—figure supplement 4-source data 2/Figure 6—figure supplement 4C-source data 2/Tubulin_2.tif]

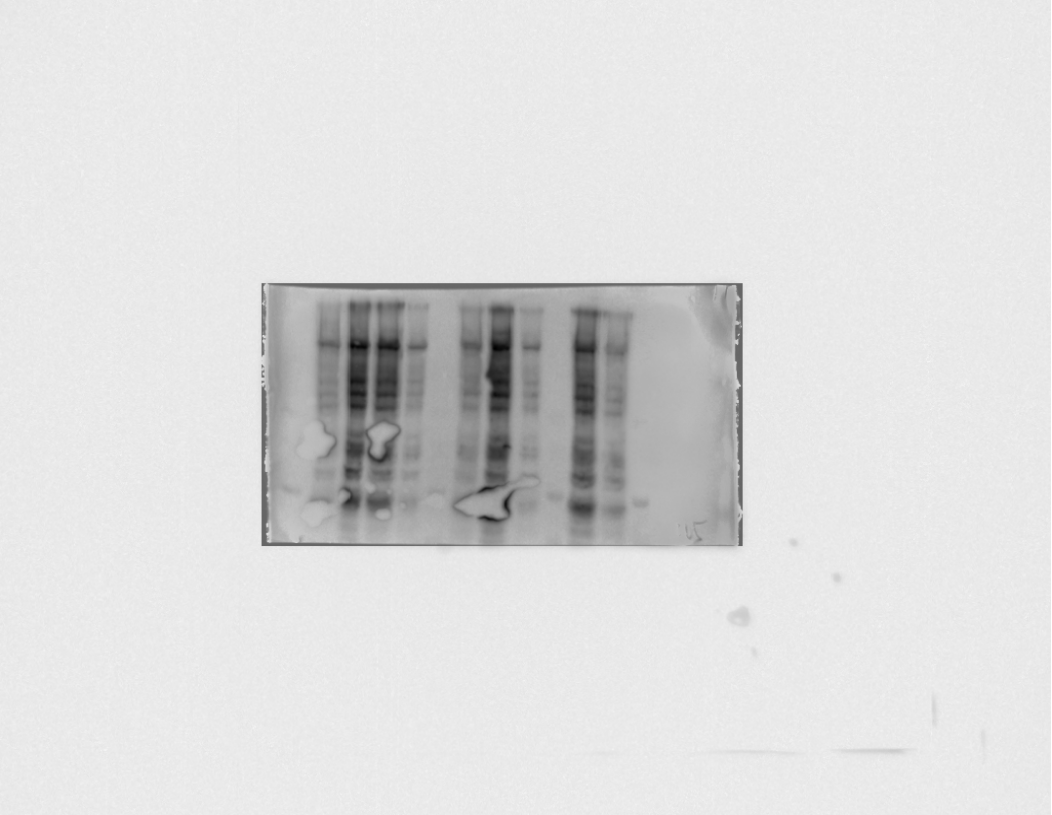

Supplement: Figure 6—figure supplement 4—source data 2. [file elife-102277-fig6-figsupp4-data2.zip › Figure 6—figure supplement 4-source data 2/Figure 6—figure supplement 4C-source data 2/UBR5.tif]

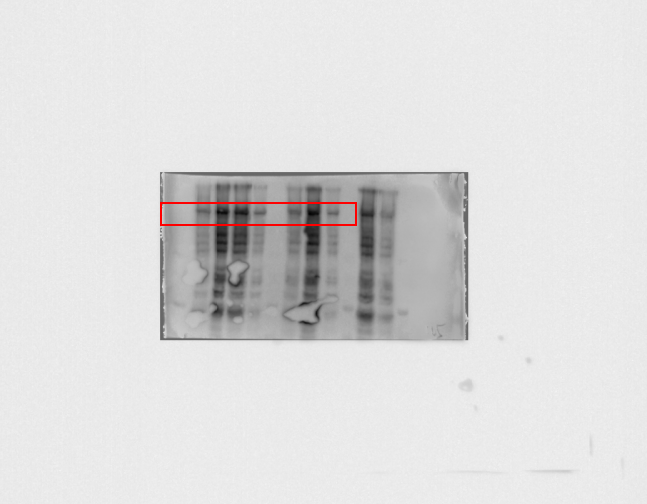

Supplement: Figure 6—figure supplement 4—source data 2. [file elife-102277-fig6-figsupp4-data2.zip › Figure 6—figure supplement 4-source data 2/Figure 6—figure supplement 4C-source data 2/UBR5_2.tif]

Figure 7B

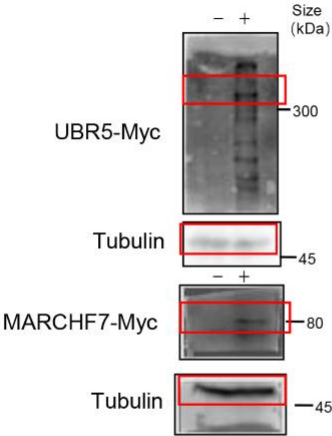

Supplement: Figure 7—source data 1. [file elife-102277-fig7-data1.zip › Figure 7-source data 1/Figure 7B-source data 1.pdf]

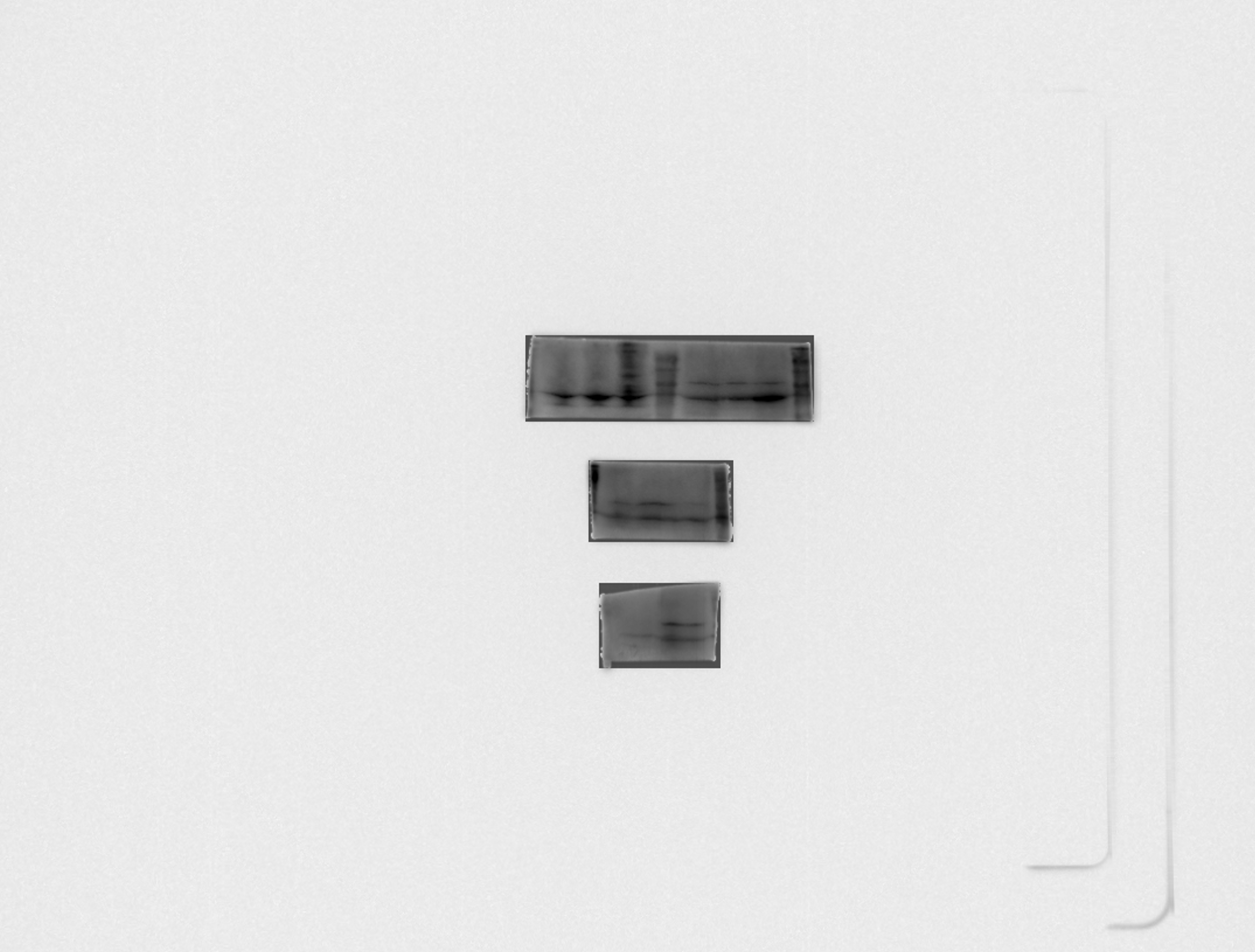

Supplement: Figure 7—source data 2. [file elife-102277-fig7-data2.zip › Figure 7-source data 2/Figure 7B-source data 2/MARCHF7.tif]

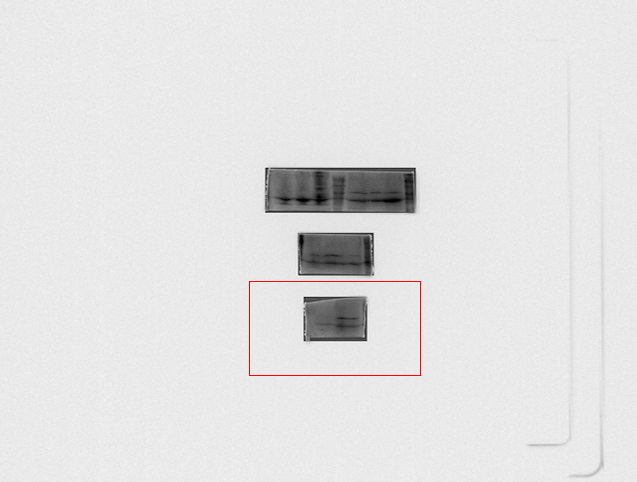

Supplement: Figure 7—source data 2. [file elife-102277-fig7-data2.zip › Figure 7-source data 2/Figure 7B-source data 2/MARCHF7_2.tif]

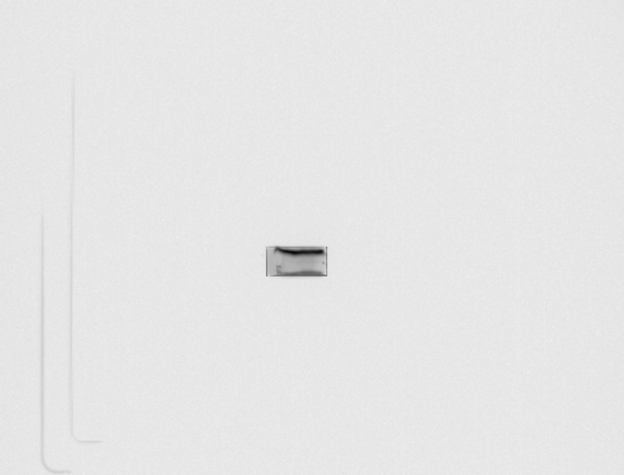

Supplement: Figure 7—source data 2. [file elife-102277-fig7-data2.zip › Figure 7-source data 2/Figure 7B-source data 2/Tubulin-2.tif]

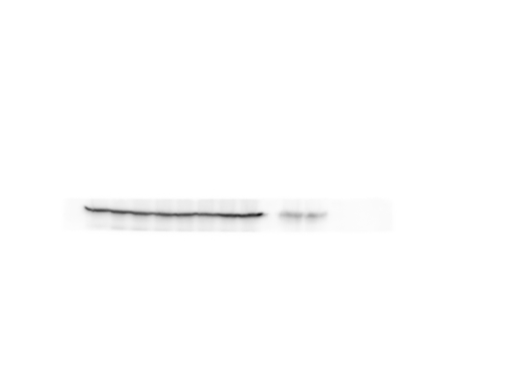

Supplement: Figure 7—source data 2. [file elife-102277-fig7-data2.zip › Figure 7-source data 2/Figure 7B-source data 2/Tubulin.tif]

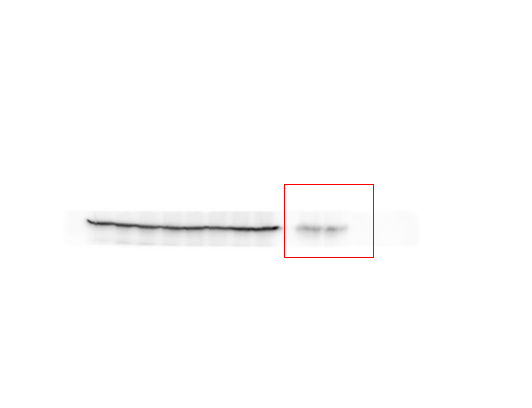

Supplement: Figure 7—source data 2. [file elife-102277-fig7-data2.zip › Figure 7-source data 2/Figure 7B-source data 2/Tubulin_2.tif]

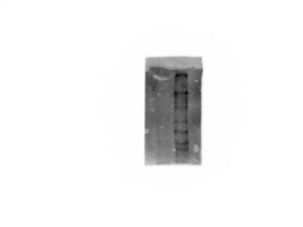

Supplement: Figure 7—source data 2. [file elife-102277-fig7-data2.zip › Figure 7-source data 2/Figure 7B-source data 2/UBR5.tif]

Figure 8—figure supplement 1A

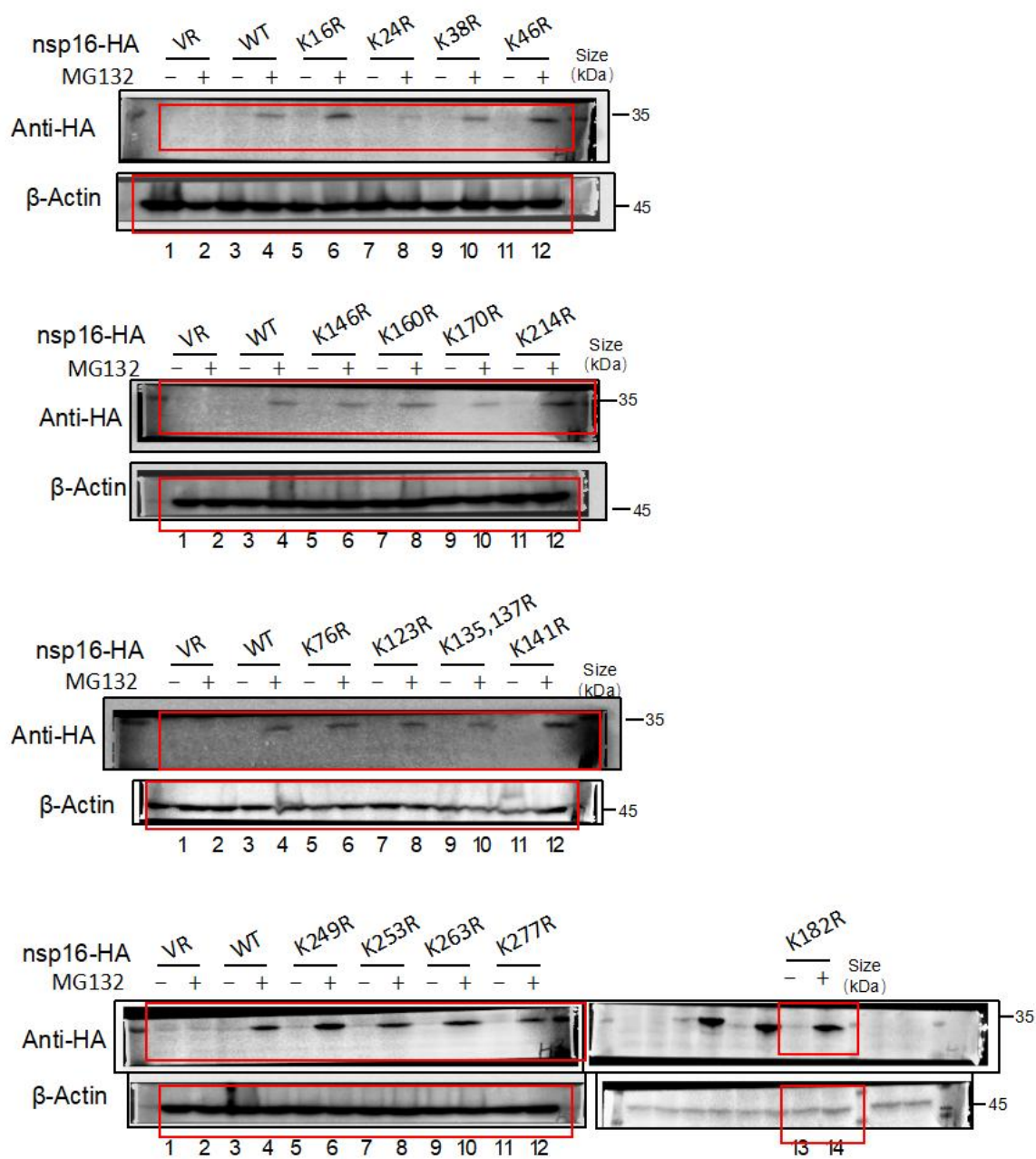

Supplement: Figure 8—figure supplement 1—source data 1. [file elife-102277-fig8-figsupp1-data1.zip › Figure 8—figure supplement 1-source data 1/Figure 8—figure supplement 1A-source data 1.pdf]

Figure 8—figure supplement 1B

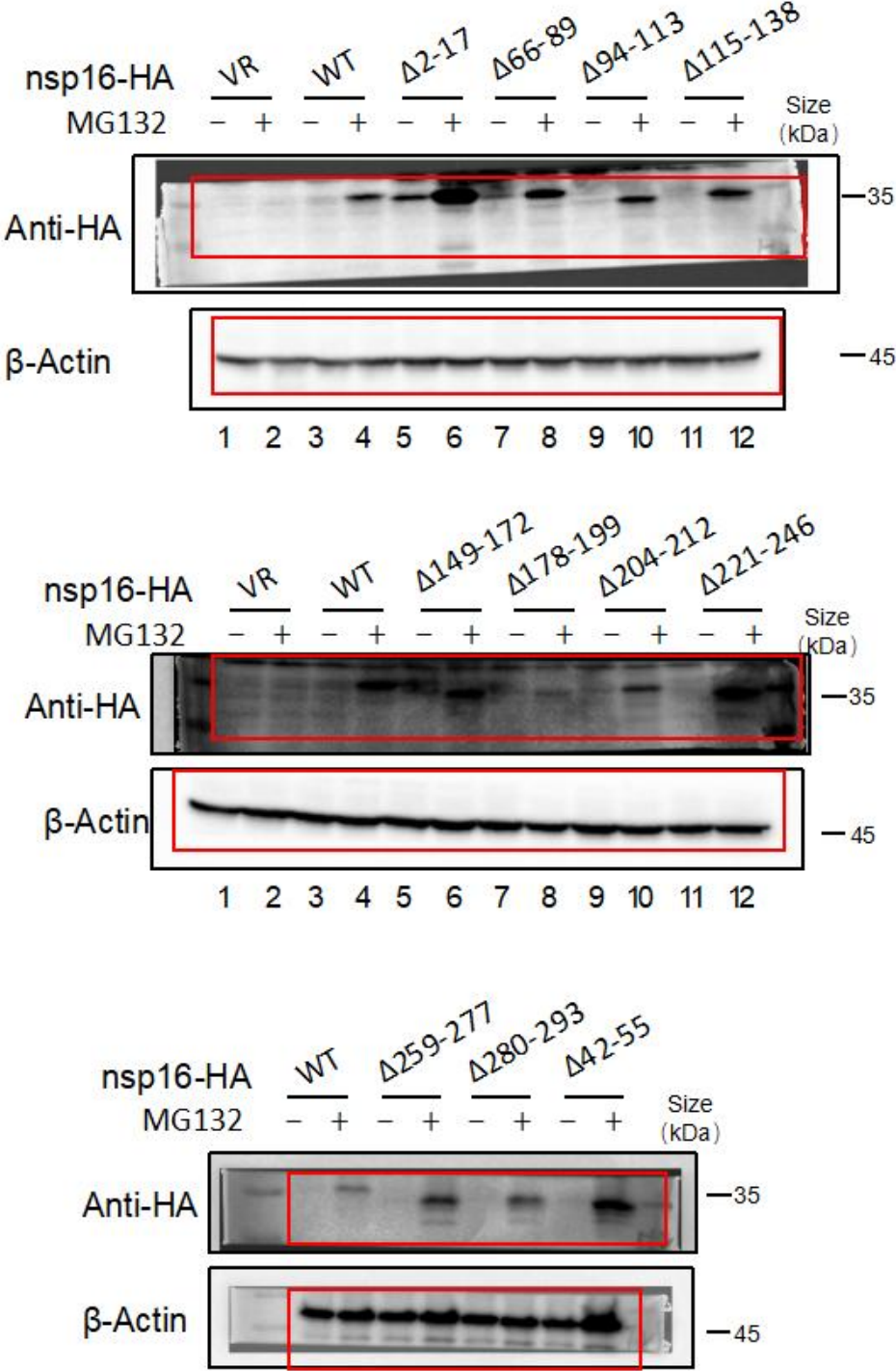

Supplement: Figure 8—figure supplement 1—source data 1. [file elife-102277-fig8-figsupp1-data1.zip › Figure 8—figure supplement 1-source data 1/Figure 8—figure supplement 1B-source data 1.pdf]

Figure 8—figure supplement 1D

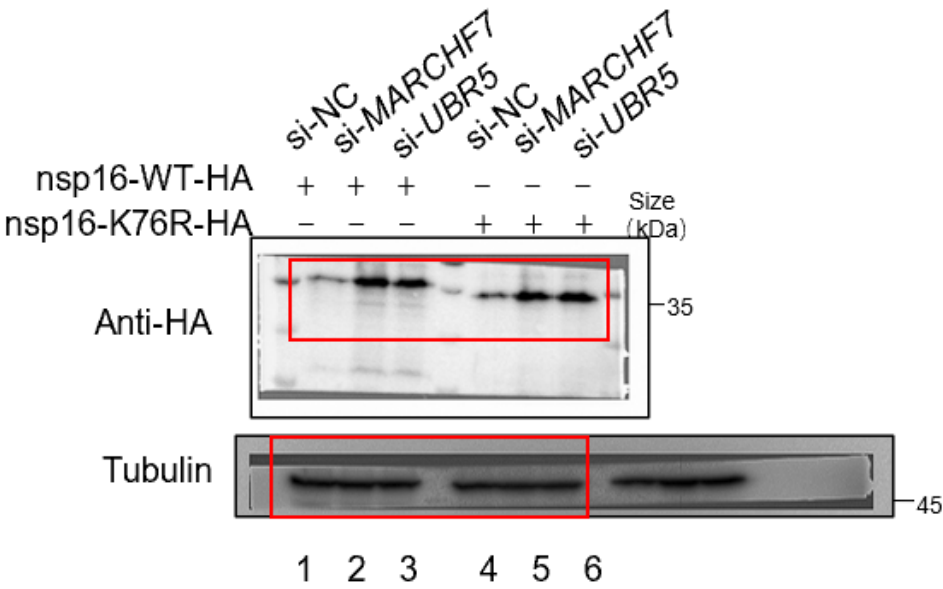

Supplement: Figure 8—figure supplement 1—source data 1. [file elife-102277-fig8-figsupp1-data1.zip › Figure 8—figure supplement 1-source data 1/Figure 8—figure supplement 1D-source data 1.pdf]

Figure 8—figure supplement 1E

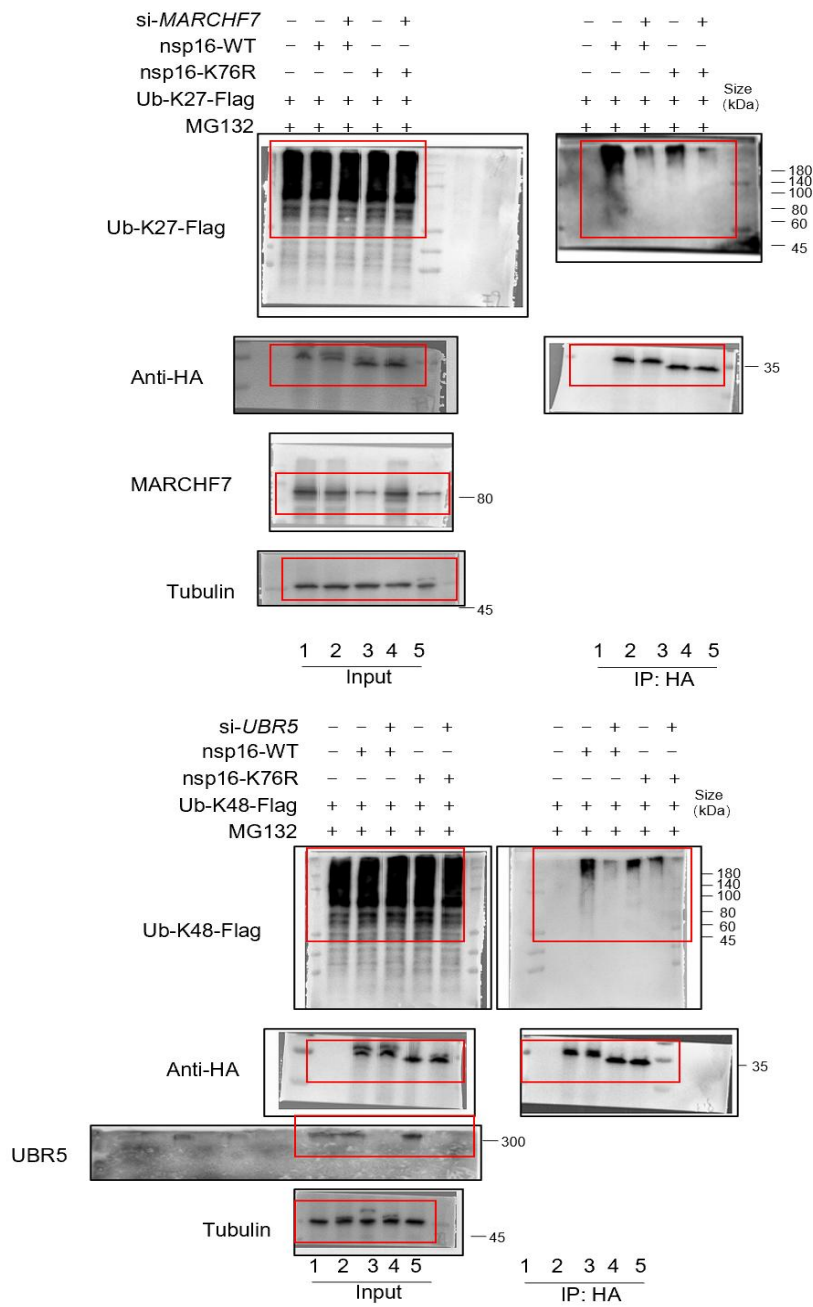

Supplement: Figure 8—figure supplement 1—source data 1. [file elife-102277-fig8-figsupp1-data1.zip › Figure 8—figure supplement 1-source data 1/Figure 8—figure supplement 1E-source data 1.pdf]

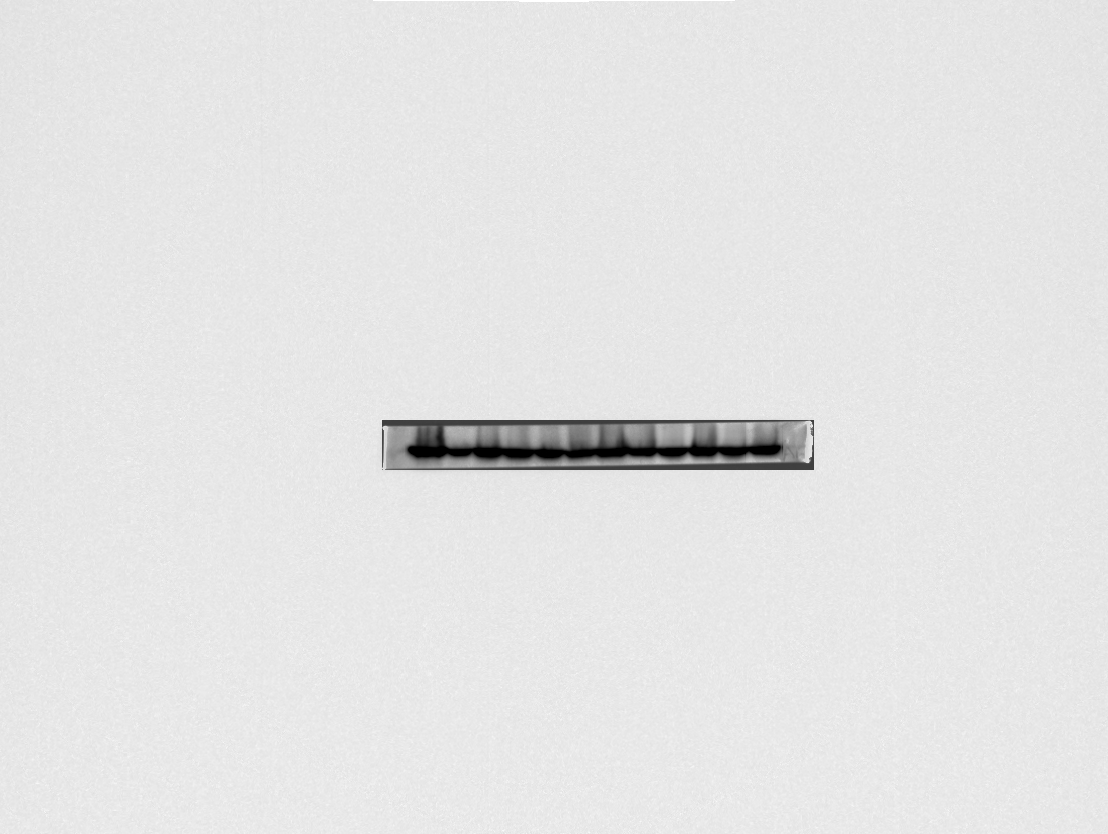

Supplement: Figure 8—figure supplement 1—source data 2. [file elife-102277-fig8-figsupp1-data2.zip › Figure 8—figure supplement 1-source data 2/Figure 8—figure supplement 1A-source data 2/ACTIN-1.tif]

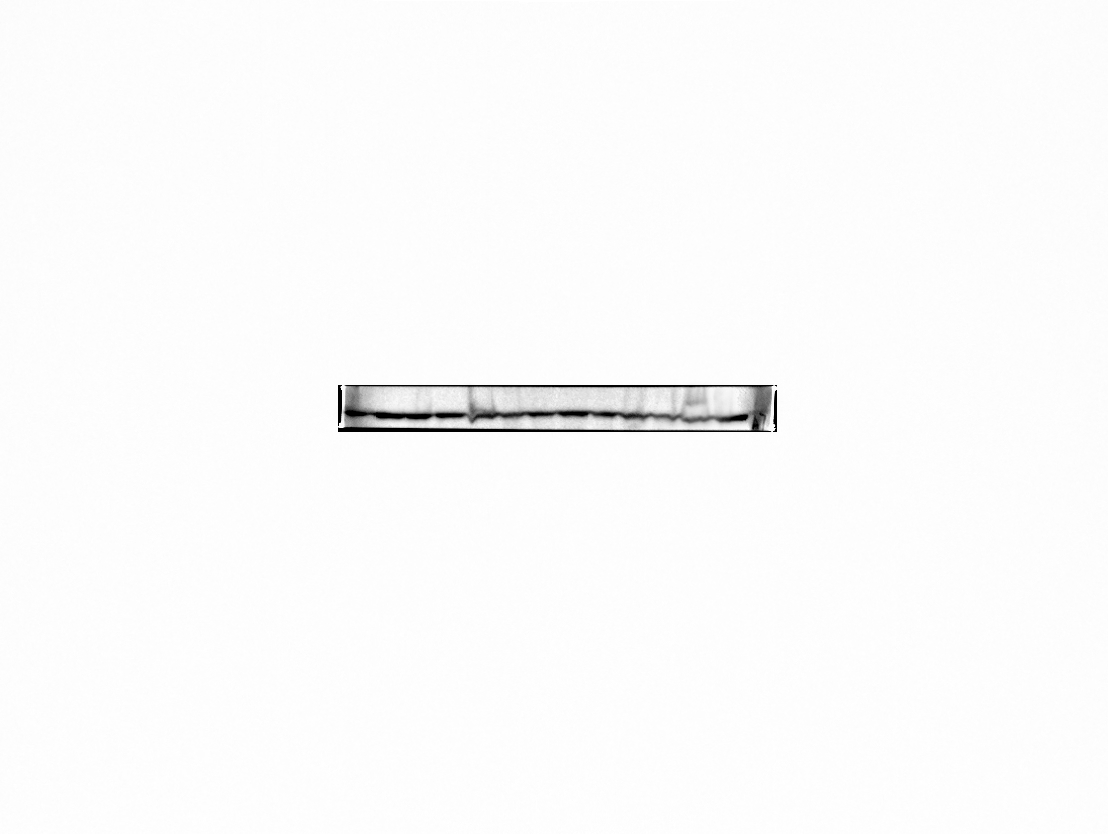

Supplement: Figure 8—figure supplement 1—source data 2. [file elife-102277-fig8-figsupp1-data2.zip › Figure 8—figure supplement 1-source data 2/Figure 8—figure supplement 1A-source data 2/ACTIN-2.tif]

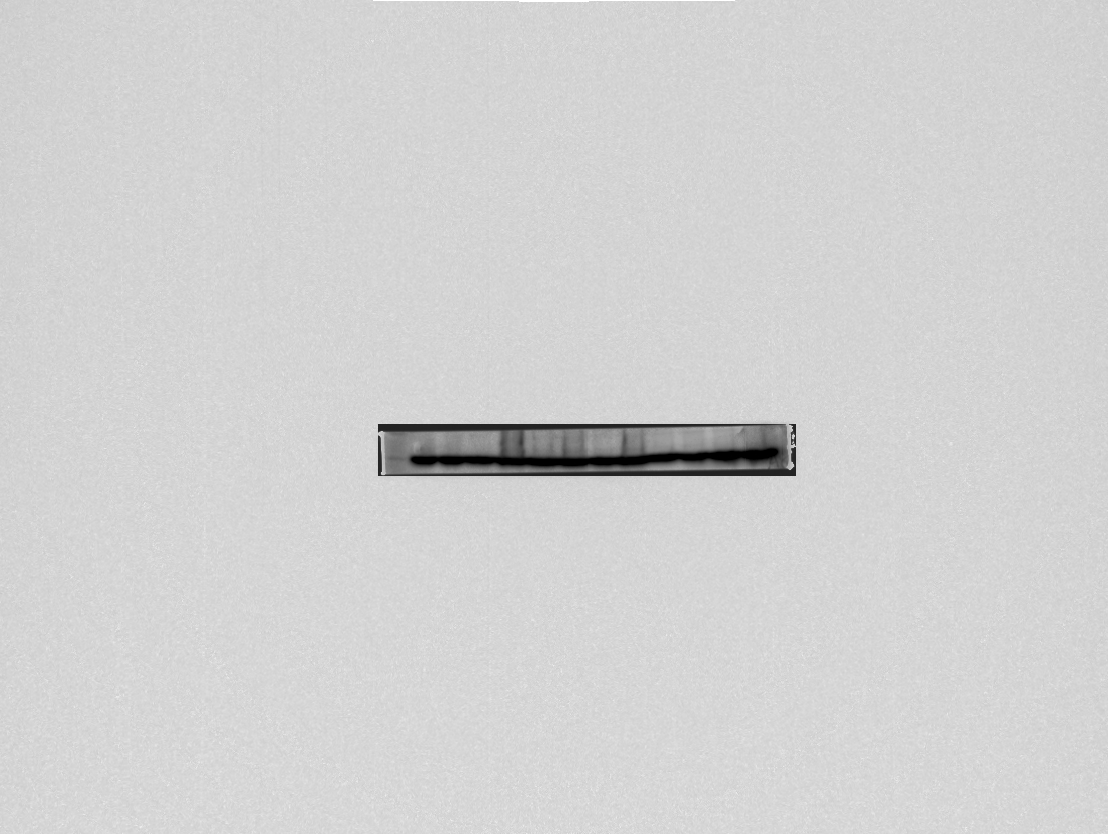

Supplement: Figure 8—figure supplement 1—source data 2. [file elife-102277-fig8-figsupp1-data2.zip › Figure 8—figure supplement 1-source data 2/Figure 8—figure supplement 1A-source data 2/ACTIN-3.tif]

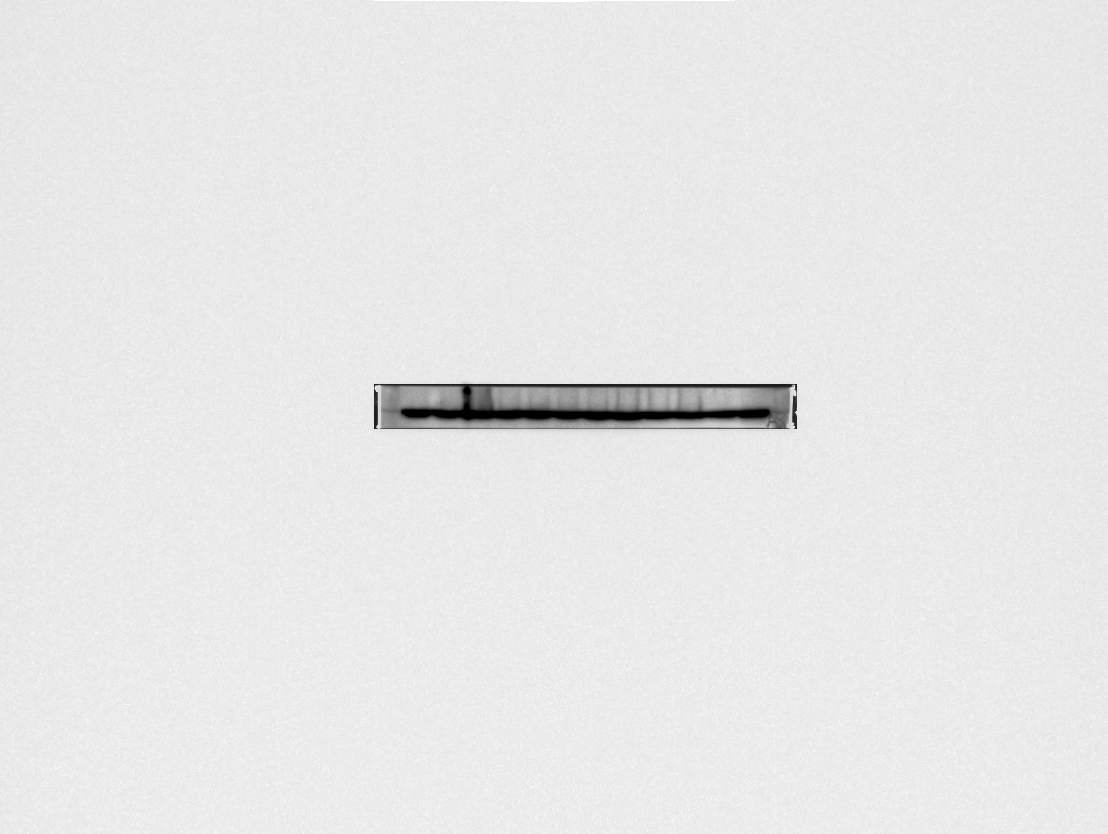

Supplement: Figure 8—figure supplement 1—source data 2. [file elife-102277-fig8-figsupp1-data2.zip › Figure 8—figure supplement 1-source data 2/Figure 8—figure supplement 1A-source data 2/ACTIN-4.tif]

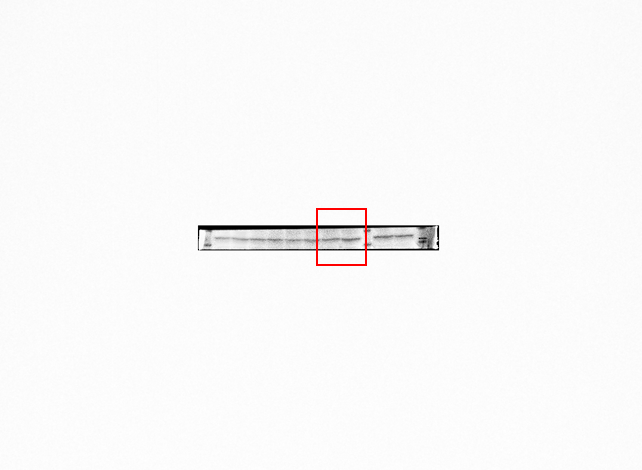

Supplement: Figure 8—figure supplement 1—source data 2. [file elife-102277-fig8-figsupp1-data2.zip › Figure 8—figure supplement 1-source data 2/Figure 8—figure supplement 1A-source data 2/ACTIN-5-1.tif]

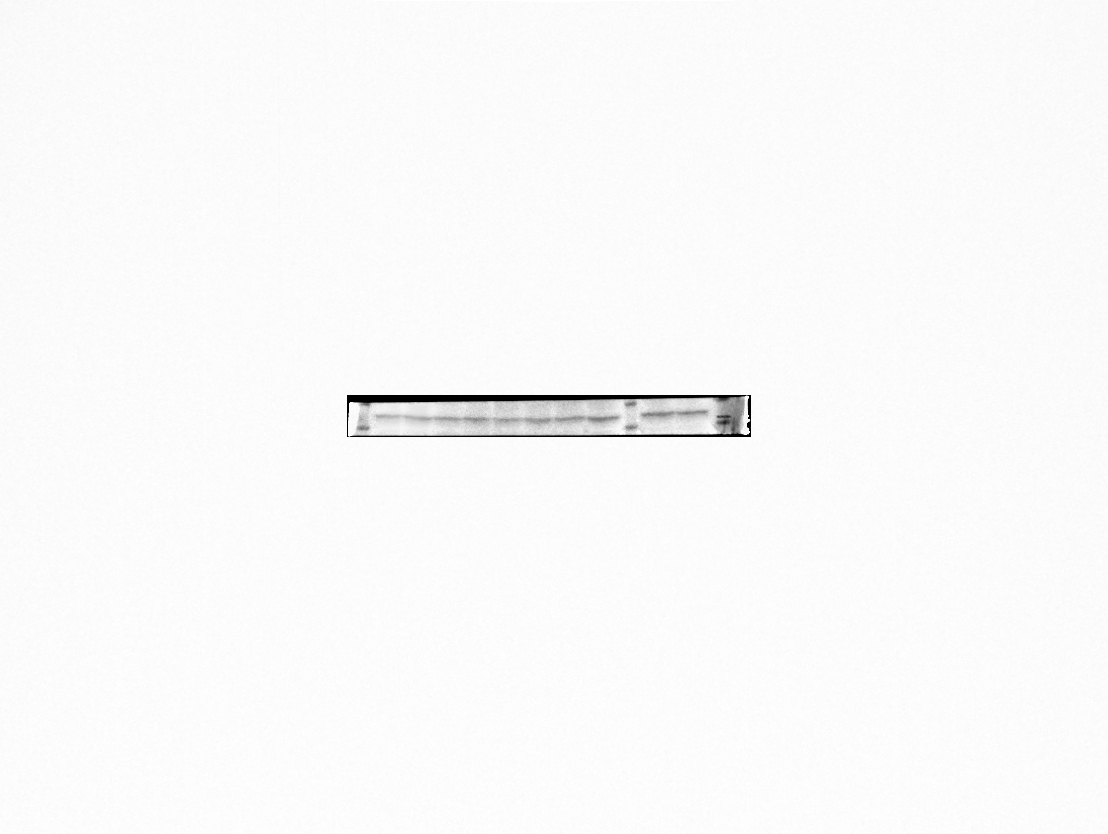

Supplement: Figure 8—figure supplement 1—source data 2. [file elife-102277-fig8-figsupp1-data2.zip › Figure 8—figure supplement 1-source data 2/Figure 8—figure supplement 1A-source data 2/ACTIN-5.tif]

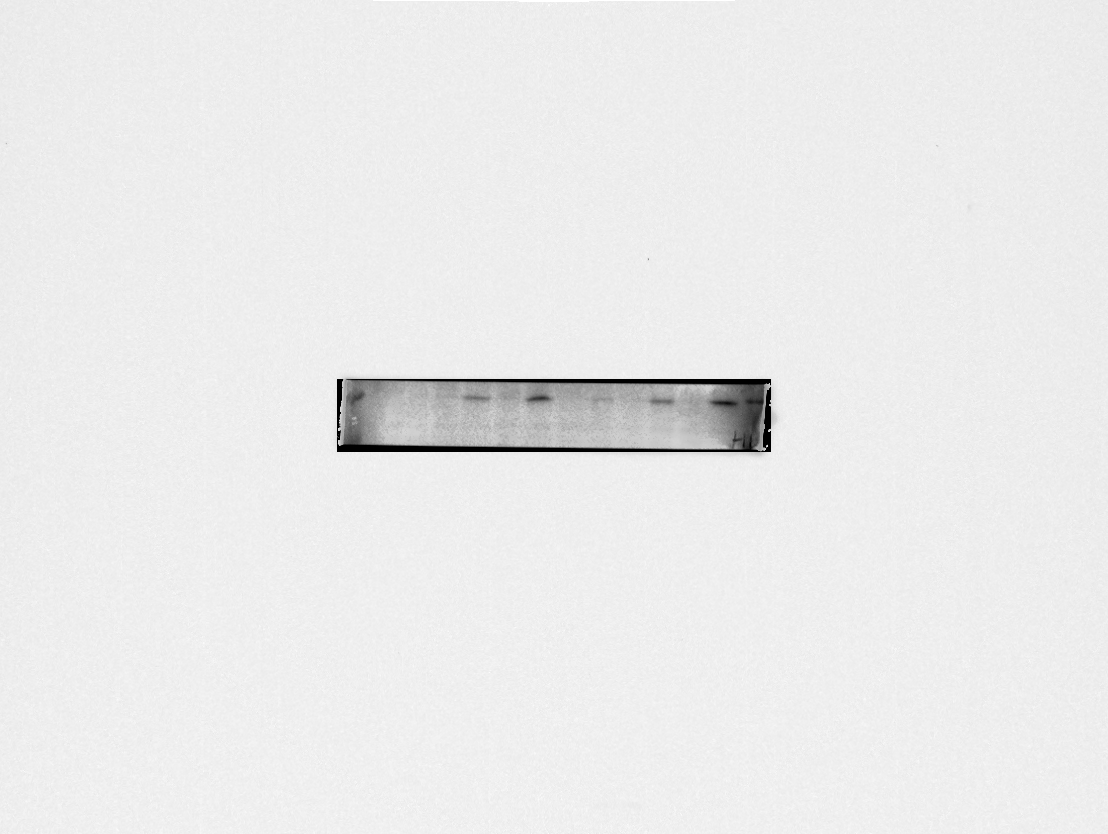

Supplement: Figure 8—figure supplement 1—source data 2. [file elife-102277-fig8-figsupp1-data2.zip › Figure 8—figure supplement 1-source data 2/Figure 8—figure supplement 1A-source data 2/nsp16-1.tif]

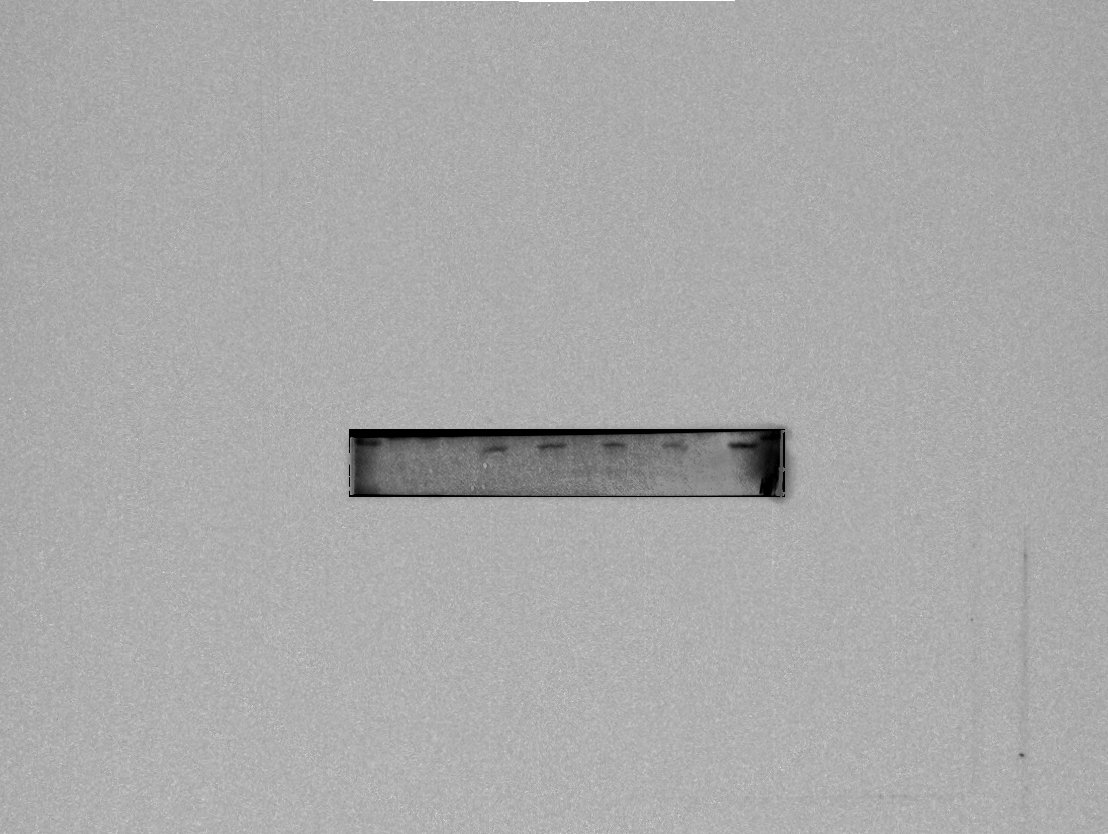

Supplement: Figure 8—figure supplement 1—source data 2. [file elife-102277-fig8-figsupp1-data2.zip › Figure 8—figure supplement 1-source data 2/Figure 8—figure supplement 1A-source data 2/nsp16-2.tif]

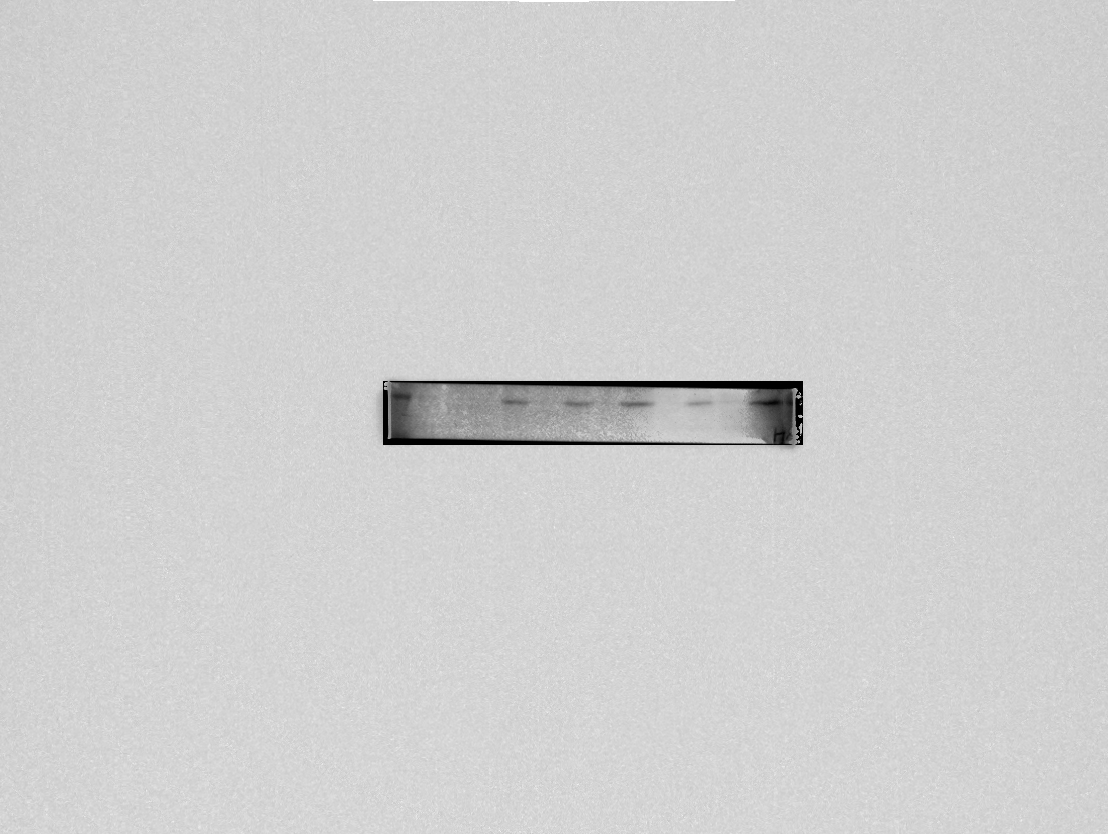

Supplement: Figure 8—figure supplement 1—source data 2. [file elife-102277-fig8-figsupp1-data2.zip › Figure 8—figure supplement 1-source data 2/Figure 8—figure supplement 1A-source data 2/nsp16-3.tif]

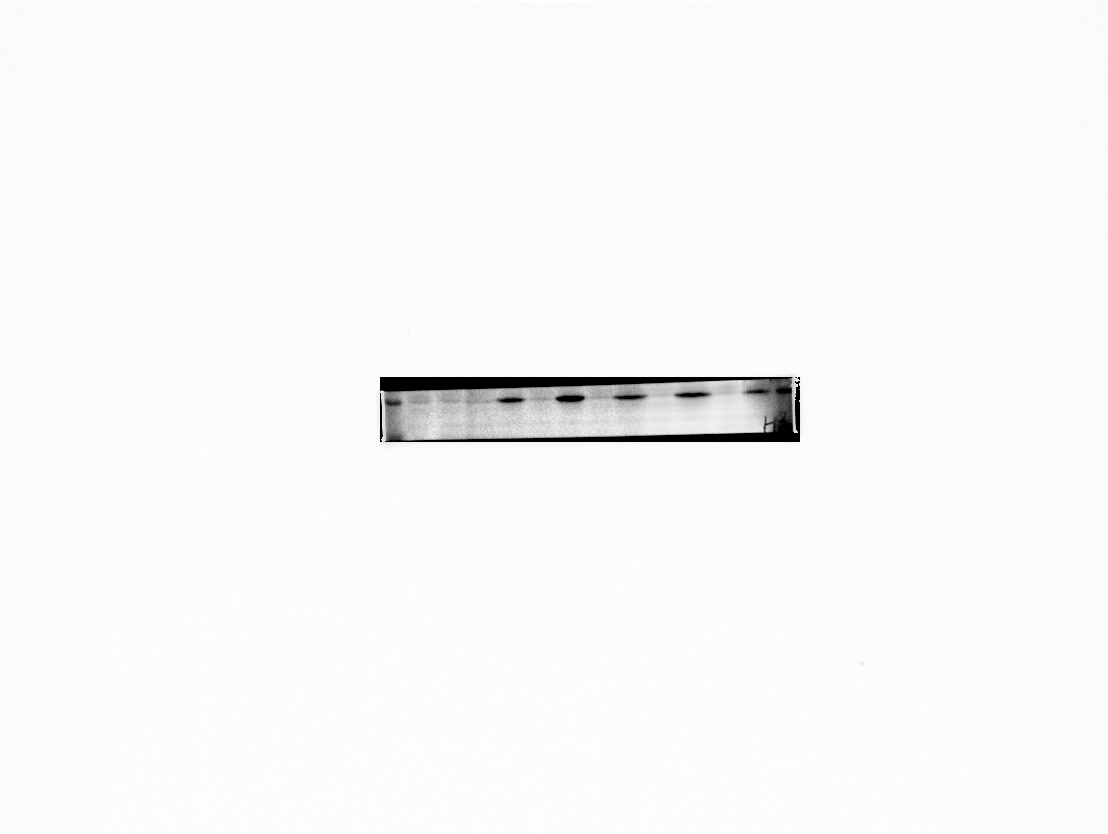

Supplement: Figure 8—figure supplement 1—source data 2. [file elife-102277-fig8-figsupp1-data2.zip › Figure 8—figure supplement 1-source data 2/Figure 8—figure supplement 1A-source data 2/nsp16-4.tif]

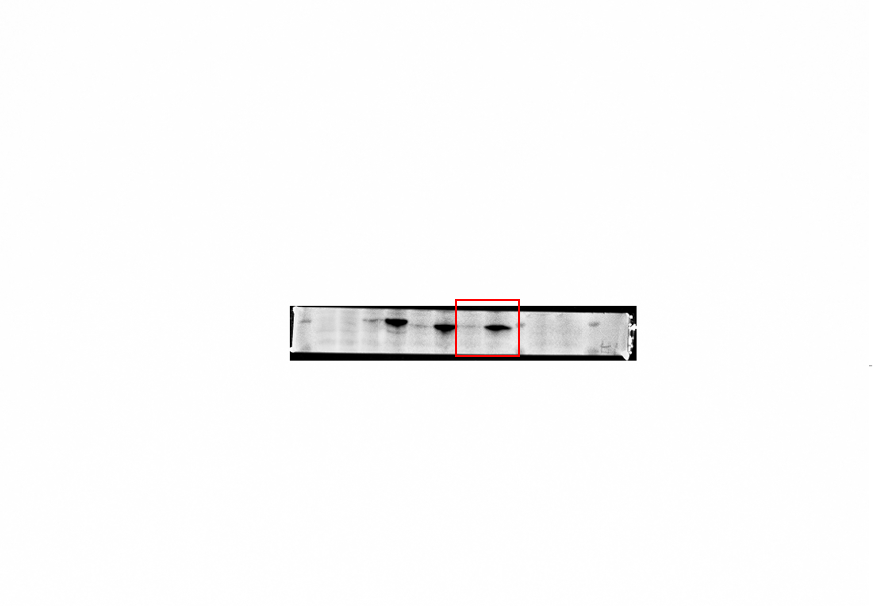

Supplement: Figure 8—figure supplement 1—source data 2. [file elife-102277-fig8-figsupp1-data2.zip › Figure 8—figure supplement 1-source data 2/Figure 8—figure supplement 1A-source data 2/nsp16-5-1.tif]

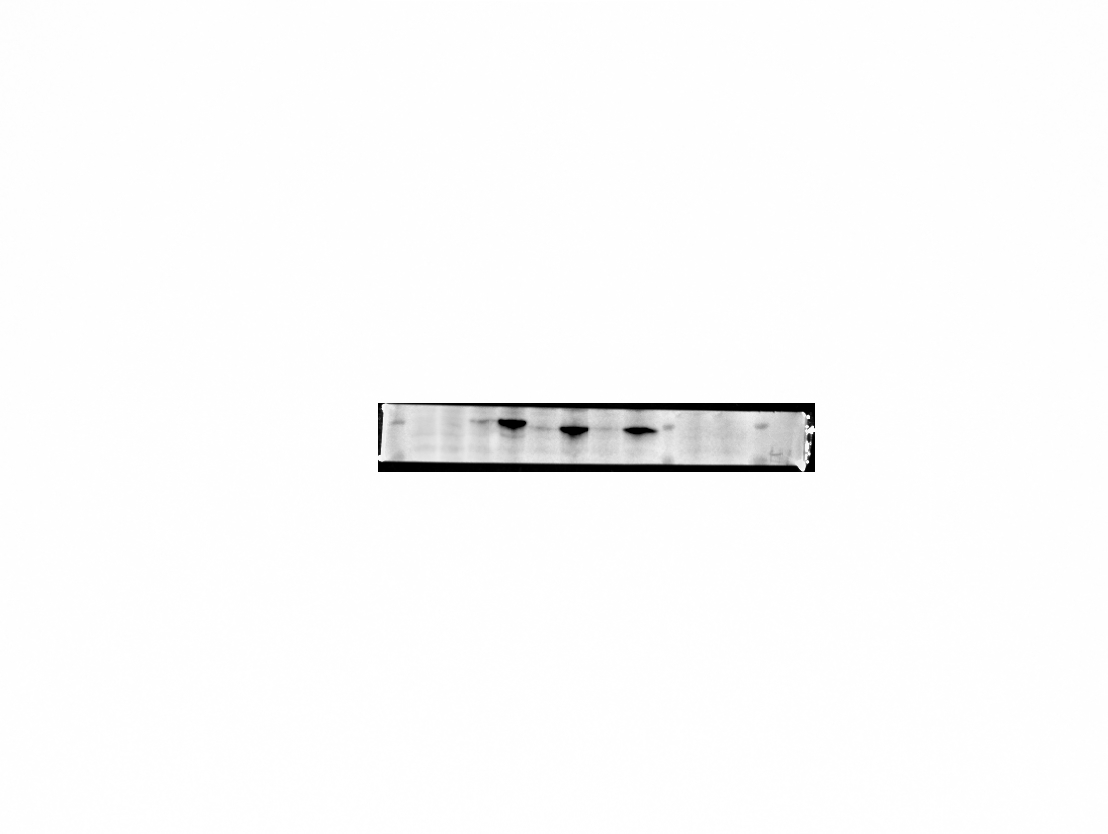

Supplement: Figure 8—figure supplement 1—source data 2. [file elife-102277-fig8-figsupp1-data2.zip › Figure 8—figure supplement 1-source data 2/Figure 8—figure supplement 1A-source data 2/nsp16-5.tif]

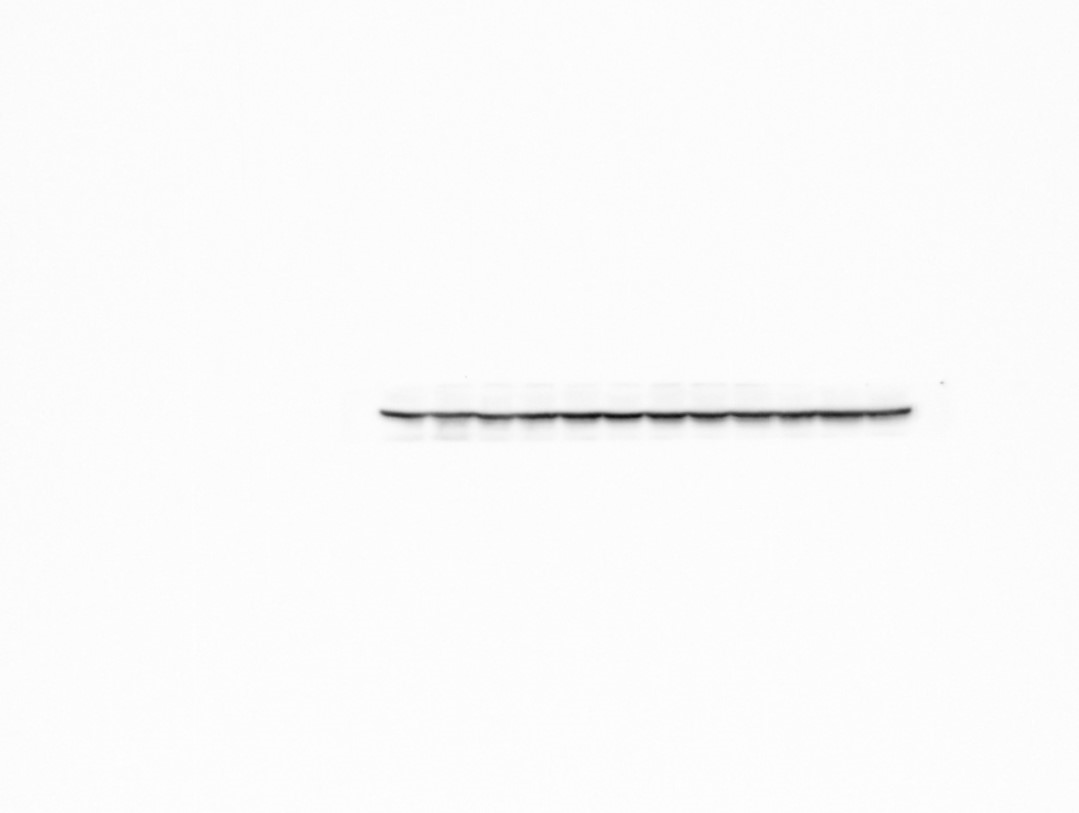

Supplement: Figure 8—figure supplement 1—source data 2. [file elife-102277-fig8-figsupp1-data2.zip › Figure 8—figure supplement 1-source data 2/Figure 8—figure supplement 1B-source data 2/Actin-1.tif]

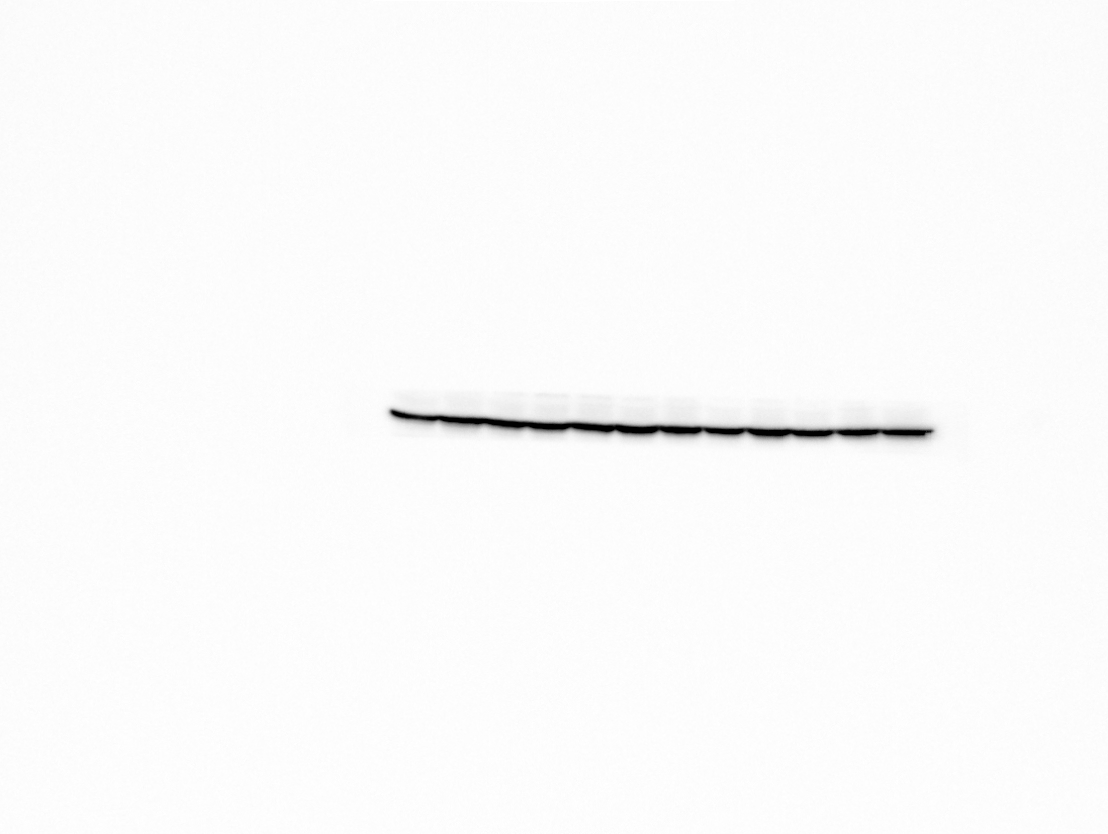

Supplement: Figure 8—figure supplement 1—source data 2. [file elife-102277-fig8-figsupp1-data2.zip › Figure 8—figure supplement 1-source data 2/Figure 8—figure supplement 1B-source data 2/Actin-2.tif]

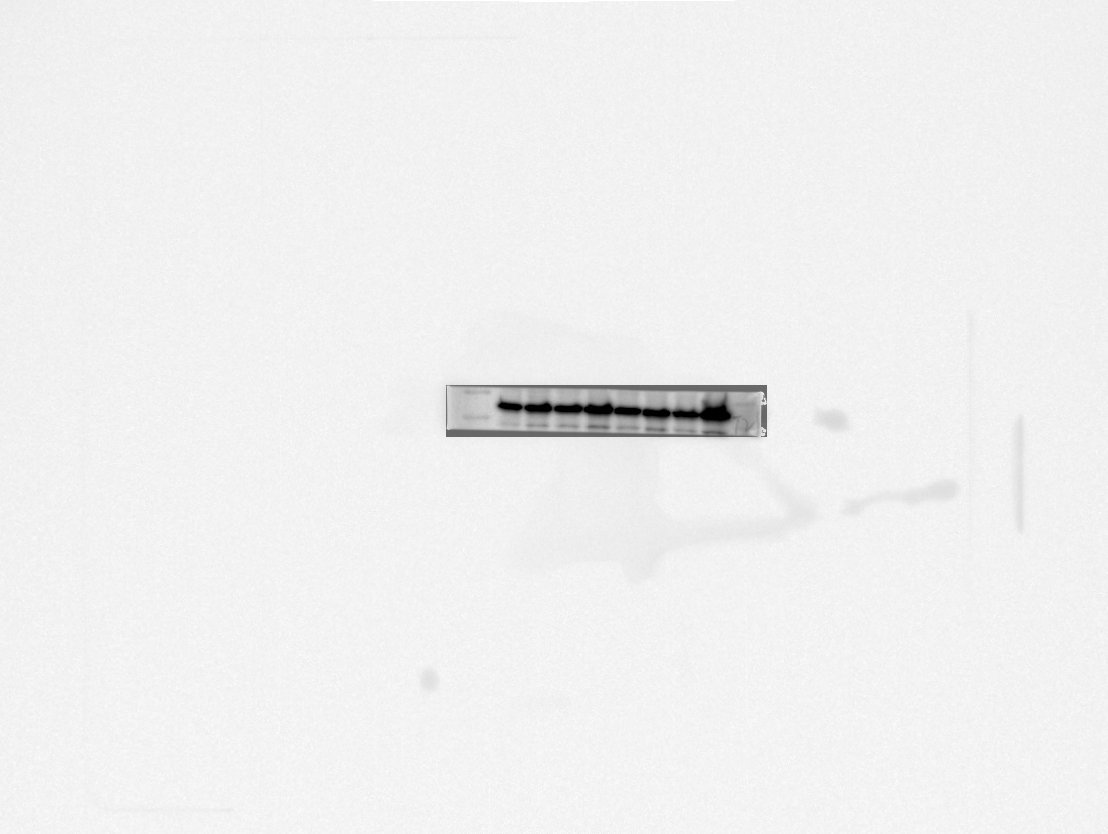

Supplement: Figure 8—figure supplement 1—source data 2. [file elife-102277-fig8-figsupp1-data2.zip › Figure 8—figure supplement 1-source data 2/Figure 8—figure supplement 1B-source data 2/Actin-3.tif]

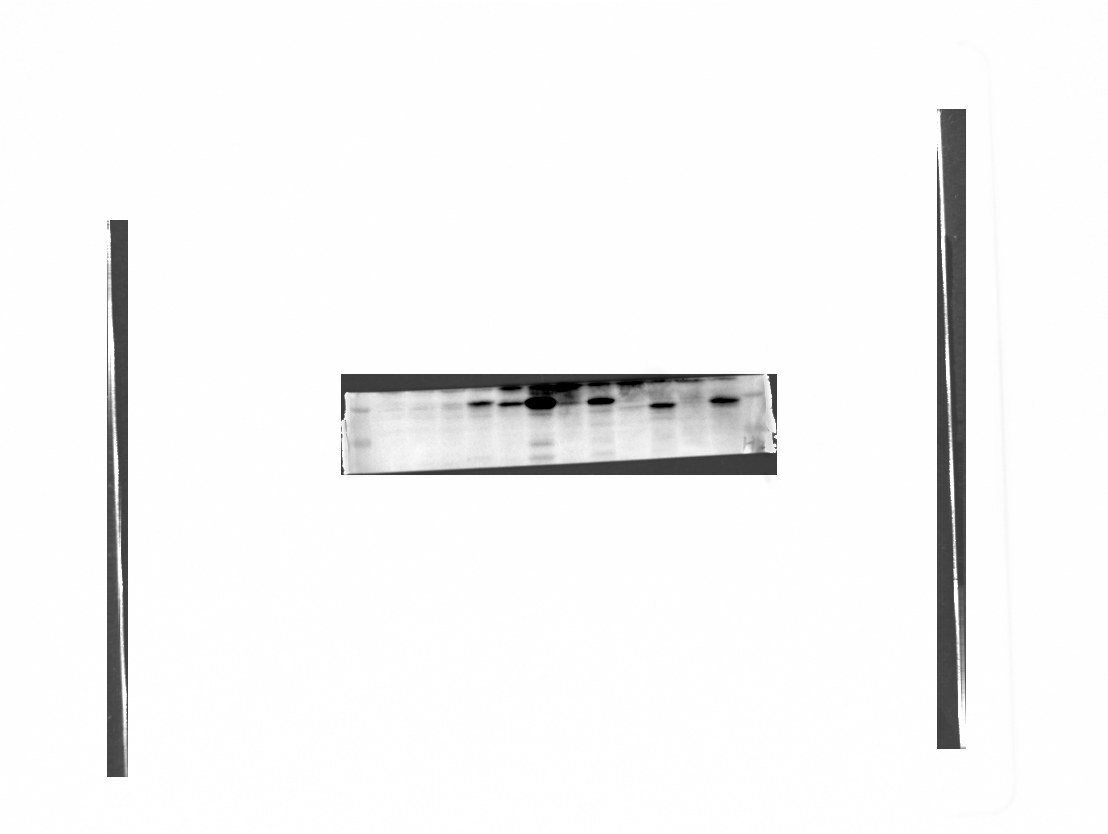

Supplement: Figure 8—figure supplement 1—source data 2. [file elife-102277-fig8-figsupp1-data2.zip › Figure 8—figure supplement 1-source data 2/Figure 8—figure supplement 1B-source data 2/nsp16-1.tif]

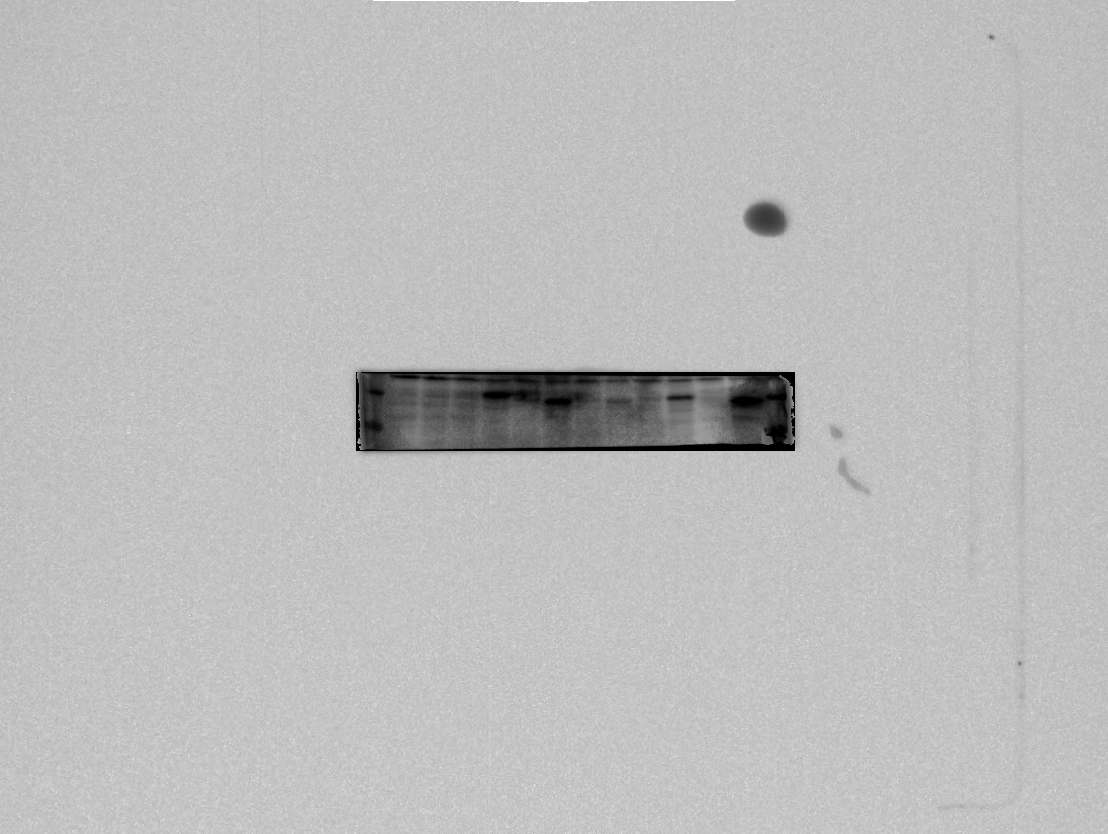

Supplement: Figure 8—figure supplement 1—source data 2. [file elife-102277-fig8-figsupp1-data2.zip › Figure 8—figure supplement 1-source data 2/Figure 8—figure supplement 1B-source data 2/nsp16-2.tif]

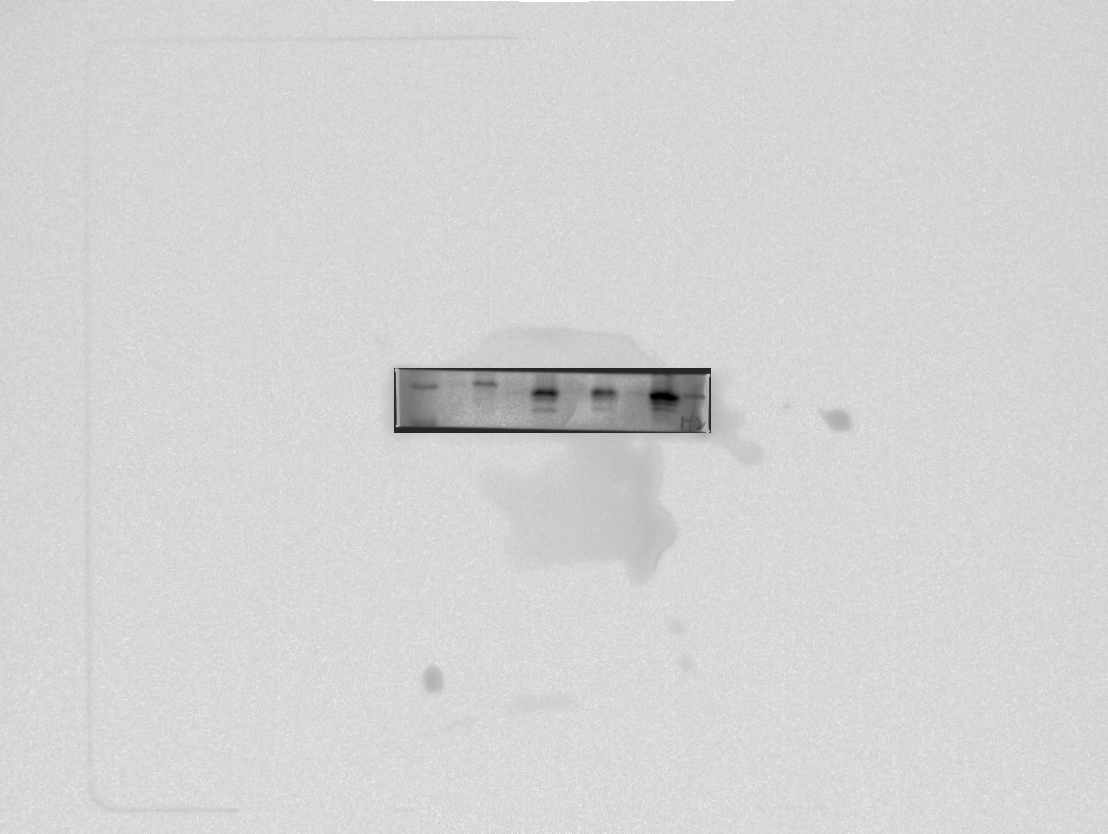

Supplement: Figure 8—figure supplement 1—source data 2. [file elife-102277-fig8-figsupp1-data2.zip › Figure 8—figure supplement 1-source data 2/Figure 8—figure supplement 1B-source data 2/nsp16-3.tif]

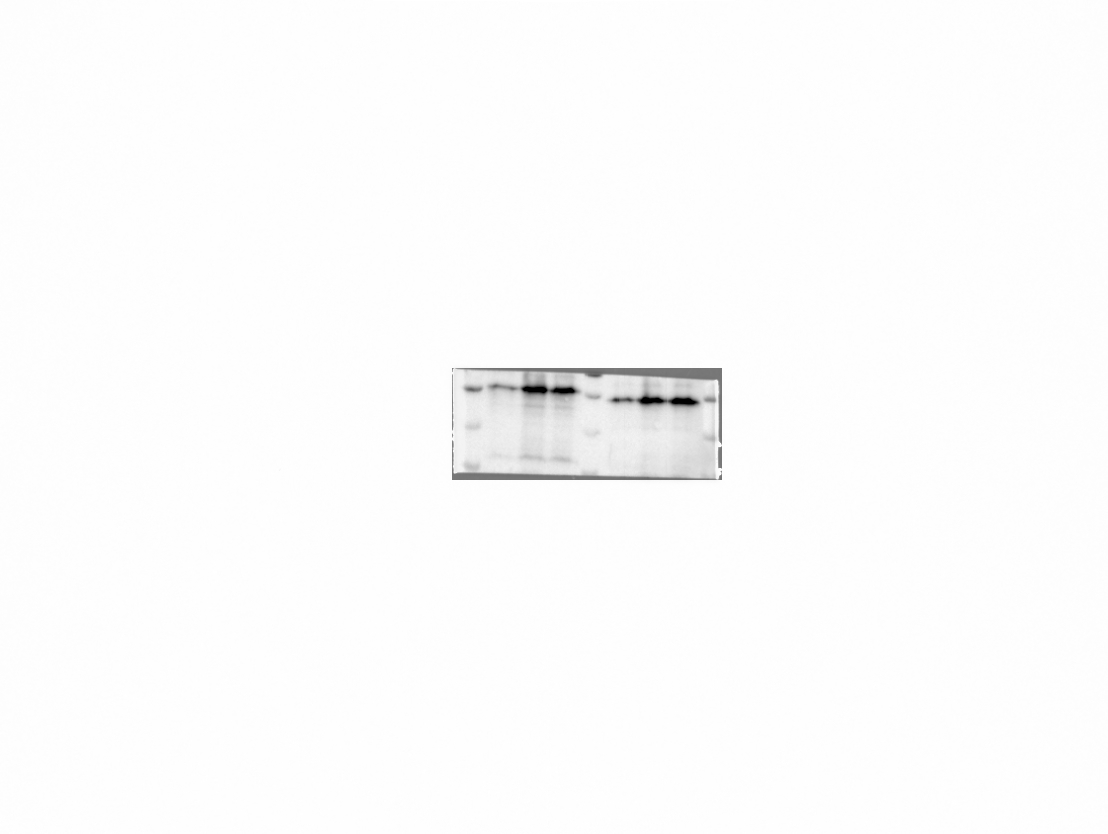

Supplement: Figure 8—figure supplement 1—source data 2. [file elife-102277-fig8-figsupp1-data2.zip › Figure 8—figure supplement 1-source data 2/Figure 8—figure supplement 1D-source data 2/nsp16.tif]

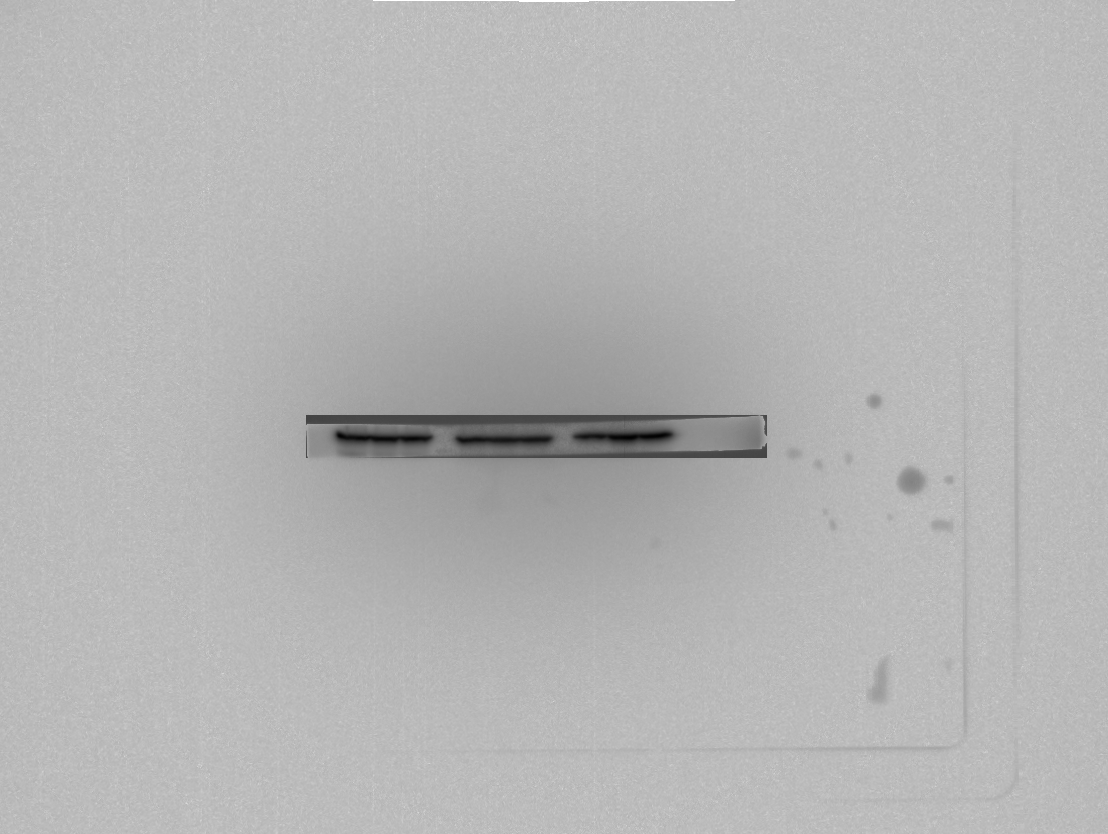

Supplement: Figure 8—figure supplement 1—source data 2. [file elife-102277-fig8-figsupp1-data2.zip › Figure 8—figure supplement 1-source data 2/Figure 8—figure supplement 1D-source data 2/Tubulin.tif]

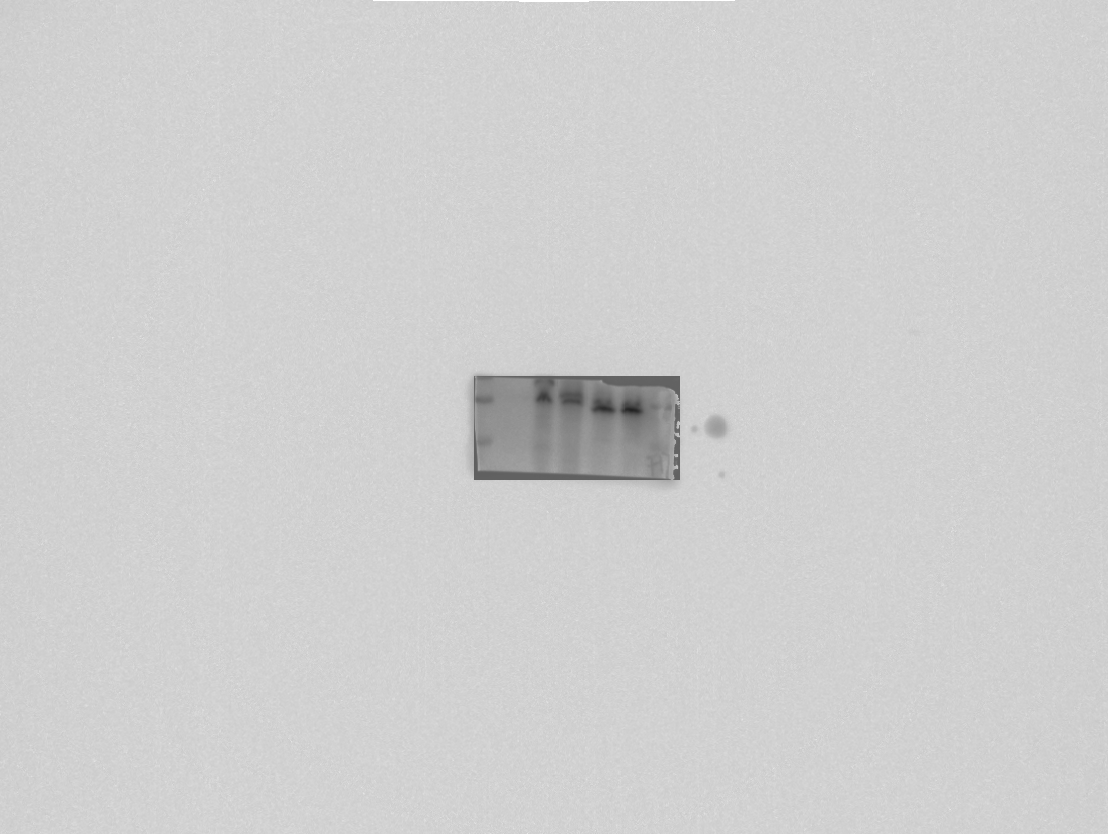

Supplement: Figure 8—figure supplement 1—source data 2. [file elife-102277-fig8-figsupp1-data2.zip › Figure 8—figure supplement 1-source data 2/Figure 8—figure supplement 1E-source data 2/HA-1.tif]

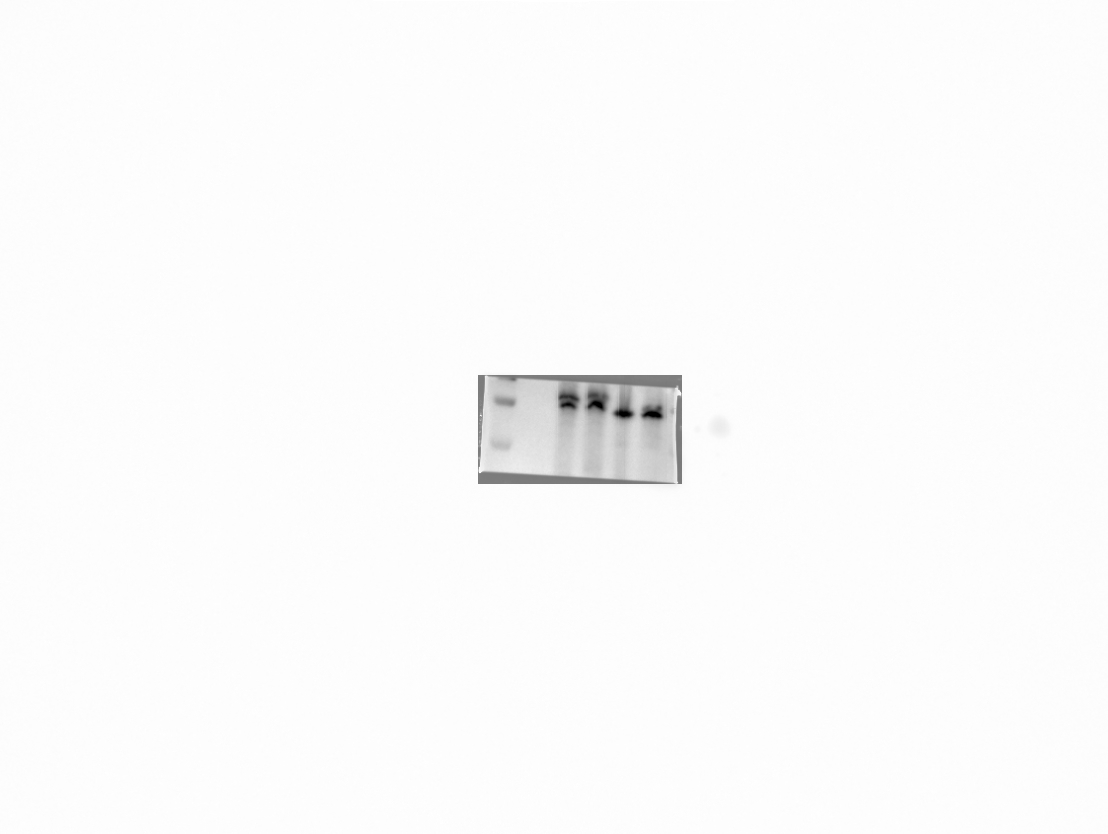

Supplement: Figure 8—figure supplement 1—source data 2. [file elife-102277-fig8-figsupp1-data2.zip › Figure 8—figure supplement 1-source data 2/Figure 8—figure supplement 1E-source data 2/HA-2.tif]

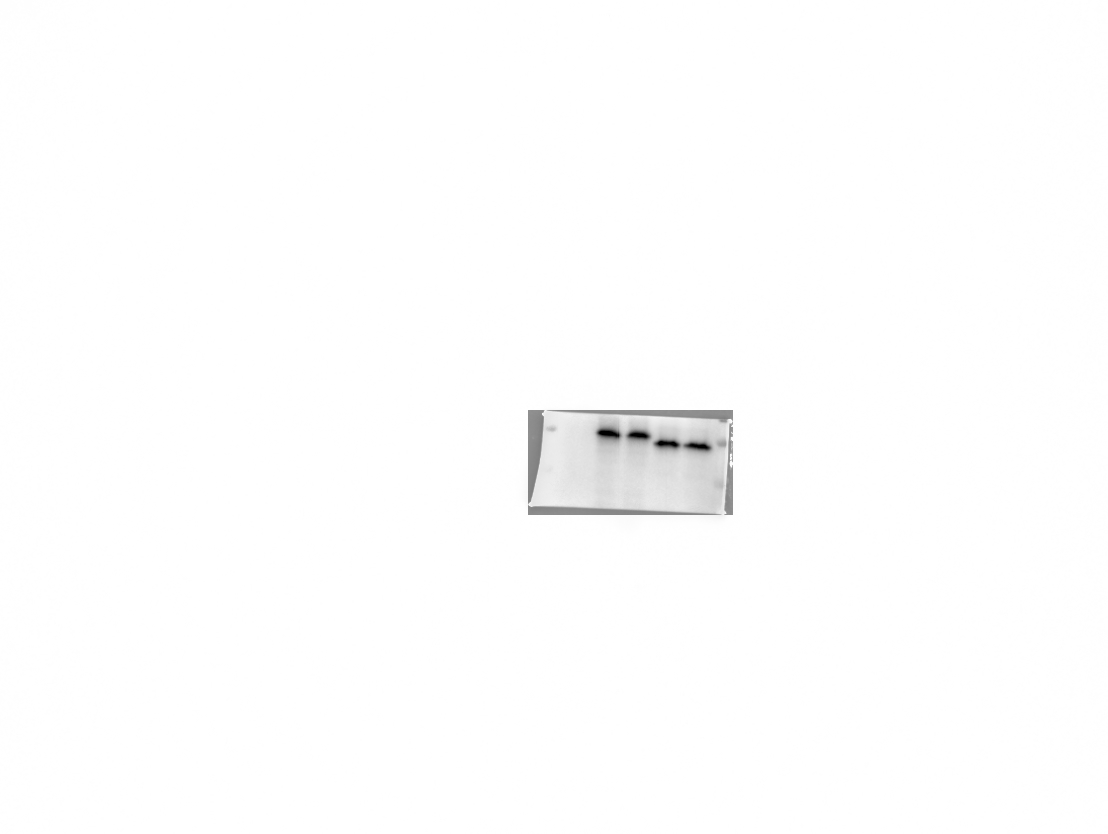

Supplement: Figure 8—figure supplement 1—source data 2. [file elife-102277-fig8-figsupp1-data2.zip › Figure 8—figure supplement 1-source data 2/Figure 8—figure supplement 1E-source data 2/HA-IP-1.tif]

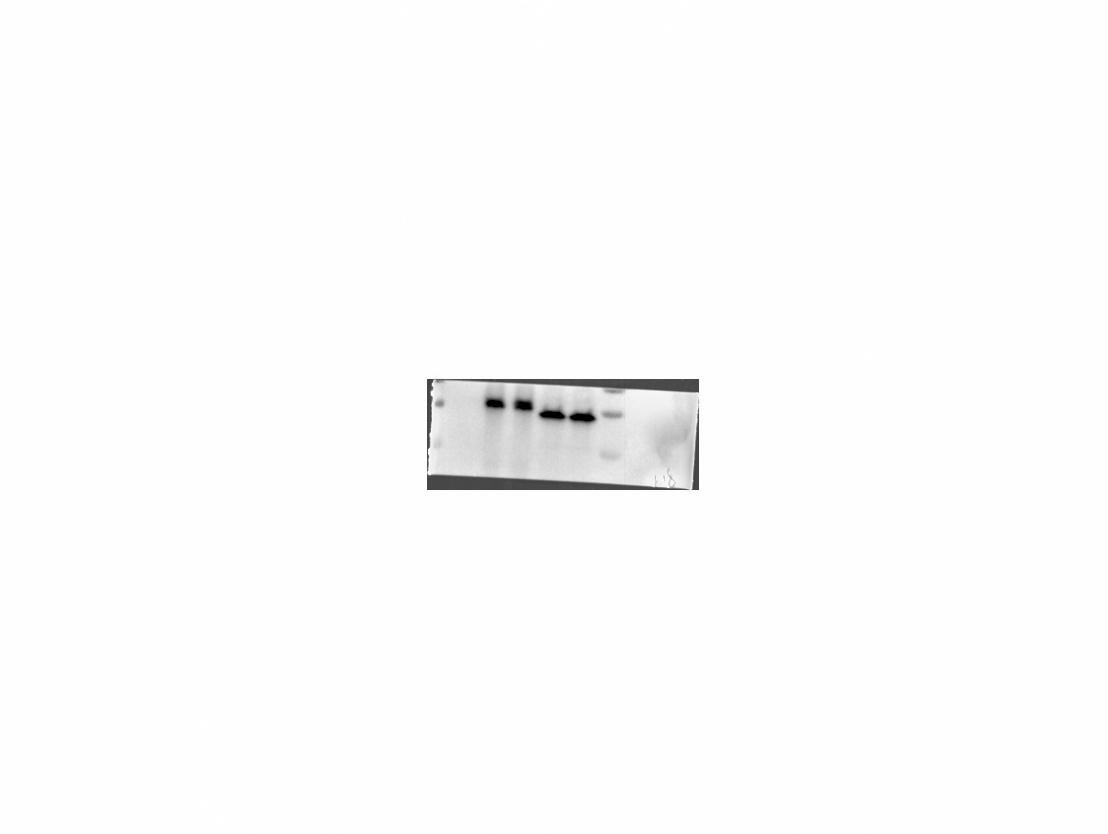

Supplement: Figure 8—figure supplement 1—source data 2. [file elife-102277-fig8-figsupp1-data2.zip › Figure 8—figure supplement 1-source data 2/Figure 8—figure supplement 1E-source data 2/HA-IP-2.tif]

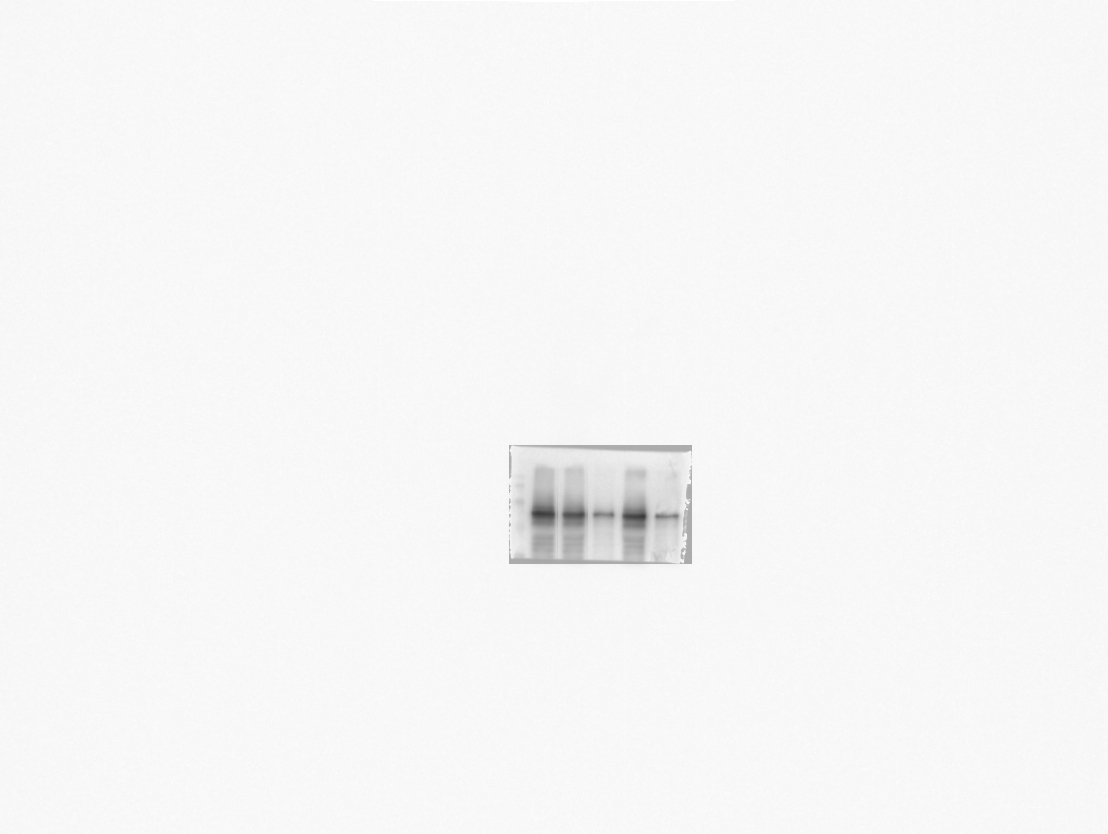

Supplement: Figure 8—figure supplement 1—source data 2. [file elife-102277-fig8-figsupp1-data2.zip › Figure 8—figure supplement 1-source data 2/Figure 8—figure supplement 1E-source data 2/MARCHF7.tif]

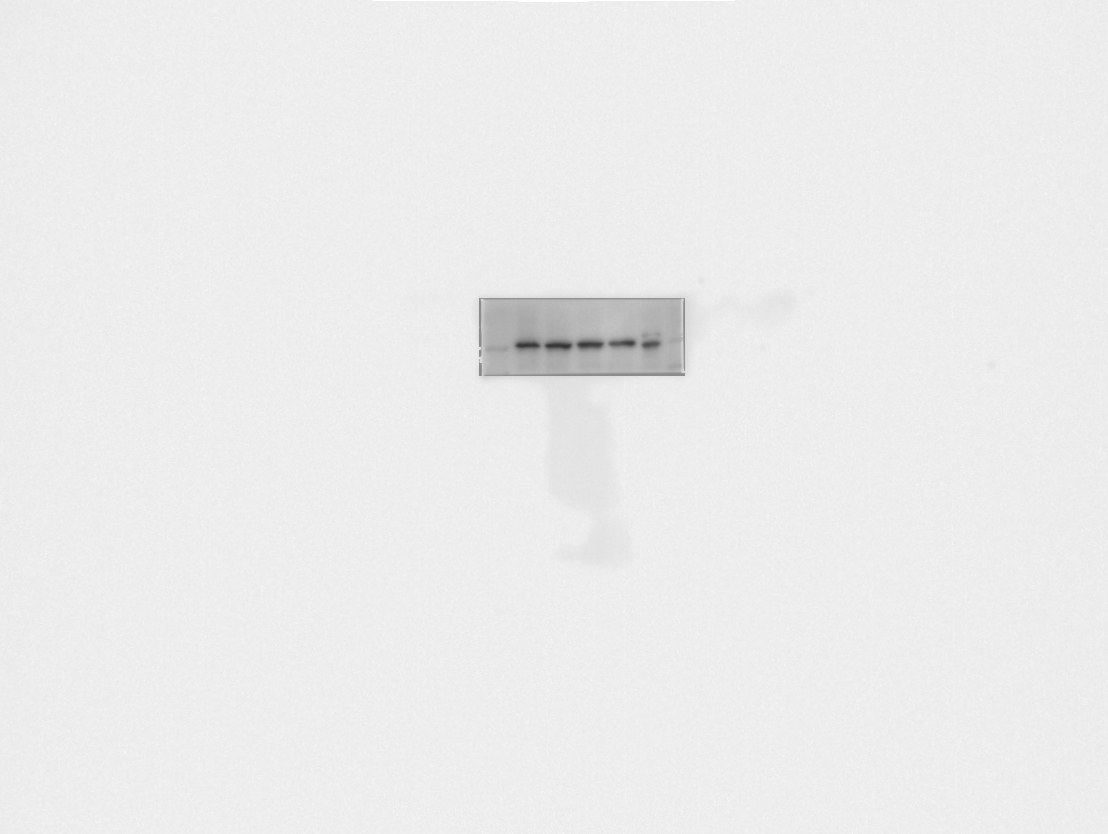

Supplement: Figure 8—figure supplement 1—source data 2. [file elife-102277-fig8-figsupp1-data2.zip › Figure 8—figure supplement 1-source data 2/Figure 8—figure supplement 1E-source data 2/Tubulin-1.tif]

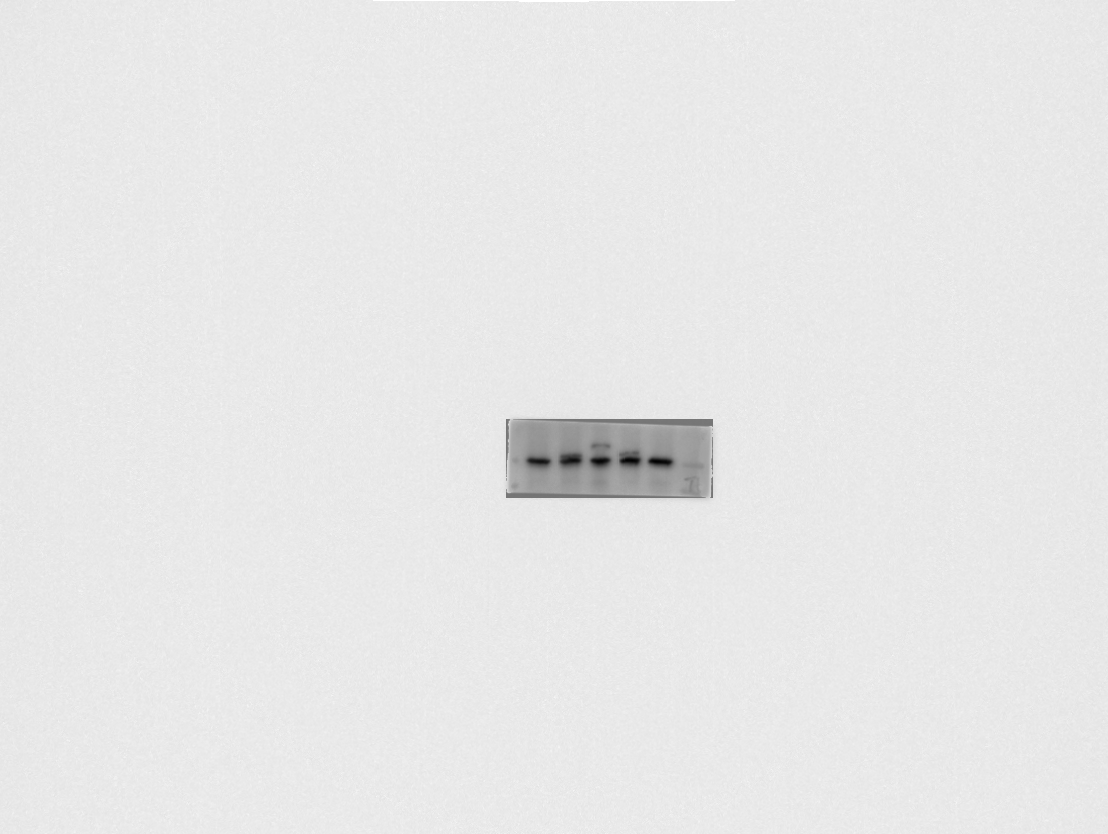

Supplement: Figure 8—figure supplement 1—source data 2. [file elife-102277-fig8-figsupp1-data2.zip › Figure 8—figure supplement 1-source data 2/Figure 8—figure supplement 1E-source data 2/Tubulin-2.tif]

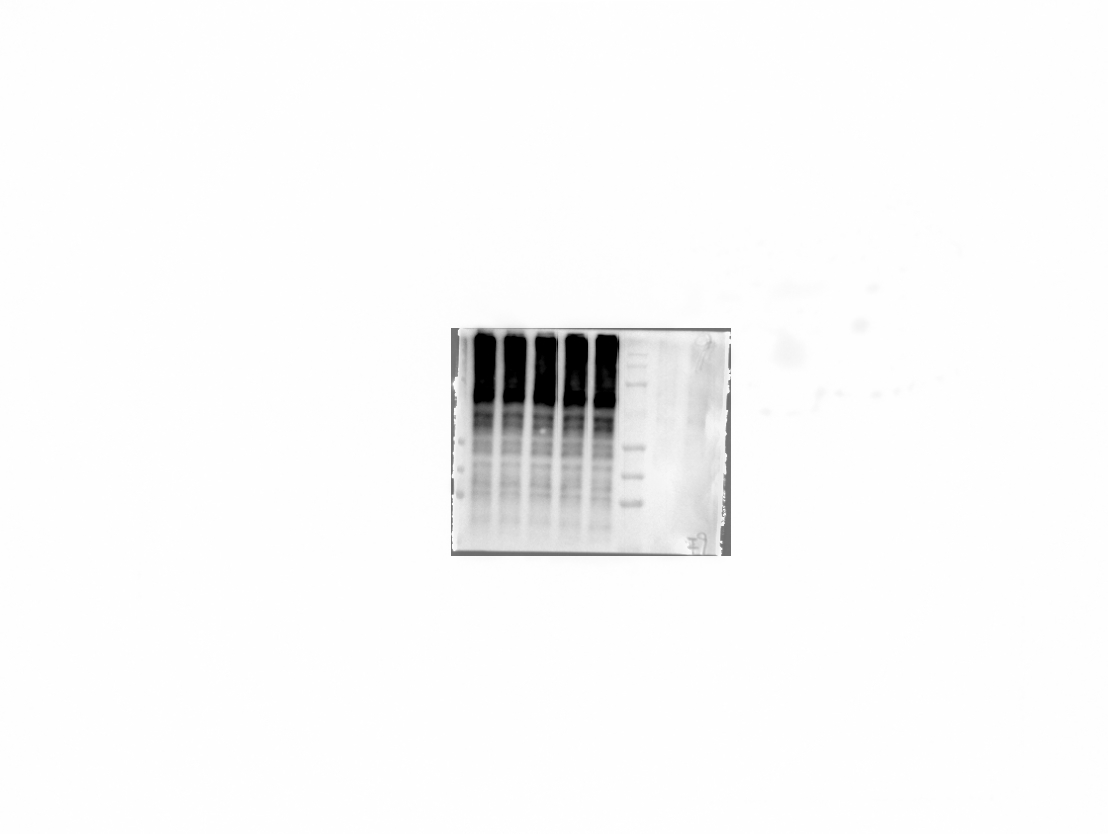

Supplement: Figure 8—figure supplement 1—source data 2. [file elife-102277-fig8-figsupp1-data2.zip › Figure 8—figure supplement 1-source data 2/Figure 8—figure supplement 1E-source data 2/Ub-input-1.tif]

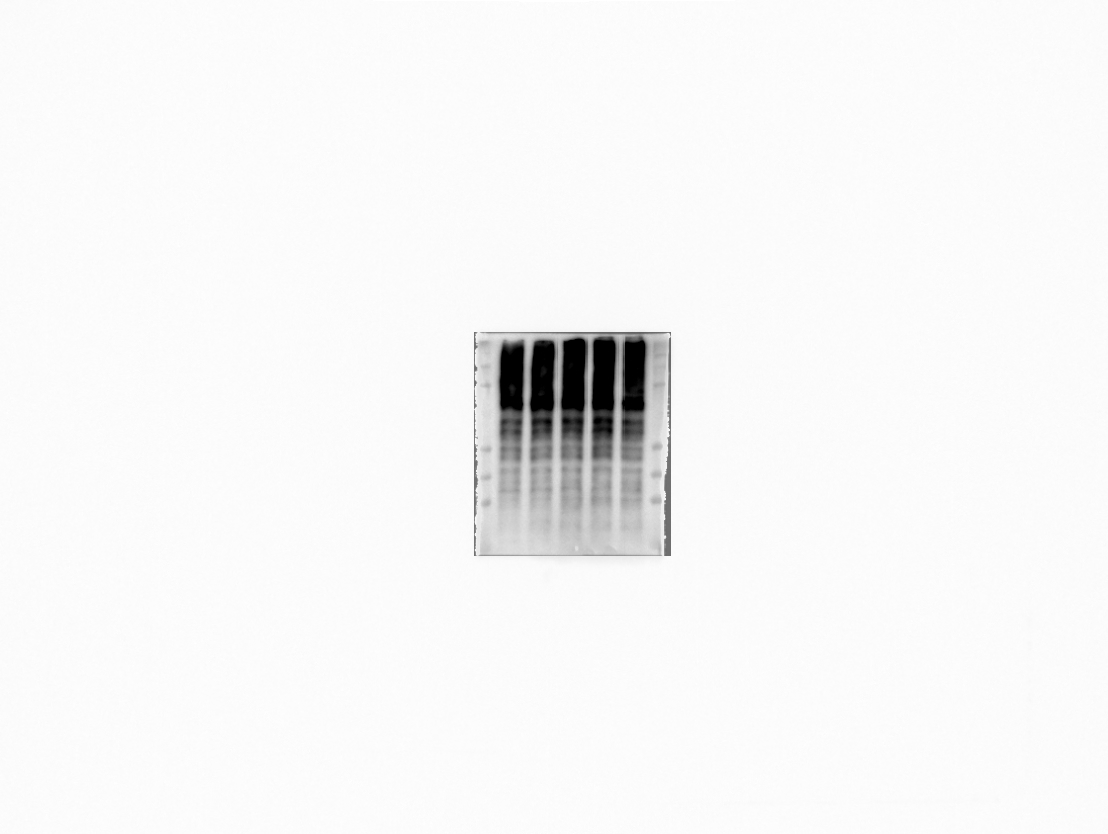

Supplement: Figure 8—figure supplement 1—source data 2. [file elife-102277-fig8-figsupp1-data2.zip › Figure 8—figure supplement 1-source data 2/Figure 8—figure supplement 1E-source data 2/Ub-input-2.tif]

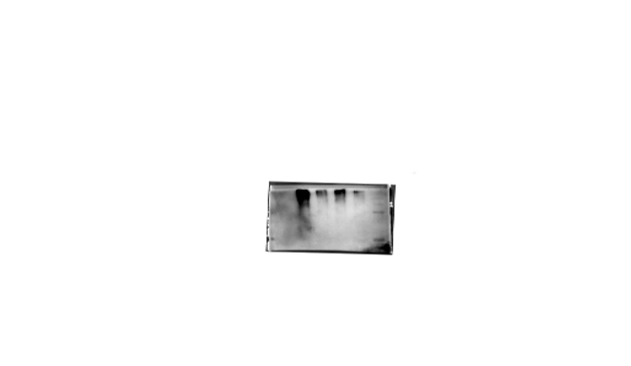

Supplement: Figure 8—figure supplement 1—source data 2. [file elife-102277-fig8-figsupp1-data2.zip › Figure 8—figure supplement 1-source data 2/Figure 8—figure supplement 1E-source data 2/Ub-ip-1.tif]

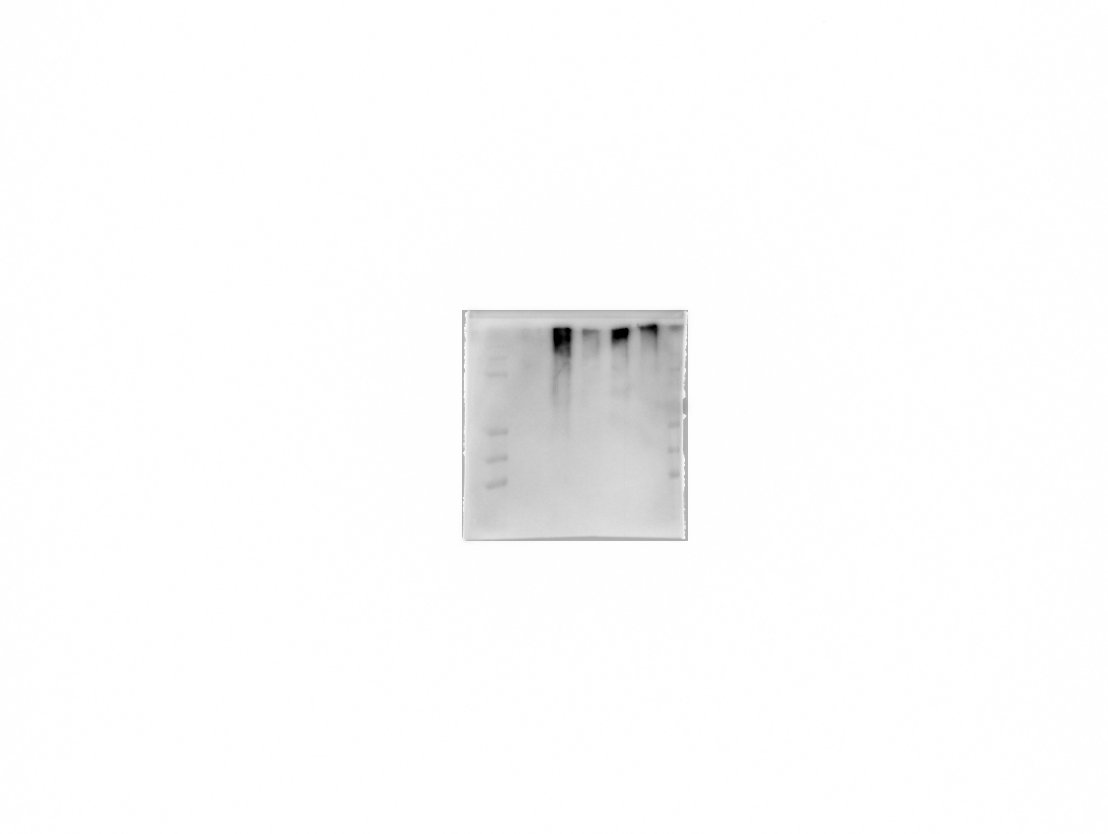

Supplement: Figure 8—figure supplement 1—source data 2. [file elife-102277-fig8-figsupp1-data2.zip › Figure 8—figure supplement 1-source data 2/Figure 8—figure supplement 1E-source data 2/Ub-ip-2.tif]

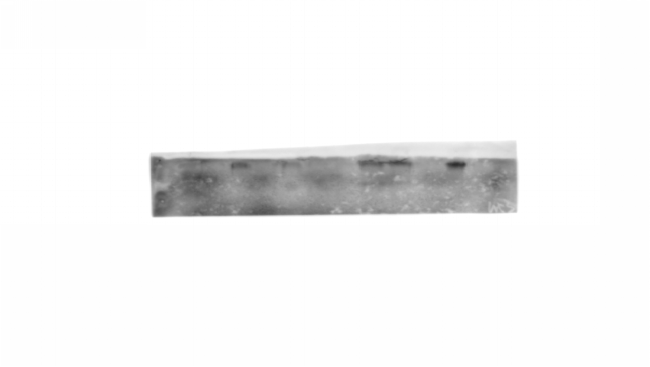

Supplement: Figure 8—figure supplement 1—source data 2. [file elife-102277-fig8-figsupp1-data2.zip › Figure 8—figure supplement 1-source data 2/Figure 8—figure supplement 1E-source data 2/UBR5.tif]
